# C1‐4 Alkylation of Aryl Bromides with Light Alkanes enabled by Metallaphotocatalysis in Flow

**DOI:** 10.1002/anie.202413846

**Published:** 2024-10-25

**Authors:** Antonio Pulcinella, Prakash Chandra Tiwari, Alberto Luridiana, Ken Yamazaki, Daniele Mazzarella, Akshay K. Sadhoe, Antonella Ilenia Alfano, Eveline H. Tiekink, Trevor A. Hamlin, Timothy Noël

**Affiliations:** ^1^ Flow Chemistry Group Van't Hoff Institute for Molecular Sciences (HIMS) University of Amsterdam Science Park 904 1098 XH Amsterdam The Netherlands; ^2^ Dipartimento di Scienze Chimiche e Geologiche Università degli Studi di Cagliari S.S. 554, bivio per Sestu 09042 Monserrato CA Italy; ^3^ Department of Chemistry and Pharmaceutical Sciences AIMMS Vrije Universiteit Amsterdam De Boelelaan 1108 1081 HZ Amsterdam The Netherlands; ^4^ Division of Applied Chemistry Okayama University Tsushimanaka 700-8530 Okayama Japan; ^5^ Department of Chemical Sciences University of Padova Via Francesco Marzolo 1 35131 Padova Italy

**Keywords:** Light Alkanes, Photocatalysis, Nickel Catalysis, Flow Chemistry, DFT Calculations

## Abstract

The homologous series of gaseous C1‐4 alkanes represents one of the most abundant sources of short alkyl fragments. However, their application in synthetic organic chemistry is exceedingly rare due to the challenging C−H bond cleavage, which typically demands high temperatures and pressures, thereby limiting their utility in the construction of complex organic molecules. In particular, the formation of C(sp^2^)−C(sp^3^) bonds is crucial for constructing biologically active molecules, including pharmaceuticals and agrochemicals. In this study, we present the previously elusive coupling between gaseous alkanes and (hetero)aryl bromides, achieved through a combination of Hydrogen Atom Transfer (HAT) photocatalysis and nickel‐catalyzed cross coupling at room temperature. Utilizing flow technology allowed us to conduct this novel coupling reaction with reduced reaction times and in a scalable fashion, rendering it practical for widespread adoption in both academia and industry. Density Functional Theory (DFT) calculations unveiled that the oxidative addition constitutes the rate‐determining step, with the activation energy barrier increasing with smaller alkyl radicals. Furthermore, radical isomerization observed in propane and butane analogues could be attributed to the electronic properties of the bromoarene coupling partner, highlighting the crucial role of oxidative addition in the observed selectivity of this transformation.

## Graphical abstract

Herein, we report a protocol for the introduction of short alkyl fragments on aryl bromides using gaseous alkanes. The major advancement in this study is the possibility to bypass the use of prefunctionalized coupling partners, often synthesized in the bulk chemical industry from the parent alkane through non‐selective and energy‐intensive processes. Additionally, DFT calculations and mechanistic studies unveiled the factors governing the selectivity of the transformation, offering a blueprint for future reaction development in the field of metallaphotocatalytic C(sp^3^)−H functionalization.

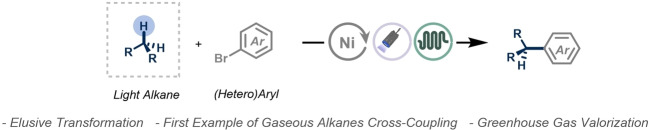

## Introduction

**Figure 1:**
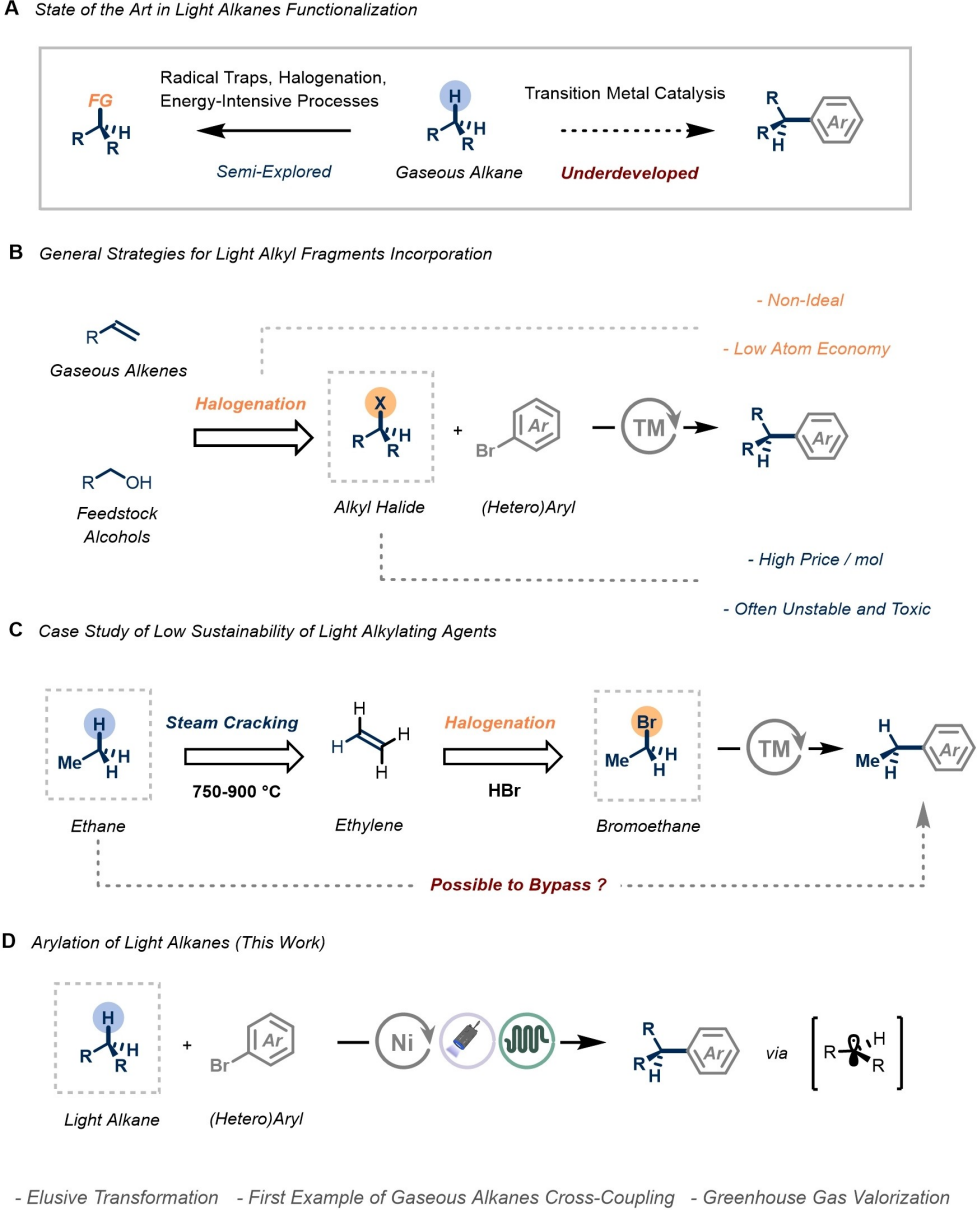
**A** State of the art in gaseous alkane functionalization. **B** General strategies for light alkyl fragment incorporation **C** Ethylation, case study. **D** Design of an efficient arylation of light hydrocarbons.

In recent years, the direct and selective conversion of alkanes into fine chemicals has garnered significant attention within the scientific community.[^1^, ^2^] This surge in interest stems from a growing desire to introduce sp^3^‐character into organic molecules, which matches more with the 3D‐nature of protein targets, thereby enhancing their efficacy and reducing attrition rates.[^3^, ^4^] Moreover, direct activation of aliphatic C−H bonds allows practitioners to bypass the use of functionalized molecules, such as haloalkanes or organometallic reagents, enabling the direct use of feedstock materials into the late‐stage modification of drugs, agrochemicals, and materials.[^5^, ^6^] Notably, gaseous hydrocarbons stand out as the most abundant, atom‐efficient, and economically viable carbon‐based feedstocks. Despite their prevalence, the C−H bonds present in these molecules exhibit extraordinary inertness, rendering conventional synthetic methodologies for converting gaseous hydrocarbons into functionalized derivatives often ineffective or lacking in selectivity (Figure 1A).^[7]^ As a consequence, gaseous hydrocarbons are predominantly utilized as energy sources in combustion processes.

In modern synthetic chemistry, arylation reactions serve as indispensable tools for assembling building blocks relevant to medicinal and crop science applications.[^8^, ^9^, ^10^, ^11^, ^12^] Consequently, transition metal‐catalyzed cross‐coupling has emerged as the gold standard for assembling such units, often achieved through the combination of prefunctionalized starting materials, including aryl or alkyl halides and organometallic reagents.^[13]^ While these prefunctionalized fragments offer high chemofidelity, they also come with drawbacks such as low atom economy, high toxicity, and often moisture and air instability. In the industrial preparation of these building blocks, main routes involve the halogenation of either the corresponding olefin with hydrohalic acids or the conversion of the corresponding alcohol (Figure 1B).[^14^, ^15^]

To illustrate, considering the introduction of an ethyl unit via conventional cross‐coupling, the apparent C2 synthon is bromoethane or species derived from it (e.g., organoboron, organozinc reagents).[^11^, ^12^, ^13^] Intriguingly, bromoethane derives from the hydrobromination of ethylene, which in turn originates from the energy‐intensive steam cracking of naturally occurring ethane (Figure 1C).[^15^, ^16^, ^17^]

Consequently, these drawbacks have spurred growing interest within the scientific community in implementing new strategies capable of utilizing more sustainable and abundant alkylating agents, ideally directly from these alkane feedstocks.^[2]^ While the direct introduction of alkyl fragments (C1‐4) into aryl halides through C−H functionalization of light alkanes is theoretically the ideal approach,^[18]^ such transformation remains elusive and restricted to few examples to date, employing limited coupling partners. For instance, the first reported example by Nelson et al. exploited the generation of stabilized aryl cations from *β*‐silylated aryl fluorides, capable of undergoing C−H insertion with methane.^[19]^ In a mechanistically distinct approach from the Chang group, a copper‐catalyzed cross‐dehydrogenative coupling of propane and butane with activated perfluoroarenes led to the introduction of the desirable light alkyl units.^[20]^ However, as a generalization, the utilization of abundant gaseous alkanes as alkylating agents in transition metal catalysis has not seen significant methodological advancement and lags behind more conventional cross‐coupling technologies that employ prefunctionalized coupling partners.

Motivated by this gap in the literature, we aimed to develop a practical catalytic method to achieve the C(sp^3^)−H arylation of light hydrocarbons, thus providing a synthetic strategy for incorporating light alkyl fragments into (hetero)arenes (Figure 1D). Specifically, we envisioned that the combination of photoinduced hydrogen atom transfer (HAT), nickel catalysis, and continuous‐flow reaction conditions would facilitate the realization of such a C(sp^2^)−C(sp^3^) cross‐coupling platform.[^21^, ^22^] The primary advancement presented in this study encompasses the ability to circumvent the use of prefunctionalized alkyl derivatives, often synthesized from the parent alkane via energy‐intensive and non‐selective processes, which feature low sustainability profiles. Additionally, to address limitations associated with gas‐to‐liquid mass transfer and the safety profile of handling gaseous reagents in conventional reactors, we propose the utilization of flow technology to ensure safe and rapid processing.[^23^, ^24^] Furthermore, the integration of high‐intensity light sources and micro capillary reactors ensures uniform irradiation and short reaction times, making the overall protocol synthetically useful and scalable. Finally, comprehensive Density Functional Theory (DFT) calculations have been conducted, identifying the oxidative addition step as the most challenging aspect of this reaction blueprint, thereby offering valuable insights for future catalyst development.

## Results and Discussion

### Reaction Optimization

Our optimization efforts commenced by reacting aryl bromide **1** with propane, employing a synergistic combination of Ni complex **I** and tetrabutylammonium decatungstate (TBADT) as the Hydrogen Atom Transfer photocatalyst (Table 1). CD_3_CN was selected as the solvent due to TBADT′s well‐known propensity to abstract hydrogen atoms from CH_3_CN under the demanding conditions necessary to activate gaseous reagents.^[25]^ A gas‐to‐liquid ratio (G : L) of 20 : 1 (8 equiv.) was employed, with 2,6‐lutidine used to quench the hydrobromic acid formed during the reaction. The solution was introduced into a continuous‐flow microreactor (ID=0.5 mm; 5 mL volume) and irradiated with UV‐A light (Chip‐on‐board LED, λ=365 nm; 144 W optical power) (See Supporting Information, Section 2.3).

**Table 1:** Optimization of the alkylation of 1 with propane.

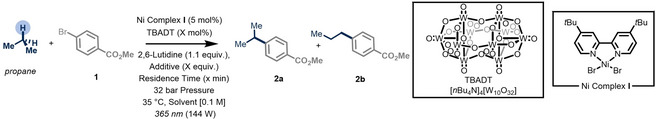						
Entry	Res. Time (minutes)	G : L (V/V)	TBADT (X mol %)	Additive (1.5 equiv.)	Solvent	Yield **2 a**+**2 b** ^[a,b]^
1	15	20 : 1	1	–	CD_3_CN	33 %
2	30	20 : 1	1	–	CD_3_CN	33 %
3	15	40 : 1	1	–	CD_3_CN	40 %
4	15	60 : 1	1	–	CD_3_CN	39 %
5	15	40 : 1	2.5	–	CD_3_CN	42 %
6	15	40 : 1	2.5	LiBr	CD_3_CN	44 %
7	15	40 : 1	2.5	LiBr	CD_3_CN 3 : 1 tBuOH	54 %
8	30	40 : 1	2.5	LiBr	CD_3_CN 3 : 1 tBuOH	55 %
9	30+20	40 : 1	2.5	LiBr	CD_3_CN 3 : 1 tBuOH	56 %
10^[c]^	30+20	40 : 1	2.5+2.5	LiBr	CD_3_CN 3 : 1 tBuOH	60 %^[d]^

[a] Reactions performed on a 0.3 mmol scale, 144 W of 365‐nm LEDs. [b] Yields determined by ^1^H NMR using trichloroethylene as an external standard. [c] A fresh solution of TBADT 2.5 mol % in 1.5 mL CD_3_CN was added before the second run. [d] **2 a**/**2 b** ratio determined to be 2 : 1. See Supporting Information for experimental details.

By utilizing a back pressure regulator, we were able to elevate the reaction pressure to 32 bar, effectively liquefying the gaseous alkanes and thereby enhancing the probability of their activation through photocatalytic HAT. Under these conditions, a residence time of only 15 minutes (entry 1) was found to be sufficient for detecting the formation of the desired product **2** in a synthetically useful yield with a regioisomeric mixture of 1.2 : 1 (**2 a** : **2 b**). Extending the residence time to 30 minutes (entry 2) did not have a positive impact on the chemical efficiency. However, increasing the G : L to 40 : 1 (16 equiv.), and 60 : 1 (24 equiv.), (entries 3 and 4, respectively) reflected into an increase in yield to 40 %. It is important to note that an excess of gas reagent should not pose any problem, as it can be easily recovered and recycled after the reaction by simply reducing pressure to atmospheric pressure, thus ensuring phase separation between the gas and liquid phases. Increasing the TBADT loading to 2.5 mol % (entry 5) led to a further rise in the chemical efficiency.

At this stage of the optimization, several ligands for the Ni catalyst were screened, with 4,4′‐di‐*tert*‐butyl‐2,2′‐dipyridyl confirming its superior activity (See Supporting Information, Section 4). An assessment of several reaction additives and solvent mixtures highlighted the positive impact of LiBr and *t*BuOH (entries 6 and 7). Although extending the residence time to 30 minutes (entry 8) and implementing a recirculation system to increase reaction times even further to 50 minutes (entry 9) resulted in only a minor increase in chemical efficiency, the introduction of a fresh solution of TBADT before the recirculation step (entry 10, See Supporting Information, Section 4.6) furnished the desired compound **2** in a 60 % yield with a regioisomeric mixture of 2 : 1 (**2 a** : **2 b**).

### Substrate Scope

**Figure 2:**
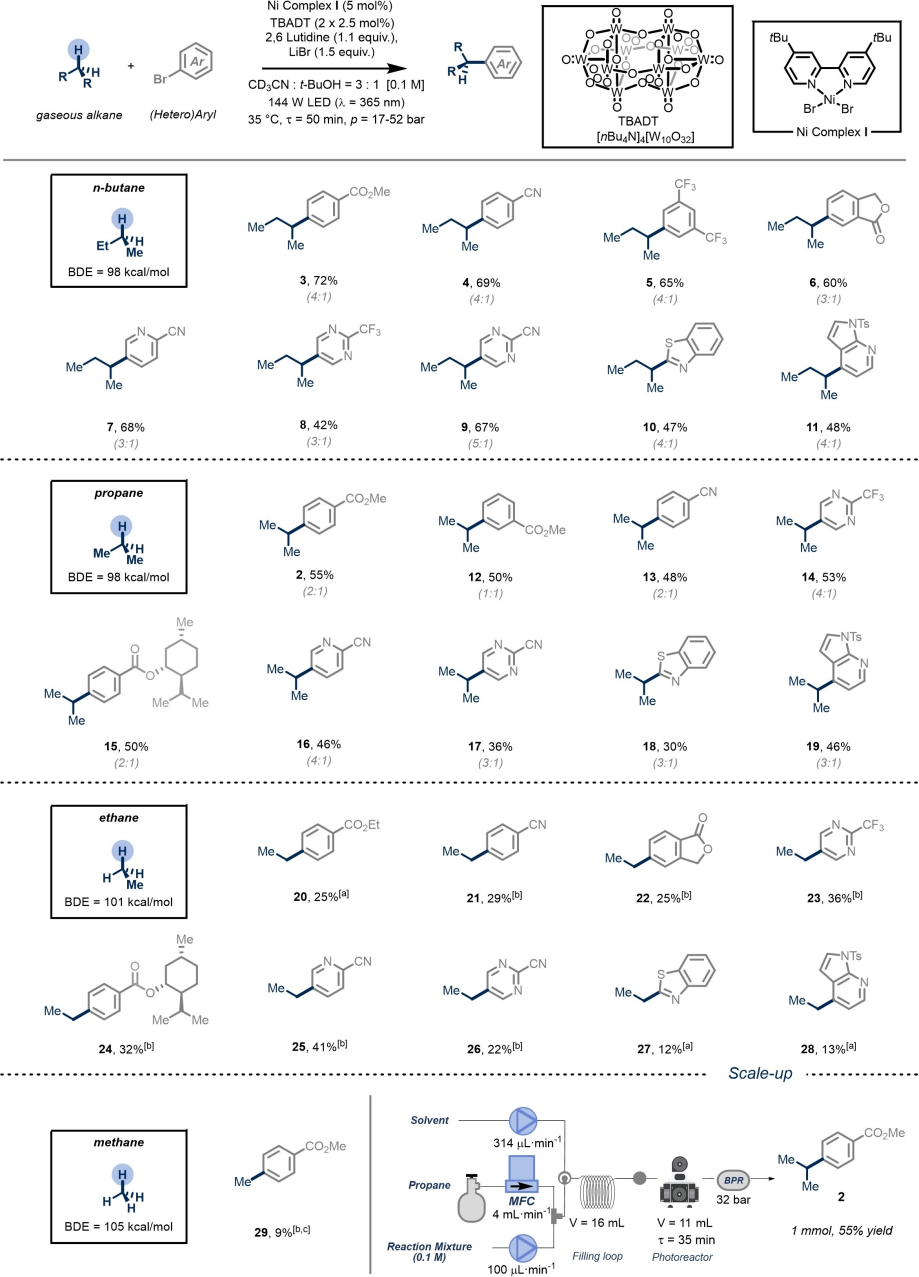
Scope of the alkylation of (hetero)aryl bromides using gaseous alkanes as alkylating agents. Reaction conditions: (hetero)aryl bromide (0.3 mmol. 1.0 equiv.), TBADT (2×2.5 mol %), Ni Complex **I** (5 mol %), 2,6 Lutidine (1.1 equiv.) and LiBr (1.5 equiv.) in 3 mL of (CD_3_CN: t‐BuOH=3 : 1), G : L=40 : 1, 144 W of 365‐nm LEDs. Selectivity reported in brackets. All yields are those of isolated products (Supporting Information for experimental details). [a] For the recirculation step TBADT (2.5 mol %) and Ni Complex **I** (5 mol %) were used. [b] For the recirculation step TBADT (3.5 mol %) and Ni Complex **I** (7 mol %) were used. [c] Yield determined by GC‐FID. See Supporting Information for experimental details.

After establishing the general reaction conditions and optimizing the design of the photochemical reactor, we explored the scope and generality of the C(sp^3^)−H (hetero)arylation of light alkanes with aryl bromides in our flow reactor platform (Figure 2). Notably, the utilization of flow technology facilitates the seamless execution of these reactions by harnessing flow energy in combination with back pressure regulators to apply pressure, while also enabling a continuous feed into the reactor to generate substantial quantities efficiently without having to reoptimize the reaction conditions (Figure 2). Additionally, due to the straightforward setup of the reactor platform, expansion of the substrate scope can be swiftly accomplished.

Diverse gaseous alkanes, including butane, propane, ethane, and methane, could be coupled with various aryl bromides efficiently. Notably, pharmaceutically relevant heteroaryl bromides, such as pyridine, pyrimidine, benzothiazole, and azaindole, with different electronic properties and ring sizes, could be alkylated in synthetically useful yields. Also, a menthol ester derivative, bearing many C(sp^3^)−H bonds with lower bond dissociation energy compared to gaseous alkanes, could be alkylated in synthetically useful yields (**15** & **24**, 32–50 % yield). Overall, our results demonstrate that *n*‐butane and propane yielded the desired alkylated analogues in moderate to good yields. In contrast, ethane and methane, which possess significantly higher bond dissociation energies (101 and 105 kcal/mol, respectively), produced lower yields. However, these yields are still considered synthetically valuable. One consideration of this methodology is that aryl bromides with strong electron‐donating groups (e.g., Ar−Br with OMe or NR_2_ substituents) did not undergo the desired reactivity.

### Mechanistic Investigation

While synthetically useful amounts were obtained for every single gaseous alkane, we observed reduced efficiency in the coupling reaction as the bond dissociation energies (BDEs) for the corresponding C−H scission increased.[^26^, ^27^] Additionally, for propane and butane as coupling partners, we noted a selectivity erosion upon radical capture by nickel compared to the direct reaction of nucleophilic alkyl radicals with Michael acceptors (Figure 2).[^25^, ^28^, ^29^] To explain these observed trends and gain further insights into the mechanistic rationale of the metallaphotocatalytic cross‐coupling of aryl bromides with gaseous alkanes, we conducted a detailed DFT calculation study and carried out some additional mechanistic experiments to support the computational observations.

**Figure 3:**
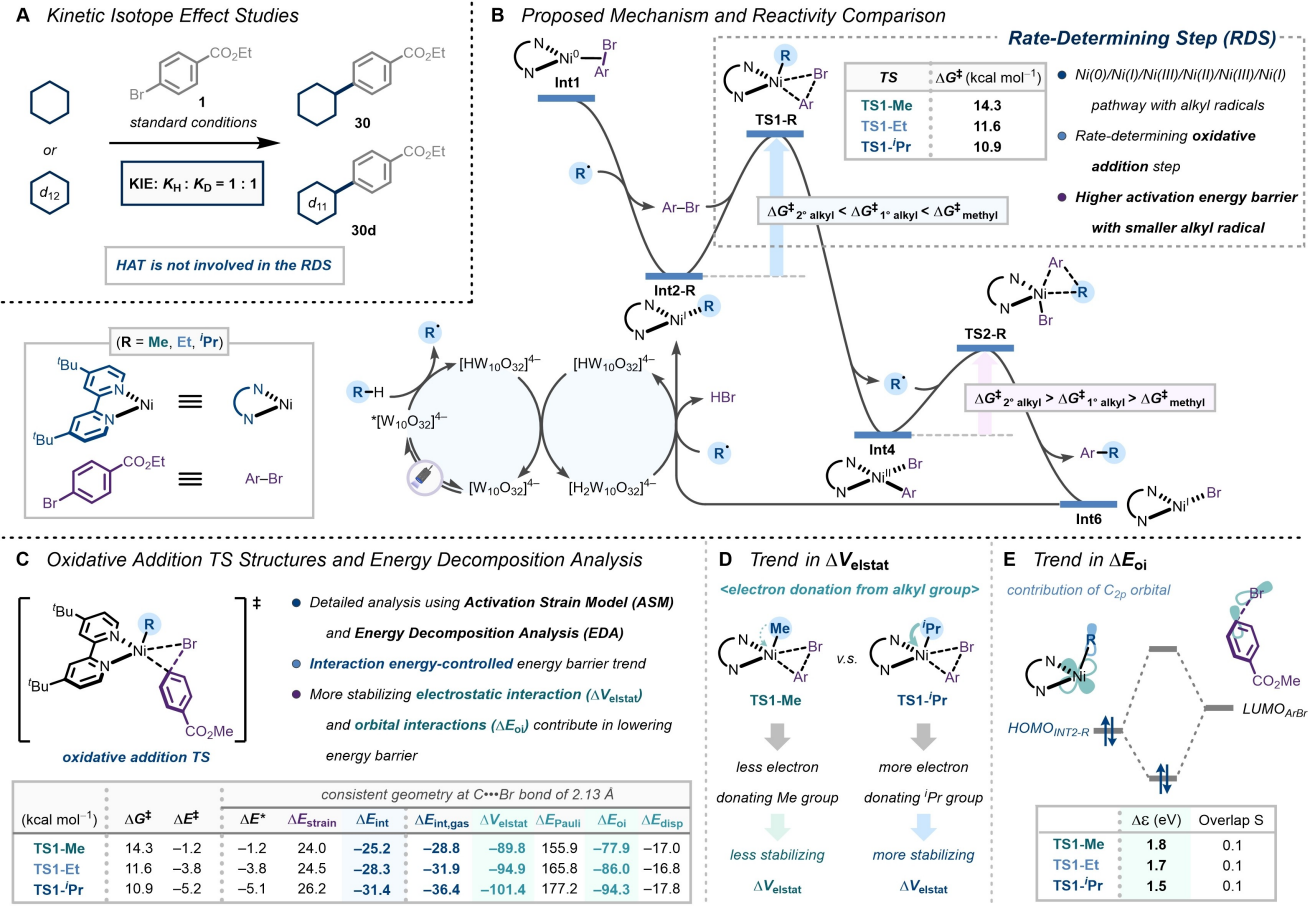
**A** Parallel kinetic isotope effect. **B** Proposed reaction mechanism and the reactivity comparison between methyl, ethyl, and iso‐propyl radicals. **C** The activation strain analysis (ASA) and the energy decomposition analysis (EDA) of rate‐determining oxidative addition TS‐like structures. **D** Origin of the trend in electrostatic interaction energies. **E** Origin of the trend in orbital interactions.

Specifically, we conducted a parallel kinetic isotope effect (KIE) study using cyclohexane as a substrate (Figure 3A). We observed a negligible reaction rate difference between cyclohexane and its deuterated substrate, indicating that the HAT process by a photoexcited decatungstate is not involved in the rate‐determining step (RDS) in our reaction conditions. With these kinetic data in hand and based on recent computational work of Gutierrez and Molander on a related reaction system with tertiary alkyl radicals,^[30]^ we investigated the reactivity trends with various alkyl radicals from gaseous alkanes using state‐of‐the‐art DFT calculations (Figure 3B and Figure S8 Supporting Information).[^31^, ^32^] The reaction begins with the generation of an alkyl radical by the HAT process between the gaseous alkane and a photoexcited decatungstate anion (*[W_10_O_32_]^4−^), and subsequent radical coordination to Ni(0) species **Int1** to furnish a stable Ni(I) intermediate **Int2‐R**. In turn, Ni(I) undergoes oxidative addition to the aryl halide through the transition state **TS1‐R** that leads to the formation of a pentacoordinate Ni(III) intermediate, which is then followed by an alkyl radical dissociation to furnish a square planar Ni(II) intermediate **Int4**. Upon the radical re‐coordination, the ligand orientation of the Ni(III) intermediate is primed for the formation of a C(sp^2^)−C(sp^3^) bond via the reductive elimination through transition state **TS2‐R**. The final Ni(I)−Br intermediate **Int6** is then regenerated as **Int2‐R** by protonation and alkyl radical addition. Throughout the catalytic cycle, we found that the RDS is the oxidative addition, and more interestingly, the activation energy barrier for this step increases with smaller alkyl radicals, whereas the reductive elimination follows the opposite trend.

To gain deeper insight into the origin of reactivity differences observed for the various radicals, we turned to the activation strain model (ASM) and our energy decomposition analysis (EDA). We focused on the analysis of the rate‐determining oxidative addition step (Figure 3C). The ASM involves the decomposition of the electronic energy Δ*E* into the strain energy Δ*E*_strain_ associated with the structural deformation of the Ni(I)‐alkyl complex and the aryl halide **1** from their equilibrium geometry and the interaction energy Δ*E*_int_ between these deformed reactants [Eq. 1].[^33^, ^34^, ^35^, ^36^] Our matching EDA separates the interaction energy (Δ*E*_int_) into the following four chemically meaningful energy terms: classical electrostatic interaction (Δ*V*_elstat_), destabilizing steric Pauli repulsion interactions (Δ*E*_Pauli_), stabilizing orbital interactions that account, among others, for HOMO−LUMO interactions (Δ*E*_oi_), and the dispersion interaction (Δ*E*_disp_) between the deformed Ni(I)‐alkyl complex and aryl halide **1** [Eq. 2].[^37^, ^38^, ^39^]
(1)

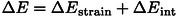



(2)

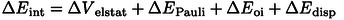




The oxidative addition with the methyl radical goes with the highest reaction barrier (Δ*G* ^≠^=14.3 kcal mol^−1^), and the energy decreases as the number of substituents of the radical increases (Δ*G* ^≠^=11.6 kcal mol^−1^ [R=Et], and 10.9 kcal mol^−1^ [R=^*i*^Pr]).

We performed our activation strain analysis (ASA) at the same point on the reaction coordinate near all transition states with a C(aryl)−Br stretch of 2.13 Å to account for the shift in the transition state position which has been previously shown to skew results.^[40]^ The trend in Gibbs free energy activation barriers is the same as for the electronic activation energy barriers (ΔE*). The differences in reactivity can be traced to the differences in the interaction energy, while the strain energies are nearly the same. The higher activation barrier for the methyl radical originates from a less stabilizing interaction energy (Δ*E*_int_=−25.2 kcal mol^−1^), whereas the lower barrier for the ^*i*^Pr radical benefits from a much more stabilizing interaction energy (Δ*E*_int_=−31.4 kcal mol^−1^). Using our EDA method, we traced this trend back to differences in both the electrostatic interactions (Δ*V*_elstat_) and orbital interactions (Δ*E*_oi_). The origin of the less stabilizing Δ*V*_elstat_ in **TS1‐Me** compared to **TS1‐** ^***i***^ **Pr** is that the less electron‐donating methyl radical does not effectively increase the electronegativity at the nickel center during the oxidative addition transition state, resulting in the increasing the activation energy barrier (Figure 3D). In addition, the HOMO orbital of Ni(I) species is strongly affected by the contribution from the 2p orbital of the adjacent alkyl substituent, where the HOMO−LUMO gap Δϵ with aryl bromide is larger for the less electron‐donating methyl group (Δϵ=1.8 eV) and the gap is much smaller and favorable for the bulkier and more electron donating ^*i*^Pr group (Δϵ=1.5 eV) (Figure 3E). These observations, along with the potential catalyst deactivation pathway with smaller alkyl radicals (See Supporting Information, Figure S9), shed light on the origin of decreased reactivity in the coupling reaction of aryl bromides with methane.

**Figure 4:**
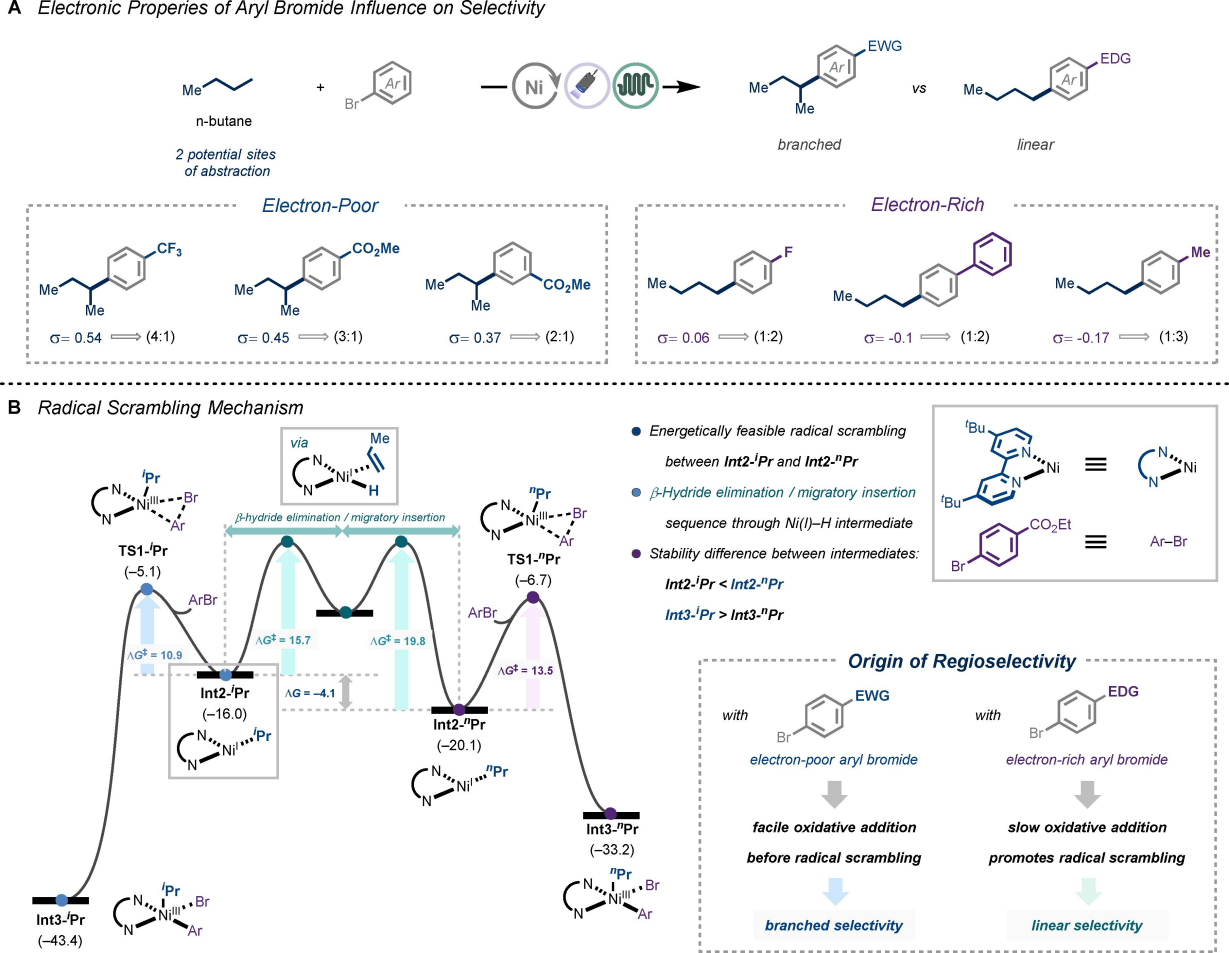
**A** Influence of the electronic property of aryl bromides on the selectivity of alkylation using n‐butane. Selectivity determined with GC‐MS. [a] Yields determined by ^1^H NMR using trichloroethylene as an external standard. [b] Volatile compounds. **B** Radical scrambling mechanism and the origin of regioselectivity. See Supporting Information for experimental details.

As described above, we observed selectivity erosion upon radical capture for the coupling reaction with propane and butane, and the selectivity is influenced by the aryl bromide coupling partners (Figures 4A, and Figures S10, and S11, Supporting Information). When an electron‐poor aryl bromide was used, the branched alkylation was a major pathway, whereas the selectivity was inverted with an electron‐rich aryl bromide, and then the linear pathway became dominant. To elucidate the origin of this regioselectivity, we again turned to DFT calculations to investigate the alkyl radical isomerization mechanism using propyl radical as a model system (Figure 4B). Initially, **Int2‐** ^***i***^ **Pr** is formed by the HAT process with a photoexcited decatungstate anion and a radical coordination to the nickel center. This intermediate could undergo β‐hydride elimination to generate Ni(I)−H intermediate **Int7**, and upon further migratory insertion of

propene leads to a more thermodynamically stable Ni(I) species **Int2‐** ^***n***^ **Pr**. Interestingly, the energy barriers for this isomerization process are energetically feasible and similar to that of the oxidative addition. This suggests that the oxidative addition step not only determines the reaction rate but also has a major influence on the selectivity. Electron‐poor aryl bromides can undergo facile oxidative addition before radical isomerization thus, the branched selectivity dominates the reaction. On the other hand, electron‐rich aryl bromides are generally less reactive because of the increased energy barrier at the RDS, and radical scrambling is more likely to occur to furnish linear selectivity.

## Conclusion

Herein, we demonstrate for the first time a practical cross‐coupling strategy between (hetero)aryl bromides and gaseous alkanes through a combination of hydrogen atom transfer photocatalysis and nickel catalysis. The use of flow technology was pivotal in offering precise control over reaction conditions, facilitating easy reaction optimization, rapid scope expansion, and seamless scalability. DFT studies enabled us to understand the origins of unique experimental observations, pinpointing the oxidative addition as the rate‐ and selectivity‐determining step. Furthermore, this integrated approach provides a framework for future developments in utilizing gaseous alkanes as reaction partners in various coupling reactions.

## Supporting Information

The authors have cited additional references within the Supporting Information.[^1^, ^38^]

## Author Contribution

T. N. conceived the initial idea for the project. A. P., P. T, A. L., D. M., A. S. and I. A. performed and analyzed the experiments. K. Y., E. H. T. and T. A. H. carried out the DFT calculations and analysis. T. N. directed and supervised the project, with regular scientific input from all authors. A. P., T. A. H. and T. N. wrote the manuscript with comments from all the other authors.

## Conflict of Interests

The authors declare no conflict of interest.

1

## Supporting information

As a service to our authors and readers, this journal provides supporting information supplied by the authors. Such materials are peer reviewed and may be re‐organized for online delivery, but are not copy‐edited or typeset. Technical support issues arising from supporting information (other than missing files) should be addressed to the authors.

Supporting Information

## Data Availability

The data that support the findings of this study are available in the supplementary material of this article.
