# Supplementary material for: C1‐4 Alkylation of Aryl Bromides with Light Alkanes enabled by Metallaphotocatalysis in Flow
Source: Angew Chem Int Ed Engl. 2024 Oct 25;64(2):e202413846. doi: 10.1002/anie.202413846 (PMC11720381; doi:10.1002/anie.202413846)
Supplement: Supplementary file 1 — Supporting Information [file ANIE-64-e202413846-s001.pdf]

Angewandte Chemie
Eine Zeitschrift der Gesellschaft Deutscher Chemiker

Supporting Information

C1-4 Alkylation of Aryl Bromides with Light Alkanes enabled by Metallaphotocatalysis in Flow

A. Pulcinella, P. Chandra Tiwari, A. Luridiana, K. Yamazaki, D. Mazzarella, A. K. Sadhoe, A. I. Alfano, E. H. Tiekink, T. A. Hamlin, T. Noël**

Supplementary Information

C1-4 Alkylation of Aryl Bromides with Light Alkanes enabled by Metallaphotocatalysis in Flow

Antonio Pulcinella,[a],† Prakash Chandra Tiwari,[a],† Alberto Luridiana,[a],[b],† Ken Yamazaki,[c],[d] Daniele Mazzarella,[a],[e] Akshay K. Sadhoe,[a] Antonella Ilenia Alfano,[a] Eveline H. Tiekink,[c] Trevor A. Hamlin,[c]* and Timothy Noël[a]*

[a] Antonio Pulcinella, Prakash Chandra Tiwari, Dr. Alberto Luridiana, Dr. Daniele Mazzarella, Akshay Sadhoe, Antonella Ilenia Alfano and Prof. Dr. Ing. Timothy Noël
Flow Chemistry Group, Van 't Hoff Institute for Molecular Sciences (HIMS), University of Amsterdam, Science Park 904, 1098 XH, Amsterdam, The Netherlands.
E-mail: t.noel@uva.nl

[b] Dr. Alberto Luridiana
Dipartimento di Scienze Chimiche e Geologiche Università degli Studi di Cagliari, S.S. 554, bivio per Sestu, Monserrato (CA), Italy.

[c] Dr. Ken Yamazaki, Eveline H. Tiekink and Prof. Dr. Trevor A. Hamlin
Department of Chemistry and Pharmaceutical Sciences, AIMMS, Vrije Universiteit Amsterdam, De Boelelaan 1108, 1081 HZ, Amsterdam, The Netherlands.
E-mail: t.a.hamlin@vu.nl

[d] Dr. Ken Yamazaki
Division of Applied Chemistry, Okayama University, Tsushimanaka, Okayama 700-8530, Japan.

[e] Dr. Daniele Mazzarella
Department of Chemical Sciences, University of Padova, Via Francesco Marzolo 1, 35131, Padova, Italy.

† These authors contributed equally

**Email:* t.a.hamlin@vu.nl (T. A. Hamlin) and t.noel@uva.nl (T. Noël)

This PDF file includes:

Materials and Methods

Figures S1 to S11

Tables S1 to S9

NMR Data

References

Table of contents

1. General information ... 3
2. Reactor Design ... 4
   2.1 Flow Equipment ... 4
   2.2 Vapourtec Setup ... 5
   2.3 Eagle Reactor ... 6
3. General Procedure for the Optimization of gas-liquid Reactions in Flow ... 8
4. Reaction Optimization ... 9
   4.1 Photoreactor Evaluation ... 9
   4.2 Catalysts loading Screening ... 10
   4.3 Ligand Screening ... 11
   4.4 Gas to Liquid Ratio Screening ... 12
   4.5 Additive Screening ... 13
   4.6 Recirculation ... 14
   4.7 Optimization for Ethane ... 15
5. General Procedures (GP) ... 16
   5.1 (GP1) *n*-Butane ... 16
   5.2 (GP2) Propane ... 16
   5.3 (GP3) Ethane ... 17
   5.4 (GP4) Methane ... 17
   5.5 Scale-up ... 17
6. Characterization data of synthesized compound ... 19
7. Mechanistic experimental studies ... 30
   7.1 Evaluation of the KIE ... 30
   7.2 Evaluation of the selectivity: Arylation of n-butane ... 31
8. Computational Studies ... 33
   8.1 Computational Methods ... 33
   8.2 Computational Results ... 34
   8.3 Computational Details ... 37
9. NMR Spectra of Compounds ... 79
10. References ... 111

1. General information

Materials. All reagents and solvents were used as received without further purification. Reagents and solvents were bought from Sigma Aldrich, TCI and Fluorochem. Technical solvents were bought from VWR International and used as received. N-butane gas with 3.5purity was purchased from Praxair, propane gas with 2.5purity was purchased from Benegas, ethane gas with 3.5purity was purchased from Gerling and Holz and Co and methane gas with 4.5purity was purchased from Nippon gases. Disposable syringes were purchased from Laboratory Glass Specialist. Syringe pumps were purchased from Chemix Inc. model Fusion 200 Touch. All capillary tubing, microfluidic fittings and Back Pressure Regulator (BPR) were purchased from IDEX Health & Science. Product isolation was performed automatically, by a Biotage® Isolation Four, with Biotage® SNAP KP-Sil 4 or 10 g flash chromatography cartridges, or manually, using silica (P60, SILICYCLE). TLC analysis was performed using Silica on aluminum foils TLC plates (F254, SILICYCLE) with visualization under ultraviolet light (254 nm and 365 nm) or appropriate TLC staining (potassium permanganate). Organic solutions were concentrated under reduced pressure on a Büchi rotary evaporator (in vacuo at 40 ºC, ~5 mbar). The TBADT catalyst[1] and nickel catalysts[2] were prepared according to reported procedures. The majority of the substrates are commercially available. The synthesis of starting materials 4-bromo-1-tosyl-1H-pyrrolo[2,3-b]pyridine[3] and (1R,2S,5R)-2-isopropyl-5-methylcyclohexyl 4-bromobenzoate[4] were prepared in accordance with literature procedures.

NMR spectroscopy. ^{1}H (400 and 300 MHz), ^{13}C (101 and 128 MHz), ^{19}F (282 and 376 MHz) spectra were recorded at ambient temperature using Bruker AV 300-I, AV 400 and AV 500-NEO. ^{1}H NMR spectra are reported in parts per million (ppm) downfield relative to $CDCl_3$ (7.26 ppm) and all 13C NMR spectra are reported in ppm relative to $CDCl_3$ (77.16 ppm) unless stated otherwise. The multiplicities of signals are designated by the following abbreviations: s (singlet), d (doublet), t (triplet), q (quartet), p (pentet), sext (sextet), m (multiplet), dd (doublet of doublets), dt (doublet of triplets), td (triplet of doublets), ddd (doublet of doublet of doublets). Coupling constants (J) are reported in hertz (Hz). NMR data was processed using the MestReNova 14 software package. Known products were characterized by comparing to the corresponding ^{1}NMR, ^{13}C NMR, ^{19}F NMR with those available in the literature.

Mass spectrometry. High resolution mass spectra (HRMS) were collected on an AccuTOF GC v 4g, JMS-T100GCV Mass spectrometer (JEOL, Japan).

Determination of Regioisomeric and Diastereomeric Ratio. The regioisomeric and diastereomeric ratios were determined by ^{1}H NMR analysis of the crude reaction mixture through integration of diagnostic signals. For cases where the integration of diagnostic signals is not possible, the ratio was determined by GC-FID analysis.

2. Reactor Design

2.1 Flow Equipment

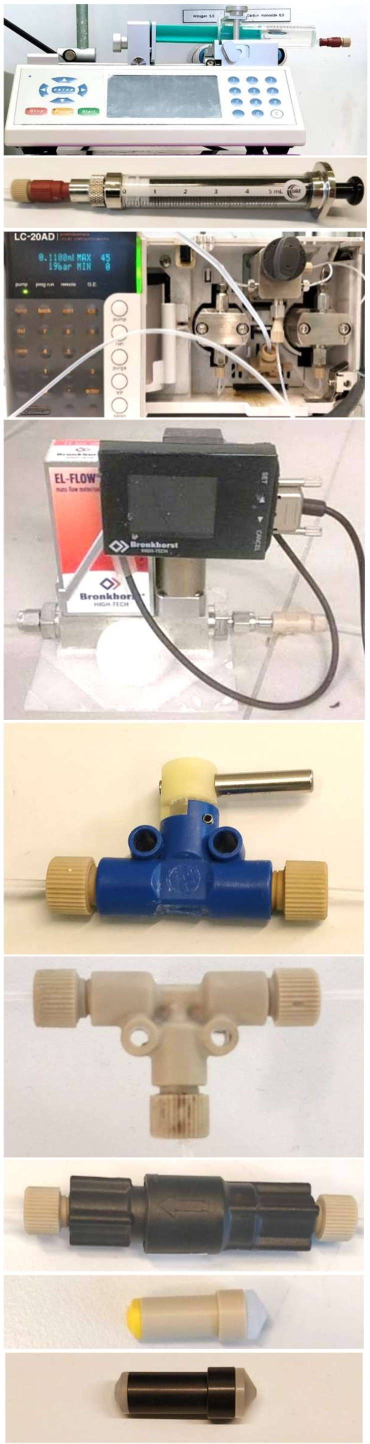

Syringe pump
(Chemyx Fusion 200)

Gastight syringe (SGE
Luer Lock 5)

HPLC pump
(Shimadzu LC-20AD)

Mass Flow Controller
(Bronkhorst EL-)

FLOW - Shut-Off

Valve (IDEX P-783) T-
mixer (IDEX P-712)

BPR holder (IDEX P-
789)

BPR cartridge

(IDEX P-789)

Figure S1: Flow equipment used for the photocatalytic reactions.

2.2 Vapourtec Setup

Preliminary experiments were carried out with a Vapourtec UV-150 photochemical reactor setup, equipped with a a 60 W 365 nm LED lamp (Figure S2).

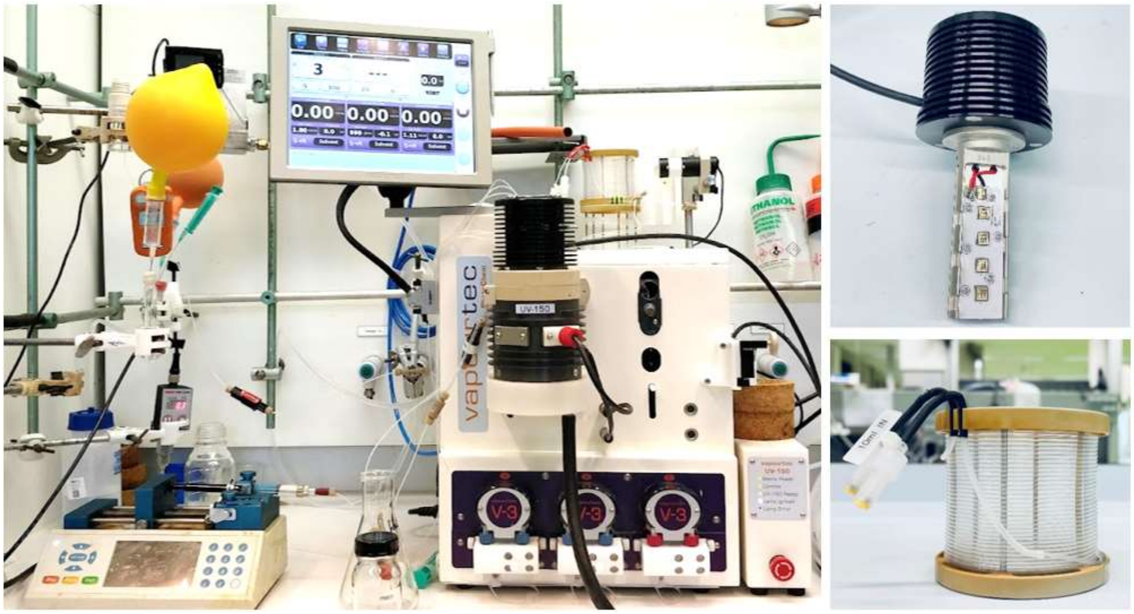

Figure S2: Overview of the Vapourtec Setup and details of the LEDs and PFA reactor coil (1.3 mm ID, 1.6 mm OD, 10 mL).

2.3 Eagle Reactor

A Signify photochemical reactor is used, consisting of a base assembly with six 365 nm UV-A chip-on-board light modules.[5] Each of these light source modules contain a fan and a heat sink to efficiently dissipate heat generated through the high power LEDs. Also the head cap assembly contains blowers to cool the interior of the reactor system, to reduce undesired thermal side-reactions. The LED modules and chamber cooling blowers are connected to a driver box, allowing to set the current of each of the LED modules individually, as well as the rotation speed of the cooling blowers. The six LED modules (365 nm, max. 144 W combined optical output power) are positioned in an hexagonal form around an aluminum cylinder support (80 mm height, 75 mm diameter), which has the reactor coil wrapped around (FEP capillary tubing: 1.6 mm OD, 0.8 mm ID, 11 mL volume or 0.5 mm ID, 2.5 mL volume).

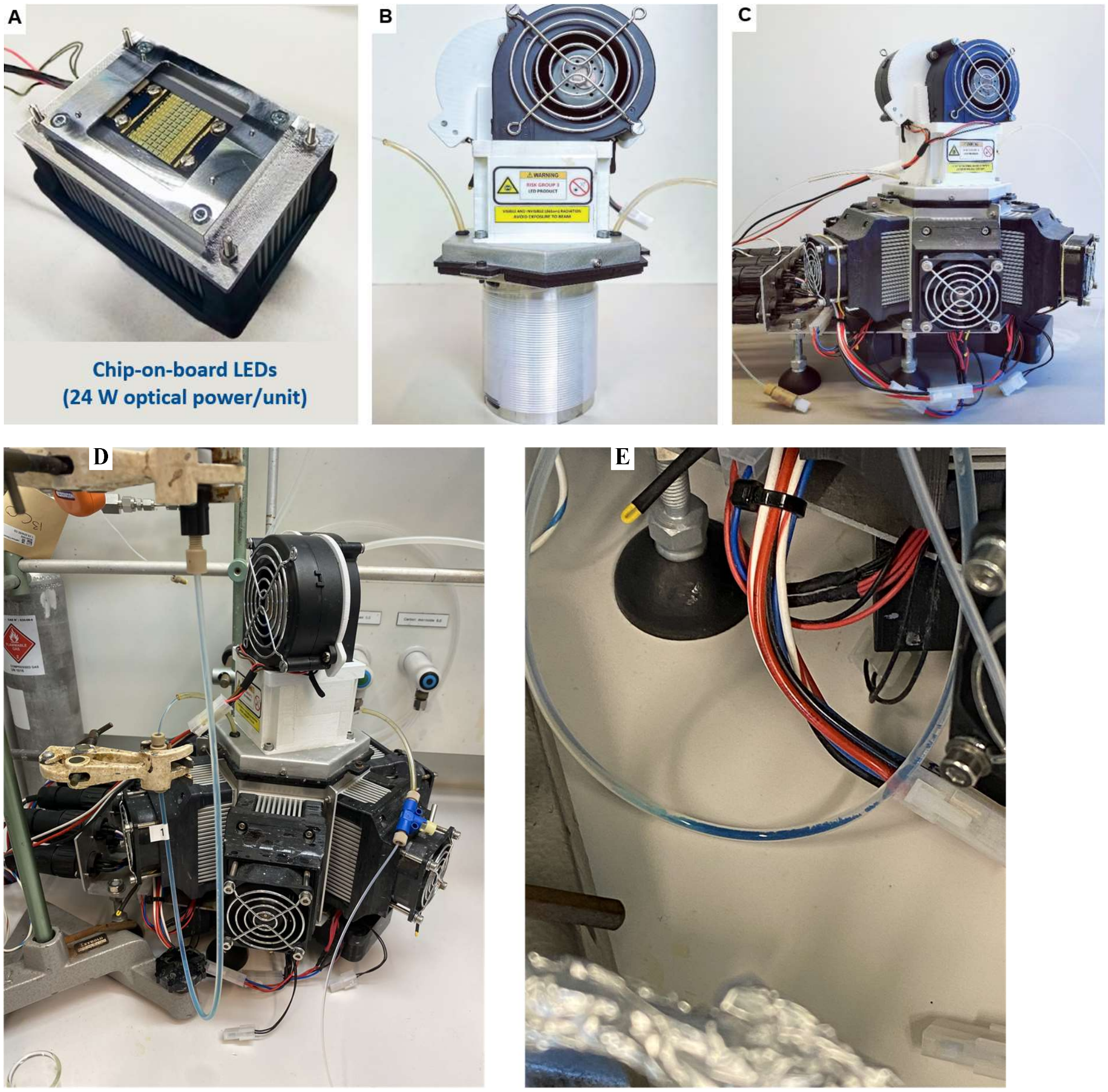

Figure S3 Signify Eagle Reactor with (A) six chip-on-board LED modules, (B) head assembly with reactor coil, and (C) complete assembly with fans, heat sinks and LED modules. (D) and (E) slurry decanter unit.

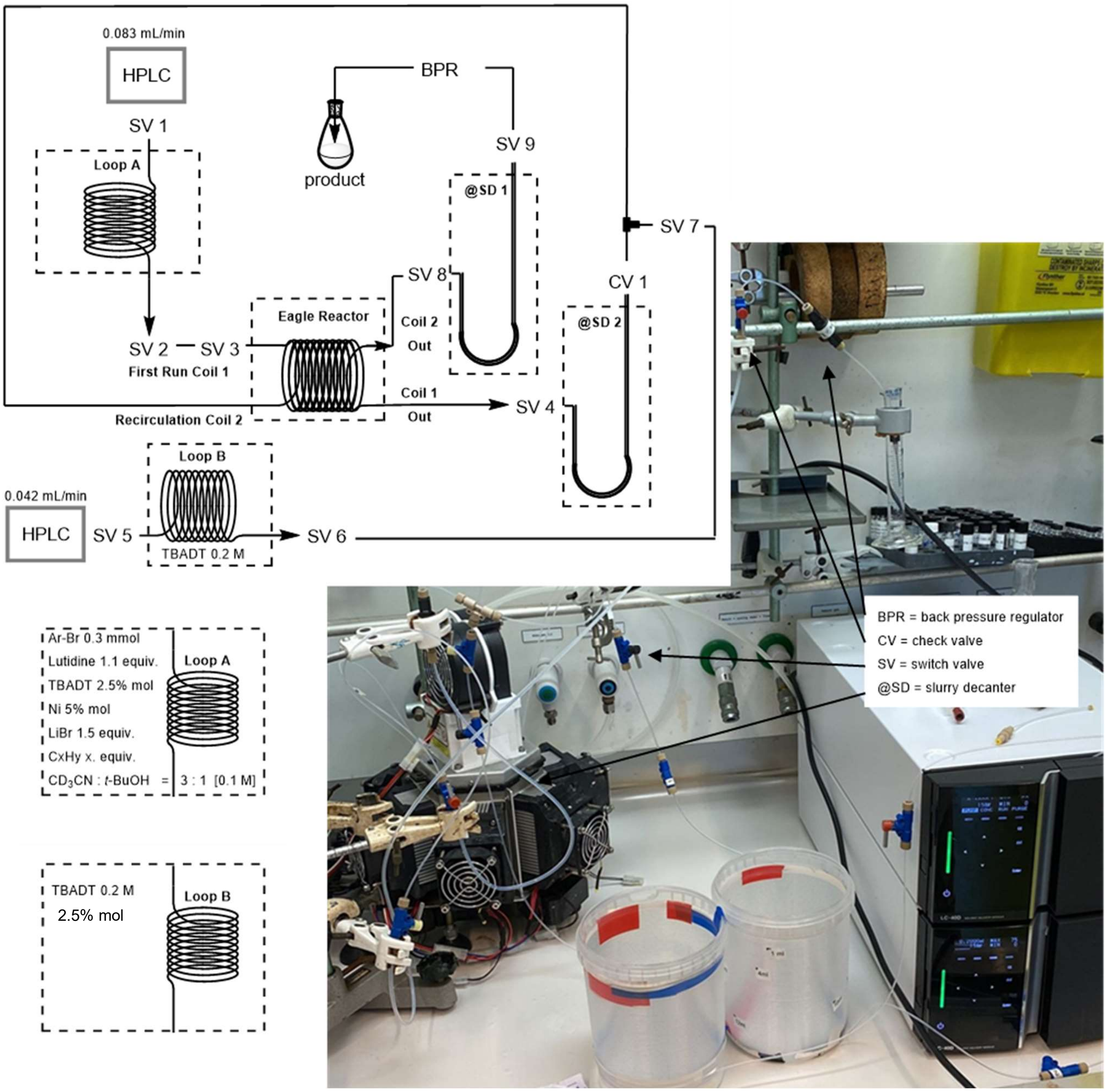

Figure S4: Overview of the Setup used with Signify Eagle Reactor (Loop A contains the reaction mixtures; Loop B contains the photocatalyst used in the recirculation step).

3. General Procedure for the Optimization of gas-liquid Reactions in Flow

An elaborate description of the procedure for optimizing gas-liquid and gas-gas-liquid reactions in flow is described in the supporting information of our previous work on photocatalytic carbonylation of light and heavy hydrocarbons. [6] The relevant descriptions and calculations for this work are repeated here. For reactions above the maximum pressure of the liquid stream (syringe pump) or above the maximum pressure of the gas stream (pressure of the gas cylinder or reducer), a loop filling method is applied (Figure S5 A). With this method, the gas and liquid stream are first combined in a filling loop, then pressurized with a HPLC pump and finally injected into the reactor coil under the desired flow rate with the HPLC pump. (Figure S5 B)

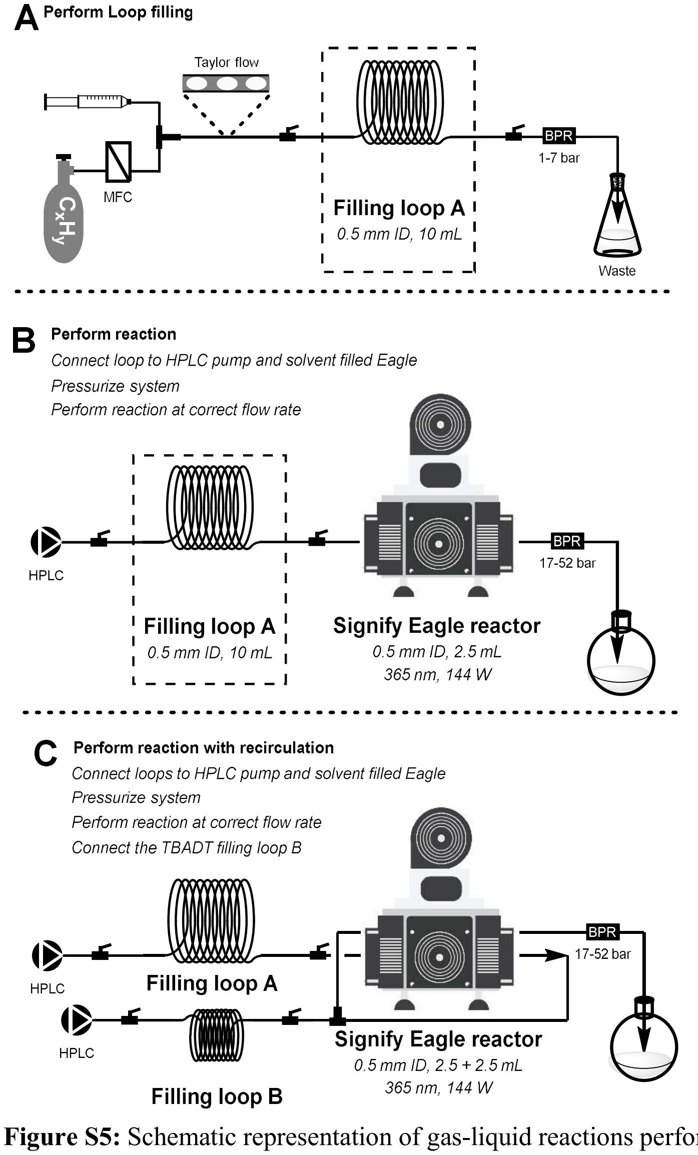

Figure S5: Schematic representation of gas-liquid reactions performed with a loop filling method. (A) Loop filling of gas-liquid mixture, (B) Pressurizing the system and run the reaction using the correct flow rate. BPR: Back-Pressure Regulator. (C) System with recirculation.

The flow over the BPR only starts when the system has reached the design pressure of the BPR. The reaction is then performed through irradiation of the solution inside the reactor and the reaction mixture is collected at the outlet. In the case of the recirculation setup the TBADT loop filling (loop filling B) is connected to the outlet of the first coil through a T mixer which let the resulting mixture enter to the second coil wrapped into the first (Figure S5 C).

4. Reaction Optimization

4.1 Photoreactor Evaluation

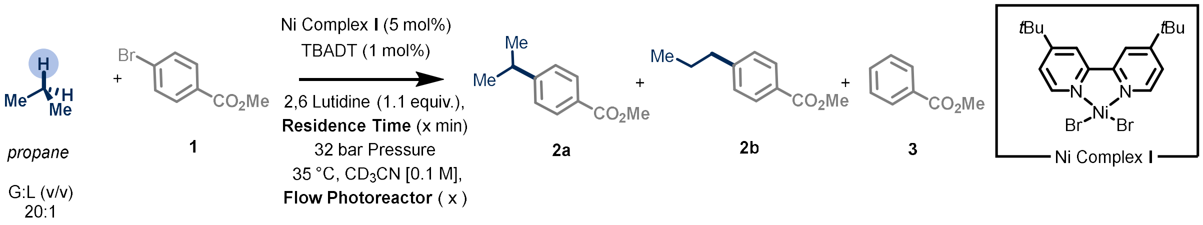

Table S1 . Evaluation of the photoreactor and residence time

	Residence Time	Yield of **1a**	Yield of **2a : 2b**	**Selectivity**	Yield of **2a + 2b**	Yield of **3**
150 W Vapourtec	15 min	95%	-	-	0%	
150 W Vapourtec	30 min	84%	7 : 4	1,8	11%	-
150 W Vapourtec	60 min	61%	13 : 8	1,75	21%	-
150 W Vapourtec	120 min	50%	15 : 8	1,9	23%	10
150 W Vapourtec	240 min	48%	15 : 7	2,0	22%	14
**144 W Eagle**	**15 min**	**40%**	**18 : 15**	**1,2**	**33%**	**21%**
144 W Eagle	30 min	41%	18 : 15	1,2	33%	23%

4.2 Catalysts loading Screening

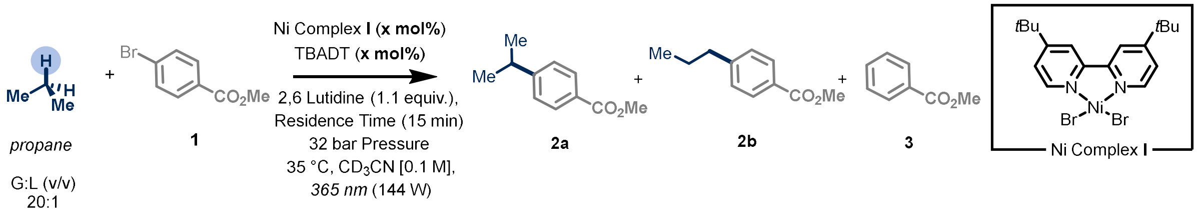

Table S2. Influence of the catalyst loading on the photocatalytic alkylation of 1 with propane.

Catalysts Loading	Yield of **1a**	Yield of **2a : 2b**	Yield of **2a + 2b**	Yield of **3**
TBADT (1 mol%), Ni I (2 mol%)	52	12 : 8	20	15
**TBADT (1 mol%), Ni I (5 mol%)**	**40**	**18 : 15**	**33**	**22**
TBADT (1 mol%), Ni I (10 mol%)	41	17 :13	30	23
TBADT (2.5 mol%), Ni I (5 mol%)	48	20 : 13	33	21
TBADT (5 mol%), Ni I (5 mol%)	clogging			

4.3 Ligand Screening

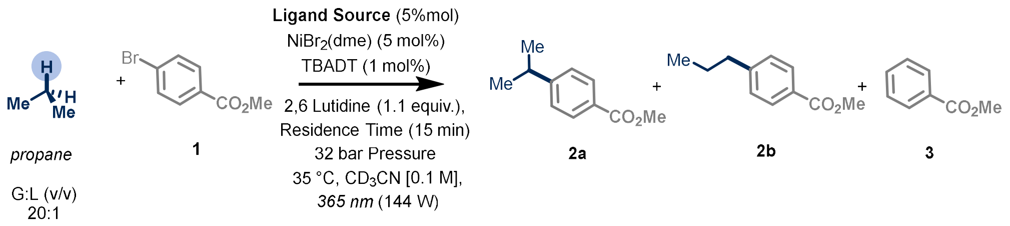

Table S3 Influence of the ligand on the photocatalytic alkylation of 1 with propane.

Ligand Source	Yield of **1**	Yield of **2a** : **2b**	Yield of **2a** + **2b**	Yield of **3**
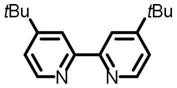	**40**	**18 : 15**	**33**	**22**
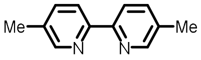	>80%	n.d.	<20%	n.d.
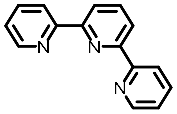	>80%	n.d.	<20%	n.d.
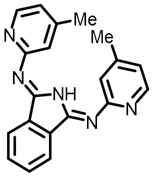	Not soluble			
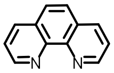	>80%	n.d.	<20%	n.d.
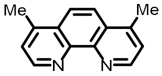	>80%	n.d.	<20%	n.d.
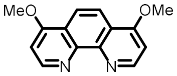	>80%	n.d.	<20%	n.d.
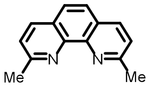	>80%	n.d.	<20%	n.d.

4.4 Gas to Liquid Ratio Screening

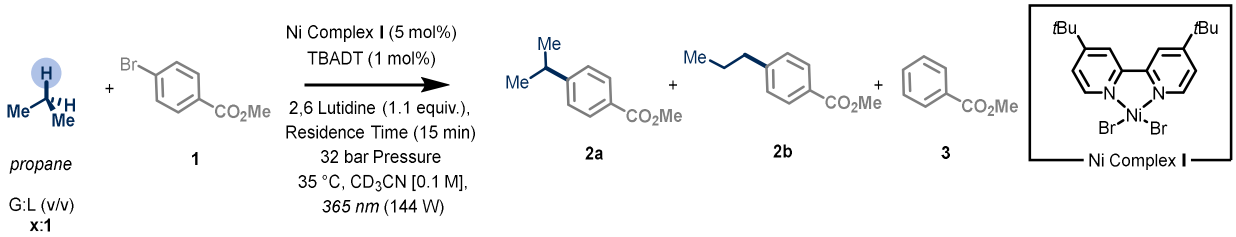

Table S4. Influence of the Gas-to-Liquid ratio on the photocatalytic alkylation of 1 with propane.

Gas : Liquid Ratio	Yield of **1a**	Yield of **2a : 2b**	Yield of **2a + 2b**	Yield of **3**
20 : 1	40	18 : 15	33	22
**40 : 1**	**46**	**29 : 11**	**40**	**10**
60 : 1	48	29 : 10	39	9
80 : 1	58	20 : 8	28	8

4.5 Additive Screening

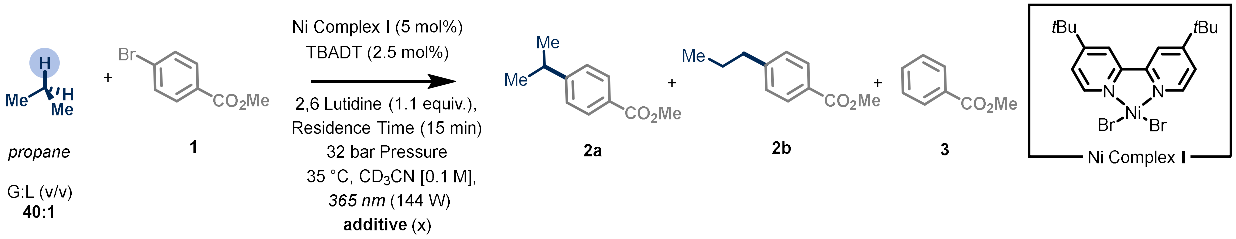

Table S5 Influence of the additives on the photocatalytic alkylation of 1 with propane.

Solvent	Additive	Yield of **1**	Yield of **2a : 2b**	Yield of **2a + 2b**	Yield of **3**
CD_3CN	no	46	29 : 11	40	10
CD_3CN	+2.5 mol% TBADT	38	34 : 12	46	6
CD_3CN	+2.5 mol% TBADT	clogging			
CD_3CN	+2.5 mol% TBADT, LiCl 1.5 equiv.	-	33 : 8	41	2
CD_3CN	+2.5 mol% TBADT, LiBr 1.5 equiv.	25	30 : 14	44	10
CD_3CN:tBuOH (3:1)	+2.5 mol% TBADT	20	29 : 19	48	10
**CD_3CN:tBuOH (3:1)**	**+2.5 mol% TBADT, LiBr 1.5 equiv.**	**16**	**36 : 18**	**54**	**12**

4.6 Recirculation

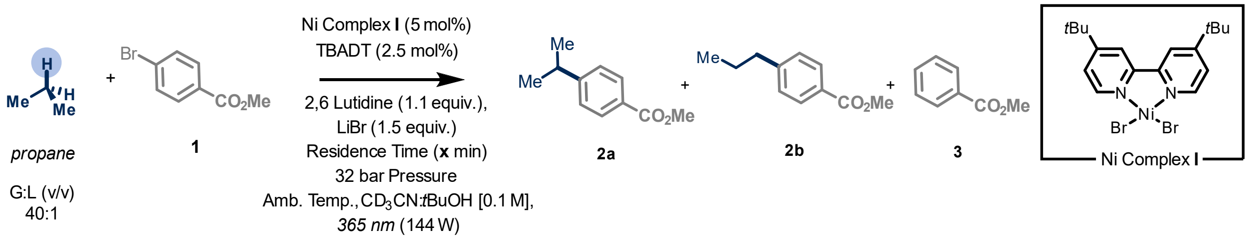

Table S6. Recirculation of the reaction mixture to extend the reaction time.

Residence time	Yield of **1**	Yield of **2a** : **2b**	Yield of **2a** + **2b**	Yield of **3**
15 min	16	36:18	54	12
30 min	14	39:17	56	8
30 min + 20 min	15	37:18	55	6
**30 min + 20 min***	-	**41:19**	**60**	**7**

Implementing a recirculation system with the introduction of a fresh solution of TBADT before the recirculation step allow to reach full conversion (Figure S6).

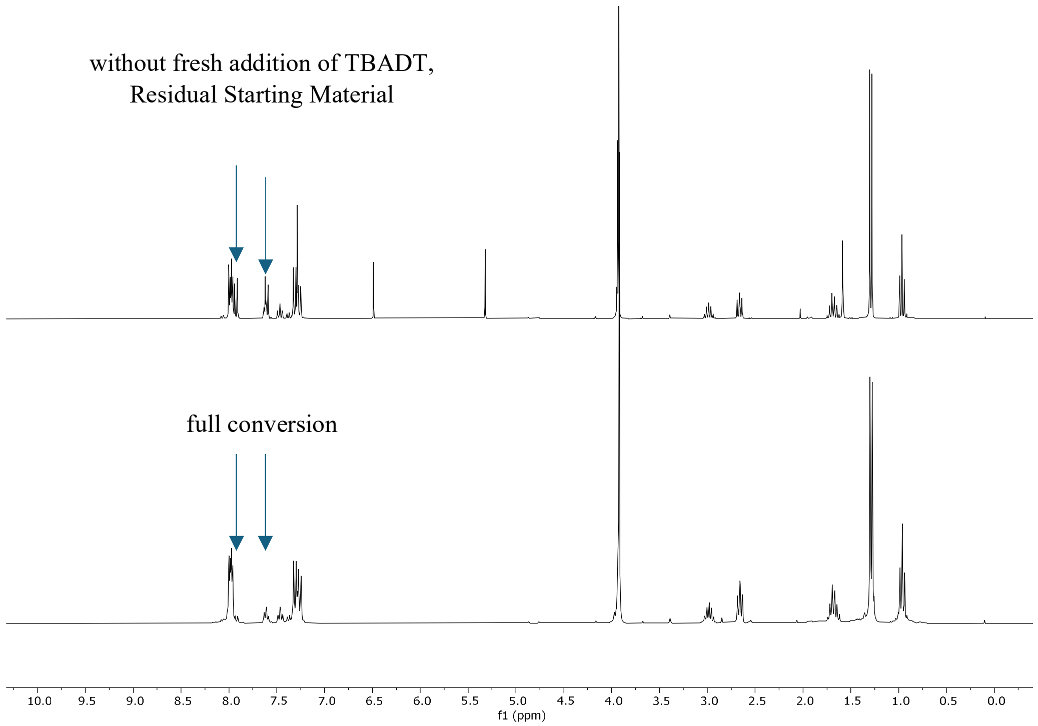

Figure S6: ^{1}H NMR of the reaction mixture without (up) and with (down) fresh addition of TBADT.

4.7 Optimization for Ethane

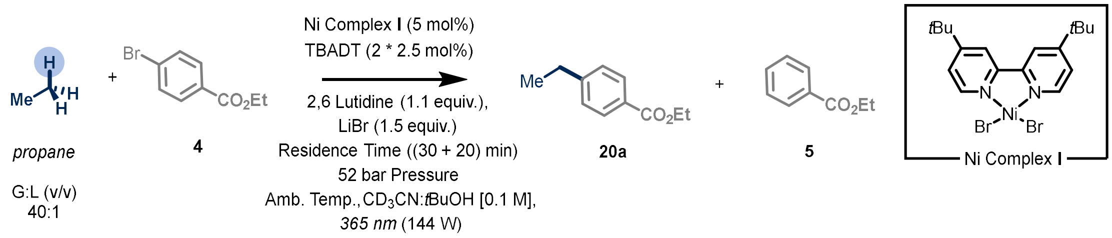

Table S7. Reaction optimization for ethane.

Deviation from standard Conditions	Yield of **4**	Yield of **20a**	Yield of **5**
None	30	17	16
30 min + 30 min	14	19	15
$FeCl_3$ (10 mol%) insted of TBADT	24	15	17
**Ni (5 mol%) in reciculation loop**	**25**	**29**	**23**

5. General Procedures (GP)

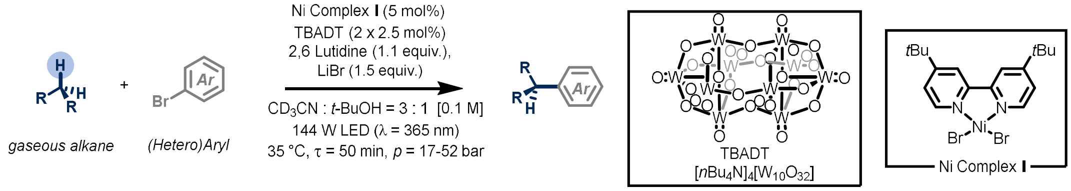

5.1 (GP1) *n*-Butane

To a nitrogen-purged, screw-capped vial, fitted with a rubber septum and charged with nickel complex **I** (7.3 mg, 15 µmol, 5 mol%), TBADT (25 mg, 7.5 µmol, 2.5 mol%), LiBr (39 mg, 0.45 mmol, 1.5 equiv.), 2,6-dimethylpyridine (38 µL, 0.33 mmol, 1.1 equiv.), the aryl bromide (0.3 mmol, 1 equiv.) solubilized in 2.25 mL CD_3CN + 0.75 mL *t*-BuOH (3:1 volume ratio 0.1 M).
The stock solution is charged in a gastight syringe, positioned in a syringe pump and combined with a stream of butane gas (120 mL, 5.14 mmol, 17 equiv.) through a T-mixer into filling loop A, with a liquid flow rate of 0.1 mL·min^{-1} and a butane gas flow rate of 4 mL·min^{-1} (40 : 1 = gas : liquid).
No back pressure regulator was used for the loop filling step.
The TBADT stock solution used for the recirculation is prepared solubilizing (25 mg, 2.5 mol%) in 1.5 mL of CD_3CN. This solution is added into a dedicated TBADT loop (filling loop B) (Figure S5).
Next, the filling loop and the TBADT loop are connected to the reactor, to the start and to the of the first coil respectively. The system is pressurized to 34 bar using an HPLC pump and the reaction mixture is pumped over the Signify Eagle reactor (365 nm, 144 W output power, FEP capillary: 0.5 mm ID, 2.5 mL) at a flow rate of 0.083 mL/min, resulting in a residence time of 30 min.
After around 29 min the TBADT stock solution is pumped over through a T mixer connected at the end of the first coil at a flow rate of 0.041 mL·min^{-1} (the total volume of TBADT stock solution used is 1.5 mL, 7.5 µmol, 2.5 mol%). The sum of the two flow rates (recirculation) is 0,125 mL/min and in the second coil (FEP capillary: 0.5 mm ID, 2.5 mL) this results in a residence time of 20 min.
The obtained reaction mixture is collected into vial, submitted to GC-FID analysis, and evaporated in vacuo. The residue is purified by column chromatography.

5.2 (GP2) Propane

To a nitrogen-purged, screw-capped vial, fitted with a rubber septum and charged with nickel complex **I** (7.3 mg, 15 µmol, 5 mol%), TBADT (25 mg, 7.5 µmol, 2.5 mol%), LiBr (39 mg, 0.45 mmol, 1.5 equiv.), 2,6-dimethylpyridine (38 µL, 0.33 mmol, 1.1 equiv.), the aryl bromide (0.3 mmol, 1 equiv.) solubilized in 2.25 mL CD_3CN + 0.75 mL *t*-BuOH (3:1 volume ratio 0.1 M).
The stock solution is charged in a gastight syringe, positioned in a syringe pump and combined with a stream of propane gas (120 mL, 4.89 mmol, 16 equiv.) through a T-mixer into filling loop A, with a liquid flow rate of 0.1 mL·min^{-1} and a butane gas flow rate of 4 mL·min^{-1} (40 : 1 = gas : liquid).
A BPR of 2.8 bar was used during the loop filling step.
The TBADT stock solution used for the recirculation is prepared solubilizing TBADT (25 mg, 2.5 mol%) in 1.5 mL of CD_3CN. This solution is added into a dedicated TBADT loop (filling loop B) (Figure S5).
Next, the filling loop and the TBADT loop are connected to the reactor, to the start and to the of the first coil respectively. The system is pressurized to 34 bar using an HPLC pump and the reaction mixture is pumped over the Signify Eagle reactor (365 nm, 144 W output power, FEP capillary: 0.5 mm ID, 2.5 mL) at a flow rate of 0.083 mL/min, resulting in a residence time of 30 min.
After around 29 min the TBADT stock solution is pumped over through a T mixer connected at the end of the first coil at a flow rate of 0.041 mL·min^{-1} (the total volume of TBADT stock solution used is 1.5 mL, 7.5 µmol, 2.5 mol%). The sum of the two flow rates (recirculation) is 0,125 mL/min and in the second coil (FEP capillary: 0.5 mm ID, 2.5 mL) this results in a residence time of 20 min.
The obtained reaction mixture is collected into vial, submitted to GC-FID analysis, and evaporated in vacuo. The residue is purified by column chromatography.

5.3 (GP3) Ethane

To a nitrogen-purged, screw-capped vial, fitted with a rubber septum and charged with nickel complex **I** (7.3 mg, 15 µmol, 5 mol%), TBADT (25 mg, 7.5 µmol, 2.5 mol%), LiBr (39 mg, 0.45 mmol, 1.5 equiv.), 2,6-dimethylpyridine (38 µL, 0.33 mmol, 1.1 equiv.), the aryl bromide (0.3 mmol, 1 equiv.) solubilized in 2.25 mL CD_3CN + 0.75 mL *t*-BuOH (3:1 volume ratio 0.1 M).
The stock solution is charged in a gastight syringe, positioned in a syringe pump and combined with a stream of ethane gas (120 mL, 5.10 mmol, 17 equiv.) through a T-mixer into filling loop A, with a liquid flow rate of 0.1 $mL{\cdot}min^{-1}$ and a butane gas flow rate of 4 $mL{\cdot}min^{-1}$ (40 : 1 = gas : liquid). A BPR of 2.8 was used during the loop filling step.
The TBADT stock solution used for the recirculation is prepared solubilizing TBADT (25 mg, 2.5 mol%) and nickel complex **I** (7.3 mg, 15 µmol, 5 mol%) in 1.5 mL of CD_3CN. This solution is added into a dedicated TBADT loop (filling loop B) (Figure S5).
Next, the filling loop and the TBADT loop are connected to the reactor, to the start and to the of the first coil respectively. The system is pressurized to 52 bar using an HPLC pump and the reaction mixture is pumped over the Signify Eagle reactor (365 nm, 144 W output power, FEP capillary: 0.5 mm ID, 2.5 mL) at a flow rate of 0.083 mL/min, resulting in a residence time of 30 min.
After around 29 min the TBADT stock solution is pumped over through a T mixer connected at the end of the first coil at a flow rate of 0.041 $mL{\cdot}min^{-1}$ (the total volume of TBADT stock solution used is 1.5 mL, 7.5 µmol, 2.5 mol%). The sum of the two flow rates (recirculation) is 0,125 mL/min and in the second coil (FEP capillary: 0.5 mm ID, 2.5 mL) this results in a residence time of 20 min.
The obtained reaction mixture is collected into vial, submitted to GC-FID analysis, and evaporated in vacuo. The residue is purified by column chromatography.

5.4 (GP4) Methane

To a nitrogen-purged, screw-capped vial, fitted with a rubber septum and charged with nickel complex **I** (7.3 mg, 15 µmol, 5 mol%), TBADT (25 mg, 7.5 µmol, 2.5 mol%), LiBr (39 mg, 0.45 mmol, 1.5 equiv.), 2,6-dimethylpyridine (38 µL, 0.33 mmol, 1.1 equiv.), the aryl bromide (0.3 mmol, 1 equiv.) solubilized in 2.25 mL CD_3CN + 0.75 mL *t*-BuOH (3:1 volume ratio 0.1 M).
The stock solution is charged in a gastight syringe, positioned in a syringe pump and combined with a stream of propane gas (120 mL, 5.36 mmol, 18 equiv.) through a T-mixer into filling loop A, with a liquid flow rate of 0.1 $mL{\cdot}min^{-1}$ and a butane gas flow rate of 4 $mL{\cdot}min^{-1}$ (40 : 1 = gas : liquid A BPR of 2.8 was used during the loop filling step.
The TBADT stock solution used for the recirculation is prepared solubilizing TBADT (35 mg, 3.5 mol%) and nickel complex **I** (10.3 mg, 15 µmol, 5 mol%) in 1.5 mL of CD_3CN. This solution is added into a dedicated TBADT loop (filling loop B) (Figure S5).
Next, the filling loop and the TBADT loop are connected to the reactor, to the start and to the of the first coil respectively. The system is pressurized to 52 bar using an HPLC pump and the reaction mixture is pumped over the Signify Eagle reactor (365 nm, 144 W output power, FEP capillary: 0.5 mm ID, 2.5 mL) at a flow rate of 0.083 mL/min, resulting in a residence time of 30 min.
After around 29 min the TBADT stock solution is pumped over through a T mixer connected at the end of the first coil at a flow rate of 0.041 $mL{\cdot}min^{-1}$ (the total volume of TBADT stock solution used is 1.5 mL, 7.5 µmol, 2.5 mol%). The sum of the two flow rates (recirculation) is 0,125 mL/min and in the second coil (FEP capillary: 0.5 mm ID, 2.5 mL) this results in a residence time of 20 min.
The obtained reaction mixture is collected into vial, submitted to GC-FID analysis, and evaporated in vacuo. The yield was determined by calibrated GC-FID using dodecane as internal standard.

5.5 Scale-up

To a nitrogen-purged, screw-capped vial, fitted with a rubber septum and charged with nickel complex **I** (24 mg, 50 µmol, 5 mol%), TBADT (83 mg, 25 µmol, 2.5 mol%), LiBr (130 mg, 1.5 mmol, 1.5 equiv.), 2,6-dimethylpyridine (127 µL, 1.1 mmol, 1.1 equiv.), methyl 4-bromobenzoate (1 mmol, 215 mg, 1 equiv.) and solubilized in 7.5 mL CD_3CN + 2.5 mL *t*-BuOH (3:1 volume ratio 0.1 M).
The stock solution is charged in a gastight syringe, positioned in a syringe pump and combined with a stream of propane gas (400 mL, 16.32 mmol, 16 equiv.) through a T-mixer into a filling loop, with a liquid flow rate of 0.1 $mL{\cdot}min^{-1}$ and a propane gas flow rate of 4 $mL{\cdot}min^{-1}$ (40 : 1 = gas : liquid).

The system is pressurized to 34 bar using an HPLC pump and the reaction mixture is pumped over the Signify Eagle reactor (365 nm, 144 W output power, FEP capillary: 0.8 mm ID, 11 mL) at a flow rate of 0.314 mL/min, resulting in a residence time of 35 min.

The obtained reaction mixture is collected into vial, submitted to GC-FID analysis and evaporated in vacuo. The residue is purified by column chromatography (from Pentane to Pentane:Ethyl Acetate 30:1) to afford the product as a colorless oil (98 mg, 55% yield).

6. Characterization data of synthesized compound

The regioisomeric ratio was assessed by GC-FID analysis of the reaction crude. In the case of propane and butane, due to overlapping signals, the reported peaks are for both linear and branched regioisomers.

n-Butane:

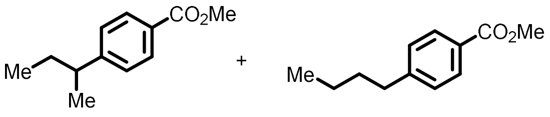

3a **3b**

***methyl 4-(sec-butyl)benzoate* (3a, major) *and methyl 4-butylbenzoate* (3b, minor).** Prepared according to GP1 from methyl 4-bromobenzoate (64.5 mg, 0.3 mmol, 1.0 equiv.) and butane. The regioisomeric ratio (r.r) was determined by GC-FID of the crude reaction mixture to be 4:1. Purified via flash column chromatography on silica gel (from Pentane to Pentane:Ethyl acetate 50:1) to afford the product as a colorless oil (42 mg, 72% yield).

^{1}H NMR (400 MHz, $CDCl_3$) δ 8.00 – 7.87 (*regioisomeric multiplet*, 2H **3a**+**3b**), 7.27 – 7.21 (*regioisomeric multiplet*, 2H **3a**+**3b**), 3.91 (s, 0.2H **3b**), 3.90 (s, 2.8H **3a**), 2.69 – 2.63 (m, 1H), 1.60 (q, *J* = 7.5 Hz, 3H), 1.40 – 1.32 (m, 1H), 0.99 – 0.75 (m, 4H).
^{13}C NMR (101 MHz, $CDCl_3$) δ 167.4, 153.4, 148.6, 129.8, 129.8, 128.6, 127.9, 127.2, 52.1, 41.9, 35.8, 33.4, 31.1, 29.8, 22.4, 21.7, 14.0, 12.3.
HRMS (ESI+) (m/z): $[M+H]^+$ calcd. for $C_{12}H_{16}O_2$, 192.1150; found: 192.1153.

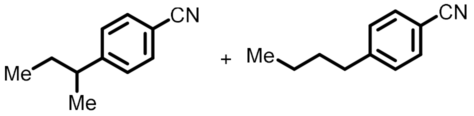

4a **4b**

***4-(sec-butyl)benzonitrile* (4a, major) *and 4-butylbenzonitrile* (4b, minor).** Prepared according to GP1 from 4-bromobenzonitrile (55 mg, 0.3 mmol, 1.0 equiv.) and butane. The regioisomeric ratio (r.r) was determined by GC-FID of the crude reaction mixture to be 4:1. Purified via flash column chromatography on silica gel (from Pentane to Pentane:Ethyl acetate 50:1) to afford the product as a colorless oil (33 mg, 69% yield).

^{1}H NMR (400 MHz, $CDCl_3$) δ 7.57 – 7.52 (*regioisomeric multiplet*, 2H **4a**+**4b**), 7.27 – 7.22 (*regioisomeric multiplet*, 2H **4a**+**4b**), 2.99 – 2.27 (m, 1H), 1.95 – 1.40 (m, 2H), 1.40 – 1.23 (m, 0.5H **4b**), 1.19 (d, *J* = 6.9 Hz, 2.2H **4a**), 0.88 (t, *J* = 7.3 Hz, 0.8H **4b**), 0.77 (t, *J* = 7.4 Hz, 2H **4a**).
^{13}C NMR (101 MHz, $CDCl_3$) δ 153.4 (**4a**), 148.7 (**4b**), 132.2, 129.8, 129.3, 128.0, 119.3, 109.8, 42.1 (**4a**), 35.9 (**4b**), 33.2 (**4b**), 30.9 (**4a**), 22.4 (**4b**), 21.5 (**4a**), 14.0 (**4b**), 12.2 (**4a**).
HRMS (ESI+) (m/z): $[M+H]^+$ calcd. for $C_{11}H_{13}N$, 159.1048; found: 159.1043.

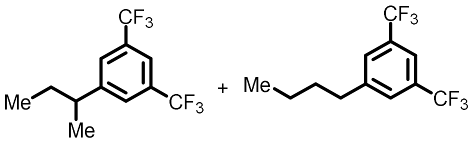

5a **5b**

***1-(sec-butyl)-3,5-bis(trifluoromethyl)benzene* (5a, major) *and 1-butyl-3,5-bis(trifluoromethyl)benzene* (4b, minor).** Prepared according to GP1 from 1-bromo-3,5-bis(trifluoromethyl)benzene (88 mg, 0.3 mmol, 1.0 equiv.) and butane. The regioisomeric ratio (r.r) was determined by GC-FID of the crude reaction mixture to be 4:1. Purified via flash column chromatography on silica gel (from Pentane to Pentane:Ethyl acetate 50:1) to afford the product as a colorless oil (53 mg, 65% yield).

^{1}**H NMR** (400 MHz, $CDCl_3$) δ 7.71 (*regioisomeric singlet* , 1H), 7.62 (*regioisomeric singlet*, 2H), 2.78-2.72 (m, *J* = 10.7, 7.5 Hz, 1H), 1.72 – 1.59 (m, 2H **5a**), 1.38 (m, *J* = 7.4 Hz, 0.8H **5b**), 1.29 (d, *J* = 7.0 Hz, 2.2H **5a**), 0.96 (t, *J* = 7.3 Hz, 1H **5b**), 0.90 – 0.85 (m, 2.6H **5a**).
^{13}**C NMR** (101 MHz, $CDCl_3$) δ 150.2 (**5a**), 145.3 (**5b**), 131.0 (q, *J* = 32.9 Hz (**5a+5b**), 128.7 (**5b**), 127.4 (**5a**), 125.0(**5a**), 125.0 (**5b**), 122.3 (**5a**), 122.2 (**5b**), 120.0 (q, *J* = 4.1 Hz **5a**), 119.9 (q, *J* = 4.1 Hz **5b**), 35.5, 33.3, 31.1, 29.9, 22.4 (**5b**), 21.6 (**5a**), 14.0 (**5b**), 12.2 (**5a**).
^{19}**F NMR** (282 MHz, $CDCl_3$) δ -62.8 (**5a**), -62.9 (**5b**).
HRMS (ESI+) (m/z): $[M+H]^+$ calcd. for $C_{12}H_{12}F_6$, 247.0843; found: 247.20850.

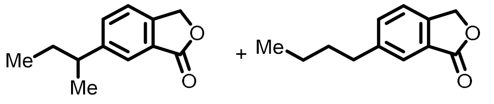

6a **6b**

***6-(sec-butyl)isobenzofuran-1(3H)-one* (6a, major) *and 6-butylisobenzofuran-1(3H)-one* (6b, minor).** Prepared according to GP1 from methyl 6-bromoisobenzofuran-1(3H)-one (64 mg, 0.3 mmol, 1.0 equiv.) and butane. The regioisomeric ratio (r.r) was determined by GC-FID of the crude reaction mixture to be 3:1. Purified via flash column chromatography on silica gel (from Pentane to Pentane:Ethyl Acetate 50:1) to afford the product as a colorless oil (35 mg, 60% yield).

^{1}**H NMR** (400 MHz, $CDCl_3$) δ 7.84 (*regioisomeric doublet*, *J* = 7.8 Hz, 1H **6a**+**6b**), 7.35 (*regioisomeric doublet*, *J* = 8.0 Hz, 1H **6a**+**6b**), 7.28 (*regioisomeric singlet*, 1H **6a**+**6b**), 5.29 (*regioisomeric singlet*, 2H **6a**+**6b**), 2.79-2.69 (m, 1H **6a**), 1.66-1.62 (m, 2H **6a**), 1.36 (m, 0.3H **6b**), 1.28 (d, *J* = 6.9 Hz, 2.7H **6a**), 0.96 – 0.91 (m, 0.5H **6b**), 0.83 (t, *J* = 7.4 Hz, 2.5H **6a**).
^{13}**C NMR** (101 MHz, $CDCl_3$) δ 171.3 (**6a**), 155.2 (**6a**), 147.2 (**6a**), 128.5 (**6a**), 125.8 (**6a**), 123.8 (**6b**), 120.5 (**6a**), 69.9 (**6a**), 42.3 (**6a**), 31.2 (**6a**), 21.9 (**6a**), 12.3 (**6a**).
HRMS (ESI+) (m/z): $[M+H]^+$ calcd. for $C_{12}H_{14}O_2$, 286.0944; found: 286.0949.

Low concentration avoids the unambiguous ^{13}C NMR characterization of the minor regioisomer.

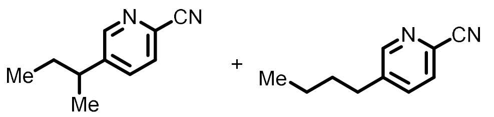

7a **7b**

***5-(sec-butyl)picolinonitrile* (7a, major) *and 5-butylpicolinonitrile* (7b, minor).** Prepared according to GP1 from methyl 5-bromopicolinonitrile (55 mg, 0.3 mmol, 1.0 equiv.) and butane. The regioisomeric ratio (r.r) was determined by GC-FID of the crude reaction mixture to be 3:1. Purified via flash column chromatography on silica gel (from Pentane to Pentane:Ethyl Acetate 10:1) to afford the product as a colorless oil (33 mg, 68% yield).

^{1}**H NMR** (400 MHz, $CDCl_3$) δ 8.56 – 8.52 (*regioisomeric multiplet*, 1H **7a**+**7b**), 7.64 – 7.59 (*regioisomeric multiplet*, 2H **7a**+**7b**), 2.93 – 2.56 (m, 1.3H), 1.86 – 1.47 (m, 2,34H), 1.40-1.33 (m, 0.6H), 1.28 (d, *J* = 7.0 Hz, 2.4H), 0.94 (t, *J* = 7.3 Hz, 0.7H), 0.84 (t, *J* = 7.4 Hz, 2.3H).
^{13}**C NMR** (101 MHz, $CDCl_3$) δ 151.6 (**7b**), 150.9 (**7a**), 147.1, 142.6, 136.6 (**7b**), 135.2 (**7a**), 131.6 (**7a**), 131.4 (**7b**), 128.4 (**7a**), 128.3 (**7b**), 117.6, 39.5, 33.0 (**7a**), 32.9, 30.8 (**7a**), 22.3 (**7b**), 21.2 (**7a**), 13.9 (**7b**), 12.1 (**7a**).
HRMS (ESI+) (m/z): $[M+H]^+$ calcd. for $C_{10}H_{12}N_2$, 161.1000; found: 161.1004.

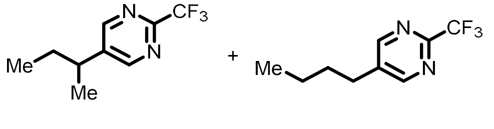

8a **8b**

***5-(sec-butyl)-2-(trifluoromethyl)pyrimidine* (8a, major) *and 5-butyl-2-(trifluoromethyl)pyrimidine* (8b, minor).** Prepared according to GP1 from methyl 5-bromopicolinonitrile (68 mg, 0.3 mmol, 1.0 equiv.) and butane. The regioisomeric ratio (r.r) was determined by GC-FID of the crude reaction mixture to be 3:1. Purified via flash column

chromatography on silica gel (from Pentane to Pentane:Ethyl 10:1) to afford the product as a colorless oil (26 mg, 42% yield).

^{1}H NMR (400 MHz, $CDCl_3$) δ 8.74 – 8.63 (*regioisomeric multiplet*, 1H **8a**+**8b**), 2.80-2.65 (m, 1H), 1.83 – 1.61 (m, 2H) (**8b**), 1.34 (d, *J* = 7.0, 3H) (**8a**), 1.04 – 0.94 (m, 1H) (**8b**), 0.89 (t, *J* = 7.4, 2H) (**8a**).
^{13}C NMR (101 MHz, $CDCl_3$) δ 156.9, 142.5, 119.85 (q, *J* = 274.7 Hz), 37.3, 30.6, 20.9, 12.0
^{19}F NMR (282 MHz, $CDCl_3$) δ -70.14 (**8a+8b**).
HRMS (ESI+) (m/z): $[M+H]^+$ calcd. for $C_9H_{11}F_3N_2$, 204.0874; found: 204.0871.

Low concentration avoids the unambiguous ^{13}C NMR characterization of the minor regioisomer.

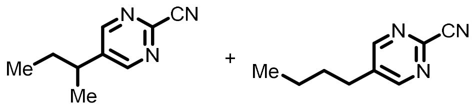

9a **9b**

***5-(sec-butyl)pyrimidine-2-carbonitrile* (9a, major) *and 5-butylpyrimidine-2-carbonitrile* (9b, minor).** Prepared according to GP1 from 5-bromopyrimidine-2-carbonitrile (55 mg, 0.3 mmol, 1.0 equiv.) and butane. The regioisomeric ratio (r.r) was determined by GC-FID of the crude reaction mixture to be 5:1. Purified via flash column chromatography on silica gel (from Pentane to Pentane:Ethyl Acetate10:1) to afford the product as a colorless oil (33 mg, 67% yield).

^{1}H NMR (400 MHz, $CDCl_3$) δ 8.67 – 8.65 (*regioisomeric multiplet*, 1H **9a**+**9b**), 2.74 (m, 1H), 1.69 (m, 2H), 1.33 (d, *J* = 7.0 Hz, 3H), 0.88 (t, *J* = 7.4 Hz, 3H).
^{13}C NMR (101 MHz, $CDCl_3$) δ 157.0 (**9a**), 143.3 (**9a**), 143.2 (**9a**), 115.9 (**9a**), 37.5 (**9a**), 30.5 (**9a**), 20.8 (**9a**), 12.0 (**9a**).
HRMS (ESI+) (m/z): $[M+H]^+$ calcd. for $C_9H_{11}N_3$, 161.0953; found: 161.0958.

Low concentration avoids the unambiguous ^{13}C NMR characterization of the minor regioisomer.

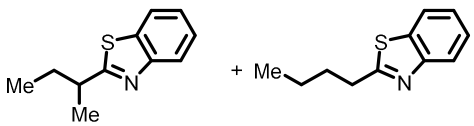

10a **10b**

***2-(sec-butyl)benzo[d]thiazole* (10a, major) *and 2-butylbenzo[d]thiazole* (10b, minor).** Prepared according to GP1 from 2-bromobenzo[d]thiazole (64 mg, 0.3 mmol, 1.0 equiv.) and butane. The regioisomeric ratio (r.r) was determined by GC-FID of the crude reaction mixture and ^{1}H NMR of the crude mixture to be 4:1. Purified via flash column chromatography on silica gel (from Pentane to Pentane:Ethyl Acetate 10:1) to afford the product as a colorless oil (26 mg, 47% yield).

Low concentration avoids the unambiguous 13C NMR characterization of the minor regioisomer.

^{1}H NMR (400 MHz, $CDCl_3$) δ 7.98 (*regioisomeric doublet*, *J* = 8.1, 1H **10a**+**10b**), 7.85 (*regioisomeric doublet*, *J* = 8.0, 1H **10a**+**10b**), 7.47 – 7.41 (*regioisomeric multiplet*, 1H **10a**+**10b**), 7.37 – 7.30 (*regioisomeric multiplet*, 1H **10a**+**10b**), 3.21 (h, *J* = 6.9 Hz, 0.9H **10a**), 3.12 (t, 0.1H **10b**), 1.97 – 1.86 (m, 1H **10a**), 1.77 (m, 1H **10a**), 1.45 (d, *J* = 7.0 Hz, 3H **10a**), 1.00-1.96 (t, 3H **10a**).
^{13}C NMR (101 MHz, $CDCl_3$) δ 178.1, 153.2, 134.8, 125.9, 124.7, 122.7, 121.7, 41.2, 30.8, 20.9, 12.0.
HRMS (ESI+) (m/z): $[M+H]^+$ calcd. for $C_{11}H_{13}NS$, 191.0769; found: 191.0773.

Low concentration avoids the unambiguous ^{13}C NMR characterization of the minor regioisomer.

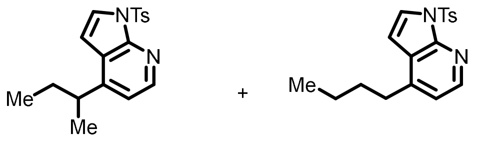

11a **11b**

***4-isopropyl-1-tosyl-1H-pyrrolo[2,3-b]pyridine* (11a, major) *and 4-propyl-1-tosyl-1H-pyrrolo[2,3-b]pyridine* (11b, minor).** Prepared according to GP1 from 4-bromo-1-tosyl-1H-pyrrolo[2,3-b]pyridine (105 mg, 0.3 mmol, 1.0 equiv.) and propane. The regioisomeric ratio (r.r) was determined by GC-FID of the crude reaction mixture and ^{1}H NMR of the crude mixture to be 4:1. Purified via flash column chromatography on silica gel (from Pentane to Pentane:Ethyl Acetate 40:1 to 20:1) to afford the product as a colorless oil (47 mg, 48% yield).

^{1}H NMR (400 MHz, $CDCl_3$) ^{1}H NMR (400 MHz, $CDCl_3$) δ 8.34 (d, *J* = 5.1 Hz, 0.7H **11a**), 8.31 (d, *J* = 5.0 Hz, 0.3H **11b**), 8.11 – 8.07 (m, 2H, *regioisomeric multiplet* **11a**+**11b**), 7.69 – 7.66 (d, *J* = 4.1 Hz, 1H, *regioisomeric multiplet* **11a**+**11b**), 7.28-7.24 (*regioisomeric multiplet*, 2H **11a**+**11b**), 7.00-6.95 (*regioisomeric multiplet*, 1H **11a**+**11b**), 6.64 (d, *J* = 4.1 Hz, 0.7H **11a**), 6.61 (d, *J* = 4.1 Hz, 0.3H **11b**), 2.95 (q, *J* = 7.0 Hz, 0.7H **11a**), 2.81 – 2.77 (m, 0.5H **11b**), 2.36 (*regioisomeric singlet*, 3H **11a**+**11b**), 1.78 – 1.58 (m, 2H), 1.39-1.30 (m, 0.7H), 1.28 (d, *J* = 7.0 Hz, 2.3H), 0.92 (t, *J* = 7.4 Hz, 0.7H), 0.81 (t, *J* = 7.4 Hz, 2.3H).
^{13}C NMR (101 MHz, $CDCl_3$) δ 150.4, 147.4, 145.6, 145.3, 145.1, 135.7, 135.7, 129.7, 128.2, 128.2, 125.6, 125.5, 122.1, 118.7, 116.2, 103.8, 103.8, 38.6, 32.4, 32.1, 30.1, 22.6, 21.8, 20.6, 15.4, 14.0, 13.0.
HRMS (ESI+) (m/z): $[M+H]^+$ calcd. for $C_{18}H_{20}N_2O_2S$, 328.1245; found: 328.1241.

Propane:

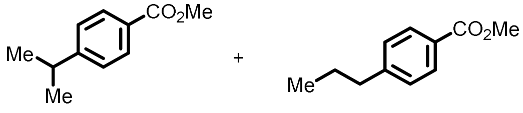

2a **2b**

***methyl 4-isopropylbenzoate* (2a, major) *and methyl 4-propylbenzoate* (2b, minor).** Prepared according to GP2 from methyl 4-bromobenzoate (64 mg, 0.3 mmol, 1.0 equiv.) and propane. The regioisomeric ratio (r.r) was determined by GC-FID of the crude reaction mixture and ^{1}H NMR of the crude mixture to be 2:1. Purified via flash column chromatography on silica gel (from Pentane to Pentane:Ethyl Acetate 20:1) to afford the product as a colorless oil (30 mg, 56% yield).

^{1}**H NMR** (400 MHz, $CDCl_3$) δ 7.98 – 7.92 (*regioisomeric multiplet*, 1H **2a**+**2b**), 7.29 (*J* = 8.2 Hz, 1H **2a**), 7.24 (*J* = 8.2 Hz, 1H **2b**), 3.90 (*regioisomeric singlet*, 3H **2a**+**2b**), 2.96 (hept, *J* = 6.9 Hz, 0.5H **2a**), 2.67 – 2.60 (m, 1H **2b** *benzylic methylene*), 1.66 (h, *J* = 7.3 Hz, 1H **2b**), 1.27 (d, *J* = 7.0 Hz, 3H **2a**), 0.94 (t, *J* = 7.4 Hz, 1.5H **2b**).
^{13}**C NMR** (101 MHz, $CDCl_3$) δ 167.4 (**2a**), 167.3 (**2b**), 154.5, 148.4, 129.9, 129.7, 128.6, 127.9, 127.8, 126.6, 52.1, 38.2 (**2b**), 34.4 (**2a**), 24.4 (**2b**), 23.8 (**2a**), 13.9(**2b**).
HRMS (ESI+) (m/z): $[M+H]^+$ calcd. for $C_{11}H_{14}O_2$, 178.0994; found: 178.0998.

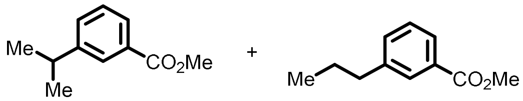

12a **12b**

***methyl 3-isopropylbenzoate* (12a, major) *and methyl 3-propylbenzoate* (12b, minor).** Prepared according to GP2 from methyl 3-bromobenzoate (64 mg, 0.3 mmol, 1.0 equiv.) and propane. The regioisomeric ratio (r.r) was determined by GC-FID of the crude reaction mixture and ^{1}H NMR of the crude mixture to be 1:1. Purified via preparative TLC (Pentane: Toluene 7:3) to afford the product as a colorless oil (27 mg, 50% yield).

^{1}**H NMR** (400 MHz, $CDCl_3$) δ 7.92 – 7.83 (*regioisomeric multiplet*, 2H 12**a**+12**b**), 7.44 – 7.31 (*regioisomeric multiplet*, 2H 12**a**+12**b**), 3.91 (*regioisomeric singlet*, 3H 12**a**+12**b**), 2.97 (hept, 0.5H **12a**), 2.68 – 2.59 (m, 1H **12b** *benzylic methylene*), 1.66 (dt, *J* = 14.8, 7.5 Hz, 1H **12b**), 1.27 (d, *J* = 6.9 Hz, 3H **12a**), 0.94 (t, 1.5H **12b**).
^{13}**C NMR** (101 MHz, $CDCl_3$) δ 167.5 (**12a**), 167.5 (**12b**), 149.3, 143.1, 133.3, 131.4, 130.3, 130.2, 129.7, 128.5, 128.4, 127.7, 127.3, 127.1, 52.2, 37.9 (**12b**), 34.2 (**12a**), 24.6 (**12b**), 24.0 (**12a**), 13.9 (**12b**).
HRMS (ESI+) (m/z): $[M+H]^+$ calcd. for $C_{11}H_{14}O_2$, 178.0994; found: 178.0997.

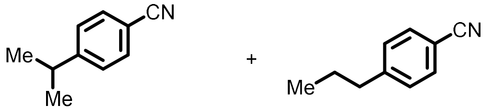

13a **13b**

***4-isopropylbenzonitrile* (13a, major) *and 4-propylbenzonitrile* (13b, minor).** Prepared according to GP2 from 4-bromobenzonitrile (55 mg, 0.3 mmol, 1.0 equiv.) and propane. The regioisomeric ratio (r.r) was determined by GC-FID of the crude reaction mixture and ^{1}H NMR of the crude mixture to be 2:1. Purified via flash column chromatography on silica gel (from Pentane:Ethyl Acetate 40:1 to 20:1) to afford the product as a colorless oil (21 mg, 48% yield).

^{1}**H NMR** (400 MHz, $CDCl_3$) δ 7.61 – 7.54 (*regioisomeric multiplet*, 2H **13a**+**13b**), 7.34 – 7.27 (*regioisomeric multiplet*, 2H **13a**+**13b**), 2.96 (hept, *J* = 6.9 Hz, 0.6H **13a**), 2.67 – 2.61 (m, 0.6H **13b** *benzylic methylene*), 1.65 (h, *J* = 7.4 Hz, 0.7H **13b**), 1.26 (d, *J* = 6.8 Hz, 4.3H **13a**), 0.94 (t, *J* = 7.3 Hz, 1H **13b**).
^{13}**C NMR** (101 MHz, $CDCl_3$) δ 154.5, 148.5, 132.4, 132.2, 129.4, 127.4, 119.3, 109.8, 109.7, 38.3 (**13b**), 34.5 (**13a**), 24.2 (**13b**), 23.7 (**13a**), 13.8(**13b**).
HRMS (ESI+) (m/z): $[M+H]^+$ calcd. for $C_{10}H_{11}N$, 145.0891; found: 145.0886.

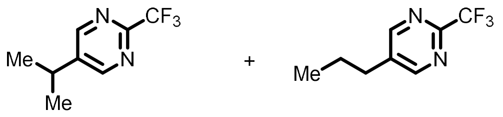

14a **14b**

***5-isopropyl-2-(trifluoromethyl)pyrimidine* (14a, major) *and 5-propyl-2-(trifluoromethyl)pyrimidine* (14b, minor).** Prepared according to GP2 from 5-bromo-2-(trifluoromethyl)pyrimidine (68 mg, 0.3 mmol, 1.0 equiv.) and propane. The regioisomeric ratio (r.r) was determined by GC-FID of the crude reaction mixture and ^{1}H NMR of the crude mixture. Purified via flash column chromatography on silica gel (from Pentane to Pentane:Ethyl Acetate 50:1) to afford the product as a colorless oil (30 mg, 53% yield).

^{1}H NMR (300 MHz, Chloroform-*d*) δ 8.76 (s, 1.5H **14a**), 8.72 (s, 0.5H **14b**), 3.06 (hept, *J* = 7.0 Hz, 0.7H **14a**), 2.69 (t, *J* = 7.7 Hz, 0.5H **14b** *benzylic methylene*), 1.79 – 1.64 (m, 0.5H **14b**), 1.36 (d, *J* = 7.0 Hz, 4.5H **14a**), 1.00 (t, *J* = 7.3 Hz, 1H **14b**).
^{13}C NMR (101 MHz, $CDCl_3$) δ 157.7 (**13b**), 156.4 (**13a**), 154.9 (q, *J* = 36.7 Hz **13a**), 143.4 (**13a**), 137.8 (**13b**), 119.8 (q, *J* = 275.0 Hz **13a**), 32.4 (**13b**), 30.1 (**13a**), 23.9 (**13b**), 23.2 (**13a**), 13.7 (**13b**).
^{19}F NMR (282 MHz, $CDCl_3$) δ -70.2.
HRMS (ESI+) (m/z): $[M+H]^+$ calcd. for $C_8H_9F_3N_2$, 190.0718; found: 190.0723.

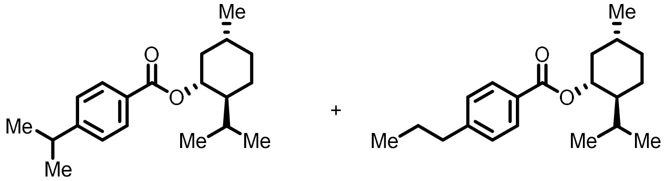

15a **15b**

***(1R,2S,5R)-2-isopropyl-5-methylcyclohexyl 4-isopropylbenzoate* (15a, major) *and (1R,2S,5R)-2-propyl-5-methylcyclohexyl 4-isopropylbenzoate* (15b, minor).** Prepared according to GP2 from (1R,2S,5R)-2-isopropyl-5-methylcyclohexyl 4-bromobenzoate (102 mg, 0.3 mmol, 1.0 equiv.) and propane. The regioisomeric ratio (r.r) was determined by ^{1}H NMR of the crude mixture to be 2:1. Purified via flash column chromatography on silica gel (from Pentane:Ethyl Acetate 30:1 to 10:1) to afford the product as a colorless oil (45 mg, 50% yield).

^{1}H NMR (400 MHz, $CDCl_3$) δ 7.99 – 7.92 (*regioisomeric multiplet*, 2H **15a+15b**), 7.29 (d, *J* = 8.2 Hz, 1.3H **15a**), 7.24 (d, *J* = 8.0 Hz, 0.6H **15b**), 4.92 (*regioisomeric* td, **15a+15b**, 1H *J* = 10.9, 4.4 Hz), 2.96 (hept, *J* = 6.9 Hz, 0.6H **15a**), 2.67 – 2.60 (m, 0.5H **15b** *benzylic methylene*), 2.16 – 2.07 (m, 1H), 2.02 – 1.88 (m, 1H), 1.76 – 1.68 (m, 2H), 1.57 (s, 4H), 1.27 (*regioisomeric doublet*, 5H **15a+15b**, *J* = 7.0 Hz), 1.17 – 1.04 (m, 2H), 0.95 – 0.89 (m, 6H), 0.79 (d, *J* = 7.0 Hz, 2.5H).
^{13}C NMR (101 MHz, $CDCl_3$) δ 166.3, 154.2, 129.9, 128.6, 126.5, 74.7, 47.5, 41.2, 34.5, 31.6, 26.6, 23.9, 23.8, 22.2, 20.9, 16.7.
HRMS (ESI+) (m/z): $[M+H]^+$ calcd. for $C_{20}H_{30}O_2$, 302.2246; found: 302.2251.

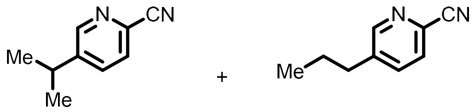

16a **16b**

***5-isopropylpicolinonitrile* (16a, major) *and 5-propylpicolinonitrile* (16b, minor).** Prepared according to GP2 from 5-bromopicolinonitrile (55 mg, 0.3 mmol, 1.0 equiv.) and propane. The regioisomeric ratio (r.r) was determined by GC-FID of the crude reaction mixture to be 4:1. Purified via flash column chromatography on silica gel (from Pentane:Ethyl Acetate 20:1 to 5:1) to afford the product as a colorless oil (21 mg, 48% yield).

^{1}H NMR (400 MHz, $CDCl_3$) δ 8.62 – 8.51 (*regioisomeric multiplet,* 1H **16a+16b**), 7.69 – 7.59 (*regioisomeric multiplet,* 2H **16a+16b**), 3.02 (hept, *J* = 7.0 Hz, 0.7H **16a**), 2.67 (dd, *J* = 8.5, 6.8 Hz, 0.4H **16b** *benzylic methylene*), 1.72 – 1.64 (m, 1H **16b**), 1.30 (d, *J* = 6.9 Hz, 4.5H **16a**), 0.96 (t, *J* = 7.3 Hz, 0.5H **16b**).

^{13}C NMR (101 MHz, CDCl$_3$) δ 151.9, 151.6, 150.4, 148.1, 136.7, 134.6, 131.5, 131.4, 128.4, 128.3, 117.6, 114.1, 35.2 (**16b**), 32.2 (**16a**), 24.0 (**16b**), 23.4 (**16a**), 13.7 (**16b**).
HRMS (ESI+) (m/z): $[M+H]^+$ calcd. for $C_9H_{10}N_2$, 146.0844; found: 146.0849.

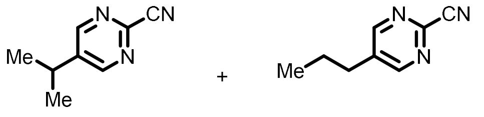

17a **17b**

***5-isopropylpyrimidine-2-carbonitrile* (17a, major) *and 5-propylpyrimidine-2-carbonitrile* (17b, minor).** Prepared according to GP2 from 5-bromopyrimidine-2-carbonitrile (55 mg, 0.3 mmol, 1.0 equiv.) and propane. The regioisomeric ratio (r.r) was determined by GC-FID of the crude reaction mixture to be 3:1. Purified via flash column chromatography on silica gel (from Pentane:Ethyl Acetate 40:1 to 20:1) to afford the product as a colorless oil (16 mg, 36% yield).

^{1}H NMR (300 MHz, CDCl$_3$) δ 8.70 (s, 1.6H **17a**), 8.66 (s, 0.4H **17b**), 3.04 (hept, *J* = 7.0 Hz, 0.8H **17a**), 2.68 (t, *J* = 7.7 Hz, 0.4H **17b** *benzylic methylene*), 1.77 – 1.65 (m, 0.5H **17b**), 1.35 (d, *J* = 7.0 Hz, 5H **17a**), 1.00 (t, 0.5H **17b**).
^{13}C NMR (101 MHz, CDCl$_3$) δ 157.9, 156.6, 144.1, 143.2, 138.6, 115.9, 115.0, 32.6 (**17b**), 30.3 (**17a**), 23.7 (**17b**), 23.1 (**17a**), 13.7(**17b**).
HRMS (ESI+) (m/z): $[M+H]^+$ calcd. for $C_8H_9N_3$, 147.0796; found: 147.0792.

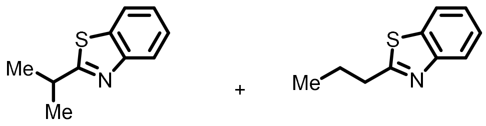

18a **18b**

***2-isopropylbenzo[d]thiazole* (18a, major) *and 2-propylbenzo[d]thiazole* (18b, minor).** Prepared according to GP2 from 2-bromobenzo[d]thiazole (64 mg, 0.3 mmol, 1.0 equiv.) and propane. The regioisomeric ratio (r.r) was determined by GC-FID of the crude reaction mixture to be 3:1. Purified via flash column chromatography on silica gel (from Pentane:Ethyl Acetate 40:1 to 10:1) to afford the product as a colorless oil (16 mg, 30% yield).

^{1}H NMR (400 MHz, CDCl$_3$) δ 8.00 – 7.95 (*regioisomeric multiplet*, 1H **18a**+**18b**), 7.88 – 7.81 (*regioisomeric multiplet*, 1H **18a**+**18b**), 7.49 – 7.42 (*regioisomeric multiplet*, 1H **18a**+**18b**), 7.38 – 7.30 (*regioisomeric multiplet*, 1H **18a**+**18b**), 3.43 (hept, *J* = 6.9 Hz, 0.8H **18a**), 3.10 (t, *J* = 7.6 Hz, 0.4H **18b** *benzylic methylene*), 1.92 (q, *J* = 7.5 Hz, 0.4H **18b**), 1.49 (d, *J* = 6.9 Hz, 4.7H **18a**), 1.06 (t, *J* = 7.4 Hz, 0.6H **18b**).
^{13}C NMR (101 MHz, CDCl$_3$) δ 178.8 (**18a** and **18b**), 153.4, 153.3, 135.3, 134.8, 126.0, 125.9, 124.8, 124.7, 122.7, 122.6, 121.7, 121.6, 36.4 (**18b**), 34.2 (**18a**), 23.2 (**18b**), 23.1 (**18a**), 13.9 (**18b**).
HRMS (ESI+) (m/z): $[M+H]^+$ calcd. for $C_{10}H_{11}NS$,177.0612; found:177.0617.

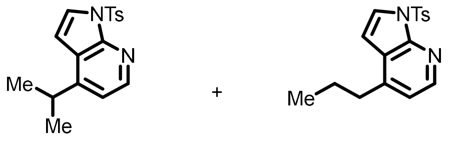

19a **19b**

***4-isopropyl-1-tosyl-1H-pyrrolo[2,3-b]pyridine* (19a, major) *and 4-propyl-1-tosyl-1H-pyrrolo[2,3-b]pyridine* (19b, minor).** Prepared according to GP2 from 4-bromo-1-tosyl-1H-pyrrolo[2,3-b]pyridine (105 mg, 0.3 mmol, 1.0 equiv.) and propane. The regioisomeric ratio (r.r) was determined by ^{1}H NMR of the crude mixture to be 3:1. Purified via flash column chromatography on silica gel (from Pentane:Ethyl Acetate 20:1 to 5:1) to afford the product as a white solid (43 mg, 46% yield).

^{1}H NMR (400 MHz, CDCl$_3$) δ 8.27 (d, *J* = 5.1 Hz, 0.8H **19a**), 8.24 (d, *J* = 5.0 Hz, 0.2H **19b**), 8.03 – 7.95 (*regioisomeric multiplet* 2H **19a**+**19b**), 7.63 – 7.58 (*regioisomeric multiplet* 1H **19a**+**19b**), 7.20 – 7.14 (*regioisomeric doublet*, *J* = 8.1 Hz, 2H **19a**+**19b**), 6.94 (d, *J* = 5.1 Hz, 0.8H **19a**), 6.89 (d, *J* = 5.0 Hz, 0.2H **19b**),

6.57 (d, *J* = 4.1 Hz, 0.8H **19a**), 6.54 (d, *J* = 4.0 Hz, 0.2H **19b**), 3.15 (hept, *J* = 6.9 Hz, 0.8H **19a**), 2.71 – 2.65 (m, 0.4H **19b**), 2.27 (*regioisomeric singlet*, 3H **19a**+**19b**), 1.62 (td, *J* = 13.2, 5.7 Hz, 0.4H **19b**), 1.22 (d, *J* = 7.0 Hz, 5H **19a**), 0.86 (t, *J* = 7.3 Hz, 0.6H **19b**).
^{13}C NMR (101 MHz, $CDCl_3$) δ 151.3, 147.4, 145.4, 145.3, 145.1, 145.1, 135.7, 129.7, 128.2, 128.1, 125.6, 125.5, 122.6, 121.6, 118.7, 115.4, 103.8, 103.7, 34.7 (**19b**), 31.2 (**19a**), 23.2 (**19b**), 22.6, 21.7 (**19a**), 14.0 (**19b**).
HRMS (ESI+) (m/z): $[M+H]^+$ calcd. for $C_{17}H_{18}N_2O_2S$, 314.1089; found: 314.1095.

Ethane:

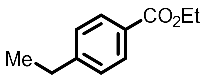

20

***ethyl 4-ethylbenzoate* (20).** Prepared according to GP3 from ethyl 4-bromobenzoate (69 mg, 0.3 mmol, 1.0 equiv.) and ethane. Purified via preparative TLC (Pentane:Toluene 1:1) to afford the product as a colorless oil (13 mg, 25% yield).

Characterization data are in accordance with literature.[7]
^{1}H NMR (300 MHz, $CDCl_3$) δ 7.96 (d, *J* = 8.2 Hz, 2H), 7.28 – 7.19 (m, 2H), 4.36 (q, *J* = 7.1 Hz, 2H), 2.69 (q, *J* = 7.6 Hz, 2H), 1.38 (t, *J* = 7.1 Hz, 3H), 1.24 (t, *J* = 7.6 Hz, 3H).
^{13}C NMR (101 MHz, $CDCl_3$) δ 166.7, 149.6, 129.7, 127.8, 60.7, 29.0, 15.3, 14.4.
HRMS (ESI) m/z calcd for $C_{11}H_{14}O_2$+ : [M]+ 178.0994; found: 178.0991.

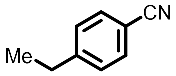

21

***4-ethylbenzonitrile* (21).** Prepared according to GP3 from 4-bromobenzonitrile (55 mg, 0.3 mmol, 1.0 equiv.) and ethane. Purified via flash column chromatography on silica gel (from Pentane to Pentane:Ethyl acetate 100:1) to afford the product as a colorless oil (11 mg, 29% yield).

Characterization data are in accordance with literature.[8]
^{1}H NMR (400 MHz, $CDCl_3$) δ 7.54 (d, *J* = 8.2 Hz, 2H), 7.27 (d, *J* = 8.1 Hz, 2H), 2.69 (q, *J* = 7.6 Hz, 2H), 1.23 (t, *J* = 7.6 Hz, 3H).
^{13}C NMR (101 MHz, $CDCl_3$) δ 149.9, 132.3, 128.8, 119.3, 109.6, 29.2, 15.1.
HRMS (ESI) m/z calcd for C_9H_9N + : [M]+ 131.0735; found: 131.0731.

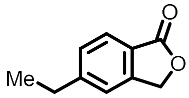

22

***5-ethylisobenzofuran-1(3H)-one* (22).** Prepared according to GP3 from methyl 6-bromoisobenzofuran-1(3H)-one (64 mg, 0.3 mmol, 1.0 equiv.) and ethane. Purified via preparative TLC (Pentane:Toluene 1:1) to afford the product as a colorless oil (12 mg, 25% yield).

^{1}H NMR (400 MHz, $CDCl_3$) δ 7.83 (d, *J* = 7.9 Hz, 1H), 7.36 (d, *J* = 7.9 Hz, 1H), 7.30 (s, 1H), 5.28 (s, 2H), 2.79 (q, *J* = 7.6 Hz, 2H), 1.29 (t, *J* = 7.6 Hz, 3H).
^{13}C NMR (101 MHz, $CDCl_3$) δ 171.3, 151.6, 147.3, 129.3, 125.8, 123.6, 121.3, 69.6, 29.5, 15.5.
HRMS (ESI) m/z calcd for $C_{10}H_{10}O_2$ + : [M]+ 162.0681; found: 162.0676.

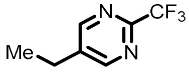

23

***5-ethyl-2-(trifluoromethyl)pyrimidine* (23).** Prepared according to GP3 from methyl 4-bromobenzoate (64.5 mg, 0.3 mmol, 1.0 equiv.) and ethane. Purified via flash column chromatography on silica gel from Pentane to Pentane:Ethyl Acetate 30:1) to afford the product as a colorless oil (19 mg, 36% yield).

^{1}H NMR (400 MHz, $CDCl_3$) δ 8.72 (s, 2H), 2.75 (q, *J* = 7.7 Hz, 2H), 1.32 (dd, *J* = 8.2, 7.1 Hz, 3H).
^{13}C NMR (101 MHz, $CDCl_3$) δ 157.4, 154.9 (q, *J* = 36.7 Hz), 139.2, 119.8 (q, *J* = 275.0 Hz), 23.7, 14.7.
^{19}F NMR (282 MHz, $CDCl_3$) δ -70.2.

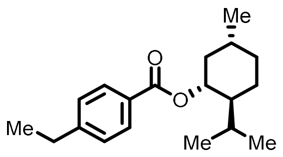

24

(1R,2S,5R)-2-isopropyl-5-methylcyclohexyl 4-ethylbenzoate **(24).** Prepared according to GP3 from (1R,2S,5R)-2-isopropyl-5-methylcyclohexyl 4-bromobenzoate (102 mg, 0.3 mmol, 1.0 equiv.) and ethane. Purified via Preparative TLC (Pentane:Toluene 1:1) to afford the product as a colorless oil (28 mg, 32% yield).

^{1}H NMR (400 MHz, $CDCl_3$) δ 7.96 (d, *J* = 8.2 Hz, 2H), 7.28 – 7.24 (m, 2H), 4.92 (td, *J* = 10.9, 4.3 Hz, 1H), 2.12 (d, *J* = 12.0 Hz, 1H), 2.00 – 1.91 (m, 1H), 1.73 (d, *J* = 12.3 Hz, 2H), 1.56 (s, 6H), 1.28 – 1.24 (m, 3H), 1.15 – 1.05 (m, 2H), 0.94 – 0.89 (m, 5H), 0.79 (d, *J* = 7.0 Hz, 3H).
^{13}C NMR (101 MHz, $CDCl_3$) δ 166.2, 149.5, 129.7, 128.4, 127.8, 74.6, 47.3, 41.0, 34.4, 31.5, 29.7, 29.0, 26.5, 23.7, 22.1, 20.8, 16.5, 15.3.
HRMS (ESI) m/z calcd for $C_{19}H_{28}O_2$ + : [M]+ 288.2089; found: 288.2093.

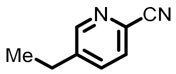

25

5-ethylpicolinonitrile **(25).** Prepared according to GP3 from 5-bromopicolinonitrile (55 mg, 0.3 mmol, 1.0 equiv.) and ethane. Purified via flash column chromatography on silica gel (from Pentane to Pentane:Ethyl Acetate 50:1) to afford the product as a pale yellow oil (16 mg, 41% yield).

^{1}H NMR (400 MHz, $CDCl_3$) δ 8.57 (s, 1H), 7.67 – 7.59 (m, 2H), 2.75 (q, *J* = 7.6 Hz, 2H), 1.29 (t, *J* = 7.6 Hz, 3H).
^{13}C NMR (101 MHz, $CDCl_3$) δ 151.3, 143.7, 136.1, 131.5, 128.4, 117.6, 26.4, 14.9.
HRMS (ESI) m/z calcd for $C_8H_8N_2$ + : [M]+ 132.0687; found: 132.0681.

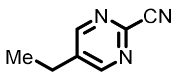

26

5-ethylpyrimidine-2-carbonitrile **(26).** Prepared according to GP3 from 5-bromopyrimidine-2-carbonitrile (55 mg, 0.3 mmol, 1.0 equiv.) and ethane. Purified via flash column chromatography on silica gel (from Pentane to Pentane:Ethyl Acetate 20:1) to afford the product as a colorless oil (9 mg, 22% yield).

^{1}H NMR (400 MHz, $CDCl_3$) δ 8.69 (s, 2H), 2.76 (q, *J* = 7.6 Hz, 2H), 1.33 (t, *J* = 7.6 Hz, 3H).
^{13}C NMR (101 MHz, $CDCl_3$) δ 157.5, 143.1, 139.9, 115.9, 24.0, 14.5.
HRMS (ESI) m/z calcd for $C_7H_7N_3$ + : [M]+ 133.0640; found: 133.0649.

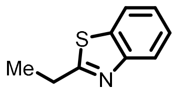

27

***2-ethylbenzo[d]thiazole* (27).** Prepared according to GP3 from 2-bromobenzo[d]thiazole (64 mg, 0.3 mmol, 1.0 equiv.) and ethane. Purified via flash column chromatography on silica gel (from Pentane to Pentane:Ethyl Acetate 20:1) to afford the product as a colorless oil (6 mg, 12% yield).

Characterization data are in accordance with literature.[9]
^{1}H NMR (300 MHz, $CDCl_3$) δ 8.03 – 7.92 (m, 1H), 7.84 (ddd, *J* = 7.9, 1.4, 0.7 Hz, 1H), 7.45 (ddd, *J* = 8.2, 7.2, 1.3 Hz, 1H), 7.38 – 7.30 (m, 1H), 3.16 (q, *J* = 7.6 Hz, 2H), 1.48 (t, *J* = 7.6 Hz, 3H).
^{13}C NMR (101 MHz, $CDCl_3$) δ 173.7, 153.5, 135.2, 126.0, 124.7, 122.7, 121.6, 27.9, 13.9.
HRMS (ESI) m/z calcd for C_9H_9NS + : [M]+ 163.0456; found: 163.0459.

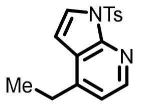

28

***4-ethyl-1-tosyl-1H-pyrrolo[2,3-b]pyridine* (28).** Prepared according to GP3 from 4-bromo-1-tosyl-1H-pyrrolo[2,3-b]pyridine (105 mg, 0.3 mmol, 1.0 equiv.) and ethane. Purified via flash column chromatography on silica gel (from Pentane to Pentane:Ethyl Acetate 20:1) to afford the product as a light yellow solid (12 mg, 13% yield).

^{1}H NMR (400 MHz, $CDCl_3$) δ 8.33 (d, *J* = 5.0 Hz, 1H), 8.07 (d, *J* = 8.3 Hz, 2H), 7.69 (d, *J* = 4.0 Hz, 1H), 7.31 – 7.24 (m, 2H), 7.00 (d, *J* = 4.9 Hz, 1H), 6.62 (d, *J* = 4.1 Hz, 1H), 2.83 (q, *J* = 7.6 Hz, 2H), 2.36 (s, 3H), 1.28 (t, *J* = 7.6 Hz, 3H).
^{13}C NMR (101 MHz, $CDCl_3$) δ 147.3, 146.8, 145.3, 145.1, 135.7, 129.7, 128.2, 125.7, 122.2, 117.9, 103.7, 25.7, 21.8, 14.1.
HRMS (ESI) m/z calcd for $C_{16}H_{17}N_2O_2S$ + : [M+H]+ 301.1005; found: 301.1008.

7. Mechanistic experimental studies

7.1 Evaluation of the KIE

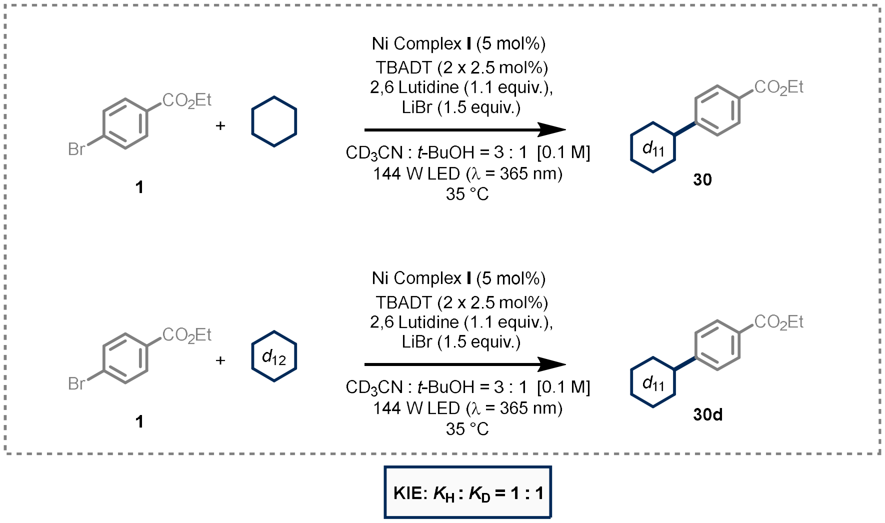

To a nitrogen-purged, screw-capped vial, fitted with a rubber septum and charged with nickel complex **I** (7.3 mg, 15 μmol, 5 mol%), TBADT (25 mg, 7.5 μmol, 2.5 mol%), LiBr (39 mg, 0.45 mmol, 1.5 equiv.), 2,6-dimethylpyridine (38 μL, 0.33 mmol, 1.1 equiv.), methyl 4-bromobenzoate (64.5 mg, 0.3 mmol, 1 equiv.) and cyclohexane (1.5 mmol, 165 μL, 5 equiv.) or cyclohexane-d_{12}, solubilized in 2.25 mL di CD_3CN + 0.75 mL *t*-BuOH (3:1 volume ratio 0.1 M).
The reaction mixture is pumped over the Signify Eagle reactor (365 nm, 144 W output power, FEP capillary: 0.5 mm ID, 2.5 mL) with appropriate flow rate to reach residence time of 30, 60, 90 and 120 seconds, and collected at the end of the reactor, and evaporated in vacuo. Trichloroethylene (28 μL, 0.3 mmol) was added as the internal standard and an aliquot of the resulting solution was taken to be directly analyzed by ^{1}H NMR.

The magnitude of the kinetic isotope effect, calculated plotting the kinetic profile of the two reactions (with cyclohexane and cyclohexane-d_{12}, respectively), was determined to be 1.

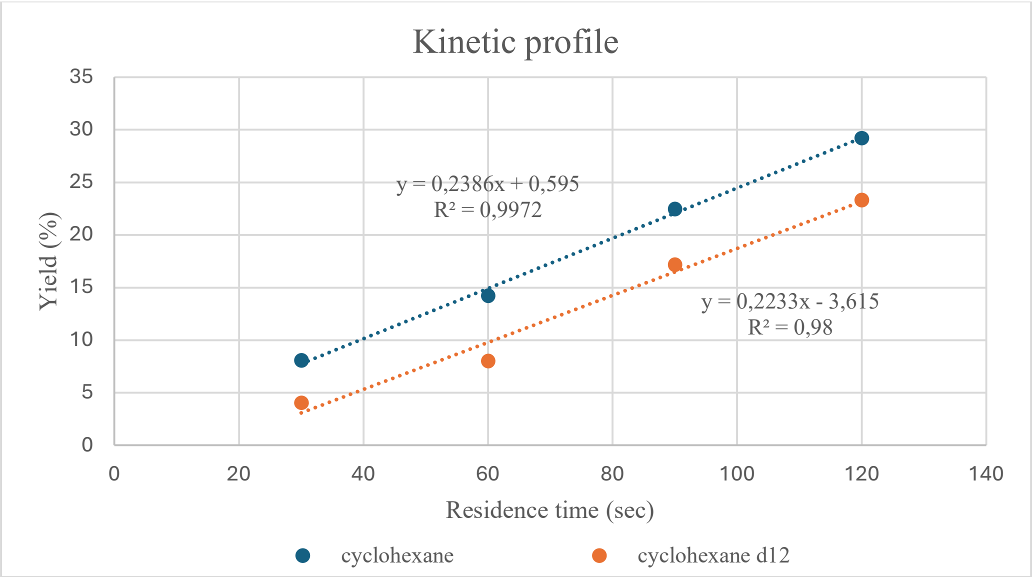

Figure S 7 Kinetic Isotope Experiment to evaluate the rate of the reaction when using cyclohexane or cyclohexane-d_{12}

7.2 Evaluation of the selectivity: Arylation of n-butane

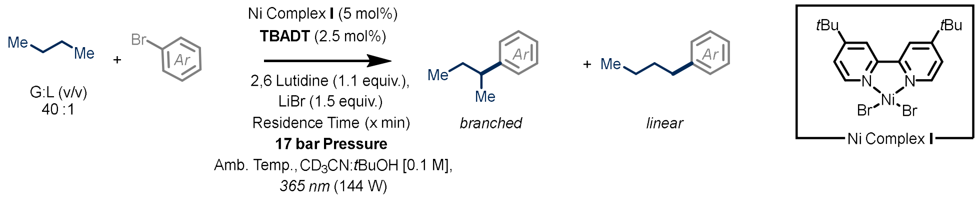

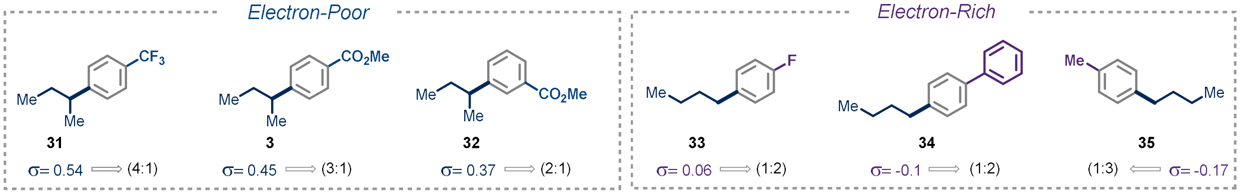

Reported herein, the evaluation of the electronic properties of the aryl bromide (substituent) on the selectivity (branched vs linear) of the reaction. Hammett values were taken from the literature. [10]

To a nitrogen-purged, screw-capped vial, fitted with a rubber septum and charged with nickel complex **I** (7.3 mg, 15 µmol, 5 mol%), TBADT (25 mg, 7.5 µmol, 2.5 mol%), LiBr (39 mg, 0.45 mmol, 1.5 equiv.), 2,6-dimethylpyridine (38 µL, 0.33 mmol, 1.1 equiv.), the aryl bromide (0.3 mmol, 1 equiv.) in 2.25 mL CD_3CN + 0.75 mL *t*-BuOH (3:1 volume ratio 0.1 M). The stock solution is charged in a gastight syringe, positioned in a syringe pump and combined with a stream of butane gas (120 mL, 5.14 mmol, 17 equiv.) through a T-mixer into a filling loop, with a liquid flow rate of 0.1 $mL \cdot min^{-1}$ and a butane gas flow rate of 4 $mL \cdot min^{-1}$ (40 : 1 = gas : liquid). No back pressure regulator was used for the loop filling step.
The system is pressurized to 34 bar using an HPLC pump and the reaction mixture is pumped over the Signify Eagle reactor (365 nm, 144 W output power, FEP capillary: 0.5 mm ID, 2.5 mL) at a flow rate of 0.083 mL/min, resulting in a residence time of 30 min.
After 29 min the TBADT stock solution is pumped over through a T mixer connected at the end of the first coil at a flow rate of 0.041 $mL \cdot min^{-1}$ (the total volume of TBADT stock solution used is 1.5 mL, 7.5 µmol, 2.5 mol%). The sum of the two flows rate (recirculation) is 0,125 mL/min and in the second coil (FEP capillary: 0.5 mm ID, 2.5 mL) this results in a residence time of 20 min.
The obtained reaction mixture is collected into vial and submitted to GC-Ms analysis to determine the branched to linear ration.
The ratio value was determined, analyzing the reaction at ≤ 50% conversion of the aryl bromide. For electron poor substrate (4-CF_3, 4-CO_2Me, 3-CO_2Me) the recirculation was omitted and a total residence time of 5 min was used in order to have comparable conversions among all tested substrates (*n.b.* for electron rich substrates the recirculation step was performed).

Table S8. Influence of the aryl substituent on the branched/linear ratio

Substituent	σ value	Ratio branched/ linear
4-CF_3	0.54	4 : 1
4-CO_2Me	0.45	3 : 1
3-CO_2Me	0.37	2 : 1
4-F	0.06	1 : 2
4-Ph	-0.1	1 : 2
4-Me	-0.17	1 : 3

These findings, supported by DFT calculation (See Figure S10), suggest that the oxidative addition significantly impacts the selectivity of the reaction (branched vs linear). Electron-deficient aryl bromides tend to undergo easy oxidative addition prior to radical isomerization (via β-hydride elimination and migratory insertion), leading to predominance of branched selectivity in the reaction. Conversely, electron-rich aryl bromides exhibit lower reactivity due to heightened energy barrier at the rate-determining step (RDS), making such isomerization pathway more facile and resulting in linear selectivity.

8. Computational Studies

8.1 Computational Methods

All geometry optimizations and frequency calculations for reported structures were performed using the B3LYP functional[11,12] with the def2-SVP basis set[13,14] using the Gaussian 16 (G16) program.[15] Dispersion interactions were included using Grimme's DFT-D3 correction.[16] The CPCM solvent effects were incorporated into all calculations with acetonitrile as the solvent.[17-21] This level is referred to as CPCM(MeCN)/B3LYP-D3/def2-SVP. Through vibrational analysis, all stationary points have been verified to be minima (zero imaginary frequencies) or transition structures (one imaginary frequency). The character of the normal mode associated with the imaginary frequency has been analyzed to ensure it resembles the reaction under consideration. Potential energies were refined by means of single point calculations using the B3LYP functional[11,12] and Grimme's DFT-D3 correction[16] with the def2-TZVPP basis set. [13,14] This level is denoted CPCM(MeCN)/B3LYP-D3/def2-TZVPP//CPCM(MeCN)/B3LYP-D3/def2-SVP. The reported Gibbs free energies in solution are calculated by adding thermal corrections computed at T = 298.15 K and P = 1 atm from vibrational frequencies obtained through numerical differentiation of the analytical gradient at CPCM(MeCN)/B3LYP-D3/def2-SVP and a standard concentration (1 mol L^{-1}) to the total electronic energy at CPCM(MeCN)/B3LYP-D3/def2-TZVPP. Optimized structures were illustrated using CYLview20. [22]

Quantitative analyses of the activation barriers associated with the transition states of the oxidative addition were obtained by means of the activation strain model (ASM), which involves the decomposition of the electronic energy ΔE into the strain energy ΔE_{strain} associated with the structural deformation of the Ni(I)−alkyl complex and the aryl halide from their equilibrium geometry and the interaction energy ΔE_{int} between these deformed reactants [Eq. 1]. [33-36] The ΔE_{strain} is determined by the rigidity of the reactants and by the extent to which they must deform to achieve the geometry of the transition structure. The ΔE_{int} is usually stabilizing and is related to the electronic structure of the reactants and how they are mutually oriented over the course of the reaction.

$$\Delta E = \Delta E_{strain} + \Delta E_{int} \qquad (1)$$

The interaction energy between the deformed reactants can be further analyzed in terms of quantitative Kohn-Sham molecular orbital theory (KS-MO) together with a canonical energy decomposition analysis (EDA). [27-30] The EDA computations were performed using the B3LYP functional[11, 12] with the TZ2P basis set[31] using the Amsterdam Density Functional (ADF) software. [32-34] Dispersion interactions were included using Grimme's DFT-D3 correction. [16] The zeroth-order regular approximation (ZORA) was used to account for scalar relativistic effects.[35-37]. The EDA decomposes the interaction energy (ΔE_{int}) into the following four chemically meaningful energy terms: classical electrostatic interaction (ΔV_{elstat}), destabilizing steric Pauli repulsion interactions (ΔE_{Pauli}), stabilizing orbital interactions that account, among others, for HOMO–LUMO interactions (ΔE_{oi}), and the dispersion interaction (ΔE_{disp}) between the deformed Ni(I)−alkyl complex and aryl halide [Eq. 2].

$$\Delta E_{int} = \Delta V_{elstat} + \Delta E_{Pauli} + \Delta E_{oi} + \Delta E_{disp} \qquad (2)$$

8.2 Computational Results

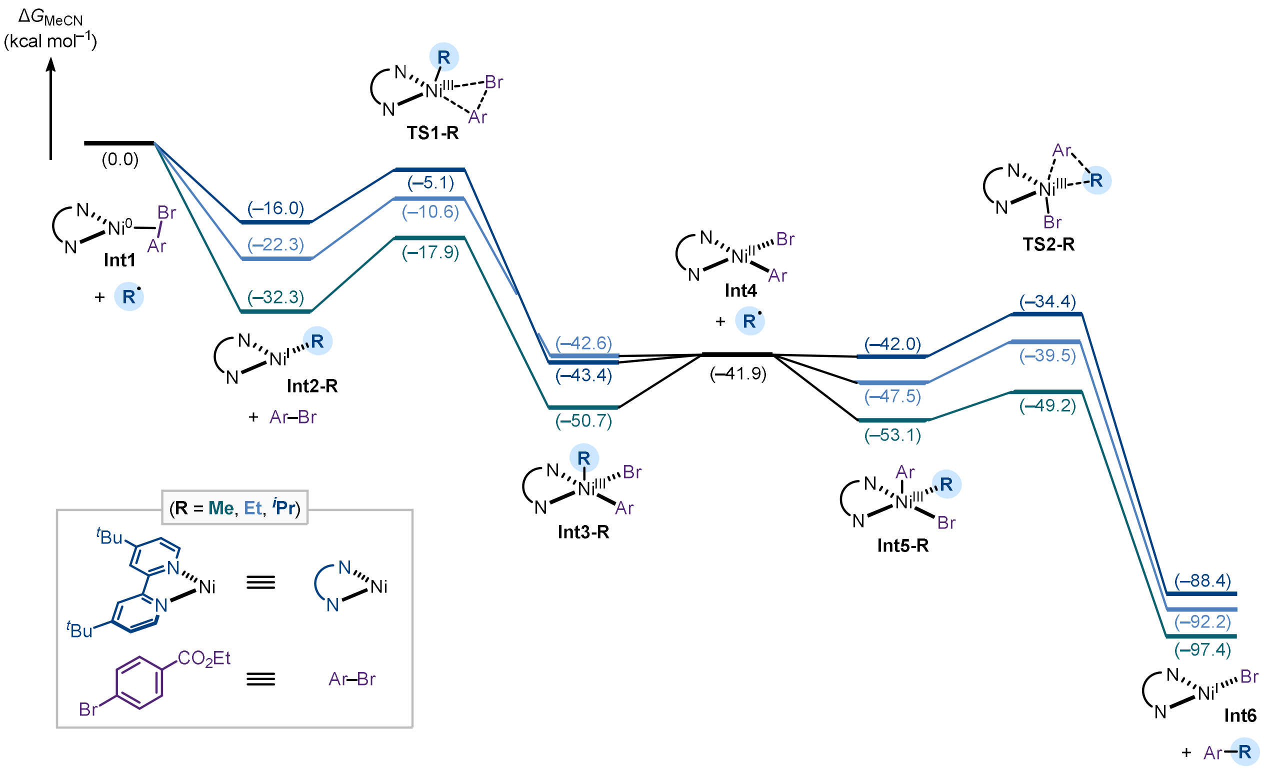

Figure S8: Complete potential energy surface (ΔG [kcal mol^{-1}]) of the C(sp^2)–C(sp^3) cross-coupling of gaseous alkanes and aryl bromides computed at CPCM(MeCN)/B3LYP-D3/def2-TZVPP//CPCM(MeCN)/B3LYP-D3/def2-SVP level of theory. Energies (kcal mol^{-1}) are provided in the insert.

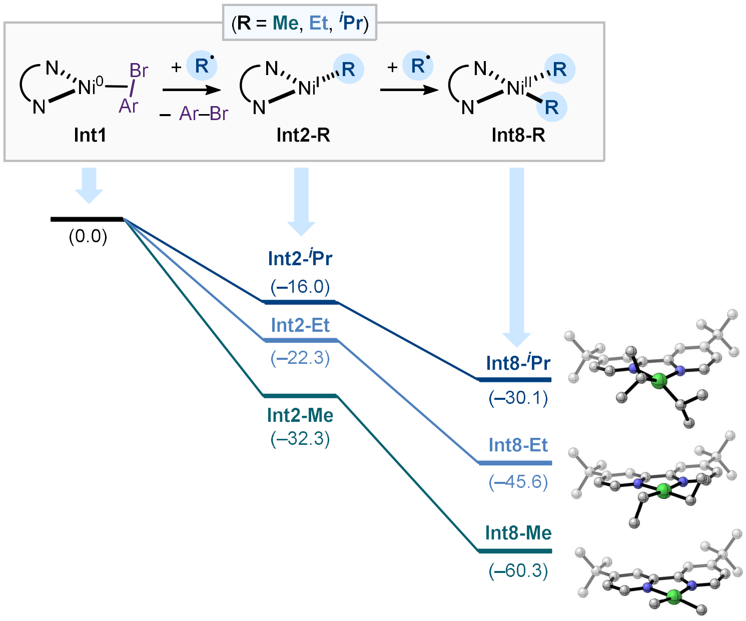

Figure S9: Possible catalyst decomposition pathway [38] through radical coordination (Δ*G* [kcal mol^{-1}]) computed at CPCM(MeCN)/B3LYP-D3/def2-TZVPP//CPCM(MeCN)/B3LYP-D3/def2-SVP level of theory. Energies (kcal mol^{-1}) are provided in the insert.

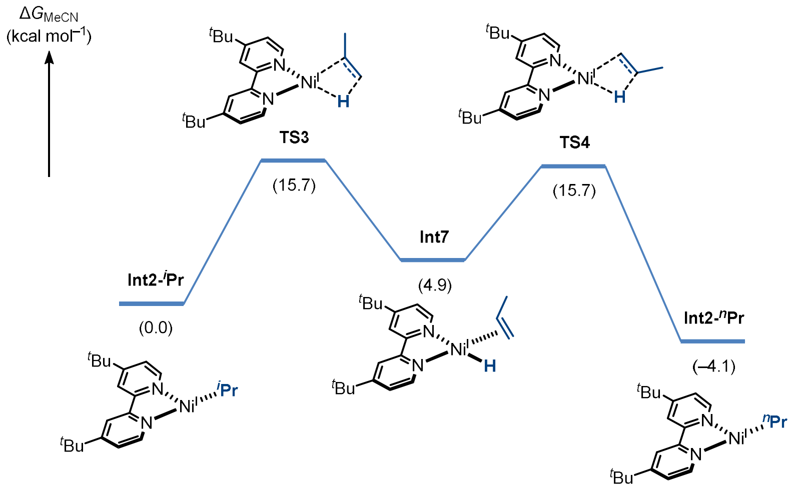

Figure S10: Alkyl radical scrambling mechanism with a propyl radical (Δ*G* [kcal mol^{-1}]) computed at CPCM(MeCN)/B3LYP-D3/def2-TZVPP//CPCM(MeCN)/B3LYP-D3/def2-SVP level of theory. Energies (kcal mol^{-1}) are provided in the insert.

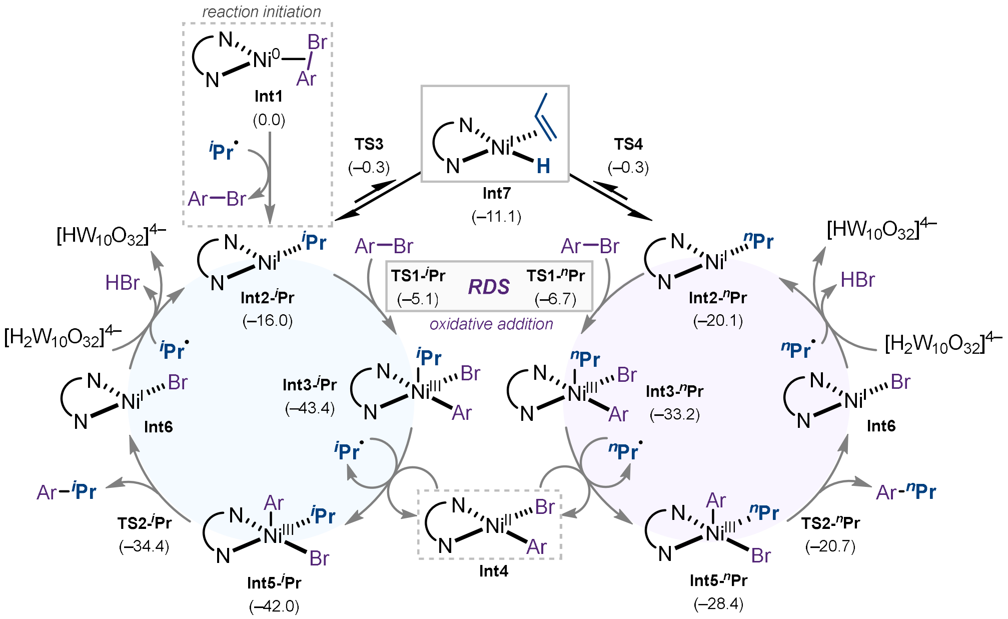

Figure S11: Complete overview of the alkyl radical scrambling mechanism with a propyl radical (ΔG [kcal mol^{-1}]) computed at CPCM(MeCN)/B3LYP-D3/def2-TZVPP//CPCM(MeCN)/B3LYP-D3/def2-SVP level of theory. Energies (kcal mol^{-1}) are provided in the insert.

8.3 Computational Details

Table S9. Cartesian coordinates (in Å), energies (in kcal mol^{-1}), and number of imaginary frequencies of all stationary points, computed at CPCM(MeCN)/B3LYP-D3/def2-TZVPP//CPCM(MeCN)/B3LYP-D3/def2-SVP.

Int1

CPCM(MeCN)/B3LYP-D3/def2-TZVPP//CPCM(MeCN)/B3LYP-D3/def2-SVP

E = -5352.603760

G = -5352.153894

N_{imag} = 0

C	2.24911000	-3.02358200	-0.74066900
C	3.30479200	-2.11037500	-0.87721500
C	3.02429000	-0.77678700	-0.52930600
C	1.75757900	-0.40739700	-0.08006900
C	1.00327800	-2.59074500	-0.28450800
C	1.37401300	0.97308400	0.30673700
C	2.22746000	2.07371600	0.25011800
C	1.78393800	3.35219600	0.63268300
C	0.45150400	3.44531300	1.06136900
C	-0.35206000	2.30584400	1.09214000
H	2.36923700	-4.07843200	-0.98230600
H	0.17089400	-3.28781000	-0.15461000
H	0.01450400	4.39166800	1.37561100
H	-1.39352500	2.35761700	1.41525300
N	0.08847400	1.09532800	0.72927600
N	0.75533400	-1.31595100	0.03841600
Ni	-0.92820700	-0.54824600	0.66005600
Br	-5.02443400	1.43513800	-1.89546000
C	4.70447400	-2.49941100	-1.37089400
C	2.73917800	4.55094700	0.56620000
C	2.06304200	5.85338300	1.02690500
H	2.78230000	6.68409300	0.96344000
H	1.19983000	6.11215700	0.39410700
H	1.72062200	5.78919200	2.07149900
C	3.95503800	4.27547800	1.48093500
H	4.51036000	3.37764500	1.16981500
H	4.65178800	5.12801700	1.44570500
H	3.63720400	4.13515600	2.52617300
C	3.21756800	4.73239800	-0.89290400
H	3.75457900	3.84640800	-1.26416800
H	2.36618500	4.92224800	-1.56544200
H	3.90465000	5.59098100	-0.95819900
C	5.73577600	-2.17170900	-0.26648100
H	6.74782400	-2.44521800	-0.60490600
H	5.74560400	-1.09990900	-0.01666600
H	5.51813200	-2.73502700	0.65468200
C	5.03262600	-1.68723600	-2.64502700
H	5.02925700	-0.60308100	-2.45572600
H	6.03427600	-1.95815100	-3.01491300
H	4.30366800	-1.89675400	-3.44373200
C	4.80209100	-3.99754000	-1.70529000
H	4.60097600	-4.62709800	-0.82458700
H	4.10066900	-4.28453000	-2.50414200

H	5.81900200	-4.23134100	-2.05561400
H	3.80223700	-0.01872300	-0.60962200
H	3.25000800	1.93500800	-0.09828200
C	-4.00011500	0.19462700	-0.83553900
C	-3.69606800	-1.08892900	-1.40936500
C	-3.59629300	0.53806900	0.42434200
C	-2.98530900	-1.99919100	-0.67460800
H	-4.05205600	-1.33356300	-2.41163700
H	-3.88837700	1.50022700	0.85257000
C	-2.50719600	-1.70381000	0.66023200
H	-2.78543000	-2.99567800	-1.07584400
C	-2.77270300	-0.35723600	1.21101000
C	-2.01405700	-2.81364700	1.47529200
H	-2.83385600	-0.22950100	2.29945200
O	-1.92943000	-2.51823100	2.80023400
O	-1.66975700	-3.91125400	1.04589900
C	-1.31511600	-3.50135800	3.62905300
H	-1.85737000	-4.45855900	3.58358400
H	-1.34087900	-3.10345800	4.65151300
H	-0.27154900	-3.67890400	3.32277800

Ar-Br (1)
CPCM(MeCN)/B3LYP-D3/def2-TZVPP//CPCM(MeCN)/B3LYP-D3/def2-SVP
E = -3033.912713
G = -3033.817106
N_{imag} = 0

C	-0.57383100	1.36038700	0.00004800
C	0.81769000	1.27474200	0.00005500
C	1.41894900	0.01068800	0.00002100
C	0.65171400	-1.15954800	0.00000500
C	-0.74012300	-1.05984300	0.00001000
C	-1.36224600	0.19902800	0.00000900
H	1.42917900	2.17819700	0.00010000
Br	3.32305500	-0.11949400	-0.00001800
H	1.13564400	-2.13727800	0.00000600
H	-1.34840800	-1.96480100	-0.00000800
C	-2.84715000	0.35537300	-0.00001800
H	-1.06638000	2.33434400	0.00005900
O	-3.48971200	-0.82078700	0.00008900
O	-3.42019000	1.42545600	-0.00007500
C	-4.92062800	-0.77270400	-0.00004300
H	-5.26133900	-1.81466100	-0.00112400
H	-5.29123900	-0.24886000	-0.89402800
H	-5.29140900	-0.25073800	0.89498800

Me radical
CPCM(MeCN)/B3LYP-D3/def2-TZVPP//CPCM(MeCN)/B3LYP-D3/def2-SVP
E = -39.86032105
G = -39.849840
N_{imag} = 0

C	0.00000000	0.00000000	-0.00000200
H	0.00000000	1.09142800	0.00000500
H	0.94520400	-0.54571400	0.00000500

H	-0.94520400	-0.54571400	0.00000500

Et radical
CPCM(MeCN)/B3LYP-D3/def2-TZVPP//CPCM(MeCN)/B3LYP-D3/def2-SVP
E = -79.19587414
G = -79.161379
$\boldsymbol{N}_{\mathbf{imag}}$ = 0

C	-0.79494400	0.00000200	-0.02417500
H	-1.35710600	-0.93545700	0.05230400
H	-1.35720600	0.93539500	0.05232500
C	0.69335800	0.00001700	-0.00201300
H	1.09281500	-0.00074600	1.03564100
H	1.11550400	0.89439900	-0.49095700
H	1.11551000	-0.89370900	-0.49218200

***i*-Pr radical**
CPCM(MeCN)/B3LYP-D3/def2-TZVPP//CPCM(MeCN)/B3LYP-D3/def2-SVP
E = -118.5326834
G = -118.472694
$\boldsymbol{N}_{\mathbf{imag}}$ = 0

C	0.00001900	0.53519900	-0.05543900
C	1.29771500	-0.19758700	0.00293200
H	-0.00010300	1.61666600	0.12225800
C	-1.29768700	-0.19762500	0.00291300
H	-2.15274900	0.44319300	-0.26620100
H	-1.50373600	-0.59449300	1.02161200
H	-1.29956400	-1.07691900	-0.66772600
H	1.29961100	-1.07676700	-0.66789500
H	1.50356500	-0.59490000	1.02152900
H	2.15269300	0.44330000	-0.26601300

***n*-Pr radical**
CPCM(MeCN)/B3LYP-D3/def2-TZVPP//CPCM(MeCN)/B3LYP-D3/def2-SVP
E = -118.5268744
G = -118.466065
$\boldsymbol{N}_{\mathbf{imag}}$ = 0

C	1.22941100	-0.24103500	-0.03437000
C	-1.30587600	-0.29532500	-0.03318700
H	-2.27966500	0.14898300	-0.25888400
H	-1.28516800	-1.33923700	0.29659900
H	-0.10659500	1.32353900	-0.74492500
H	-0.08905600	1.13124400	0.99638100
H	1.30751600	-0.78161600	-0.99195400
C	-0.08048800	0.55043400	0.04649200
H	1.28914400	-0.98817600	0.77483900
H	2.10553900	0.42082000	0.05433600

Ar-Me (29)
CPCM(MeCN)/B3LYP-D3/def2-TZVPP//CPCM(MeCN)/B3LYP-D3/def2-SVP
E = -499.6670653
G = -499.534942
$\boldsymbol{N}_{\mathbf{imag}}$ = 0

C	-0.57674200	1.29969200	-0.00417600

C	-1.96329300	1.15505600	-0.00921700
C	-2.55840200	-0.11873700	-0.01036400
C	-1.71586200	-1.24528900	-0.01125200
C	-0.32846800	-1.10943200	-0.00621400
C	0.25529600	0.16905800	-0.00201100
H	-2.59816300	2.04515600	-0.01396000
C	-4.05681200	-0.27964600	0.01595800
H	-2.15785800	-2.24544400	-0.01774300
H	0.31033700	-1.99349600	-0.00823800
C	1.73097200	0.37551800	0.00129100
H	-0.11870300	2.29067600	-0.00439000
O	2.41705000	-0.77907700	0.00146600
O	2.27292100	1.46296800	0.00393900
C	3.84396600	-0.67865000	0.00564800
H	4.22346300	-1.70733200	0.00717000
H	4.19445500	-0.14221200	0.90050300
H	4.19967700	-0.14308900	-0.88769900
H	-4.38045100	-1.13405000	-0.59833500
H	-4.56970600	0.62446100	-0.34388800
H	-4.40674700	-0.47122200	1.04536700

Ar-Et (20)
CPCM(MeCN)/B3LYP-D3/def2-TZVPP//CPCM(MeCN)/B3LYP-D3/def2-SVP
E = -538.998536
G = -538.838201
N_{imag} = 0

C	-0.11185300	1.33970100	-0.10581800
C	-1.49480200	1.22897700	-0.23659000
C	-2.11208800	-0.02911500	-0.35317000
C	-1.29757100	-1.17476600	-0.33218500
C	0.08728800	-1.07322200	-0.20139100
C	0.69331100	0.18914500	-0.08648200
H	-2.11033700	2.13275300	-0.25211600
C	-3.61581100	-0.14914800	-0.44140000
H	-1.75853900	-2.16231500	-0.42311300
H	0.70620200	-1.97126100	-0.19032500
C	2.16720700	0.35959800	0.05331000
H	0.36551300	2.31769000	-0.01911600
O	2.82706700	-0.81002900	0.06329900
O	2.72854800	1.43267700	0.15009800
C	4.24991600	-0.74355700	0.19575400
H	4.60726900	-1.78010300	0.18538300
H	4.53089500	-0.25329200	1.14028100
H	4.69317600	-0.17972600	-0.63912100
H	-3.87893800	-1.03319100	-1.04538100
H	-4.02405600	0.72931800	-0.96781600
C	-4.27430100	-0.26428700	0.94292200
H	-3.90475300	-1.15144500	1.48201000
H	-5.36880700	-0.35021500	0.85227000
H	-4.05032700	0.62063700	1.56016600

Ar-^{i}Pr (2)
CPCM(MeCN)/B3LYP-D3/def2-TZVPP//CPCM(MeCN)/B3LYP-D3/def2-SVP

E = -578.3304668
G = -578.143551
N_{imag} = 0

C	0.17863500	1.24070800	-0.00034100
C	-1.20064200	1.04956700	-0.00020600
C	-1.75369200	-0.24504800	0.00032500
C	-0.87349000	-1.33960300	0.00073200
C	0.51052800	-1.15779800	0.00059000
C	1.04981300	0.13815100	0.00004900
H	-1.85862200	1.92197400	-0.00053200
C	-3.25976700	-0.45539100	0.00033300
H	-1.28077800	-2.35434100	0.00118300
H	1.17801400	-2.02043300	0.00091500
C	2.51772000	0.39536500	-0.00009400
H	0.60294500	2.24660900	-0.00074000
O	3.24353900	-0.73452200	0.00011500
O	3.02125800	1.50101800	-0.00039300
C	4.66619500	-0.58432000	-0.00001600
H	5.08128500	-1.59917900	-0.00023800
H	5.00040300	-0.03651200	0.89421000
H	5.00021800	-0.03616400	-0.89409200
H	-3.43481100	-1.54462700	0.00128200
C	-3.91055600	0.11828400	-1.27002300
C	-3.91113600	0.12069900	1.26924400
H	-3.46550600	-0.31173100	2.17887000
H	-4.99163600	-0.09525300	1.28016600
H	-3.78832500	1.21503800	1.31942100
H	-3.46443700	-0.31587600	-2.17857200
H	-3.78771500	1.21252400	-1.32216800
H	-4.99105700	-0.09768900	-1.28103000

Int2-Me
CPCM(MeCN)/B3LYP-D3/def2-TZVPP//CPCM(MeCN)/B3LYP-D3/def2-SVP
E = -2358.600097
G = -2358.238083
N_{imag} = 0

Ni	-0.06289400	3.00463400	0.00005200
H	5.57679000	-2.61513800	-1.27775200
H	5.44560700	-0.84195900	-1.29648900
H	-2.22523200	-3.57447400	-0.89346100
H	4.25185000	-1.82900300	-2.17756700
H	-5.23783500	-3.13060000	1.27881200
H	-5.27526400	-1.35302100	1.29614300
H	3.98642300	-4.02827900	-0.00027300
H	2.61513000	-3.33697000	-0.89286900
H	2.61518000	-3.33716900	0.89252900
H	4.25177100	-1.82929800	2.17747500
N	1.24493100	1.55609400	0.00015200
C	2.99693400	-0.67559500	0.00002900
C	2.58362400	1.71600200	0.00008700
C	0.75847300	0.28584600	0.00018000
C	1.60643600	-0.82946300	0.00010400
C	3.47408000	0.65309700	0.00002400

N	-1.32649800	1.41487200	0.00067300
C	-2.85286400	-0.95784000	0.00012800
C	-2.66507800	1.45909500	0.00080000
C	-0.71711400	0.20816700	0.00029100
C	-1.45349100	-0.98566700	0.00000100
C	-3.45640700	0.31688900	0.00055800
H	2.93778700	2.74973000	0.00006800
H	1.16223300	-1.82195600	0.00013700
H	4.54336800	0.86936400	-0.00002400
H	-3.10615500	2.45960900	0.00110800
H	-0.92101200	-1.93367300	-0.00036400
H	-4.54141400	0.42919900	0.00066300
C	-0.94178600	4.78539300	-0.00101100
H	-3.99315300	-2.22184300	2.17754600
H	5.44557800	-0.84216100	1.29657100
C	-3.71643000	-2.22647100	-0.00019500
C	-2.86630300	-3.50848100	-0.00059400
C	-4.60754100	-2.22711700	1.26324500
C	-4.60765800	-2.22639300	-1.26354700
C	3.97550100	-1.85703800	-0.00005300
C	4.86354000	-1.77560300	1.26295200
C	3.24770700	-3.21225900	-0.00017400
C	4.86357900	-1.77541400	-1.26301000
H	-5.23799900	-3.12983500	-1.27955000
H	-3.99335500	-2.22064800	-2.17790300
H	-5.27534000	-1.35224300	-1.29590900
H	-3.52692500	-4.38887500	-0.00077600
H	-2.22510500	-3.57493700	0.89214800
H	5.57672800	-2.61534900	1.27761100
H	-1.59703200	4.94816400	-0.88360000
H	-0.23506500	5.64226100	-0.00185800
H	-1.59676500	4.94971000	0.88147900

Int2-Et
CPCM(MeCN)/B3LYP-D3/def2-TZVPP//CPCM(MeCN)/B3LYP-D3/def2-SVP
E = -2397.921976
G = -2397.533674
N_{imag} = 0

Ni	-0.06562100	2.74851600	0.00003600
H	5.58375700	-2.85866400	-1.27760700
H	5.44867000	-1.08577900	-1.29659100
H	-2.21246800	-3.83606300	-0.89401200
H	4.25705300	-2.07554600	-2.17747400
H	-5.22576600	-3.40220700	1.27943400
H	-5.26890600	-1.62473600	1.29681600
H	3.99635900	-4.27502100	0.00009900
H	2.62355000	-3.58673300	-0.89252600
H	2.62366100	-3.58668500	0.89284700
H	4.25713200	-2.07535000	2.17752600
N	1.24333700	1.30376100	-0.00009200
C	2.99968900	-0.92440500	-0.00001300
C	2.58199600	1.46646900	-0.00020700
C	0.75893000	0.03238200	0.00006700

C	1.60962700	-1.08108700	0.00011800
C	3.47438400	0.40548300	-0.00017900
N	-1.32942000	1.15666100	0.00040200
C	-2.84826600	-1.22159700	-0.00002300
C	-2.66837800	1.19610200	0.00042600
C	-0.71615700	-0.04848100	0.00015500
C	-1.44903400	-1.24477200	-0.00006200
C	-3.45597700	0.05134900	0.00021900
H	2.93397700	2.50096500	-0.00033500
H	1.16764500	-2.07457800	0.00028600
H	4.54326500	0.62378200	-0.00028300
H	-3.11347500	2.19486300	0.00063000
H	-0.91352000	-2.19106600	-0.00029600
H	-4.54134900	0.16013900	0.00025500
C	-1.00938400	4.49933800	-0.00012400
H	-3.98366800	-2.48944500	2.17769500
H	5.44866200	-1.08559600	1.29651000
C	-3.70774800	-2.49301900	-0.00014300
C	-2.85351200	-3.77230100	-0.00095800
C	-4.59838800	-2.49668700	1.26362700
C	-4.59952700	-2.49588900	-1.26309000
C	3.98077200	-2.10376900	0.00003400
C	4.86873300	-2.02036000	1.26296200
C	3.25591300	-3.46056200	0.00011800
C	4.86868900	-2.02050700	-1.26293600
H	-5.22687600	-3.40142700	-1.27890800
H	-3.98564400	-2.48803700	-2.17771700
H	-5.27014800	-1.62397900	-1.29513700
H	-3.51126700	-4.65484600	-0.00105200
H	-2.21181400	-3.83680900	0.89156900
H	5.58384400	-2.85848000	1.27767600
H	-1.69494200	4.56804600	-0.87459000
C	-0.10163400	5.74486000	-0.00022700
H	-1.69507900	4.56825100	0.87421100
H	0.56486900	5.76249500	0.88125600
H	-0.64683000	6.71353100	-0.00033200
H	0.56489400	5.76230900	-0.88169200

Int2-^{i}Pr
CPCM(MeCN)/B3LYP-D3/def2-TZVPP//CPCM(MeCN)/B3LYP-D3/def2-SVP
E = -2437.250353
G = -2436.834965
N_{imag} = 0

Ni	0.67315900	-2.47299500	-0.19986400
H	-6.28800800	1.50735000	-1.11681600
H	-5.68716500	-0.15962700	-1.26582500
H	0.94530600	4.49283000	-0.73411100
H	-4.81809100	1.17164500	-2.07063400
H	4.06397700	4.74433500	1.31634200
H	4.57901300	3.04646900	1.19876000
H	-5.10948600	3.20052400	0.26452000
H	-3.62200200	2.96819600	-0.67791700
H	-3.58510600	2.84076700	1.10248200

H	-4.73540700	0.86130300	2.27259600
N	-0.97646300	-1.42977400	-0.15051400
C	-3.25970700	0.24468800	0.01606700
C	-2.22301900	-1.94294100	-0.15516800
C	-0.84624200	-0.07850100	-0.06739300
C	-1.96139500	0.76565600	0.01959700
C	-3.36485100	-1.16025300	-0.07756800
N	1.46515500	-0.60216500	-0.18425100
C	2.29479200	2.09310000	-0.05660100
C	2.76462300	-0.27905500	-0.23105200
C	0.55410000	0.39246900	-0.08474000
C	0.94151200	1.73891800	-0.01501200
C	3.21795000	1.03307100	-0.17183400
H	-2.28739400	-3.03167300	-0.22335400
H	-1.79899800	1.83871800	0.08749800
H	-4.33736500	-1.65447700	-0.08950200
H	3.45965000	-1.11816100	-0.31863400
H	0.17385100	2.50432300	0.06945300
H	4.29161500	1.22032000	-0.21556400
C	2.06645500	-3.88981600	-0.09183100
H	3.15288400	3.47512300	2.17762000
H	-5.63902400	-0.34362600	1.32023400
C	2.78410300	3.54590900	0.01470600
C	1.62097300	4.54553000	0.13379200
C	3.69996000	3.70659400	1.24993500
C	3.58269100	3.87067100	-1.26867200
C	-4.51821000	1.11756600	0.10327900
C	-5.32800000	0.71144000	1.35613500
C	-4.17954700	2.61478200	0.20346900
C	-5.37586800	0.89095800	-1.16310600
H	3.94353500	4.91107200	-1.23427800
H	2.95126600	3.75618700	-2.16401300
H	4.45987000	3.21626000	-1.38414100
H	2.01908000	5.57053700	0.18256100
H	1.02738500	4.37620600	1.04564300
H	-6.23907600	1.32636100	1.43246800
H	2.82086100	-3.71865200	-0.89337500
C	1.52294100	-5.31289000	-0.28961600
C	2.81522300	-3.79521400	1.24562300
H	0.75417800	-5.55893700	0.46689800
H	2.30616600	-6.10214100	-0.20365200
H	1.04273100	-5.44099100	-1.27575000
H	3.25820800	-2.79659200	1.40505200
H	3.64074000	-4.53814300	1.34468000
H	2.13659900	-3.97417700	2.10026200

Int2-^{n}Pr

CPCM(MeCN)/B3LYP-D3/def2-TZVPP//CPCM(MeCN)/B3LYP-D3/def2-SVP

E = -2437.254383

G = -2436.841576

N_{imag} = 0

Ni	-2.09073400	-1.28940300	-0.00032400
H	5.67131600	-3.05282700	1.27868600

H	4.10203800	-3.88874900	1.29747100
H	2.31774800	4.05049300	0.89314900
H	4.29881300	-2.35206300	2.17781100
H	0.33676400	6.36365200	-1.27884900
H	-1.18747600	5.44824200	-1.29565300
H	6.01658100	-0.95386600	-0.00029300
H	4.69973200	-0.16342700	0.89201900
H	4.69971000	-0.16416000	-0.89328200
H	4.29942800	-2.35416800	-2.17724200
N	-0.16782900	-1.61996000	-0.00029100
C	2.65381300	-1.90853600	-0.00010400
C	0.41164300	-2.83725700	-0.00030200
C	0.64526400	-0.52966600	-0.00018400
C	2.04125000	-0.65082200	-0.00009100
C	1.78577300	-3.02209700	-0.00021300
N	-1.42415600	0.63018800	-0.00029300
C	-0.23049500	3.18747100	-0.00003200
C	-2.17507600	1.73951600	-0.00031500
C	-0.07805200	0.75875500	-0.00016800
C	0.53894500	2.01848800	-0.00002400
C	-1.63069800	3.01806100	-0.00020300
H	-0.27322000	-3.68876700	-0.00036300
H	2.64361300	0.25449800	-0.00000800
H	2.17452300	-4.04144500	-0.00019900
H	-3.25678700	1.57987900	-0.00041000
H	1.62480800	2.07325200	0.00010000
H	-2.30409700	3.87614100	-0.00023000
C	-4.07162800	-1.45529200	0.00020000
H	0.23065000	4.82625000	-2.17761000
H	4.10264800	-3.89004600	-1.29548500
C	0.38257300	4.59443100	0.00013200
C	1.92039100	4.55800700	0.00034600
C	-0.09197600	5.34888600	-1.26317500
C	-0.09232500	5.34876500	1.26337000
C	4.17527000	-2.10450900	0.00014400
C	4.58091300	-2.89941900	-1.26239900
C	4.93213400	-0.76529300	-0.00038600
C	4.58048800	-2.89823500	1.26357500
H	0.33635200	6.36355400	1.27922600
H	0.23010500	4.82608300	2.17784800
H	-1.18783900	5.44805900	1.29560600
H	2.31376300	5.58599800	0.00046400
H	2.31800500	4.05060700	-0.89240800
H	5.67176400	-3.05388300	-1.27705400
H	-4.50918500	-0.92218100	0.87591500
C	-4.62588200	-2.89191200	0.00012400
H	-4.50981800	-0.92180200	-0.87496100
H	-4.23242800	-3.44280200	0.87632500
H	-4.23322200	-3.44244900	-0.87665900
C	-6.15701600	-2.99874600	0.00077200
H	-6.58441500	-2.50042600	0.88905000
H	-6.51208400	-4.04538600	0.00077000
H	-6.58519800	-2.50015800	-0.88697800

TS1-Me
CPCM(MeCN)/B3LYP-D3/def2-TZVPP//CPCM(MeCN)/B3LYP-D3/def2-SVP
E = -5392.514677
G = -5392.032335
N_{imag} = 1, 76.6 *i* cm^{-1}

Ni	0.67536400	-1.88799400	1.30551300
C	-0.40617000	-2.44003600	-0.33957000
C	-2.79137300	-1.16868500	-1.15754700
C	-1.66739800	-2.95646100	0.06396900
C	-0.37573000	-1.40948400	-1.31949600
C	-1.54518200	-0.75977400	-1.68132700
C	-2.82724000	-2.29791200	-0.30629800
H	-1.70497100	-3.81256700	0.73668300
H	0.58204800	-1.08006700	-1.72190000
H	-1.50207500	0.08426800	-2.37147300
H	-3.79399200	-2.63570100	0.07347100
N	-0.33088300	-0.07765600	1.53075700
C	-1.76767700	2.31042000	1.16605200
C	-1.59986800	0.01999300	1.93286200
C	0.26174000	0.98868100	0.96163100
C	-0.42576600	2.19372600	0.77657300
C	-2.34728700	1.18193600	1.77187200
N	2.03084100	-0.54491900	0.46656000
C	3.80193100	1.47517100	-0.38634900
C	3.25785300	-0.84961400	0.02539500
C	1.65123200	0.75006800	0.49126400
C	2.51275400	1.77217300	0.07539100
C	4.16168000	0.11471500	-0.40605600
H	-2.03051300	-0.88048200	2.37542500
H	0.08176000	3.02330400	0.29095300
H	-3.38726600	1.18521600	2.09959000
H	3.50835800	-1.91301000	0.00832200
H	2.16829400	2.80195100	0.12562400
H	5.14375100	-0.20704500	-0.75426800
Br	1.15909700	-3.88828500	-0.60641100
C	0.04464600	-3.09216700	2.78778900
C	-4.05558000	-0.48021000	-1.47351700
C	4.79156200	2.55057100	-0.85237200
C	4.19945000	3.96674400	-0.75248300
C	6.05842500	2.48090700	0.03122800
C	5.16966300	2.27626200	-2.32628400
C	-2.60718400	3.56713500	0.90681100
C	-3.19499100	4.07934100	2.24044600
C	-1.78166900	4.69616100	0.26708200
C	-3.75161400	3.18237800	-0.05993600
H	0.65425600	-2.95646500	3.70654700
H	4.94259900	4.70116800	-1.09817300
H	3.93287400	4.22750800	0.28359500
H	3.30264100	4.08105800	-1.38108600
H	0.10512600	-4.16609800	2.52616500
H	-4.76679900	2.04441300	-3.39975800
H	-5.59985100	1.66683600	-1.84937300

H	-5.77905500	0.56189200	-3.24290900
H	-1.00494900	-2.90018300	3.08453900
O	-5.14767900	-0.78131000	-1.02247200
O	-3.89726700	0.55182100	-2.33418100
C	-5.08548700	1.24150500	-2.72393200
H	-2.42233900	5.57720200	0.11044300
H	-1.37780400	4.40310100	-0.71441800
H	-0.94251200	5.00395200	0.91041000
H	-2.39349600	4.35124500	2.94549500
H	-3.83617400	3.32794400	2.72513700
H	-3.81021300	4.97482400	2.05887200
H	-4.36357900	4.06785300	-0.29419300
H	-4.41550400	2.41935800	0.37445400
H	-3.34835100	2.77739800	-0.99995400
H	6.78198400	3.24650900	-0.29081500
H	6.55496000	1.50129100	-0.03761400
H	5.81148500	2.66561100	1.08865000
H	5.88161000	3.03961900	-2.67813300
H	4.27964300	2.31260500	-2.97417100
H	5.64499700	1.29198900	-2.45331500

TS1-Et
CPCM(MeCN)/B3LYP-D3/def2-TZVPP//CPCM(MeCN)/B3LYP-D3/def2-SVP
E = -5431.840763
G = -5431.332229
N_{imag} = 1, 59.2 *i* cm^{-1}

Ni	0.56226900	-1.83306400	1.05702400
C	-0.54828400	-2.45623000	-0.51452800
C	-2.91202700	-1.07108600	-1.22499700
C	-1.82572300	-2.97671900	-0.15270000
C	-0.50527600	-1.36396600	-1.43302100
C	-1.66073200	-0.65980800	-1.73251500
C	-2.96849300	-2.26680700	-0.46445500
H	-1.88220000	-3.88136100	0.45273400
H	0.45518100	-1.03723200	-1.83056600
H	-1.60139300	0.22856900	-2.36339600
H	-3.94141400	-2.61093300	-0.10641600
N	-0.36359000	0.00648000	1.32970900
C	-1.65672300	2.49127900	1.09281300
C	-1.63428400	0.15077100	1.71339500
C	0.29924500	1.07011200	0.83598700
C	-0.31649700	2.32122600	0.71643900
C	-2.31227200	1.36123600	1.61235100
N	1.99301800	-0.52922600	0.28417600
C	3.87487700	1.43447500	-0.45695700
C	3.20617000	-0.87589700	-0.16609100
C	1.68062600	0.78176500	0.36961800
C	2.59916300	1.77586700	0.01080300
C	4.16263600	0.05916900	-0.54406400
H	-2.12328700	-0.74876900	2.09257200
H	0.24659700	3.14991900	0.29487600
H	-3.35680400	1.40381700	1.92278800
H	3.40060800	-1.94919800	-0.23400600

H	2.30870500	2.81877900	0.10819800
H	5.12902800	-0.29611200	-0.90344900
Br	0.95576600	-3.91035100	-0.90686200
C	-0.13152400	-3.00735200	2.51031900
C	-4.15753500	-0.32773700	-1.46722400
C	4.92353300	2.47781300	-0.86312100
C	4.40958200	3.91679800	-0.68627400
C	6.18217400	2.29128800	0.01518100
C	5.29063100	2.26335400	-2.34971300
C	-2.41216900	3.81443800	0.91779800
C	-2.95759800	4.27636500	2.28773800
C	-1.51509400	4.92514900	0.34572200
C	-3.58544500	3.57393600	-0.06007500
C	0.61006000	-2.66019800	3.80519000
H	0.27069800	-3.23345500	4.69385200
H	1.69658300	-2.84059300	3.71255700
H	0.49219100	-1.59042800	4.05770400
H	0.00374100	-4.07780500	2.25949100
H	-4.80402200	2.43722300	-3.05888800
H	-5.66569400	1.87187500	-1.58406400
H	-5.83790100	0.96264800	-3.11280400
H	-1.22042200	-2.85965300	2.64623200
O	-5.26259900	-0.66175900	-1.07155500
O	-3.97056700	0.80493600	-2.18708000
C	-5.14264500	1.55675700	-2.49931600
H	-2.09755800	5.85359800	0.24583600
H	-1.13035000	4.66720700	-0.65322700
H	-0.65820500	5.13902700	1.00355700
H	-2.13588200	4.44923800	3.00069300
H	-3.64286300	3.53723800	2.72919800
H	-3.51422300	5.21973900	2.17082100
H	-4.13420300	4.51367300	-0.23092000
H	-4.29948100	2.83437000	0.33191300
H	-3.21809100	3.20267200	-1.02847200
H	6.94738400	3.03251500	-0.26495500
H	6.62480000	1.29125100	-0.10681400
H	5.94271400	2.43162700	1.08110100
H	6.04520700	3.00365500	-2.65957700
H	4.40612800	2.38421100	-2.99491500
H	5.71060600	1.26225900	-2.52958000
H	5.19368800	4.62719200	-0.98901500
H	4.15204800	4.13397000	0.36211100
H	3.52382400	4.11514300	-1.30957900

TS1-^{i}Pr
CPCM(MeCN)/B3LYP-D3/def2-TZVPP//CPCM(MeCN)/B3LYP-D3/def2-SVP
E = -5471.171284
G = -5470.634664
N_{imag} = 1, 89.3 *i* cm^{-1}

Ni	0.45529300	-1.78355600	0.82985300
C	-0.76482700	-2.35473400	-0.63306300
C	-3.08115400	-0.83768400	-1.22166700
C	-2.05393200	-2.82792400	-0.24727400

C	-0.69921200	-1.23364700	-1.51841800
C	-1.82989900	-0.46891900	-1.75883300
C	-3.17089600	-2.05641900	-0.50081800
H	-2.13598700	-3.74891300	0.33029900
H	0.26212200	-0.93986900	-1.93965300
H	-1.74908900	0.43702900	-2.36171200
H	-4.14681900	-2.36533900	-0.11943800
N	-0.35273100	0.11869300	1.18908900
C	-1.43892900	2.71491100	1.01496900
C	-1.62122600	0.35756700	1.53892200
C	0.40375900	1.14768500	0.75333400
C	-0.10731000	2.44785200	0.66687100
C	-2.19677000	1.62170700	1.46761100
N	1.97258100	-0.54402800	0.12588200
C	4.02940400	1.29652300	-0.44018800
C	3.16286300	-0.96277900	-0.32585000
C	1.76867700	0.77864300	0.29799300
C	2.77691800	1.71331800	0.02850500
C	4.20241200	-0.08949300	-0.62069600
H	-2.20008100	-0.50567100	1.86511100
H	0.53281300	3.24130200	0.29001100
H	-3.24417400	1.73397500	1.74963800
H	3.26785000	-2.04220700	-0.46228500
H	2.57502800	2.76798200	0.19754800
H	5.14344800	-0.50013800	-0.98819700
Br	0.66201900	-3.86851900	-1.17816200
C	-0.03684600	-3.10235700	2.24732000
C	-4.29212400	-0.01976200	-1.38146500
C	5.17239500	2.27080800	-0.75252600
C	4.77874000	3.73339800	-0.48433300
C	6.38903600	1.91641600	0.13331200
C	5.55573500	2.12797900	-2.24365200
C	-2.07638800	4.10351400	0.88493400
C	-2.58365800	4.55841700	2.27214500
C	-1.08341100	5.15183400	0.35534000
C	-3.26447700	4.00544100	-0.09937700
C	1.26446800	-3.23478000	3.04285100
H	1.17852600	-3.94186800	3.89752400
H	2.09961500	-3.58929900	2.41397900
H	1.57243400	-2.26406000	3.47582400
H	-0.26398200	-4.07262600	1.76691300
H	-4.83300500	2.87456800	-2.76936200
H	-5.67762800	2.25595300	-1.30611200
H	-5.95043100	1.46586600	-2.88517700
C	-1.20121200	-2.69725100	3.13725200
H	-1.01868400	-1.71837500	3.61597800
H	-2.14256300	-2.61624500	2.56752100
H	-1.38540100	-3.42095300	3.96004300
H	-1.58371900	6.12948400	0.28343200
H	-0.71348800	4.89427100	-0.64936400
H	-0.21702400	5.27055200	1.02460800
H	-1.75196000	4.63153500	2.99070200
H	-3.33111300	3.86393500	2.68445400

H	-3.05621500	5.55032200	2.19233900
H	-3.72820500	4.99586600	-0.23136100
H	-4.04147700	3.31719000	0.26396500
H	-2.93089600	3.64320900	-1.08368900
H	7.22074100	2.60672000	-0.07921600
H	6.74864900	0.89293700	-0.05125100
H	6.13624800	2.00263300	1.20191300
H	6.37753200	2.81999700	-2.48697900
H	4.70044100	2.36861800	-2.89465600
H	5.89359100	1.10916200	-2.48603600
H	5.62742900	4.39223800	-0.72279500
H	4.51641000	3.89994000	0.57206700
H	3.92790600	4.04985400	-1.10758300
O	-5.40057400	-0.31776700	-0.96668200
O	-4.06609200	1.14293600	-2.03962800
C	-5.20269000	1.97708800	-2.25878800

TS1-*n*Pr

CPCM(MeCN)/B3LYP-D3/def2-TZVPP//CPCM(MeCN)/B3LYP-D3/def2-SVP

E = -5471.173132

G = -5470.637245

$\boldsymbol{N}_{\mathbf{imag}}$ = 1, 62.1 *i* cm^{-1}

Ni	0.58042100	-1.83758500	0.70953200
C	-0.53197700	-2.26594700	-0.92346300
C	-2.90797700	-0.83318000	-1.47092400
C	-1.80209900	-2.83599200	-0.61927700
C	-0.49905600	-1.07712500	-1.71154700
C	-1.66180100	-0.35501200	-1.92988800
C	-2.95239700	-2.10619800	-0.84848500
H	-1.84795300	-3.80264400	-0.11786500
H	0.45840200	-0.69767400	-2.06744100
H	-1.61216600	0.59975900	-2.45588500
H	-3.92141000	-2.49665200	-0.52971400
N	-0.35898600	-0.05131400	1.19888600
C	-1.67931800	2.42922200	1.25443000
C	-1.62824100	0.03369900	1.60391300
C	0.28985800	1.06873600	0.82711000
C	-0.33982200	2.31845600	0.85394700
C	-2.31936100	1.24014300	1.64576700
N	1.99999000	-0.43876200	0.09403000
C	3.85702500	1.61849900	-0.42276800
C	3.21658600	-0.71789700	-0.39208300
C	1.67265500	0.85046300	0.32749500
C	2.57852100	1.88957100	0.08205300
C	4.16130900	0.26518400	-0.66285700
H	-2.10460600	-0.90894300	1.88056700
H	0.21172500	3.19619200	0.52679500
H	-3.36211800	1.23554400	1.96471900
H	3.42403200	-1.77421900	-0.58041100
H	2.27603900	2.91132700	0.29661700
H	5.13110700	-0.03603700	-1.06039800
Br	0.99540900	-3.64890300	-1.46027500
C	-0.09952000	-3.16693200	2.03316300

C	-4.16107100	-0.07823600	-1.62580300
C	4.89113800	2.71380100	-0.71280800
C	4.36007400	4.11767200	-0.37631500
C	6.15539800	2.44740100	0.13632100
C	5.25481900	2.67021100	-2.21504100
C	-2.45110900	3.75433400	1.23424900
C	-2.99719400	4.04955200	2.64924800
C	-1.56958000	4.93418000	0.79111400
C	-3.62505500	3.61230300	0.23786000
C	0.62415900	-2.95511900	3.36688000
C	0.16416800	-3.87583500	4.50698300
H	1.71464800	-3.08532600	3.22398900
H	0.49882200	-1.90238600	3.68720500
H	0.05004200	-4.20809900	1.68141900
H	-4.83868800	2.83077400	-2.91939400
H	-5.67741800	2.11788500	-1.49618600
H	-5.86862300	1.36394500	-3.10510700
H	-1.19187400	-3.05211100	2.18473800
O	-5.26007000	-0.46067300	-1.25873600
O	-3.98768200	1.12392500	-2.22553100
C	-5.16668000	1.89757300	-2.44549100
H	-2.16306700	5.86095900	0.80093900
H	-1.18652300	4.79781500	-0.23216600
H	-0.71213300	5.08071700	1.46641800
H	-2.17506700	4.14934900	3.37558200
H	-3.67228900	3.25653500	3.00444500
H	-3.56526800	4.99331300	2.64248200
H	-4.18508700	4.55883800	0.17546200
H	-4.32931100	2.82478300	0.54528000
H	-3.25697200	3.35719300	-0.76717500
H	6.90988300	3.22530900	-0.06127300
H	6.61073900	1.47322200	-0.09719200
H	5.91803400	2.46511500	1.21176900
H	5.99877100	3.44995700	-2.44300900
H	4.36617100	2.85049800	-2.84038900
H	5.68637100	1.70069800	-2.50614900
H	5.13410100	4.86718600	-0.60056300
H	4.10430500	4.21399800	0.69036200
H	3.46947200	4.37259200	-0.97153300
H	-0.91255400	-3.73952500	4.70919700
H	0.31368000	-4.93627500	4.23909600
H	0.70997000	-3.68627900	5.44852000

Int3-Me
CPCM(MeCN)/B3LYP-D3/def2-TZVPP//CPCM(MeCN)/B3LYP-D3/def2-SVP
E = -5392.569240
G = -5392.084509
N_{imag} = 0

H	-0.31798700	4.27528700	0.20089700
C	1.61184600	0.96835700	0.15967700
N	0.26291300	0.97006200	0.24461000
Ni	-0.58192600	-0.94449100	0.43876400
C	3.23468500	-2.92018800	0.44180300

C	4.15399400	-1.88230900	0.19735900
C	3.62418400	-0.59048200	0.09372800
C	2.24694600	-0.37107800	0.22837000
N	1.39944600	-1.39084600	0.45152500
C	1.88013400	-2.63461700	0.55487900
C	-0.37693700	2.13963500	0.26976300
H	3.56075600	-3.95610000	0.53958400
C	5.65055400	-2.18610600	0.05377200
H	4.27655000	0.25715300	-0.09739500
C	2.33302800	2.15920400	0.05703000
H	1.14532100	-3.42450200	0.72697300
C	1.67696600	3.40162900	0.05539500
C	0.28070000	3.36652300	0.17557400
H	5.31546200	-4.10324300	-0.98912200
H	-1.46222500	2.09530000	0.37380400
H	3.41887300	2.12000400	-0.00890100
C	2.48574200	4.69988800	-0.05930800
C	3.46785300	4.78625800	1.13189700
C	1.58250800	5.94466700	-0.04351600
C	3.27814500	4.67957400	-1.38685900
H	4.17757900	3.94528100	1.14397400
H	2.92507800	4.78944100	2.09026000
H	4.05381400	5.71661500	1.06618100
H	1.01193300	6.02433500	0.89485600
H	0.87091600	5.94427000	-0.88380500
H	2.20232000	6.84969800	-0.13289100
H	3.86453900	5.60663800	-1.48633300
H	2.59807900	4.60920400	-2.25035000
H	3.98045500	3.83360600	-1.43537300
C	6.15301900	-2.85172600	1.35560100
C	6.47926300	-0.91543300	-0.20027600
C	5.85006400	-3.15276700	-1.13644900
H	7.22752500	-3.07784700	1.26888300
H	5.62874300	-3.79629300	1.56476100
H	6.01164900	-2.18322600	2.21948700
H	7.54260100	-1.18301700	-0.29377000
H	6.38981600	-0.19555500	0.62813000
H	6.18145300	-0.41123300	-1.13277000
H	6.92032500	-3.38505100	-1.25424300
H	5.49161100	-2.70066000	-2.07467400
C	-4.75006100	-1.34435300	1.22489800
C	-3.36302400	-1.48684300	1.30080600
C	-2.50274700	-0.72210200	0.49262500
C	-3.09308500	0.18981500	-0.40376300
C	-4.47675500	0.35711900	-0.47258600
C	-5.32344200	-0.41250500	0.34492400
H	-2.95218000	-2.20937100	2.00990800
Br	-0.62342900	-1.68610700	-1.93894900
H	-2.46294900	0.75813200	-1.09032200
H	-4.90848800	1.07551300	-1.17164900
C	-6.80431600	-0.28509300	0.31032300
H	-5.40800500	-1.94869000	1.85369100
O	-7.22811500	0.64306100	-0.56698200

O	-7.57123400	-0.93816300	0.99150300
C	-8.64136300	0.83108800	-0.66960700
H	-8.79292500	1.61373800	-1.42271500
H	-9.06559600	1.14634200	0.29603400
H	-9.13827600	-0.09952700	-0.98402500
C	-0.60181300	-0.86567200	2.40258800
H	0.39471700	-0.52081900	2.71558200
H	-0.76656600	-1.90380800	2.73694000
H	-1.37922200	-0.21071000	2.81524100

Int3-Et

CPCM(MeCN)/B3LYP-D3/def2-TZVPP//CPCM(MeCN)/B3LYP-D3/def2-SVP

E = -5431.893581

G = -5431.383150

N_{imag} = 0

H	-0.37233500	4.40905700	0.08490100
C	1.63756700	1.14638800	-0.05589500
N	0.28983400	1.11791700	-0.02211500
Ni	-0.60566900	-1.00836600	0.26872800
C	3.49434200	-2.66235300	-0.05550500
C	4.34279500	-1.54721800	-0.16909100
C	3.72074100	-0.29322300	-0.18291900
C	2.32726400	-0.17583300	-0.07420700
N	1.54629300	-1.26934900	0.03048800
C	2.11982200	-2.47956800	0.03391800
C	-0.37857600	2.26906900	0.03571700
H	3.88908000	-3.67877500	-0.03834200
C	5.86256600	-1.72790000	-0.26871600
H	4.31986100	0.60708000	-0.28011800
C	2.33230900	2.36036800	-0.05208000
H	1.43393500	-3.32704200	0.09310900
C	1.64754300	3.58643100	-0.00887200
C	0.24879800	3.51614000	0.03995300
H	5.72101800	-3.58515300	-1.45447600
H	-1.46751500	2.19442500	0.08418700
H	3.42012600	2.35678600	-0.07341300
C	2.43089300	4.90574300	-0.00634100
C	3.34842300	4.94144700	1.23772500
C	1.49926500	6.12894300	0.03429800
C	3.29055400	4.98195100	-1.28922000
H	4.07478200	4.11463200	1.23882900
H	2.75711800	4.87808100	2.16481700
H	3.91649700	5.88498400	1.25671500
H	0.87903800	6.14111700	0.94402700
H	0.83228300	6.16407000	-0.84117500
H	2.10149200	7.05016200	0.03079400
H	3.85784500	5.92601700	-1.30378700
H	2.65761900	4.94807900	-2.19000600
H	4.01559200	4.15626100	-1.35019200
C	6.36076800	-2.44734000	1.00612800
C	6.60122200	-0.38464600	-0.39580400
C	6.18440300	-2.58876500	-1.51190100
H	7.45176400	-2.58968900	0.95317200

H	5.89898500	-3.43888900	1.12611800
H	6.13549300	-1.85403300	1.90639100
H	7.68462000	-0.56596900	-0.46262500
H	6.42715100	0.26333300	0.47731800
H	6.30039500	0.16447700	-1.30164100
H	7.27342000	-2.73057000	-1.59676900
H	5.82946100	-2.09891600	-2.43234800
C	-4.77555600	-0.68618500	1.23071500
C	-3.42300000	-1.00225400	1.36445300
C	-2.48124500	-0.57742700	0.41377300
C	-2.94591300	0.17610800	-0.68259500
C	-4.29674200	0.50128400	-0.82263500
C	-5.22801500	0.07155800	0.13750300
H	-3.10946200	-1.59600500	2.22589500
Br	-1.13836600	-3.14805300	-1.04782100
H	-2.24896500	0.50831900	-1.45797900
H	-4.63385200	1.08584400	-1.68021700
C	-6.67915400	0.38309700	0.03953600
H	-5.50213200	-1.02317400	1.97355100
O	-6.98372100	1.10836100	-1.05152500
O	-7.51782200	0.02959400	0.84549500
C	-8.35980400	1.45194600	-1.23081700
H	-8.41276800	2.03041300	-2.16096400
H	-8.72525200	2.05628700	-0.38644400
H	-8.98162400	0.54709300	-1.31027500
C	-0.42748200	-0.87977500	2.24220000
H	0.53573900	-0.35756500	2.35145400
C	-0.39231200	-2.29750200	2.77317500
H	-1.22167700	-0.26929700	2.68991100
H	-0.25999100	-2.30340700	3.87189900
H	0.43796700	-2.87577800	2.33967000
H	-1.32357600	-2.84266500	2.55204200

Int3-^{i}Pr

CPCM(MeCN)/B3LYP-D3/def2-TZVPP//CPCM(MeCN)/B3LYP-D3/def2-SVP

E = -5471.233643

G = -5470.695722

N_{imag} = 0

H	0.39564200	4.47587000	-0.30481300
C	1.84140400	0.94803600	0.05310100
N	0.50475500	1.13087700	-0.03646500
Ni	-0.61942900	-0.64225600	0.11748000
C	2.89680500	-3.14035100	0.14920000
C	3.96653100	-2.22549800	0.15941800
C	3.62713800	-0.86695400	0.13792100
C	2.28417000	-0.46773100	0.11877000
N	1.28984600	-1.37244900	0.12575700
C	1.58763100	-2.67583000	0.12944700
C	0.03825800	2.37243600	-0.17035300
H	3.07026000	-4.21670100	0.15441800
C	5.41683300	-2.72466700	0.18492000
H	4.40455800	-0.10797300	0.12825000
C	2.72343700	2.03014800	0.04614700

H	0.74030300	-3.36500200	0.10612400
C	2.24981900	3.34749700	-0.07109800
C	0.86161500	3.49847800	-0.19317300
H	5.00417800	-4.47129400	-1.10169300
H	-1.04237700	2.47474000	-0.27227400
H	3.79268300	1.84497600	0.13032600
C	3.23410900	4.52384500	-0.06711200
C	4.01600700	4.51603600	1.26689200
C	2.51571200	5.87636000	-0.20866900
C	4.21818200	4.35987500	-1.24834000
H	4.59169700	3.58792100	1.40257400
H	3.33295200	4.62286300	2.12428500
H	4.72752100	5.35661100	1.28797000
H	1.81446800	6.05569500	0.62113500
H	1.95841500	5.94645600	-1.15572200
H	3.25736400	6.68942700	-0.19863300
H	4.93075700	5.19968400	-1.26034100
H	3.68120200	4.35144200	-2.20990900
H	4.80089800	3.42925300	-1.17435400
C	5.62892900	-3.57420700	1.45931300
C	6.42933700	-1.56678200	0.19139500
C	5.66823700	-3.59446100	-1.06848000
H	6.66621000	-3.94312600	1.49447200
H	4.96204500	-4.44910800	1.48706700
H	5.44661100	-2.97598700	2.36600700
H	7.45188300	-1.97289100	0.21354700
H	6.30908600	-0.92247000	1.07617500
H	6.34122900	-0.94016200	-0.70974000
H	6.70682200	-3.96142300	-1.06675300
H	5.51177400	-3.01150900	-1.98983000
C	-4.68537700	-1.52479200	-0.01178500
C	-3.29325400	-1.55948100	0.03517500
C	-2.52993800	-0.38482800	0.20063800
C	-3.22887400	0.82710300	0.31521600
C	-4.62400300	0.87922500	0.25507100
C	-5.36964500	-0.30009600	0.09234300
H	-2.78795700	-2.52420400	-0.07062400
Br	-0.70295700	-1.05119400	-2.35957000
H	-2.68962700	1.76109300	0.48203600
H	-5.14113500	1.83633300	0.34243300
C	-6.85379200	-0.30771800	0.02887300
H	-5.26430300	-2.44267700	-0.13732000
O	-7.38892600	0.92328700	0.13385600
O	-7.53650500	-1.30558000	-0.10384500
C	-8.81455700	1.00837100	0.08142100
H	-9.06270700	2.07184600	0.18263500
H	-9.27192500	0.43224900	0.90044500
H	-9.19528700	0.61928200	-0.87546200
C	-0.62329000	-0.62709600	2.13090000
H	0.46587200	-0.52786800	2.26303300
C	-1.07655100	-1.98934900	2.62464600
C	-1.29615700	0.53179100	2.84050700
H	-0.84916600	-2.10499400	3.70202400

H	-0.57165800	-2.81510600	2.09778800
H	-2.16218200	-2.12224800	2.49840700
H	-2.39199700	0.44280200	2.84119100
H	-1.03426300	1.49965400	2.38591800
H	-0.96340800	0.56471000	3.89575000

Int3-^{n}Pr

CPCM(MeCN)/B3LYP-D3/def2-TZVPP//CPCM(MeCN)/B3LYP-D3/def2-SVP

E = -5471.225852

G = -5470.689321

N_{imag} = 0

H	-0.35822900	4.46708800	0.14449400
C	1.64621200	1.21017500	-0.13337800
N	0.29794500	1.18443800	-0.13133500
Ni	-0.60123800	-0.94913200	0.09817300
C	3.49789300	-2.59685000	-0.29946300
C	4.34664600	-1.47892200	-0.37761800
C	3.72581000	-0.22485900	-0.33810700
C	2.33344300	-0.11086900	-0.21299200
N	1.55162200	-1.20689300	-0.14799900
C	2.12404900	-2.41678800	-0.19452300
C	-0.36852800	2.33335000	-0.02489200
H	3.89165700	-3.61351500	-0.32350200
C	5.86536900	-1.65668900	-0.49685500
H	4.32442500	0.67832200	-0.40854600
C	2.34388900	2.41893400	-0.04033100
H	1.43765700	-3.26534600	-0.16328000
C	1.66142500	3.64315200	0.05720600
C	0.26169900	3.57576500	0.06458700
H	5.71251200	-3.47039500	-1.74708500
H	-1.45846800	2.26049800	-0.00500400
H	3.43187600	2.41146300	-0.02978400
C	2.44777900	4.95657900	0.16072300
C	3.33130100	4.91265900	1.42896500
C	1.51836700	6.17878100	0.25070300
C	3.34224400	5.10702300	-1.09140300
H	4.05588200	4.08480800	1.39886500
H	2.71500000	4.79460200	2.33416500
H	3.90041500	5.85123200	1.52115700
H	0.87362600	6.13790800	1.14235600
H	0.87545300	6.26968800	-0.63862300
H	2.12250400	7.09616800	0.31952400
H	3.91253700	6.04757800	-1.03284800
H	2.73396600	5.13124800	-2.00932100
H	4.06616300	4.28319000	-1.18328000
C	6.37348700	-2.42113600	0.74755100
C	6.60409800	-0.31016500	-0.58247200
C	6.17623500	-2.47283300	-1.77252000
H	7.46391500	-2.56203900	0.68072300
H	5.91189600	-3.41607700	0.83610300
H	6.15592100	-1.86000800	1.67007000
H	7.68667300	-0.48967400	-0.66563200
H	6.43861800	0.30635400	0.31475000

H	6.29558700	0.27115800	-1.46537100
H	7.26439600	-2.61208600	-1.87138900
H	5.81417100	-1.95012300	-2.67188600
C	-4.76810400	-0.63515600	1.07184300
C	-3.41675100	-0.95940700	1.19700400
C	-2.47440500	-0.51454200	0.25623100
C	-2.93630300	0.26766100	-0.82094800
C	-4.28614800	0.60091700	-0.95199700
C	-5.21834200	0.15097700	-0.00212500
H	-3.10440800	-1.57575600	2.04291900
Br	-1.14755400	-3.07088900	-1.24048000
H	-2.23851900	0.61694300	-1.58812800
H	-4.62162200	1.20776600	-1.79459600
C	-6.66840500	0.47014200	-0.09141600
H	-5.49552300	-0.98787300	1.80648300
O	-6.97088000	1.22360500	-1.16374500
O	-7.50788200	0.09959500	0.70598800
C	-8.34584000	1.57631900	-1.33366900
H	-8.39677900	2.18053700	-2.24741100
H	-8.70970600	2.15797900	-0.47285000
H	-8.97039300	0.67592500	-1.43842900
C	-0.42495300	-0.87942500	2.07124900
H	0.54058600	-0.36510600	2.20482500
C	-0.39528900	-2.31176900	2.57126900
H	-1.21660600	-0.28160800	2.54239000
C	-0.24312000	-2.39740600	4.09714000
H	0.43298200	-2.85708700	2.08909000
H	-1.31600400	-2.83609700	2.26314200
H	0.68544200	-1.90578700	4.43152000
H	-1.08577700	-1.90088300	4.60605400
H	-0.21220200	-3.44630300	4.43511500

Int4
CPCM(MeCN)/B3LYP-D3/def2-TZVPP//CPCM(MeCN)/B3LYP-D3/def2-SVP
E = -5352.670995
G = -5352.220650
N_{imag} = 0

Ni	-0.34426600	-1.15745200	0.05980600
C	-2.20146200	-0.84838200	0.09307200
C	-4.97513400	-0.23592700	0.13874800
C	-2.90193600	-0.72654400	1.30978000
C	-2.93100000	-0.67313600	-1.09933400
C	-4.29318900	-0.36828800	-1.08488100
C	-4.26247400	-0.42117700	1.33726200
H	-2.37025100	-0.86707700	2.25564600
H	-2.42150500	-0.76897500	-2.06293300
H	-4.83770600	-0.22974500	-2.02076300
H	-4.79597600	-0.32129400	2.28555400
N	0.10214000	0.74251400	0.05969000
C	1.00719000	3.43079300	0.04501600
C	-0.76201400	1.77330800	0.09573800
C	1.42814500	1.03183600	0.01906300
C	1.89547100	2.34976000	0.00958400

C	-0.35810100	3.10184500	0.09062800
N	1.67399000	-1.33211000	0.01042700
C	4.49627100	-1.22923200	-0.08150100
C	2.40507300	-2.45536700	-0.01229300
C	2.31992100	-0.14606600	-0.01090500
C	3.71604700	-0.06637600	-0.05618000
C	3.79452300	-2.44724300	-0.05738700
H	-1.81831100	1.51400000	0.12961100
H	2.96754700	2.52166100	-0.02525500
H	-1.12853100	3.87296700	0.12235900
H	1.83653000	-3.38714700	0.00618600
H	4.18802000	0.91199500	-0.07176300
H	4.31808600	-3.40375500	-0.07396700
Br	-0.81488300	-3.47358100	0.06768600
H	6.25271100	-1.43150200	2.04018900
C	-6.42112700	0.09287600	0.21771500
C	6.02866300	-1.20814300	-0.13268000
C	6.58885500	0.22422400	-0.15643500
C	6.57925900	-1.93452700	1.11632500
C	6.49529200	-1.94319500	-1.41047600
C	1.46375200	4.89368900	0.03683600
C	0.95469000	5.58129900	1.32480500
C	2.99542500	5.02291100	-0.01901900
C	0.86155300	5.59666600	-1.20171200
H	7.59550600	-1.93902200	-1.46330100
H	6.10661000	-1.44739800	-2.31397100
H	6.16363100	-2.99228600	-1.42672200
H	7.68813100	0.18859800	-0.19302200
H	6.30548300	0.79011800	0.74464300
H	-8.66743500	0.64734900	-2.06576800
H	-8.57452500	1.51359700	-0.48842800
H	-8.97337300	-0.22833600	-0.52101100
H	6.24570300	0.78375900	-1.04055500
O	-7.04880200	0.21914500	1.25223700
O	-6.99133600	0.24364400	-0.99317300
C	-8.38401200	0.56279100	-1.00965000
H	3.27432000	6.08740300	-0.02395400
H	3.41215700	4.56667700	-0.93046300
H	3.47819900	4.55797700	0.85461400
H	1.36493200	5.09074200	2.22154400
H	-0.14319100	5.56104000	1.39588000
H	1.27180400	6.63607100	1.33518000
H	1.17688100	6.65182200	-1.22215000
H	-0.23859000	5.57634000	-1.19261600
H	1.20515500	5.11769700	-2.13208900
H	7.68055700	-1.93199200	1.09512200
H	6.24790400	-2.98282600	1.16257000

Int5-Me

CPCM(MeCN)/B3LYP-D3/def2-TZVPP//CPCM(MeCN)/B3LYP-D3/def2-SVP

E = -5392.572059

G = -5392.088310

N_{imag} = 0

C	-0.85762700	-2.94798700	-1.90903800
H	-1.86048600	-3.05693500	-2.34221700
H	-0.09620600	-3.24469700	-2.65234900
H	-7.13779400	-1.13724500	3.81428200
H	-7.79150300	0.19491500	2.79178300
H	5.89944700	-3.24628600	0.61743800
H	6.35520900	-1.56911300	0.22164700
C	1.92171100	-0.30711300	-0.00616700
C	3.05737800	-0.37171900	0.80994200
C	3.71358400	-1.59017600	1.02655100
C	3.16896400	-2.71581600	0.38255300
C	2.03426400	-2.58209300	-0.40963700
C	1.18530800	0.95156400	-0.28495700
C	1.54162600	2.19480100	0.25225000
C	0.79611400	3.34195100	-0.04801500
C	-0.30334000	3.16664400	-0.90846000
C	-0.60085300	1.90515700	-1.40942500
H	3.42532200	0.53873000	1.27454900
H	3.61629700	-3.70432900	0.49081200
H	1.59576500	-3.44755800	-0.90939300
H	2.40476000	2.25793800	0.90895700
H	-0.93843900	4.00516100	-1.19607900
H	-1.44033800	1.73733600	-2.08690700
N	1.41927700	-1.40891700	-0.60093400
N	0.12567200	0.82352800	-1.10686000
Br	-1.00036800	-0.44463200	-3.99006000
Ni	-0.36981300	-1.08131500	-1.64811100
C	-1.70631800	-1.18306100	-0.28795200
C	-1.42248600	-1.51211600	1.04535500
C	-2.99460600	-0.73867800	-0.62250200
C	-2.40110700	-1.37589400	2.03083800
H	-0.42842200	-1.87285200	1.32090300
C	-3.97830900	-0.60020400	0.35930700
H	-3.22569700	-0.49398600	-1.66328400
H	-2.18337000	-1.62210200	3.07255300
H	-4.97615500	-0.24739900	0.09315800
C	-3.68754200	-0.91605600	1.69863000
C	-4.69347200	-0.78561400	2.78651500
C	4.95992200	-1.72240300	1.91013800
C	6.12615900	-2.25962500	1.04861900
C	5.38133200	-0.37706200	2.52540600
C	4.65655700	-2.71629800	3.05507300
C	1.13330900	4.72816700	0.51484200
C	1.42468900	5.68683800	-0.66269800
C	-0.07911000	5.24712300	1.32197000
C	2.36216700	4.69680900	1.43955200
H	-6.60476500	0.54480300	4.10205400
H	7.03060800	-2.36468800	1.66843600
H	-0.76847800	-3.55811600	-0.99895000
O	-4.48657900	-1.05160900	3.95476900
O	-5.88197400	-0.33401100	2.34741100
C	-6.90998800	-0.17619900	3.32824100
H	2.28163900	5.33164300	-1.25654700

H	1.66648300	6.68993100	-0.27715600
H	0.56016000	5.78637200	-1.33605700
H	-0.98094600	5.33900000	0.69826600
H	0.14535600	6.24348100	1.73440800
H	-0.31096600	4.57251700	2.16122800
H	2.20307400	4.04195700	2.31041400
H	2.56213000	5.71023600	1.81908900
H	3.26577700	4.35741100	0.90968100
H	5.63413800	0.36601600	1.75316400
H	6.27583600	-0.52313000	3.14948500
H	4.59330500	0.04419200	3.16889800
H	4.39700300	-3.71598100	2.67556000
H	3.82033200	-2.35808300	3.67604900
H	5.54238100	-2.82315700	3.70086000

Int5-Et
CPCM(MeCN)/B3LYP-D3/def2-TZVPP//CPCM(MeCN)/B3LYP-D3/def2-SVP
E = -5431.902354
G = -5431.390911
N_{imag} = 0

C	-1.04645200	-2.96272200	-1.61783000
H	-2.11759500	-3.01425900	-1.85769000
C	-0.19117900	-3.58054800	-2.71793700
H	-6.96354000	-0.47493200	4.27122000
H	-7.61556000	0.81885900	3.19953100
H	-0.50065400	-3.22136000	-3.70834400
H	6.25983600	-1.89982100	0.37251800
C	1.93815200	-0.31613400	0.00447500
C	3.07961800	-0.36764600	0.81535500
C	3.64039300	-1.59469400	1.18864200
C	2.99206400	-2.74525200	0.70648000
C	1.86390800	-2.62176400	-0.09547700
C	1.28483600	0.95839100	-0.38806900
C	1.75939000	2.21826700	0.00035200
C	1.07701100	3.38409500	-0.36809700
C	-0.08927600	3.20824000	-1.13434800
C	-0.50049600	1.93038800	-1.49325900
H	3.52354000	0.56195800	1.15950700
H	3.35217000	-3.74552500	0.94866600
H	1.35288900	-3.51109700	-0.46489700
H	2.66470300	2.28094900	0.59711500
H	-0.68808800	4.05932900	-1.46017100
H	-1.39282300	1.76268300	-2.09884800
N	1.34350200	-1.43945300	-0.44943200
N	0.17258700	0.82984300	-1.13654400
Br	-1.44865900	-0.48681200	-3.76765400
Ni	-0.46915900	-1.09206900	-1.50694600
C	-1.75506200	-1.01031000	-0.08489300
C	-1.42198200	-1.27292300	1.25288700
C	-3.03883300	-0.52392200	-0.37877100
C	-2.34104200	-1.03401300	2.27576100
H	-0.43386500	-1.66499500	1.50547300
C	-3.96402300	-0.28289400	0.63944500

H	-3.31162100	-0.32854900	-1.41923800
H	-2.08064300	-1.23095700	3.31824100
H	-4.95754500	0.10069000	0.40059600
C	-3.62106600	-0.53391200	1.98016900
C	-4.56349900	-0.29347100	3.10505100
C	4.88583100	-1.71179200	2.07501700
C	5.98948900	-2.45328600	1.28559900
C	5.42522700	-0.33643800	2.50309500
C	4.52178200	-2.51745500	3.34350600
C	1.54752400	4.78891200	0.02829300
C	1.81764600	5.60447800	-1.25724600
C	0.43500700	5.47375900	0.85531000
C	2.83508000	4.75465000	0.86919800
H	-6.35026600	1.19712800	4.42564600
H	-0.27972500	-4.68357100	-2.70248200
H	-0.88579000	-3.44694200	-0.64203300
O	-4.30873600	-0.49681500	4.27656800
O	-5.75389900	0.18254800	2.69689200
C	-6.72300300	0.44385700	3.71467200
H	2.60311900	5.13012100	-1.86652800
H	2.15458900	6.61924900	-0.99259200
H	0.91535200	5.70287300	-1.87940500
H	-0.50067700	5.57343500	0.28483900
H	0.75680000	6.48534100	1.14937700
H	0.21786200	4.90277300	1.77183800
H	2.69443000	4.20271000	1.81156900
H	3.13239700	5.78208300	1.12808600
H	3.67177700	4.29607400	0.31952400
H	5.72396300	0.27454800	1.63713200
H	6.31541000	-0.47174700	3.13572500
H	4.68463600	0.22961200	3.08926100
H	4.17458600	-3.53315000	3.10144600
H	3.72855200	-2.01207700	3.91667300
H	5.40676300	-2.61231200	3.99233900
H	5.67551200	-3.46605100	0.99115600
H	6.89286600	-2.55023300	1.90829700
H	0.88234400	-3.35022200	-2.61127800

Int5-^{i}Pr
CPCM(MeCN)/B3LYP-D3/def2-TZVPP//CPCM(MeCN)/B3LYP-D3/def2-SVP
E = -5471.230583
G = -5470.693499
$\boldsymbol{N}_{\mathbf{imag}}$ = 0

C	-0.98877000	-3.32628100	-0.79914300
H	-1.97448500	-3.33642900	-1.28765100
C	0.00489100	-4.12093100	-1.64410000
H	-6.98135100	0.64688400	4.17009800
H	-7.76061300	1.36144100	2.71076600
H	-0.02541700	-3.81861400	-2.69889700
H	1.04581500	-4.02784300	-1.29887800
C	2.01589900	-0.15308000	0.07734000
C	3.20297700	0.08016500	0.78431800
C	3.88824700	-0.96814600	1.40859400

C	3.31344000	-2.24378900	1.27915000
C	2.13375700	-2.40332700	0.56206500
C	1.25354700	0.93712500	-0.58154800
C	1.61414300	2.28898700	-0.49356400
C	0.83495000	3.27779000	-1.10553700
C	-0.30514200	2.83245500	-1.79859300
C	-0.60140000	1.47627600	-1.85182400
H	3.59003300	1.09316400	0.84174600
H	3.77123700	-3.12477100	1.73001500
H	1.68763600	-3.39160400	0.46341600
H	2.50350600	2.56424700	0.06555000
H	-0.97448400	3.53239800	-2.29993200
H	-1.47492300	1.09997200	-2.38634200
N	1.48711900	-1.39161500	-0.03237800
N	0.16255300	0.54690700	-1.26453600
Br	-1.28270400	-1.43649200	-3.51904900
Ni	-0.38031500	-1.45014000	-1.10954700
C	-1.70636600	-0.96774800	0.18445500
C	-1.35814400	-0.65963600	1.50832300
C	-3.02630000	-0.75290100	-0.23782700
C	-2.30687000	-0.14326700	2.39071500
H	-0.33687700	-0.82782900	1.85931700
C	-3.98299400	-0.23795900	0.64079600
H	-3.30330500	-0.98700100	-1.26927100
H	-2.03881300	0.10022200	3.42137600
H	-5.00814700	-0.07381800	0.30404200
C	-3.63019900	0.07256000	1.96617000
C	-4.60446500	0.62320700	2.94510000
C	5.19004900	-0.76894000	2.19306000
C	6.30448800	-1.61009700	1.52876000
C	5.63315200	0.70382200	2.21864800
C	4.97341700	-1.24605200	3.64774000
C	1.17215000	4.77210300	-1.03399700
C	1.35785400	5.31222300	-2.47066100
C	0.00224800	5.51325700	-0.34623400
C	2.46049100	5.03987400	-0.23749900
H	-6.55657000	2.30706600	3.66133000
H	-0.24448400	-5.19877500	-1.59269700
C	-1.11314600	-3.83946900	0.62804700
H	-0.23820500	-3.59590200	1.25021900
H	-1.99251400	-3.42204900	1.13623700
H	-1.21200600	-4.94149200	0.62850300
H	2.18230600	4.79191800	-2.98328900
H	1.59780600	6.38679800	-2.43819800
H	0.44770100	5.19106000	-3.07706600
H	-0.94121800	5.39719400	-0.90057300
H	0.22532400	6.59027800	-0.28621800
H	-0.15405800	5.13752700	0.67729100
H	2.37590400	4.69860600	0.80595700
H	2.66165800	6.12162200	-0.21659500
H	3.33384900	4.54864900	-0.69409400
H	5.82655800	1.09151900	1.20637000
H	6.56758500	0.79600800	2.79247700

H	4.88264000	1.34936600	2.70087200
H	4.70071000	-2.31098300	3.69628300
H	4.17565900	-0.66573000	4.13760000
H	5.90068900	-1.11134300	4.22669300
H	6.06236800	-2.68348500	1.52316700
H	7.24855600	-1.48221100	2.08159800
H	6.47064400	-1.29125600	0.48766800
O	-4.34089000	0.90800800	4.09757900
O	-5.83557900	0.78136700	2.42572700
C	-6.83743900	1.30582400	3.30009300

Int5-^{n}Pr
CPCM(MeCN)/B3LYP-D3/def2-TZVPP//CPCM(MeCN)/B3LYP-D3/def2-SVP
E = -5471.234593
G = -5470.697373
N_{imag} = 0

C	-1.03428800	-3.12378700	-0.76013000
H	-2.10462900	-3.24731600	-0.98057700
C	-0.17597200	-4.02307900	-1.64750100
H	-7.01906000	0.79007900	4.21225300
H	-7.63700600	1.78940000	2.84601400
H	-0.50157700	-3.91402700	-2.69205400
H	6.27507600	-1.61948000	0.83754500
C	1.94623800	-0.14495000	0.12985900
C	3.10014700	0.01202100	0.90877900
C	3.66951800	-1.07907800	1.57590000
C	3.01645400	-2.31405200	1.41653600
C	1.87639500	-2.39938300	0.62644700
C	1.28391800	0.98512600	-0.56999100
C	1.74825500	2.30575600	-0.51076700
C	1.05804600	3.33621500	-1.16073100
C	-0.10433300	2.96425500	-1.86002600
C	-0.50522700	1.63411800	-1.88610900
H	3.54855900	0.99756700	0.99383500
H	3.38261000	-3.21894300	1.90229100
H	1.36208300	-3.35298800	0.50443200
H	2.65112800	2.52350000	0.05233800
H	-0.70831800	3.70128300	-2.39032900
H	-1.39433100	1.31253900	-2.43124700
N	1.34885400	-1.34654100	-0.01123400
N	0.17406800	0.66424400	-1.26228000
Br	-1.37215300	-1.29471900	-3.49643000
Ni	-0.45997900	-1.28918900	-1.13255500
C	-1.75951400	-0.84932500	0.20718200
C	-1.44541900	-0.77371800	1.57267700
C	-3.03421100	-0.44345400	-0.21817500
C	-2.37499100	-0.28180000	2.49016600
H	-0.46427200	-1.09779300	1.92805300
C	-3.96948700	0.05017700	0.69431200
H	-3.29225400	-0.51235900	-1.27840600
H	-2.13060000	-0.21537200	3.55283900
H	-4.95591300	0.36917800	0.35312000
C	-3.64578900	0.13810000	2.06019700

C	-4.59944400	0.65746000	3.07615600
C	4.92828500	-0.96578600	2.44384800
C	6.01600200	-1.90288000	1.86988300
C	5.48265400	0.46863700	2.47877400
C	4.57768400	-1.39628800	3.88699400
C	1.51538000	4.79969000	-1.12750700
C	1.77695100	5.27319000	-2.57598700
C	0.39629000	5.65756600	-0.49320500
C	2.80359400	4.98772300	-0.30819900
H	-6.38228500	2.44177100	3.96300000
C	-0.25118100	-5.49630500	-1.22055400
H	-0.87793200	-3.33873200	0.30976300
O	-4.36152600	0.74839300	4.26517300
O	-5.77881900	1.02783800	2.54508300
C	-6.75710000	1.54174500	3.45187600
H	2.56730900	4.67035800	-3.05050700
H	2.10332100	6.32523000	-2.57243000
H	0.87368900	5.20509400	-3.20072500
H	-0.54068800	5.60258000	-1.06755600
H	0.70756900	6.71368900	-0.46098500
H	0.18567800	5.33024000	0.53719000
H	2.66894200	4.68660300	0.74234800
H	3.09103300	6.04999100	-0.31394100
H	3.64436600	4.41460400	-0.72906700
H	5.76877200	0.82200300	1.47585700
H	6.38342300	0.49877400	3.11017500
H	4.75633800	1.17880700	2.90376600
H	4.21886900	-2.43558400	3.93143300
H	3.79709000	-0.74626000	4.31277300
H	5.47173400	-1.32295100	4.52614400
H	5.69337700	-2.95490100	1.86499900
H	6.92829100	-1.83550600	2.48338000
H	0.88257700	-3.70523200	-1.63521800
H	-1.29185300	-5.86032900	-1.24121000
H	0.12738600	-5.63674600	-0.19381200
H	0.34543600	-6.13744300	-1.89056300

TS2-Me
CPCM(MeCN)/B3LYP-D3/def2-TZVPP//CPCM(MeCN)/B3LYP-D3/def2-SVP
E = -5392.566447
G = -5392.082170
$\boldsymbol{N}_{\mathbf{imag}}$ = 1, 327.0 *i* cm^{-1}

H	-1.25056300	3.31222200	-2.37849300
C	1.10580600	0.83024200	-0.65545400
N	0.03712300	0.38870800	-1.35308100
Ni	-0.37143400	-1.60933100	-1.19056700
C	3.35127900	-2.32798100	1.02868900
C	3.85524100	-1.03030400	1.23243800
C	3.11405100	0.01948800	0.67532600
C	1.93453200	-0.23638100	-0.03484100
N	1.47211800	-1.49233600	-0.20111400
C	2.17144700	-2.50979000	0.31635800
C	-0.76601200	1.27562100	-1.94239600

H	3.86533400	-3.20545100	1.42222300
C	5.14822700	-0.81087300	2.02752000
H	3.44950900	1.04678200	0.78758700
C	1.38089500	2.19418900	-0.54359100
H	1.76277400	-3.51007200	0.15727300
C	0.54853400	3.14847100	-1.15377600
C	-0.54978100	2.65172400	-1.87082900
H	6.11161700	-2.64653200	1.26725700
H	-1.60723000	0.85322400	-2.49735900
H	2.24806000	2.51929000	0.02847300
C	0.86014700	4.64392700	-1.01098200
C	0.82235100	5.02049800	0.48837600
C	-0.15621200	5.51906900	-1.76385800
C	2.26993700	4.92200500	-1.58156300
H	1.56596400	4.45845600	1.07353000
H	-0.17185100	4.82240000	0.91902500
H	1.04197500	6.09296700	0.61069500
H	-1.17870100	5.38187600	-1.37908200
H	-0.15983400	5.30415300	-2.84382000
H	0.10659000	6.58026000	-1.63681900
H	2.50624900	5.99385000	-1.48874300
H	2.32407900	4.64972300	-2.64742400
H	3.05063300	4.36119500	-1.04567700
C	4.95384700	-1.36981000	3.45611800
C	5.52256500	0.67764200	2.12857000
C	6.30153700	-1.56424800	1.32567500
H	5.87381200	-1.22216800	4.04358500
H	4.72996300	-2.44716700	3.45008800
H	4.13017700	-0.85206600	3.97255900
H	6.45255300	0.78391000	2.70723900
H	4.74380300	1.26231500	2.64254200
H	5.69599900	1.12485000	1.13738400
H	7.23853000	-1.41860800	1.88613900
H	6.45214300	-1.18730300	0.30177400
C	-2.13998600	-0.70210900	2.43167900
C	-1.26100700	-1.31389900	1.54311900
C	-1.65197100	-1.58467700	0.21617200
C	-2.97138900	-1.27566500	-0.17179100
C	-3.85280800	-0.66322800	0.71700400
C	-3.44578700	-0.36364800	2.03036400
H	-0.25290800	-1.57293800	1.87340800
Br	-1.35790000	-1.89748600	-3.49691500
H	-3.28987200	-1.50357900	-1.19153800
H	-4.86493600	-0.41154500	0.39610300
C	-4.34151000	0.29175300	3.01618300
H	-1.82769100	-0.47354000	3.45299200
O	-5.56203000	0.56061200	2.51602000
O	-4.02927700	0.56770500	4.15913700
C	-6.49041200	1.19423500	3.39877000
H	-7.41575300	1.32920000	2.82584300
H	-6.10694900	2.16957100	3.73578900
H	-6.67961800	0.56730500	4.28364200
C	-0.97389100	-3.41300800	-0.49513900

H	-0.69650100	-3.76079400	0.50629400
H	-0.23126900	-3.78273100	-1.23026700
H	-1.96142700	-3.78637300	-0.78507300

TS2-Et
CPCM(MeCN)/B3LYP-D3/def2-TZVPP//CPCM(MeCN)/B3LYP-D3/def2-SVP
E = -5431.890166
G = -5431.378191
N_{imag} = 1, 288.2 *i* cm^{-1}

H	-1.68168100	2.74242100	-2.69089100
C	1.01545600	0.81849500	-0.76775100
N	-0.03839500	0.16678000	-1.30194900
Ni	-0.26442000	-1.80106000	-0.73871500
C	3.64139900	-1.73051300	1.34188400
C	4.05314300	-0.39366100	1.19781000
C	3.18296300	0.44831400	0.49531800
C	1.97681700	-0.03817300	-0.02579800
N	1.61024400	-1.32857200	0.12610000
C	2.43209600	-2.14622800	0.79804100
C	-0.96248900	0.86394100	-1.96505300
H	4.25213100	-2.46015000	1.87448500
C	5.38627200	0.08193100	1.78752900
H	3.43845000	1.49302600	0.34177000
C	1.15564200	2.20127400	-0.90413600
H	2.10767600	-3.18197100	0.90892900
C	0.19607000	2.95688100	-1.59903900
C	-0.88626500	2.24594800	-2.13763400
H	6.43055200	-1.81332600	1.34980300
H	-1.78806100	0.27825600	-2.37611700
H	2.01366700	2.69893300	-0.45603000
C	0.36121300	4.47670100	-1.72908200
C	0.38487800	5.10029500	-0.31430000
C	-0.78729800	5.11505400	-2.52842100
C	1.69408300	4.77781600	-2.45248800
H	1.22044500	4.71762300	0.29118100
H	-0.55224300	4.88900400	0.22457200
H	0.49924000	6.19336000	-0.38864000
H	-1.76249800	4.95301100	-2.04351800
H	-0.84017200	4.71975900	-3.55478600
H	-0.62755900	6.20177900	-2.59828400
H	1.82664200	5.86648100	-2.55545300
H	1.70374700	4.33413100	-3.46057300
H	2.56154600	4.38704000	-1.89923400
C	5.36745800	-0.15163000	3.31583600
C	5.63901900	1.57559700	1.52118100
C	6.53302500	-0.73609000	1.14994200
H	6.31951200	0.18367400	3.75677200
H	5.23801700	-1.21472800	3.56866300
H	4.55100200	0.41504300	3.79067900
H	6.60276000	1.86982400	1.96351800
H	4.85969900	2.20953100	1.97205700
H	5.68736900	1.79764300	0.44374200
H	7.50028100	-0.40918900	1.56326600

H	6.55988200	-0.59172500	0.05834100
C	-2.05317100	-0.09412900	2.50584100
C	-1.15153900	-0.91032500	1.83061900
C	-1.53536200	-1.59586300	0.65801400
C	-2.87773200	-1.48920900	0.22999900
C	-3.77930100	-0.67272500	0.90596500
C	-3.37769200	0.04327600	2.05095300
H	-0.12648500	-1.00036600	2.19627000
Br	-1.34518200	-2.51156000	-2.93012600
H	-3.19314400	-2.03638400	-0.66041200
H	-4.80630300	-0.58219400	0.54832900
C	-4.29491400	0.93482500	2.80104000
H	-1.74283300	0.45716000	3.39622100
O	-5.52954000	0.98700200	2.26479500
O	-3.98982200	1.56551500	3.79614400
C	-6.47853900	1.82722600	2.92439400
H	-7.41190900	1.74279500	2.35474400
H	-6.13458700	2.87298000	2.93618700
H	-6.63703700	1.49933400	3.96330100
C	-0.70582900	-3.41663900	0.55586100
H	-0.15878200	-3.31605700	1.50252200
C	0.01064700	-4.41848600	-0.37035800
H	-1.69666100	-3.82311600	0.78513100
H	0.18377700	-5.34894600	0.20209300
H	0.99633700	-4.08354200	-0.73224400
H	-0.59599900	-4.65786100	-1.25155600

TS2-^{i}Pr
CPCM(MeCN)/B3LYP-D3/def2-TZVPP//CPCM(MeCN)/B3LYP-D3/def2-SVP
E = -5471.220443
G = -5470.681448
N_{imag} = 1, 271.1 *i* cm^{-1}

H	-1.23598000	3.07316000	-2.80657200
C	1.13759500	0.75495100	-0.89137100
N	-0.00665400	0.27336500	-1.42117000
Ni	-0.52049000	-1.64420800	-0.88220400
C	3.34539800	-2.14777600	1.24083600
C	3.92363900	-0.86940000	1.14707200
C	3.19429400	0.08577000	0.43028100
C	1.95833100	-0.23595600	-0.14729100
N	1.42989500	-1.47321200	-0.04293600
C	2.11884300	-2.39924800	0.63854300
C	-0.81442400	1.10755300	-2.07967600
H	3.83735600	-2.95760200	1.78044500
C	5.27496900	-0.57083900	1.80733300
H	3.58231600	1.09375700	0.31575100
C	1.48997600	2.09950300	-1.03174800
H	1.66690100	-3.38942200	0.71206400
C	0.65842400	2.99436000	-1.72561800
C	-0.52462900	2.46012400	-2.25577900
H	6.09929300	-2.57463500	1.38314000
H	-1.72403200	0.65835200	-2.48463600
H	2.41726500	2.45896600	-0.58969500

C	1.05905000	4.46885800	-1.86356300
C	1.17800100	5.08860400	-0.45192200
C	0.02505600	5.27429500	-2.66852000
C	2.42304800	4.55474600	-2.58633500
H	1.94118900	4.58173800	0.15786900
H	0.21815200	5.03064000	0.08523600
H	1.46360200	6.14954800	-0.53122400
H	-0.96356100	5.27126600	-2.18371700
H	-0.08930900	4.88505600	-3.69221000
H	0.35370400	6.32189400	-2.74565200
H	2.72367000	5.60888300	-2.69468000
H	2.36417100	4.10961700	-3.59211000
H	3.21875600	4.03663100	-2.02996500
C	5.15131300	-0.82050800	3.32827500
C	5.72255900	0.88340600	1.58186600
C	6.34296500	-1.51565400	1.20981100
H	6.11415700	-0.61181000	3.82091700
H	4.87912000	-1.86263300	3.55368100
H	4.38812000	-0.16338300	3.77430300
H	6.69318200	1.04959500	2.07308300
H	5.00725000	1.60339400	2.00901500
H	5.84877800	1.11198000	0.51211200
H	7.32101200	-1.31664300	1.67578000
H	6.44238000	-1.36052200	0.12387800
C	-1.68806700	0.06340200	2.65510400
C	-1.04035200	-0.87612900	1.86017200
C	-1.61043800	-1.32200000	0.64709100
C	-2.89218800	-0.83607100	0.30326100
C	-3.53951900	0.10791700	1.09524500
C	-2.94363700	0.57755800	2.28139200
H	-0.06562600	-1.25623800	2.17204600
Br	-1.94200400	-2.09369700	-2.96467500
H	-3.36392700	-1.18719700	-0.61553400
H	-4.51609000	0.49176900	0.79548300
C	-3.58529000	1.58840100	3.15580400
H	-1.22764900	0.41868100	3.57961300
O	-4.77732600	2.00558200	2.68667000
O	-3.10702000	2.01520800	4.19041600
C	-5.46518200	2.98534700	3.46662300
H	-6.39905400	3.20185400	2.93382300
H	-4.86318600	3.90187700	3.56461800
H	-5.68424800	2.60098200	4.47474900
C	-1.20474000	-3.29021400	0.34169600
H	-0.64970200	-3.33109600	1.28730300
C	-0.46978200	-4.18752700	-0.68397400
C	-2.61782400	-3.82678700	0.52733700
H	-0.24505700	-5.14800900	-0.18350500
H	0.49310000	-3.80715300	-1.06334600
H	-1.10129300	-4.38951800	-1.55725300
H	-3.13633500	-3.37136200	1.38211200
H	-2.58322300	-4.91891300	0.68911700
H	-3.21360900	-3.64380400	-0.38032900

TS1-^{n}Pr
CPCM(MeCN)/B3LYP-D3/def2-TZVPP//CPCM(MeCN)/B3LYP-D3/def2-SVP
E = -5471.222791
G = -5470.685018
N_{imag} = 1, 275.8 *i* cm^{-1}

H	-1.81979400	2.85612300	-2.65607600
C	0.96416200	1.01862900	-0.77251600
N	-0.05641800	0.33001400	-1.32495200
Ni	-0.20418800	-1.65113700	-0.77904700
C	3.70988000	-1.45693600	1.27107400
C	4.06378600	-0.10119100	1.15123600
C	3.15409500	0.71723200	0.47115400
C	1.96547700	0.19066400	-0.05102100
N	1.65421300	-1.11641500	0.07896000
C	2.51480600	-1.91251000	0.72772500
C	-1.01142600	0.99729300	-1.97458200
H	4.35498000	-2.17047100	1.78449400
C	5.37965500	0.41856900	1.74252300
H	3.36508700	1.77422500	0.33504000
C	1.03650100	2.40961000	-0.87299200
H	2.23486700	-2.96294400	0.81983400
C	0.04155900	3.13463600	-1.55077300
C	-1.00239900	2.38528000	-2.11251300
H	6.49915900	-1.42448000	1.26789300
H	-1.80704100	0.38223700	-2.40167000
H	1.86748300	2.93731200	-0.40837200
C	0.12932300	4.66389000	-1.63782000
C	0.12176800	5.24744200	-0.20588200
C	-1.05061000	5.26543700	-2.41976000
C	1.44484500	5.05272900	-2.35097500
H	0.97624100	4.89114500	0.38921800
H	-0.80294100	4.97306600	0.32593800
H	0.17985600	6.34659700	-0.24902300
H	-2.01605900	5.03924300	-1.94104000
H	-1.08328100	4.89821800	-3.45733000
H	-0.94700400	6.36052300	-2.45762900
H	1.52201900	6.14922000	-2.42258900
H	1.47658700	4.63909100	-3.37130700
H	2.33118200	4.69066900	-1.80821600
C	5.37811400	0.15874600	3.26674800
C	5.56953600	1.92571000	1.50067100
C	6.55586500	-0.34064200	1.08612600
H	6.31783800	0.52540500	3.70919800
H	5.29350000	-0.91286700	3.50202100
H	4.54151300	0.68335100	3.75469000
H	6.52336600	2.25149700	1.94239400
H	4.76806100	2.51948100	1.96714800
H	5.60159100	2.16804100	0.42698400
H	7.51083800	0.01886700	1.50090500
H	6.57140400	-0.17677100	-0.00294100
C	-2.04591200	-0.02111900	2.47895800
C	-1.11484800	-0.79703800	1.79618600
C	-1.47679000	-1.49406000	0.62357600

C	-2.82421700	-1.44105500	0.20206200
C	-3.75523300	-0.66532100	0.88596000
C	-3.37757100	0.06219500	2.03187400
H	-0.08509200	-0.84650600	2.15635000
Br	-1.26597700	-2.39373000	-2.96467400
H	-3.12100600	-1.99778400	-0.68879700
H	-4.78704300	-0.61624900	0.53414300
C	-4.32761600	0.91041800	2.79130800
H	-1.75404000	0.53938700	3.36981200
O	-5.56745100	0.90859200	2.26463900
O	-4.04330600	1.55147300	3.78595200
C	-6.54821200	1.70363900	2.93371000
H	-7.48139400	1.57785900	2.37142200
H	-6.25211200	2.76393200	2.94489000
H	-6.68321400	1.36700500	3.97313800
C	-0.57502300	-3.27966300	0.51204700
H	-0.02098900	-3.15823800	1.45349100
C	0.16894400	-4.27452600	-0.40716500
H	-1.54584000	-3.72572900	0.75671200
C	0.50677600	-5.55875300	0.36322600
H	1.10519000	-3.85682000	-0.81683800
H	-0.45723600	-4.50724300	-1.27826000
H	1.18084200	-5.35490800	1.21229800
H	-0.40459100	-6.02874600	0.76835100
H	1.00358500	-6.29188700	-0.29314200

Int6
CPCM(MeCN)/B3LYP-D3/def2-TZVPP//CPCM(MeCN)/B3LYP-D3/def2-SVP
E = -4892.953292
G = -4892.623957
N_{imag} = 0

Ni	1.36935100	-1.87428600	-0.00004400
H	-6.51226100	-0.87851600	-1.27832000
H	-5.33394000	-2.20990800	-1.29659800
H	-0.88868100	4.61468800	-0.89284800
H	-4.98169600	-0.70155300	-2.17774400
H	1.77537200	6.08947200	1.27924700
H	2.88431000	4.69981100	1.29633300
H	-6.10561600	1.20832000	0.00009600
H	-4.59661000	1.49101500	-0.89282700
H	-4.59648900	1.49103200	0.89280500
H	-4.98153700	-0.70155100	2.17779900
N	-0.54612600	-1.56711000	-0.00004300
C	-3.28632500	-0.85772400	-0.00001900
C	-1.50829200	-2.50782000	-0.00006300
C	-0.92490200	-0.26288800	-0.00001300
C	-2.27411400	0.10994600	0.00000200
C	-2.86216200	-2.20197700	-0.00005000
N	1.42177600	0.10599500	-0.00000900
C	1.19811200	2.91594600	0.00000800
C	2.51551500	0.88033500	-0.00000200
C	0.20475600	0.69521100	-0.00000200
C	0.06727900	2.08904600	0.00001800

C	2.44995400	2.26826400	-0.00000700
H	-1.16149700	-3.54378800	-0.00008500
H	-2.52410600	1.16810100	0.00003300
H	-3.58143900	-3.02204000	-0.00006600
H	3.47012900	0.34724000	0.00000200
H	-0.93099900	2.51951900	0.00004200
H	3.38082200	2.83674800	-0.00002500
Br	3.42883200	-2.99801800	0.00004100
H	1.33799800	4.61170700	2.17785200
H	-5.33385200	-2.20990300	1.29667500
C	1.11484500	4.44828200	-0.00000100
C	-0.33898700	4.95100200	0.00003200
C	1.82301400	4.98894500	1.26360900
C	1.82294900	4.98892200	-1.26365400
C	-4.78063000	-0.51094500	0.00002000
C	-5.43617800	-1.11467800	1.26346700
C	-5.02336400	1.00804000	0.00002200
C	-5.43626600	-1.11468300	-1.26337800
H	1.77534200	6.08945000	-1.27928800
H	1.33786500	4.61169700	-2.17786600
H	2.88423300	4.69975200	-1.29644900
H	-0.34826700	6.05158500	0.00001700
H	-0.88863100	4.61471800	0.89295600
H	-6.51217100	-0.87850400	1.27848900

TS3
CPCM(MeCN)/B3LYP-D3/def2-TZVPP//CPCM(MeCN)/B3LYP-D3/def2-SVP
E = -2437.226164
G = -2436.809981
$\boldsymbol{N}_{\mathbf{imag}}$ = 1, 53.2 *i* cm^{-1}

Ni	0.53459900	-2.55632200	-0.11977700
H	-6.20776900	1.69059600	-1.14427700
H	-5.67534800	-0.00237500	-1.26270200
H	1.10023800	4.33336900	-0.82964800
H	-4.75048700	1.27716700	-2.08734300
H	4.18902400	4.54368400	1.27647100
H	4.63458900	2.82230000	1.22900600
H	-4.95344400	3.35872900	0.20335700
H	-3.47337500	3.03765700	-0.72682700
H	-3.44892500	2.94481700	1.05455600
H	-4.69368700	1.05163100	2.25935500
N	-1.05089900	-1.50439900	-0.08108600
C	-3.23824200	0.31438100	0.02244900
C	-2.33100500	-1.94092500	-0.08969800
C	-0.82782900	-0.13471500	-0.03870500
C	-1.93167500	0.76337300	0.02359400
C	-3.42756500	-1.10501400	-0.04515800
N	1.45316100	-0.80275400	-0.12744300
C	2.34900600	1.91227000	-0.05053400
C	2.76415900	-0.48281600	-0.14561100
C	0.54551500	0.25003100	-0.06667000
C	1.00411600	1.59809100	-0.03150800
C	3.25656300	0.80669300	-0.10937300

H	-2.46066200	-3.02329900	-0.13638600
H	-1.71625400	1.82857800	0.07001500
H	-4.42403500	-1.54687600	-0.06134700
H	3.46670300	-1.31544400	-0.19117900
H	0.25605400	2.38665400	0.01232300
H	4.33647500	0.95508800	-0.12752500
C	2.29590200	-3.61065100	-0.14639800
H	3.20720100	3.34298700	2.15861800
H	-5.64242300	-0.13617400	1.33110800
C	2.88708000	3.34750800	-0.01234700
C	1.75977500	4.39204900	0.05031800
C	3.78315600	3.51963500	1.23604400
C	3.72566700	3.60864700	-1.28495500
C	-4.45443700	1.24628800	0.08677700
C	-5.28866600	0.90589500	1.34347800
C	-4.05130500	2.72893900	0.15828400
C	-5.32139100	1.03609800	-1.17629900
H	4.13093200	4.63365700	-1.27158900
H	3.10801100	3.49642100	-2.19047200
H	4.57529600	2.91379400	-1.36562000
H	2.19087800	5.40495800	0.07675900
H	1.14044600	4.26958800	0.95263600
H	-6.17410200	1.55975800	1.40250200
H	2.92251600	-3.07759300	-0.86866700
C	1.30858600	-4.44018500	-0.65632100
C	2.84104500	-3.74785900	1.25149600
H	1.13973900	-4.50955700	-1.73622900
H	-0.34574400	-3.68190600	0.09069200
H	0.92734400	-5.28287100	-0.07366400
H	3.07699500	-2.76538900	1.68878600
H	3.77260300	-4.34174500	1.25564500
H	2.11588300	-4.24750800	1.91192500

TS4
CPCM(MeCN)/B3LYP-D3/def2-TZVPP//CPCM(MeCN)/B3LYP-D3/def2-SVP
E = -2437.226721
G = -2436.809909
N_{imag} = 1, 46.2 *i* cm^{-1}

Ni	-0.08537100	2.56102300	-0.38072100
H	5.62785800	-3.02246900	-1.11157500
H	5.46230000	-1.26068000	-1.29245800
H	-2.11470800	-4.06935100	-0.55240600
H	4.29490100	-2.34661800	-2.08652100
H	-5.19975600	-3.42267700	1.46807000
H	-5.25410200	-1.65544200	1.27197900
H	4.04615300	-4.34340000	0.27333700
H	2.66956600	-3.75318200	-0.68385800
H	2.65751900	-3.59608000	1.09311800
H	4.26178000	-1.96248000	2.24930200
N	1.24707100	1.20787400	-0.26336700
C	3.00460900	-1.01677700	-0.02863900
C	2.58913900	1.36974500	-0.26527600
C	0.74178800	-0.07772300	-0.12196400

C	1.63271400	-1.18358400	-0.02028400
C	3.48643500	0.32819800	-0.14792800
N	-1.33933500	1.05873300	-0.23141900
C	-2.80794800	-1.37682500	-0.00373600
C	-2.68788200	1.03827500	-0.27716700
C	-0.68296800	-0.15882900	-0.09606300
C	-1.42644400	-1.36626800	0.03065500
C	-3.45042200	-0.10856200	-0.17656900
H	2.93974100	2.39822800	-0.36628900
H	1.19865300	-2.17721500	0.06849400
H	4.55321000	0.55294600	-0.15040900
H	-3.18010300	2.00351200	-0.40541000
H	-0.87099900	-2.29362300	0.15399500
H	-4.53602700	-0.02228000	-0.22851000
C	-1.53166800	3.98180400	-0.21553100
H	-3.98865300	-2.39643200	2.28296300
H	5.44218100	-1.03080400	1.29467400
C	-3.65005100	-2.65148400	0.12988400
C	-2.78211900	-3.90871300	0.30869600
C	-4.57771500	-2.51912800	1.35990800
C	-4.50928400	-2.82785900	-1.14349200
C	3.99945000	-2.17874700	0.08137800
C	4.87915400	-1.97546700	1.33676800
C	3.29546100	-3.54142700	0.19714200
C	4.89838600	-2.19863600	-1.17659600
H	-5.13070600	-3.73462200	-1.06204300
H	-3.87060100	-2.92941900	-2.03557900
H	-5.18390400	-1.97343400	-1.30481900
H	-3.42672000	-4.79670300	0.40088200
H	-2.16277800	-3.85059300	1.21743400
H	5.60885500	-2.79652900	1.42847900
H	-2.00474700	4.17158300	-1.18554700
C	-0.32807600	4.59646100	0.11401200
H	-2.19452400	3.64025900	0.58553400
H	0.12053200	5.28541700	-0.60824800
H	0.98111000	3.44583900	-0.79808700
C	0.15166600	4.76121300	1.53271000
H	-0.10994800	5.77056700	1.89716200
H	1.24445700	4.65511500	1.59859100
H	-0.30964500	4.02168100	2.20400300

Int7

CPCM(MeCN)/B3LYP-D3/def2-TZVPP//CPCM(MeCN)/B3LYP-D3/def2-SVP

E = -2437.242678

G = -2436.827131

$\boldsymbol{N}_{\mathbf{imag}}$ = 0

Ni	0.50289600	2.72757800	-0.56594600
H	4.33438900	-4.52190700	-1.09473600
H	4.72032200	-2.80377100	-1.34211700
H	-3.26787000	-3.26324600	-0.40942200
H	3.25887900	-3.50092600	-2.08654700
H	-6.00242100	-1.68647300	1.59817300
H	-5.59767800	0.01544700	1.28051600

H	2.45833200	-5.24279200	0.36251700
H	1.31150800	-4.30328000	-0.61503900
H	1.37728500	-4.07735900	1.15506000
H	3.42960800	-2.95925100	2.23198800
N	1.44185700	0.83841000	-0.35878100
C	2.47707100	-1.77461000	-0.07517600
C	2.76187600	0.61062100	-0.38375300
C	0.61033200	-0.21368400	-0.20712700
C	1.10166200	-1.52079900	-0.06398400
C	3.31575500	-0.65578700	-0.24359000
N	-1.12346000	1.41393700	-0.45428600
C	-3.19753900	-0.45512000	-0.03207600
C	-2.40936000	1.79144300	-0.47905800
C	-0.83645000	0.11685100	-0.21991200
C	-1.85028000	-0.83037800	-0.00743700
C	-3.46108100	0.90713500	-0.27733000
H	3.39675000	1.48748600	-0.52804900
H	0.39546600	-2.33882900	0.04955800
H	4.40121200	-0.75915100	-0.27157300
H	-2.59311200	2.85277200	-0.66850000
H	-1.57413100	-1.86406700	0.18302200
H	-4.48204900	1.28955000	-0.31276500
C	0.51893600	3.30081200	1.43682500
H	-4.54189100	-0.96698200	2.32755700
H	4.82282400	-2.48398300	1.22735400
C	-4.34854500	-1.44444400	0.19361600
C	-3.84319800	-2.87532100	0.44560600
C	-5.16878900	-0.98808000	1.42203900
C	-5.25331900	-1.45469700	-1.05993300
C	3.07214300	-3.18060300	0.07719000
C	3.99419600	-3.20239300	1.31792900
C	1.98548000	-4.25483000	0.25441300
C	3.89551500	-3.51572800	-1.18774100
H	-6.08872000	-2.15813100	-0.91500300
H	-4.68803600	-1.77234100	-1.95041400
H	-5.68323900	-0.46262000	-1.26481400
H	-4.70118500	-3.54713900	0.60026000
H	-3.20995100	-2.93367200	1.34458300
H	4.43293700	-4.20521900	1.44233400
H	-0.50195100	3.61823300	1.68173300
C	1.37551900	4.14532700	0.72344300
H	0.92407800	2.48955900	2.05346900
H	0.98345600	5.12114300	0.40518800
H	0.80828300	3.26124000	-2.14289200
C	2.88052900	4.06606600	0.81158400
H	3.27539500	4.79714000	1.54202200
H	3.36064400	4.28206100	-0.15640800
H	3.20828600	3.06720900	1.13773800

Int8-Me
CPCM(MeCN)/B3LYP-D3/def2-TZVPP//CPCM(MeCN)/B3LYP-D3/def2-SVP
E = -2398.534036
G = -2398.132596

N_{imag} = 0

Ni	-0.00014700	2.83380300	0.00000900
C	1.32119400	4.20156900	0.36870100
H	5.36028900	-1.25006300	-1.28665500
H	-2.35950300	-3.68727200	-0.73567800
H	4.12023500	-2.11355500	-2.23162100
H	-5.38533800	-3.02618100	1.36494000
H	-5.36090100	-1.24990900	1.28546100
H	3.70028300	-4.41036400	-0.17683400
H	2.38482700	-3.59341000	-1.04618200
H	2.36016600	-3.68687200	0.73704900
H	4.06829400	-2.34732600	2.11769000
N	1.29223100	1.31725600	0.10195800
C	2.91258200	-1.00983800	-0.00477800
C	2.63314800	1.39603700	0.12244300
C	0.73811700	0.08403400	0.02028400
C	1.51545800	-1.07981200	-0.03115200
C	3.46260800	0.28261400	0.07277400
N	-1.29221800	1.31721200	-0.10211300
C	-2.91249600	-1.00999300	0.00477600
C	-2.63315700	1.39586500	-0.12309100
C	-0.73809000	0.08400200	-0.01998100
C	-1.51538200	-1.07986200	0.03163800
C	-3.46255700	0.28240500	-0.07342800
H	3.05360300	2.39939600	0.18454600
H	1.01439200	-2.04153000	-0.10203800
H	4.54187000	0.43898600	0.09488800
H	-3.05368700	2.39916200	-0.18554600
H	-1.01426700	-2.04151900	0.10303500
H	-4.54181900	0.43871200	-0.09603000
C	-1.32150200	4.20165100	-0.36829500
H	-4.12129300	-2.11292400	2.23144200
H	5.33068000	-1.39091300	1.30031200
C	-3.81788200	-2.24642200	0.06120900
C	-3.01064400	-3.55329600	0.14217000
C	-4.72391900	-2.14692700	1.31001900
C	-4.69236300	-2.28439300	-1.21326500
C	3.81802900	-2.24623000	-0.06116900
C	4.69320700	-2.28362800	1.21283700
C	3.01085300	-3.55320300	-0.14118300
C	4.72337300	-2.14714800	-1.31051700
H	-5.35233300	-3.16613200	-1.18940900
H	-4.06694800	-2.34844300	-2.11774700
H	-5.32981900	-1.39173700	-1.30146500
H	-3.70002100	-4.41050000	0.17782100
H	-2.38507100	-3.59307500	1.04750200
H	5.35320100	-3.16534900	1.18898800
H	5.38483600	-3.02636700	-1.36546200
H	1.97013300	3.87715500	1.20849400
H	0.92720200	5.19686200	0.63978800
H	1.98706800	4.35812200	-0.50673500
H	-1.97046500	3.87797400	-1.20834800
H	-0.92754500	5.19720000	-0.63850700

H	-1.98750500	4.35754800	0.50718600

Int8-Et
CPCM(MeCN)/B3LYP-D3/def2-TZVPP//CPCM(MeCN)/B3LYP-D3/def2-SVP
E = -2477.189037
G = -2476.732235
N_{imag} = 0

Ni	-0.00021600	2.49534500	0.00005900
C	1.31652400	3.89378600	0.27141000
H	5.38907600	-1.63559200	-1.13465600
H	-2.31623900	-4.05141700	-0.80478900
H	4.17217100	-2.49320500	-2.11435000
H	-5.40485300	-3.41320000	1.21009600
H	-5.38877500	-1.63662300	1.13479900
H	3.67807800	-4.78418100	-0.06990800
H	2.39308600	-3.96025100	-0.97752900
H	2.31681100	-4.05122800	0.80431700
H	3.99168100	-2.72045600	2.23180100
N	1.29529300	0.95986200	0.12299200
C	2.90663700	-1.37862700	0.07440900
C	2.63534900	1.02787400	0.18815000
C	0.73779200	-0.27136500	0.03546900
C	1.51060900	-1.43963200	0.00915000
C	3.46087600	-0.08934400	0.16683800
N	-1.29541900	0.95969800	-0.12253000
C	-2.90642100	-1.37905000	-0.07431400
C	-2.63549700	1.02751400	-0.18764500
C	-0.73774300	-0.27146600	-0.03521200
C	-1.51038500	-1.43985200	-0.00907900
C	-3.46085100	-0.08983000	-0.16650800
H	3.06206300	2.02723900	0.26544700
H	1.00717800	-2.39919300	-0.07119800
H	4.53961200	0.06206100	0.22325400
H	-3.06237000	2.02682300	-0.26475200
H	-1.00680100	-2.39934600	0.07110300
H	-4.53961200	0.06142500	-0.22286600
C	-1.31710700	3.89356700	-0.27173500
H	-4.17169800	-2.49423700	2.11427200
H	5.28256300	-1.77186900	1.45073900
C	-3.80534800	-2.62111400	-0.04666200
C	-2.99263600	-3.92319100	0.05464200
C	-4.74739400	-2.52991500	1.17584800
C	-4.64277600	-2.66169000	-1.34564800
C	3.80575000	-2.62055400	0.04654600
C	4.64313200	-2.66126500	1.34555600
C	2.99323400	-3.92273100	-0.05505300
C	4.74783300	-2.52897300	-1.17590900
H	-5.29824400	-3.54711000	-1.34222700
H	-3.99134800	-2.72060600	-2.23192800
H	-5.28234200	-1.77236600	-1.45062700
H	-3.67735200	-4.78474500	0.06936300
H	-2.39243600	-3.96080700	0.97708000
H	5.29873500	-3.54658600	1.34198400

H	5.40542700	-3.41215200	-1.21029700
H	2.07660200	3.54223900	1.00136100
H	0.88916300	4.80873600	0.72271400
C	1.99983000	4.26179100	-1.04916500
H	-2.07711300	3.54161000	-1.00155700
H	-0.88979900	4.80836500	-0.72339500
C	-2.00046900	4.26210100	1.04866300
H	2.46082900	3.38246200	-1.53354400
H	2.80054500	5.02200600	-0.94152200
H	1.26943700	4.66578100	-1.77118000
H	-2.46132600	3.38295600	1.53351100
H	-2.80131800	5.02212400	0.94063800
H	-1.27012900	4.66658100	1.77045400

Int8-^{i}Pr
CPCM(MeCN)/B3LYP-D3/def2-TZVPP//CPCM(MeCN)/B3LYP-D3/def2-SVP
E = -2555.838652
G = -2555.330175
N_{imag} = 0

Ni	0.00008400	2.23675000	-0.00003300
C	1.01682200	3.64479700	0.91146500
H	5.33838900	-1.77821300	-1.45098400
H	-2.36371500	-4.36927000	-0.41099600
H	4.08395300	-2.56180500	-2.44531100
H	-5.35942300	-3.54194200	1.67525100
H	-5.33776500	-1.77781800	1.45185700
H	3.69100400	-5.01841000	-0.57549000
H	2.36556600	-4.13412900	-1.35987800
H	2.36315800	-4.36954100	0.41017600
H	4.09398100	-3.14698700	1.87087800
N	1.28685700	0.67192400	0.20332900
C	2.90830200	-1.64330900	-0.11891000
C	2.62916900	0.74333900	0.20918400
C	0.73689700	-0.55183200	0.02196600
C	1.51128900	-1.70917000	-0.13391600
C	3.45865600	-0.36004800	0.05526600
N	-1.28678100	0.67198100	-0.20334800
C	-2.90838300	-1.64314500	0.11881500
C	-2.62910500	0.74346800	-0.20935800
C	-0.73690600	-0.55178900	-0.02188700
C	-1.51137800	-1.70908100	0.13398000
C	-3.45866000	-0.35986500	-0.05549800
H	3.05475200	1.73432500	0.34981500
H	1.00458800	-2.65852500	-0.28851300
H	4.53794300	-0.20341800	0.07535100
H	-3.05458600	1.73448300	-0.35012600
H	-1.00474500	-2.65845700	0.28866200
H	-4.53794100	-0.20320400	-0.07570900
C	-1.01660200	3.64481100	-0.91156100
H	-4.08309000	-2.56192100	2.44548600
H	5.34453300	-2.12640700	1.11541200
C	-3.81261700	-2.86987400	0.28881700
C	-3.00346500	-4.16652600	0.46204200

C	-4.69970200	-2.67017600	1.53914200
C	-4.70662600	-3.00933700	-0.96483500
C	3.81244000	-2.87012000	-0.28881900
C	4.70586200	-3.00991900	0.96523100
C	3.00315800	-4.16661000	-0.46263400
C	4.70012300	-2.67038000	-1.53870200
H	-5.36632900	-3.88543900	-0.86040200
H	-4.09518400	-3.14634800	-1.87078900
H	-5.34526900	-2.12573500	-1.11460300
H	-3.69139800	-5.01826500	0.57483100
H	-2.36564300	-4.13442400	1.35913500
H	5.36549600	-3.88609500	0.86096300
H	5.35969400	-3.54227700	-1.67468700
C	1.43955200	3.13470300	2.29548100
H	0.29872300	4.46517000	1.09691800
C	2.18175600	4.26886400	0.14107100
C	-1.43963300	3.13483000	-2.29552200
H	-0.29847600	4.46513200	-1.09715000
C	-2.18131100	4.26885100	-0.14082100
H	2.97792200	3.54679700	-0.10815400
H	2.67591900	5.08342500	0.71212900
H	1.84242800	4.70239600	-0.81454300
H	-2.97737200	3.54674300	0.10860500
H	-2.67566500	5.08344500	-0.71167600
H	-1.84171900	4.70232800	0.81472300
H	0.57246600	2.76531000	2.86974200
H	1.91377000	3.93444700	2.90336400
H	2.16423600	2.30658200	2.25118300
H	-2.16430300	2.30671000	-2.25116500
H	-0.57266300	2.76544900	-2.86996700
H	-1.91391100	3.93464800	-2.90325900

9. NMR Spectra of Compounds

^{1}H NMR (400 MHz, $CDCl_3$) of **3**

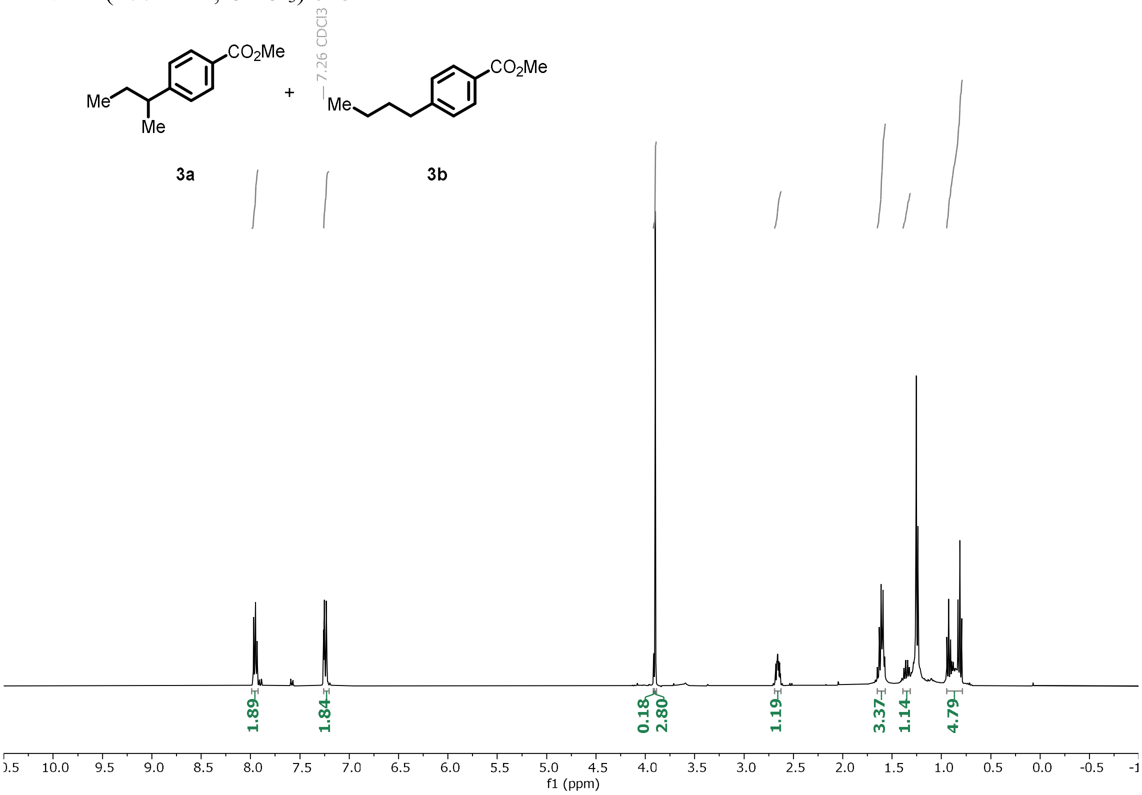

^{13}C NMR (101 MHz, $CDCl_3$) of **3**

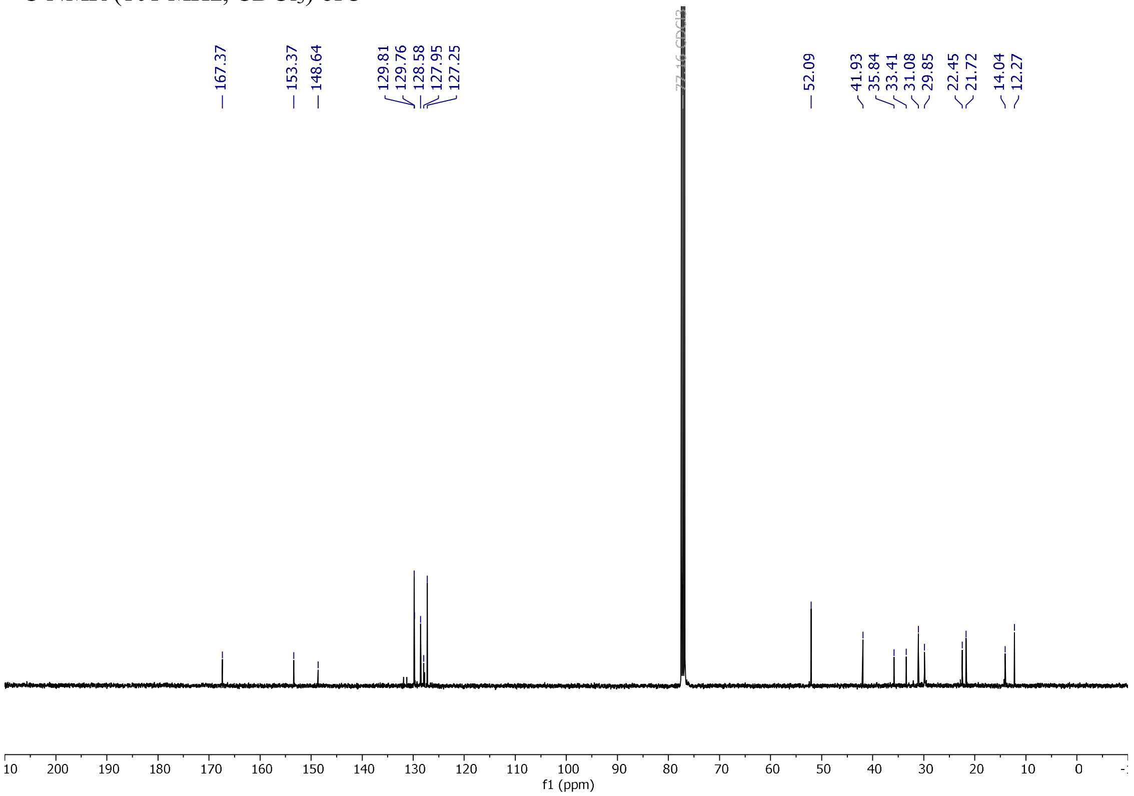

^{1}H NMR (400 MHz, $CDCl_3$) of **4**

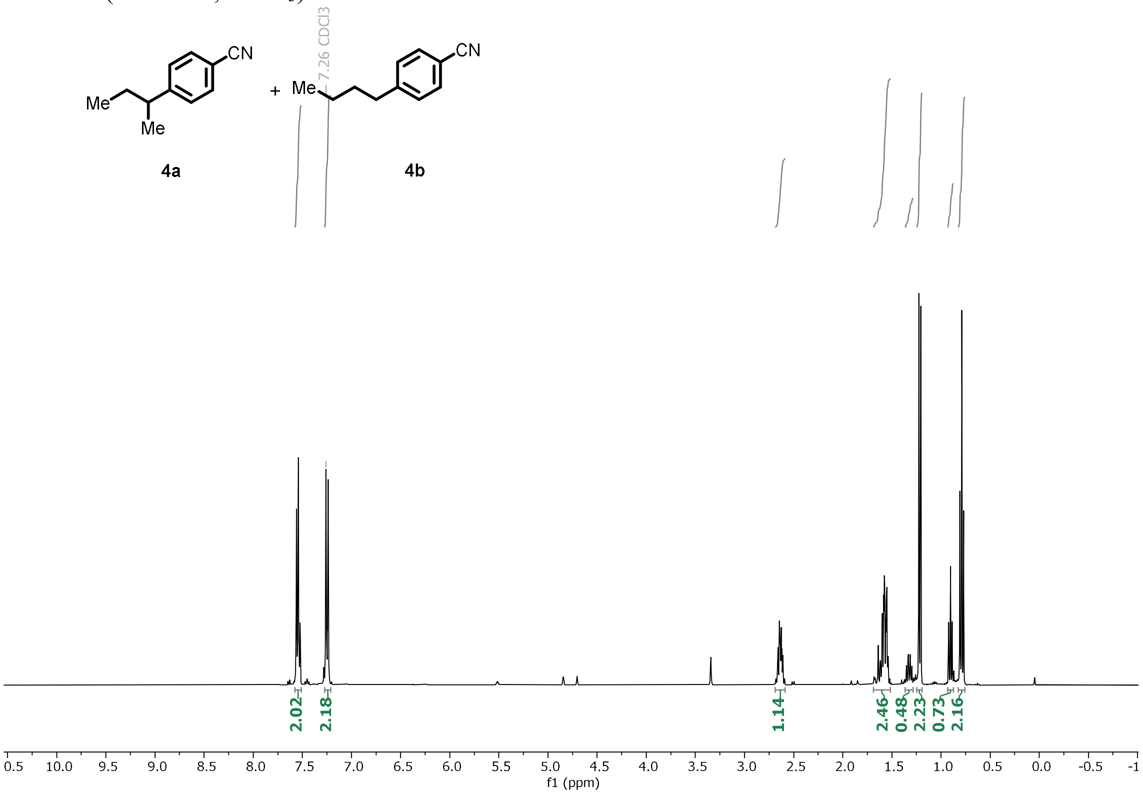

^{13}C NMR (101 MHz, $CDCl_3$) of **4**

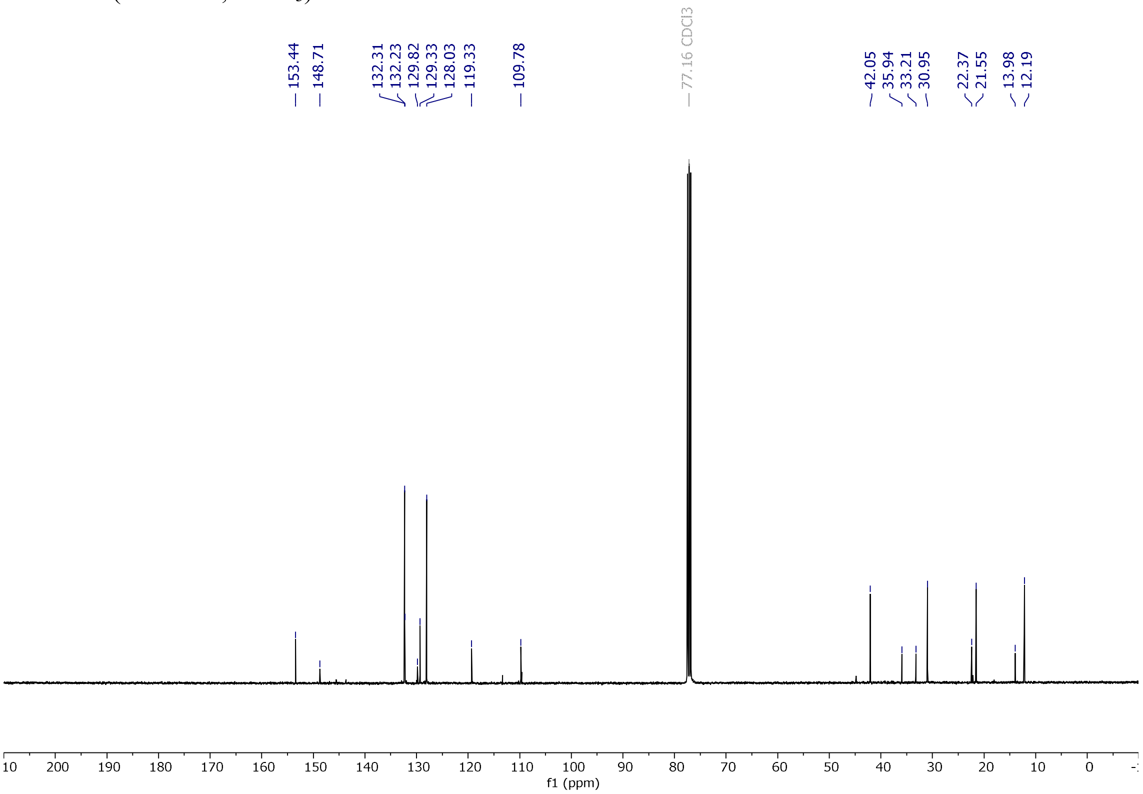

^{1}H NMR (400 MHz, $CDCl_3$) of **5**

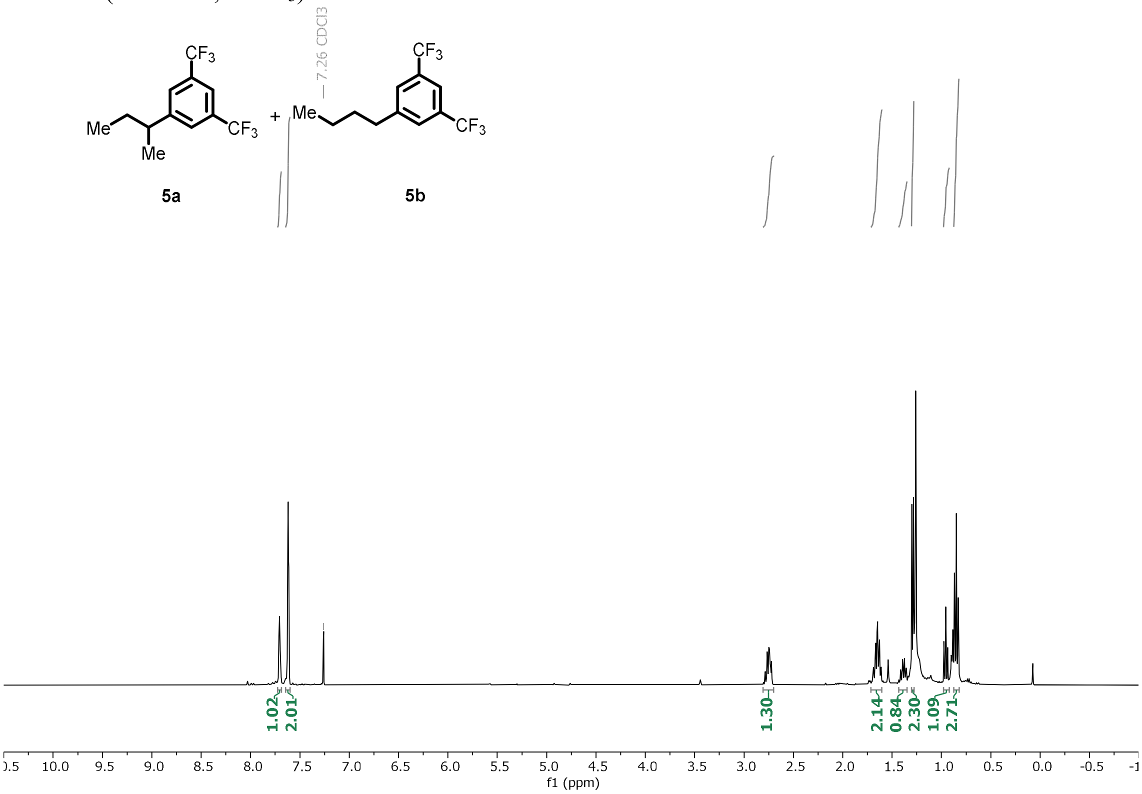

^{13}C NMR (101 MHz, $CDCl_3$) of **5**

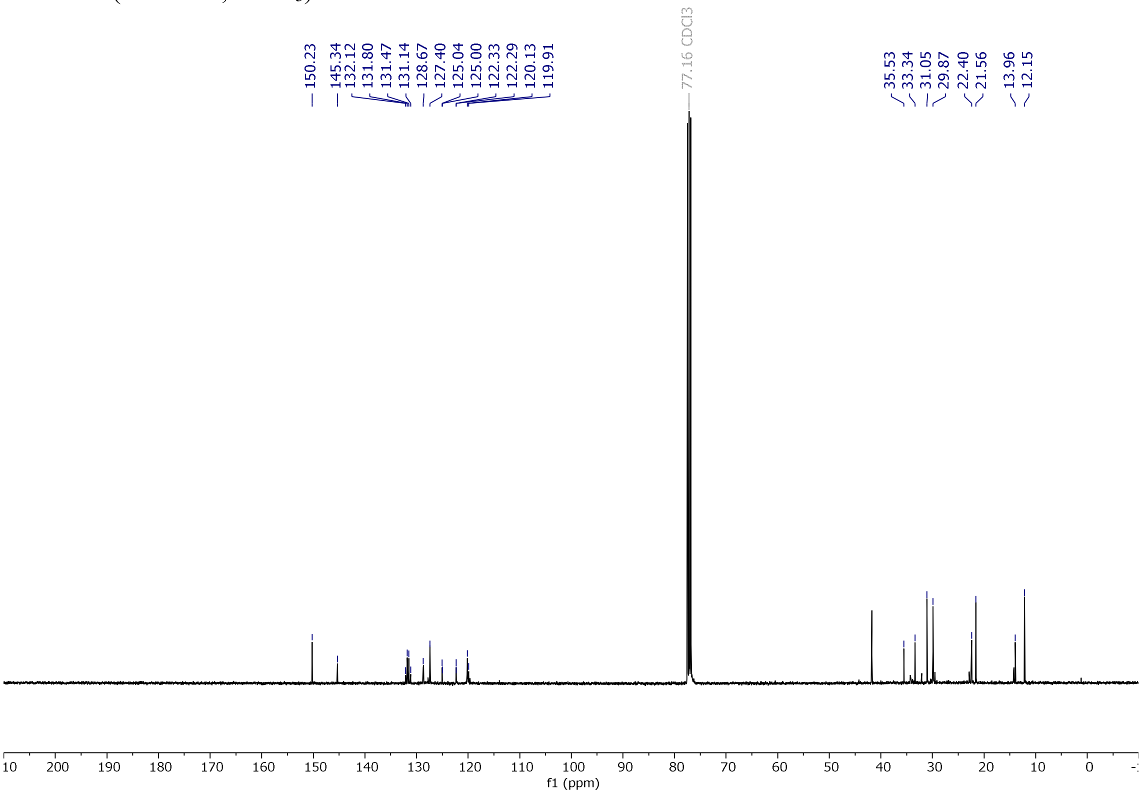

^{19}F NMR (282 MHz, $CDCl_3$) of **5**

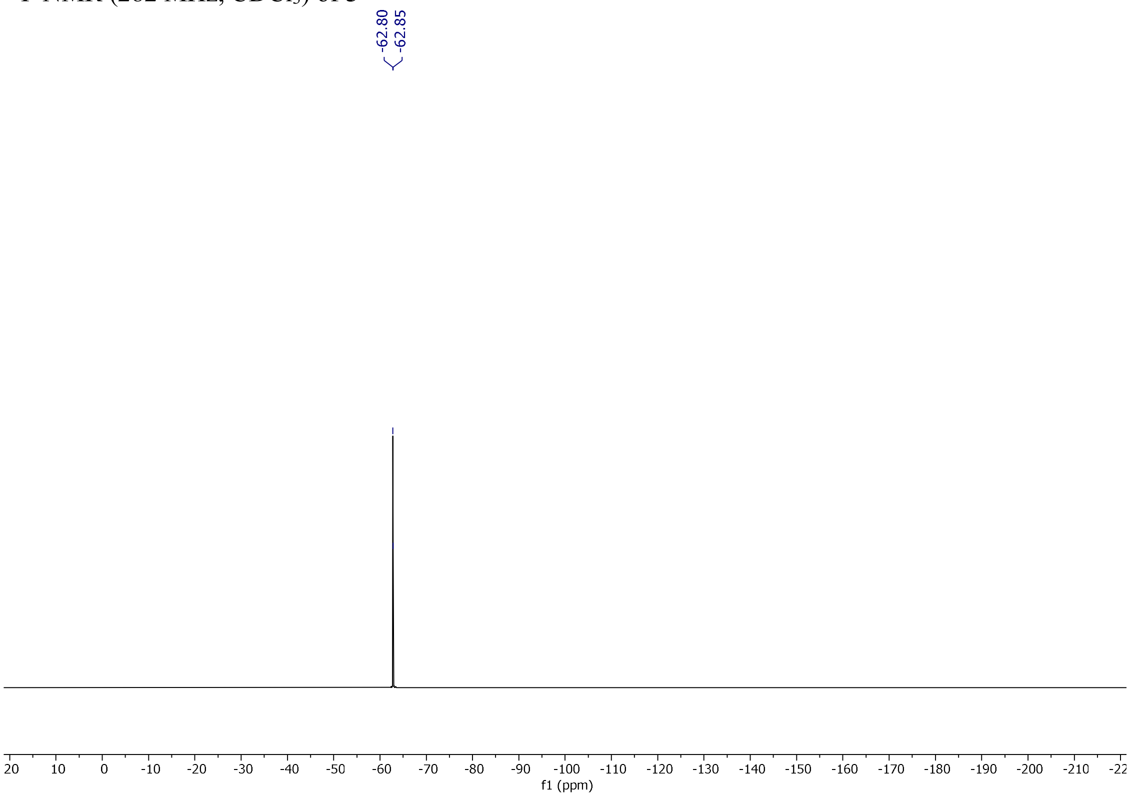

^{1}H NMR (400 MHz, $CDCl_3$) of **6**

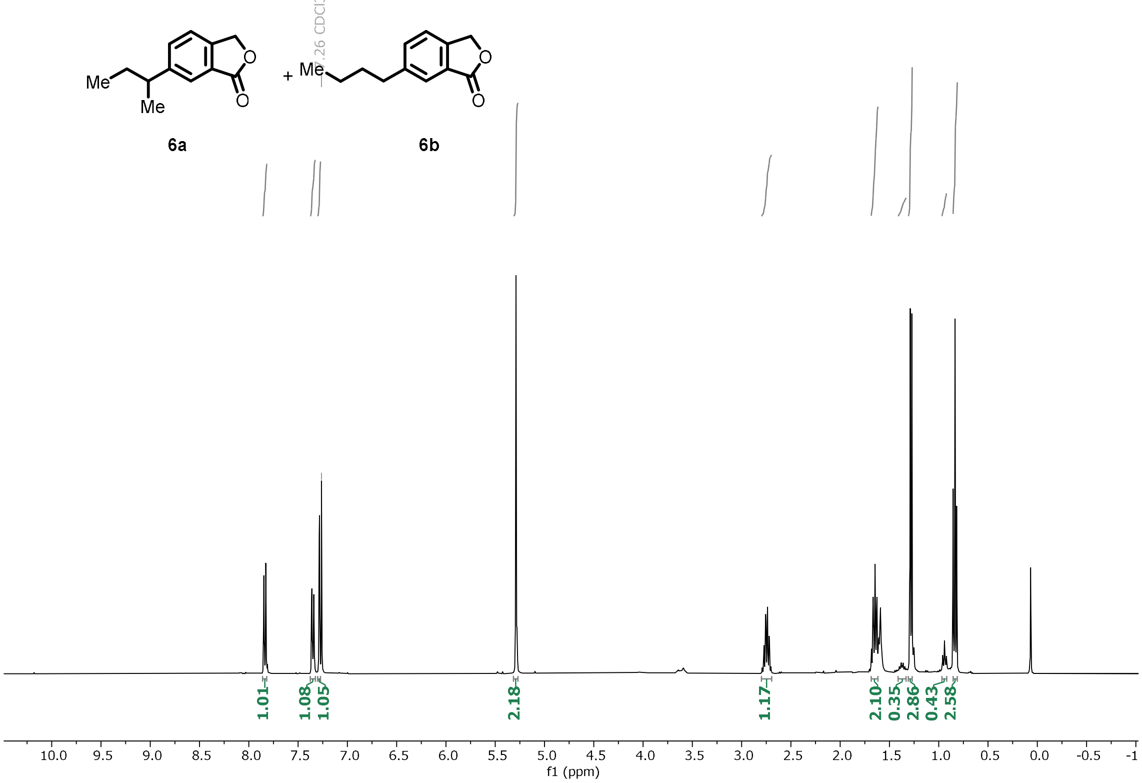

^{13}C NMR (101 MHz, $CDCl_3$) of **6**

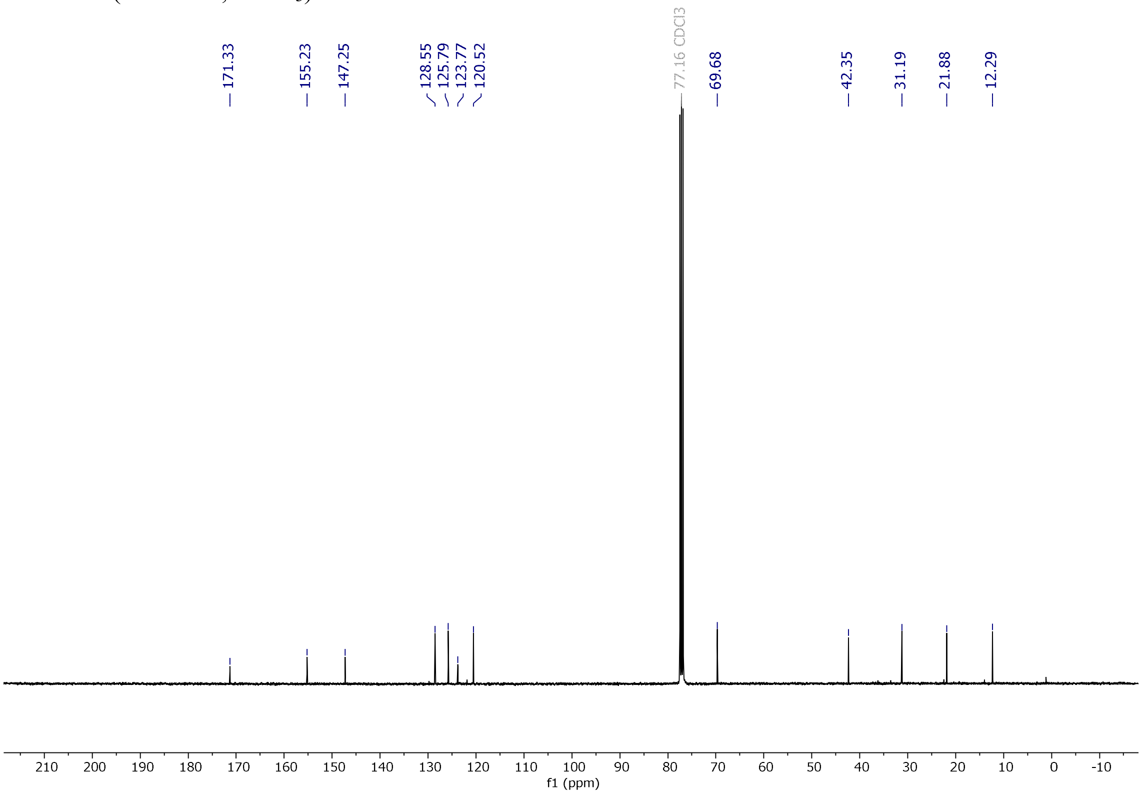

^{1}H NMR (400 MHz, $CDCl_3$) of **7**

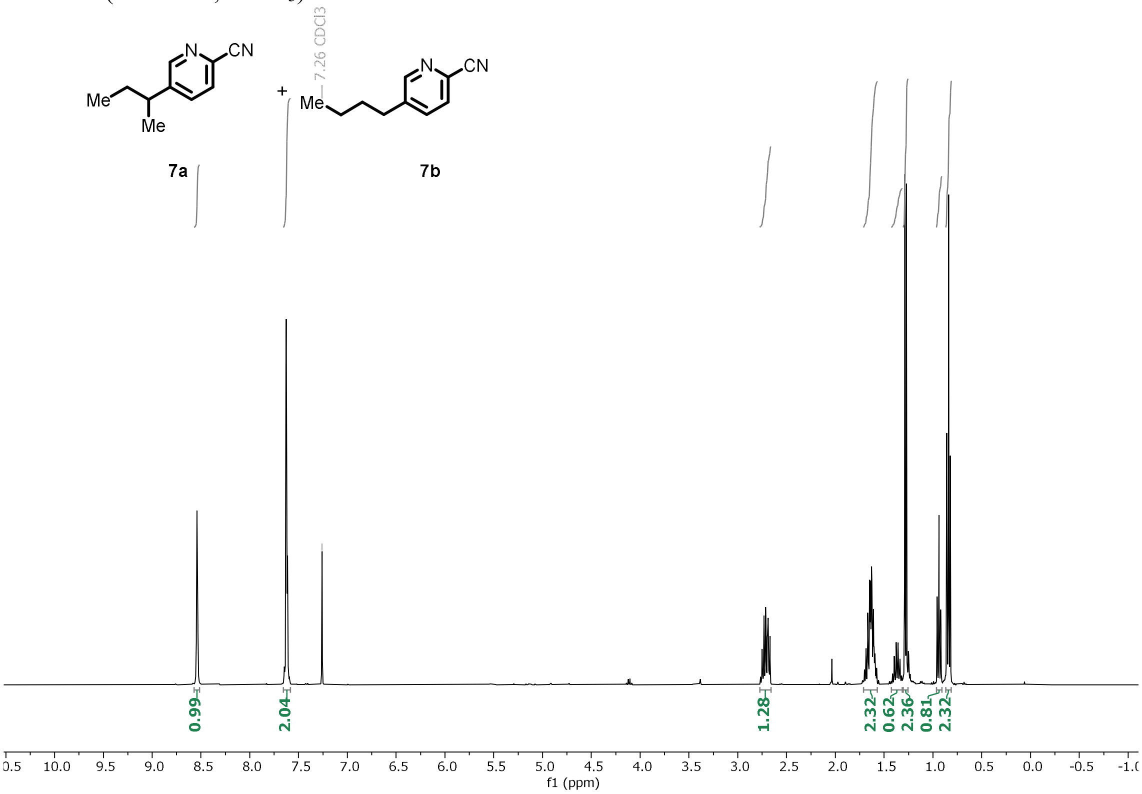

^{13}C NMR (101 MHz, $CDCl_3$) of **7**

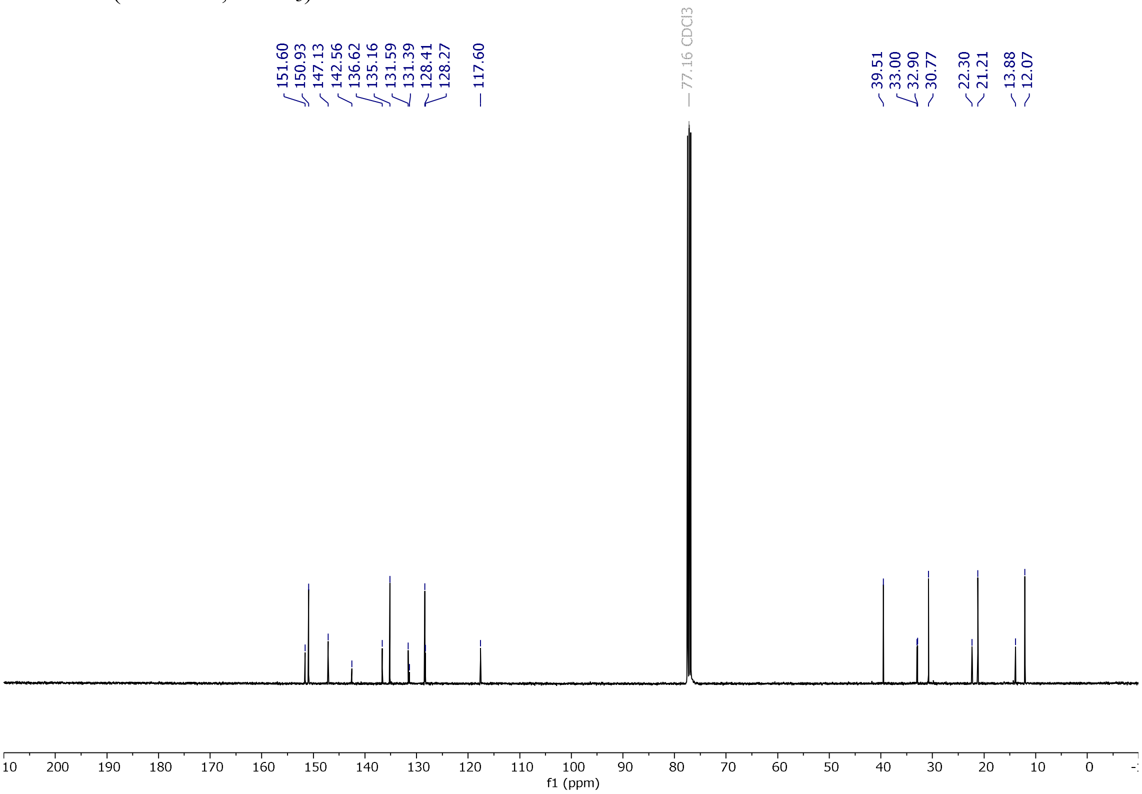

^{1}H NMR (400 MHz, $CDCl_3$) of **7**

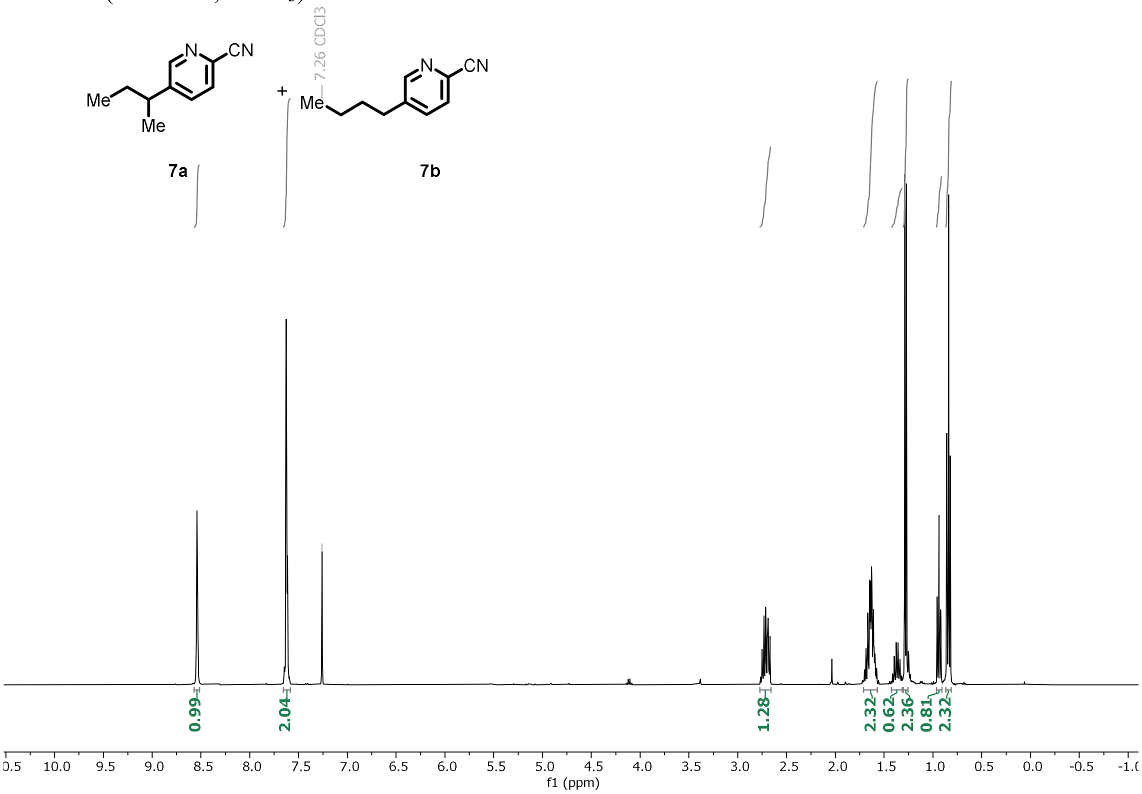

^{13}C NMR (101 MHz, $CDCl_3$) of **7**

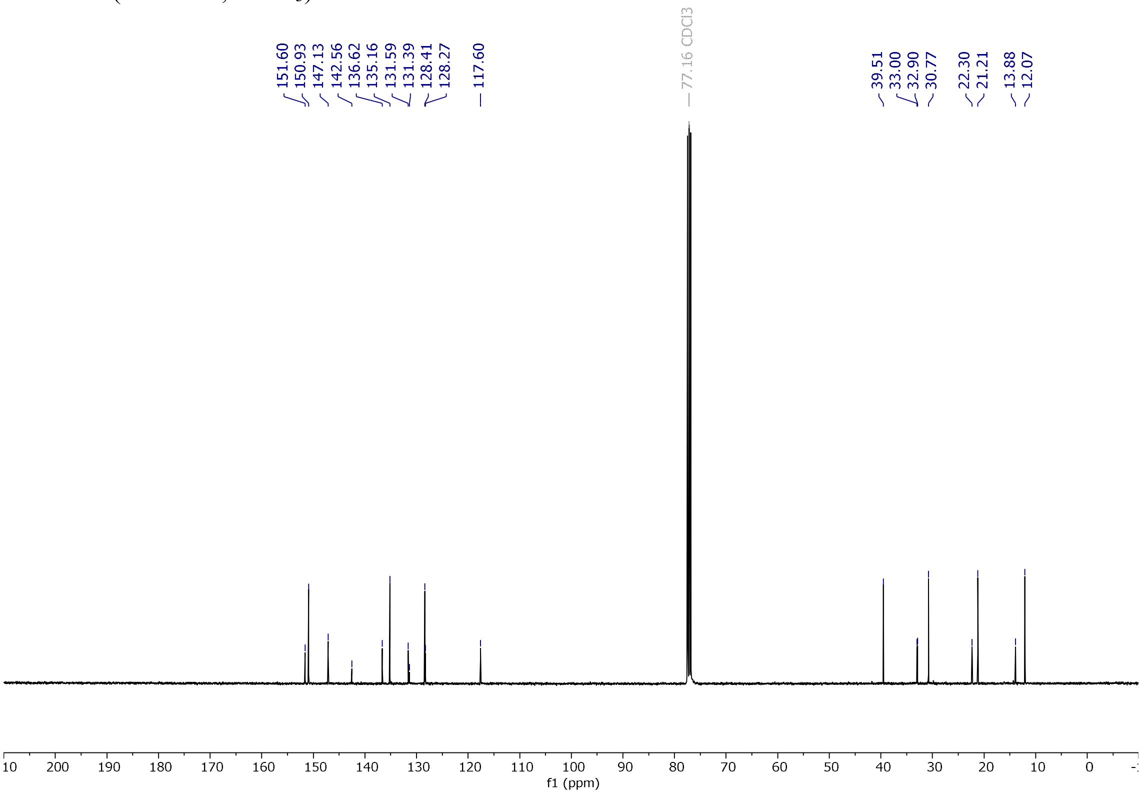

^{1}H NMR (400 MHz, $CDCl_3$) of **8**

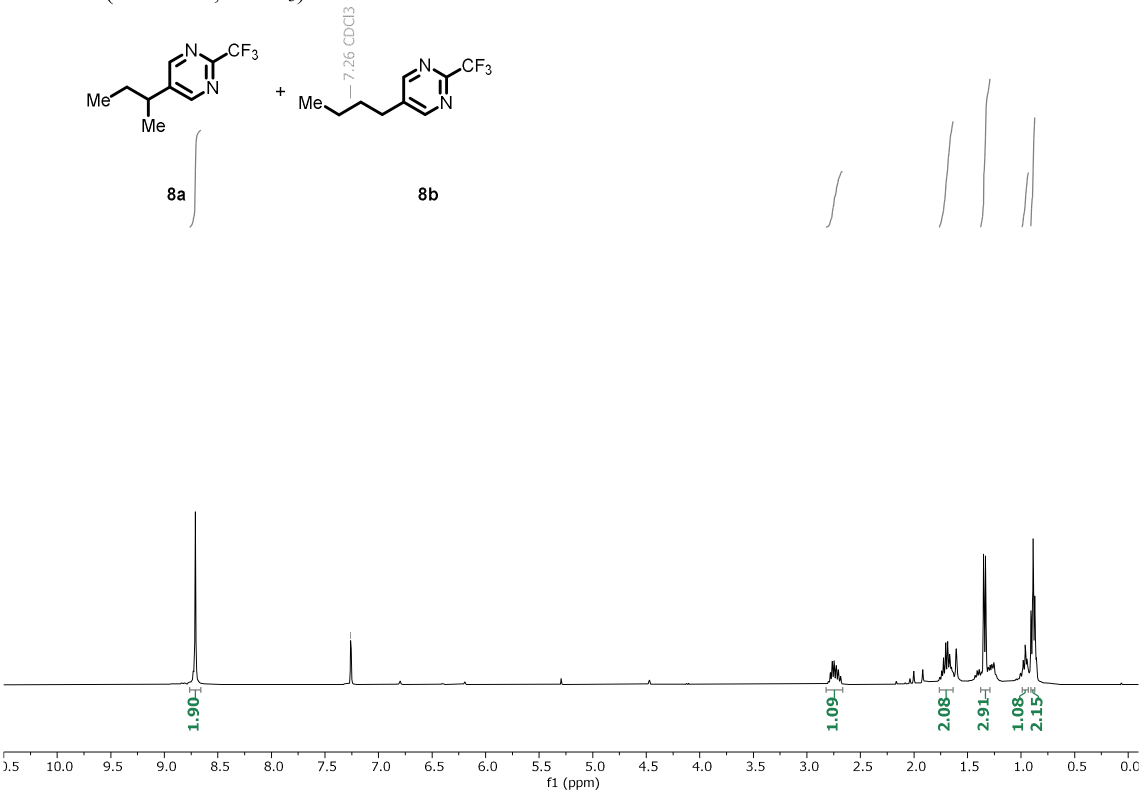

^{13}C NMR (101 MHz, $CDCl_3$) of **8**

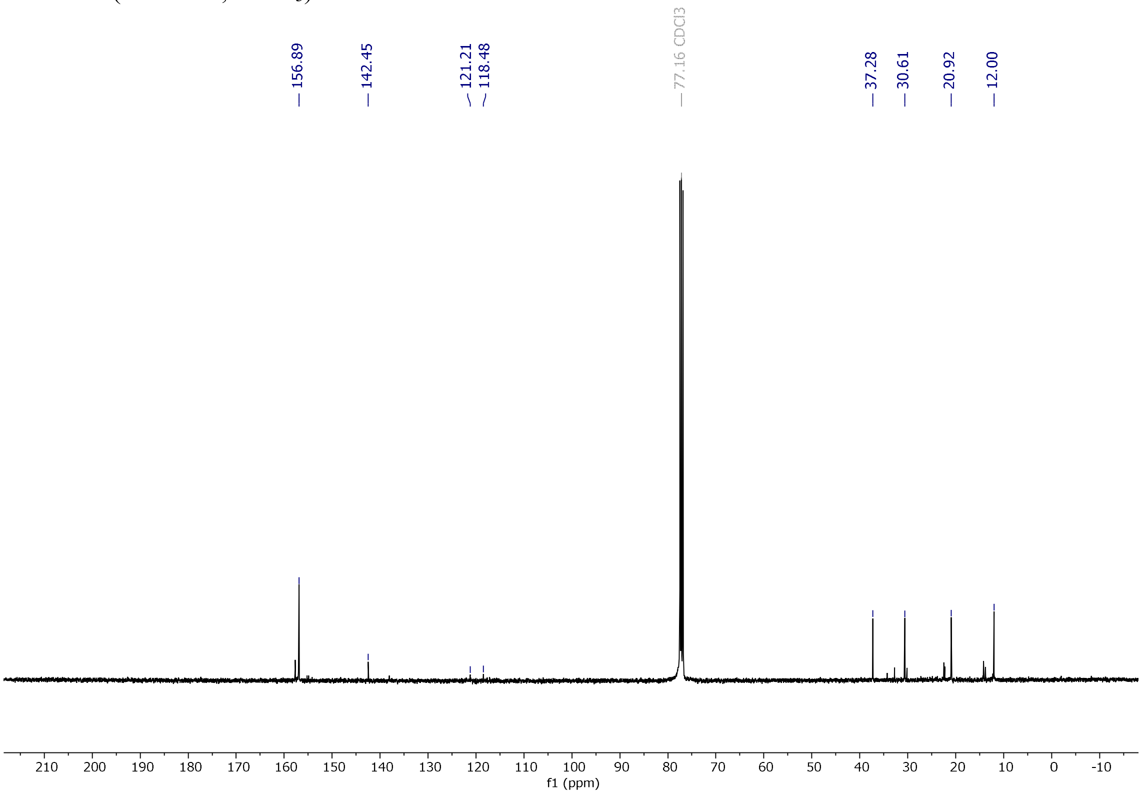

^{19}F NMR (282 MHz, $CDCl_3$) of **8**

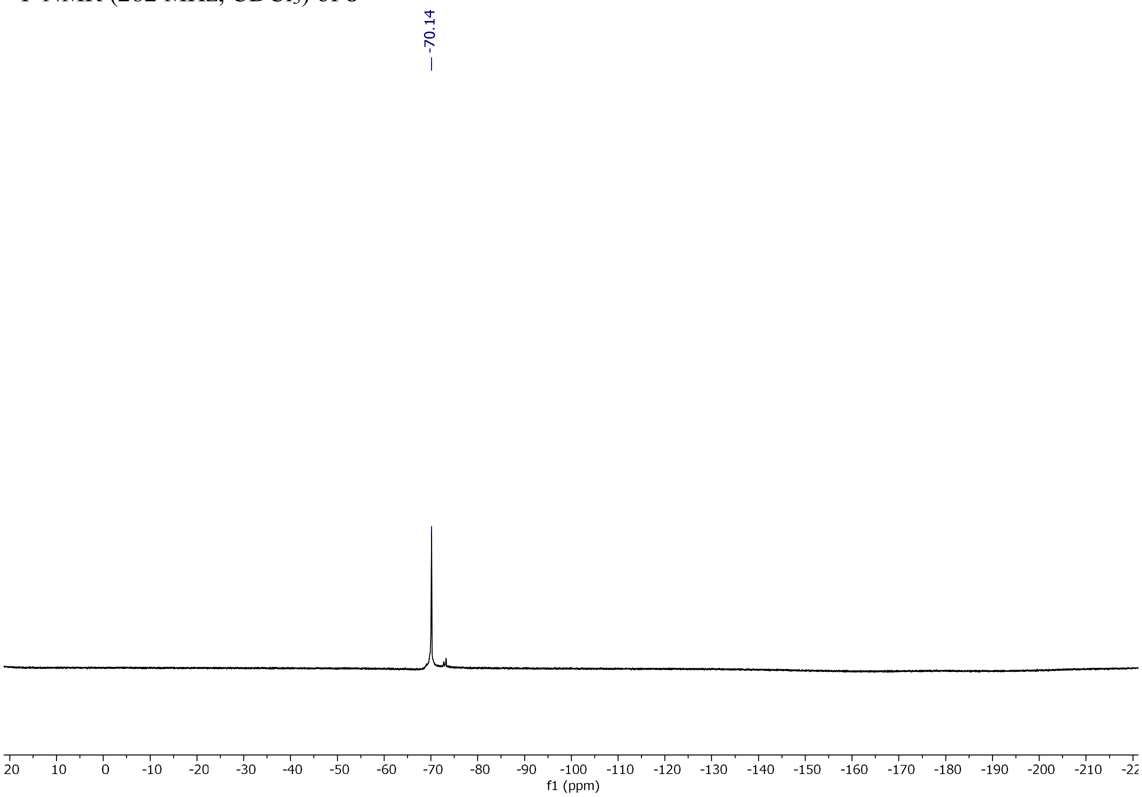

^{1}H NMR (400 MHz, $CDCl_3$) of **9**

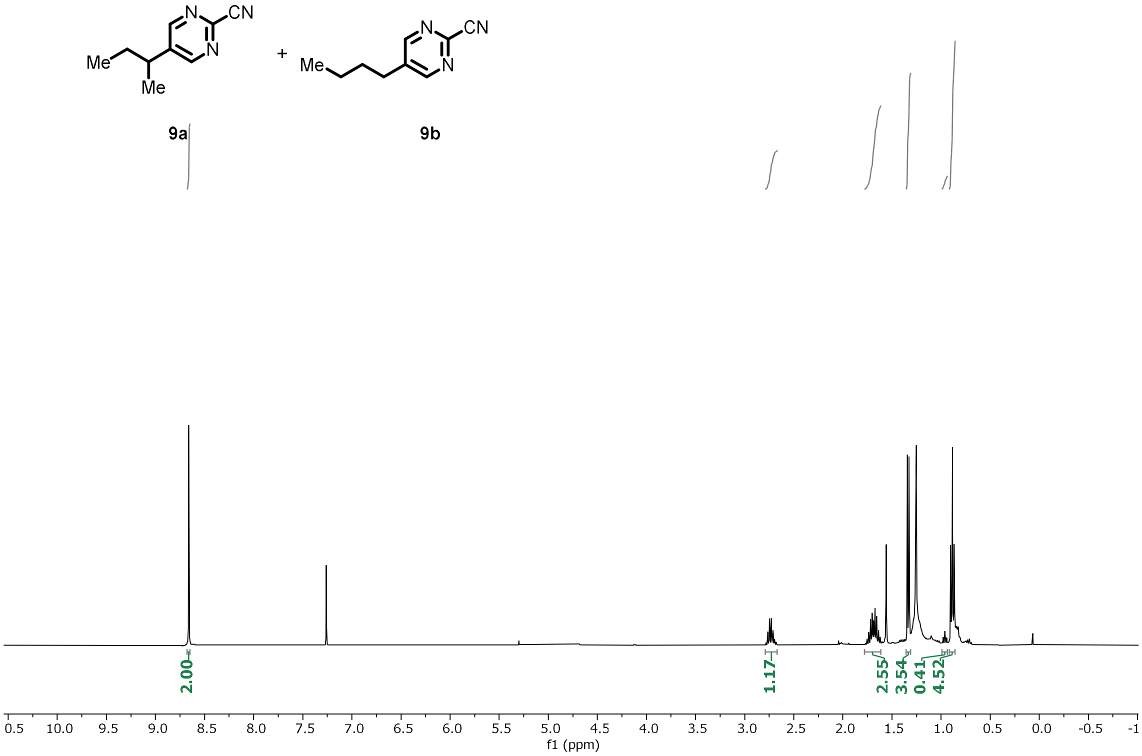

^{13}C NMR (101 MHz, $CDCl_3$) of **9**

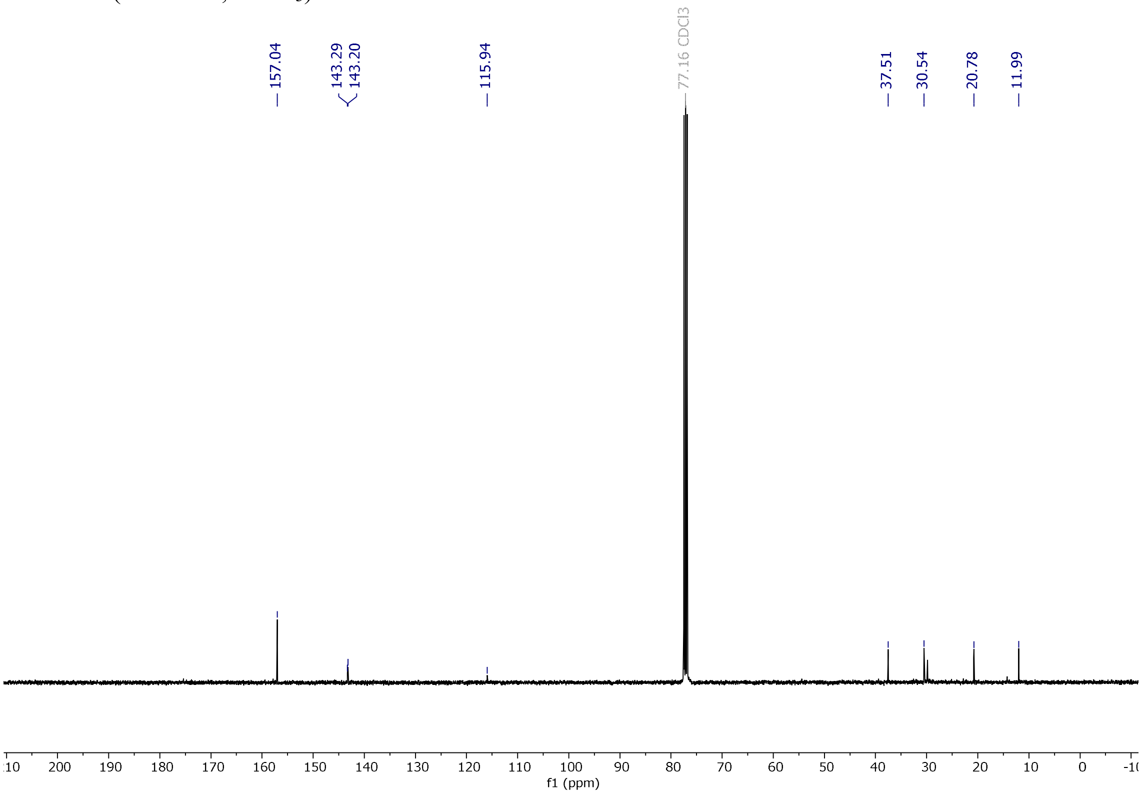

^{1}H NMR (400 MHz, $CDCl_3$) of **10**

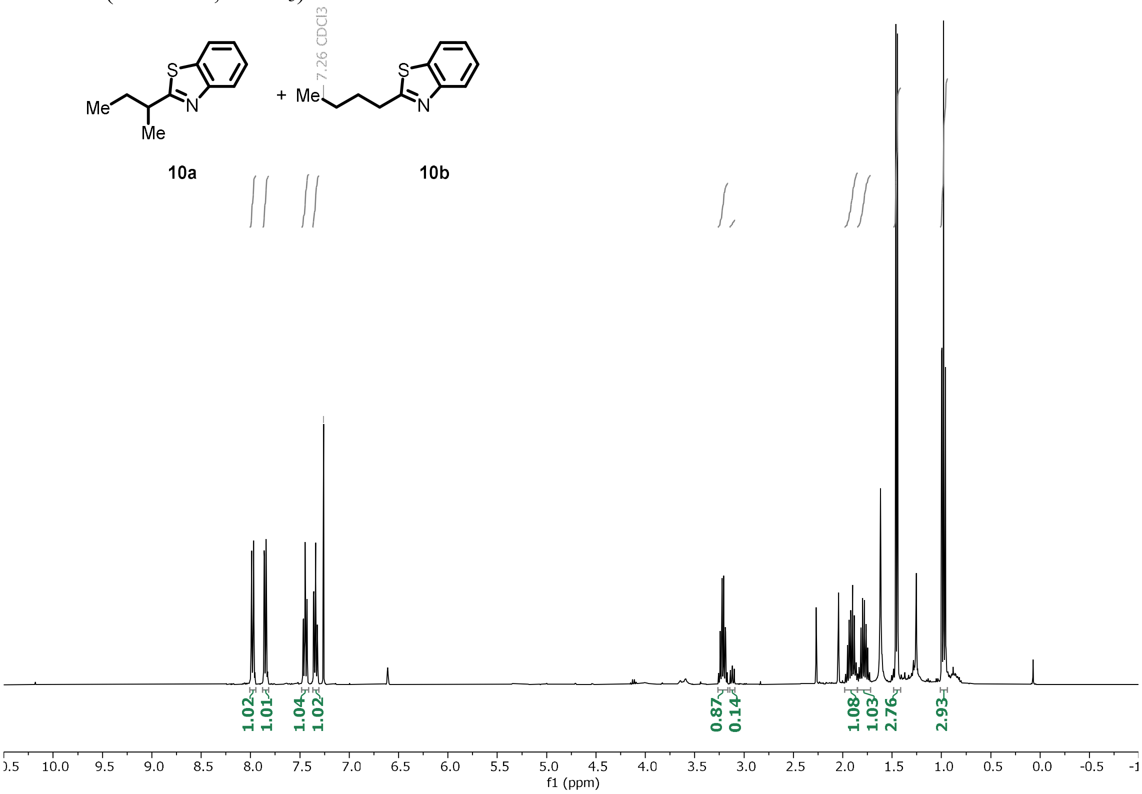

^{13}C NMR (101 MHz, $CDCl_3$) of **10**

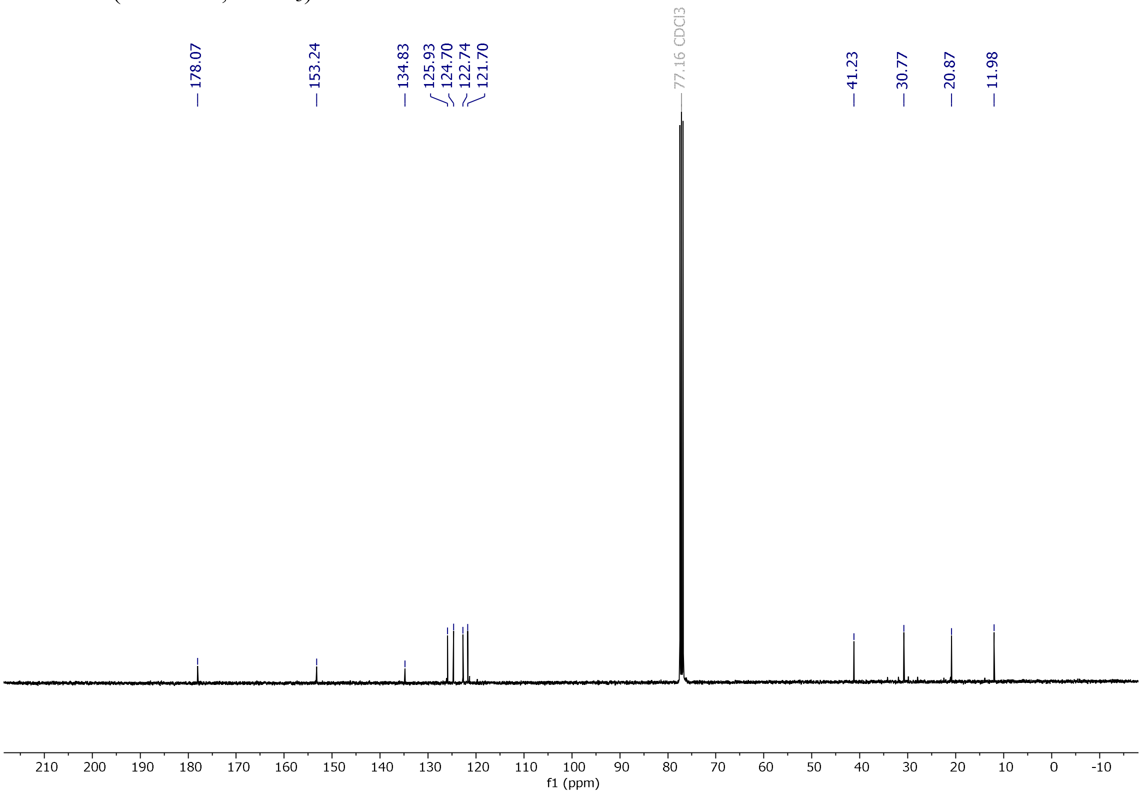

^{1}H NMR (400 MHz, $CDCl_3$) of **11**

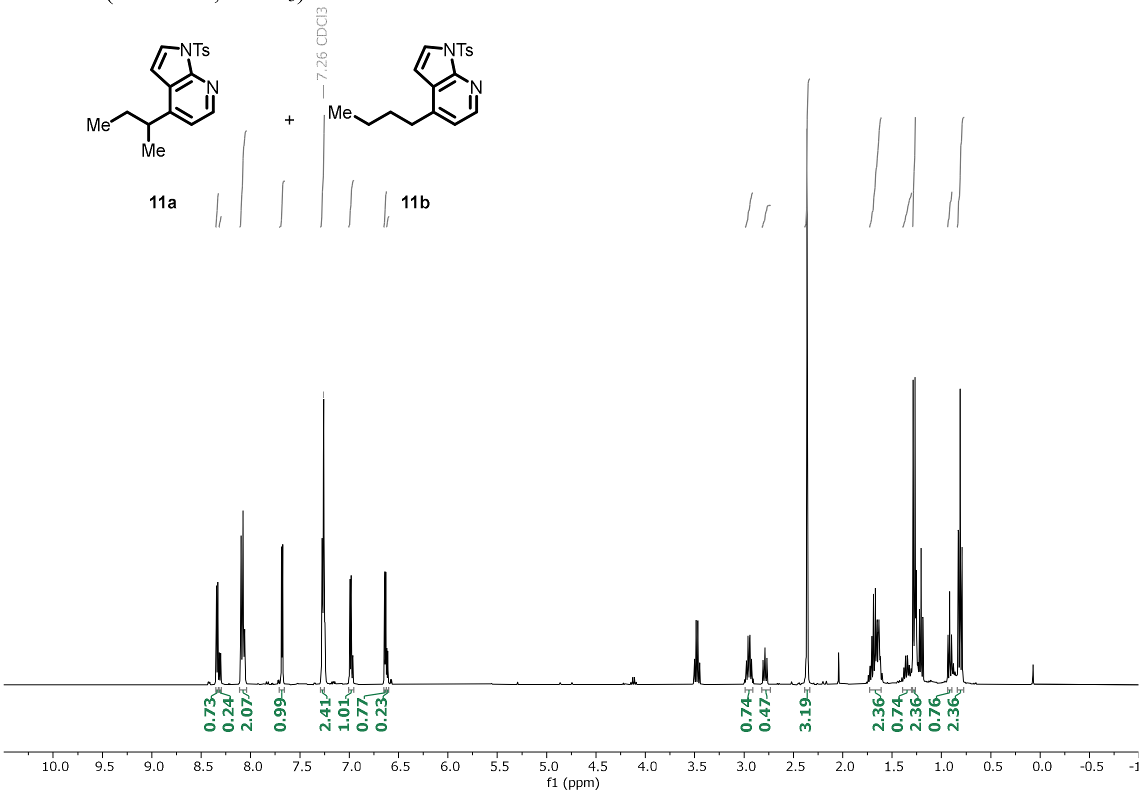

^{13}C NMR (101 MHz, $CDCl_3$) of **11**

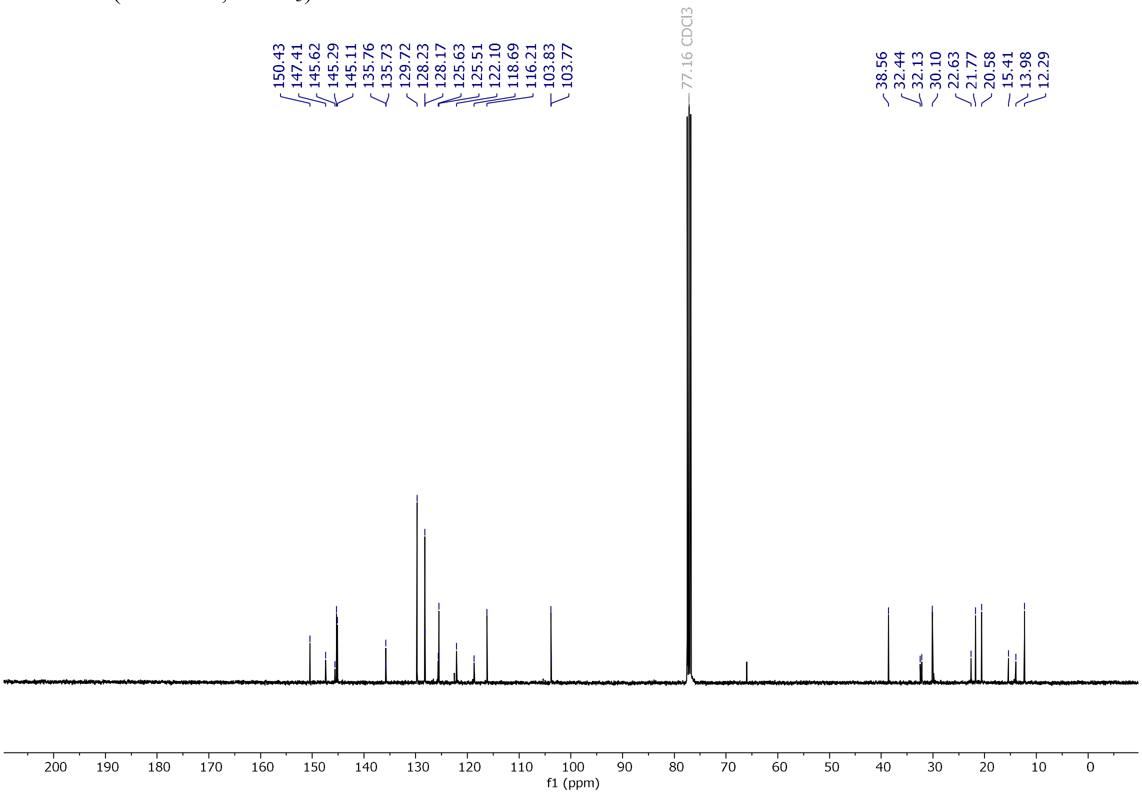

^1H NMR (400 MHz, $CDCl_3$) of **2**

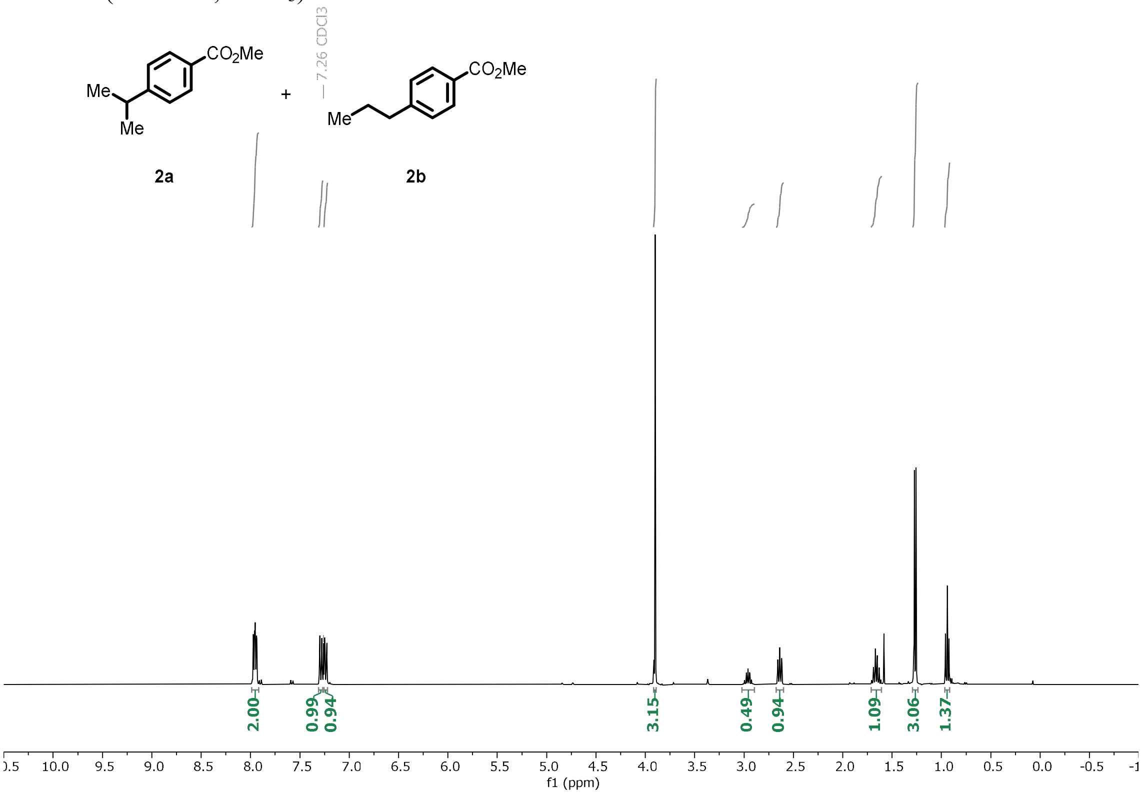

^{13}C NMR (101 MHz, $CDCl_3$) of **2**

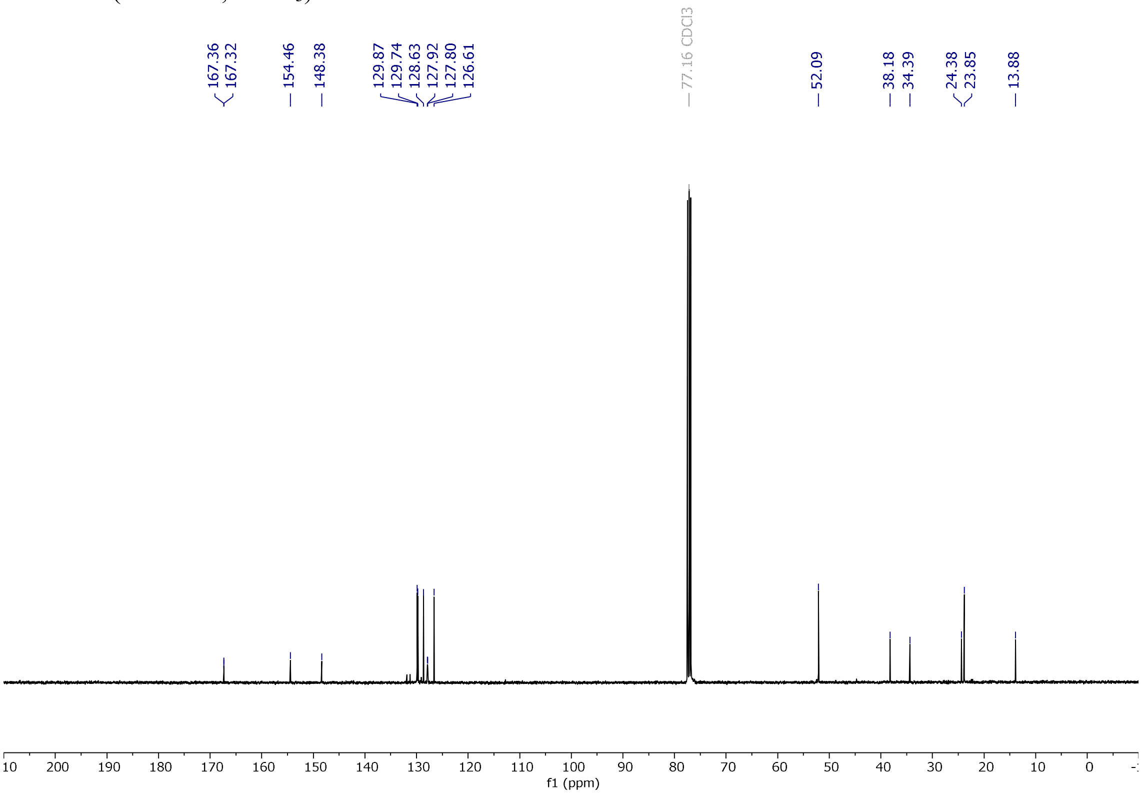

^{1}H NMR (400 MHz, $CDCl_3$) of **12**

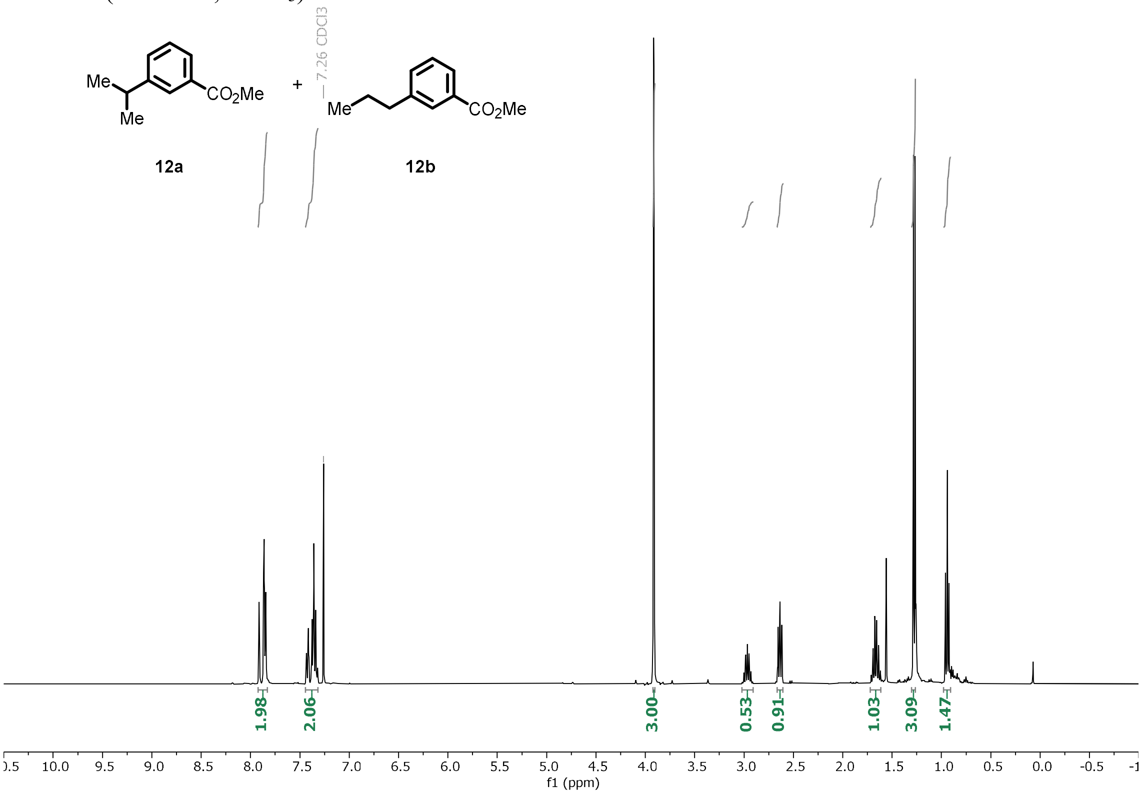

^{13}C NMR (101 MHz, $CDCl_3$) of **12**

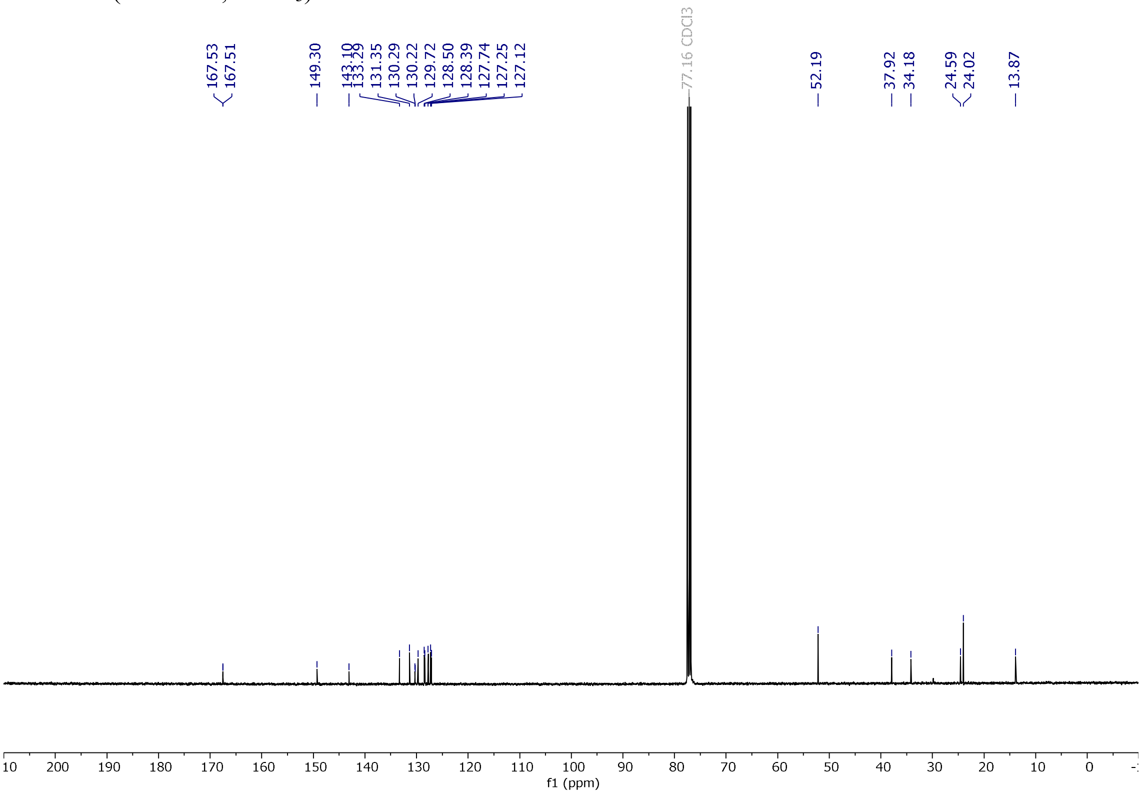

^{1}H NMR (400 MHz, $CDCl_3$) of **13**

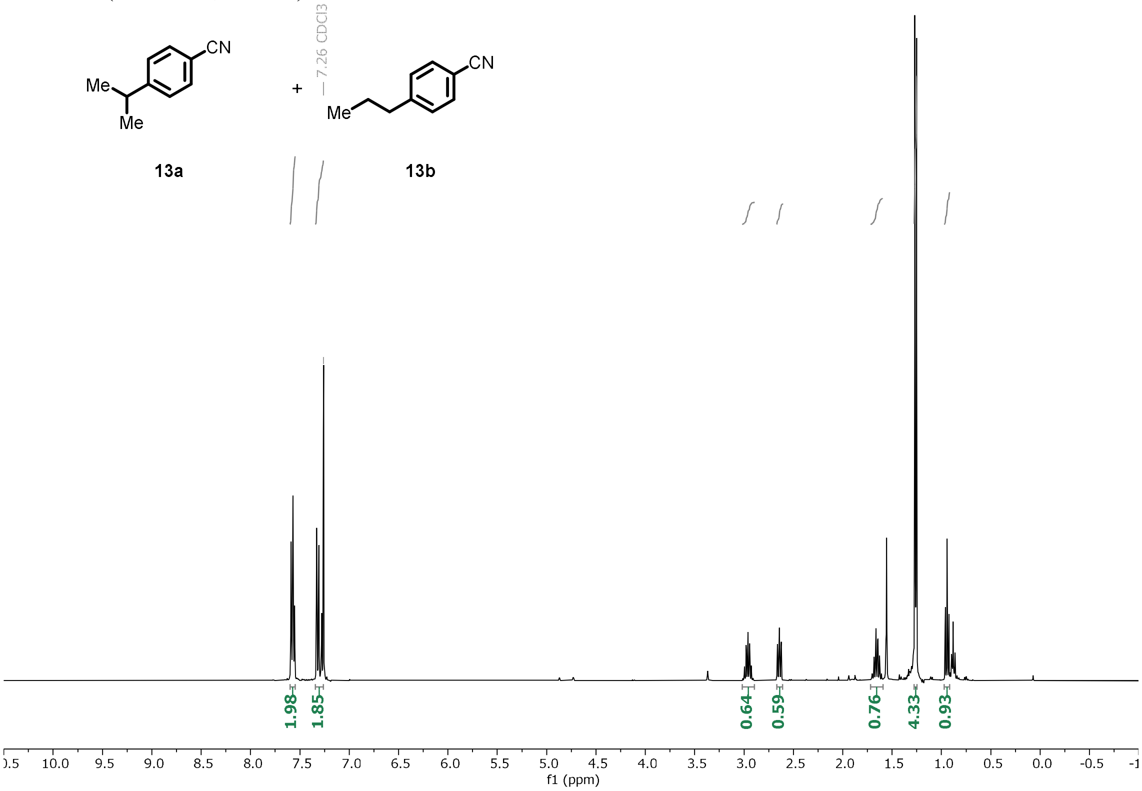

^{13}C NMR (101 MHz, $CDCl_3$) of **13**

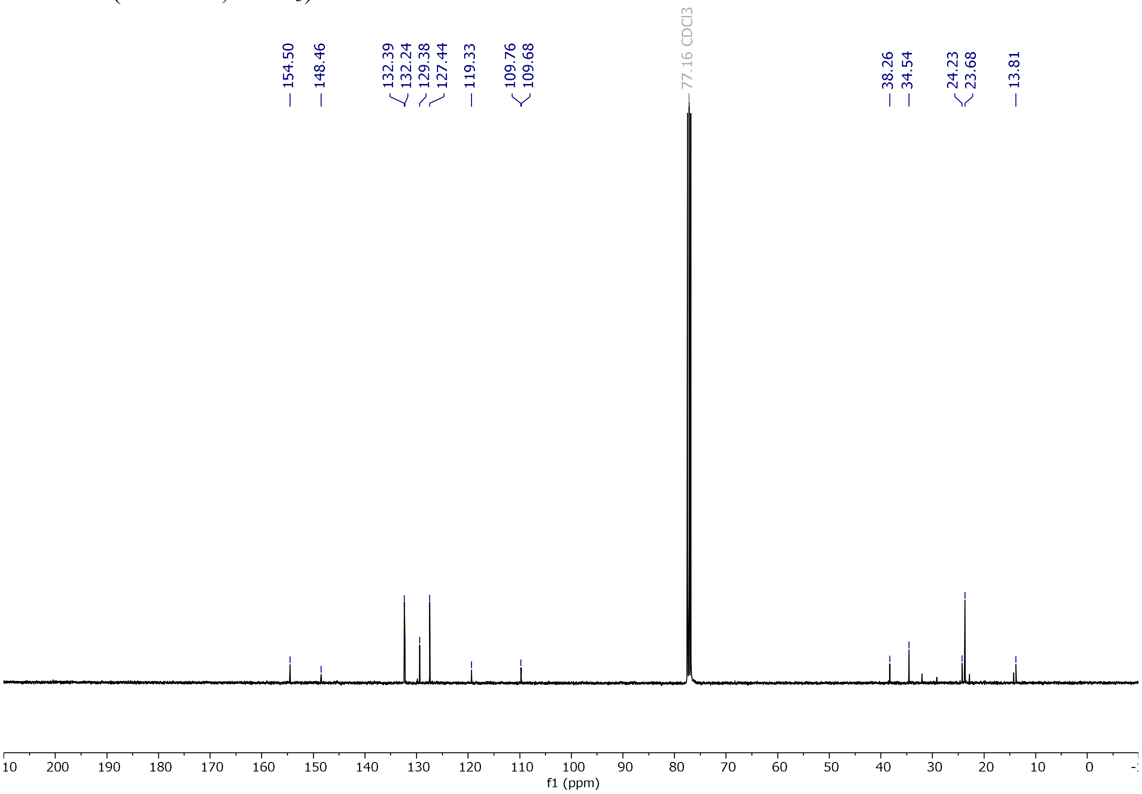

^{1}H NMR (400 MHz, $CDCl_3$) of **14**

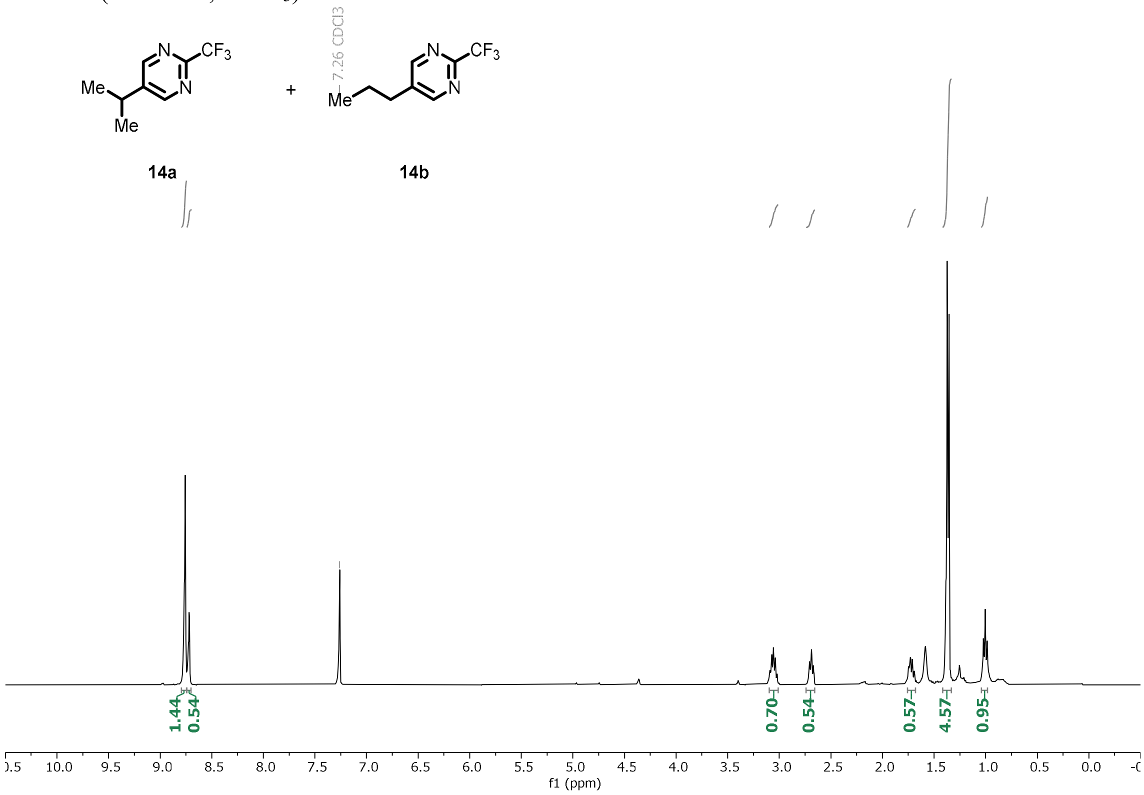

^{13}C NMR (101 MHz, $CDCl_3$) of **14**

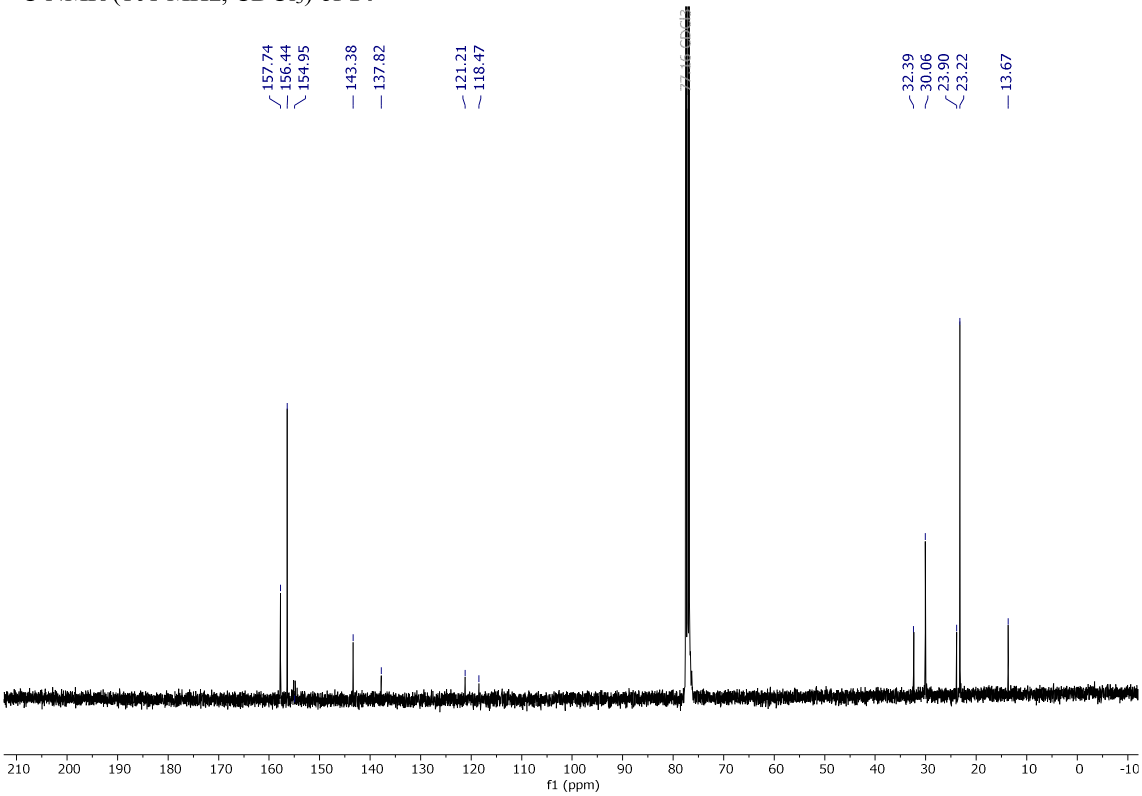

^{19}F NMR (282 MHz, $CDCl_3$) of **14**

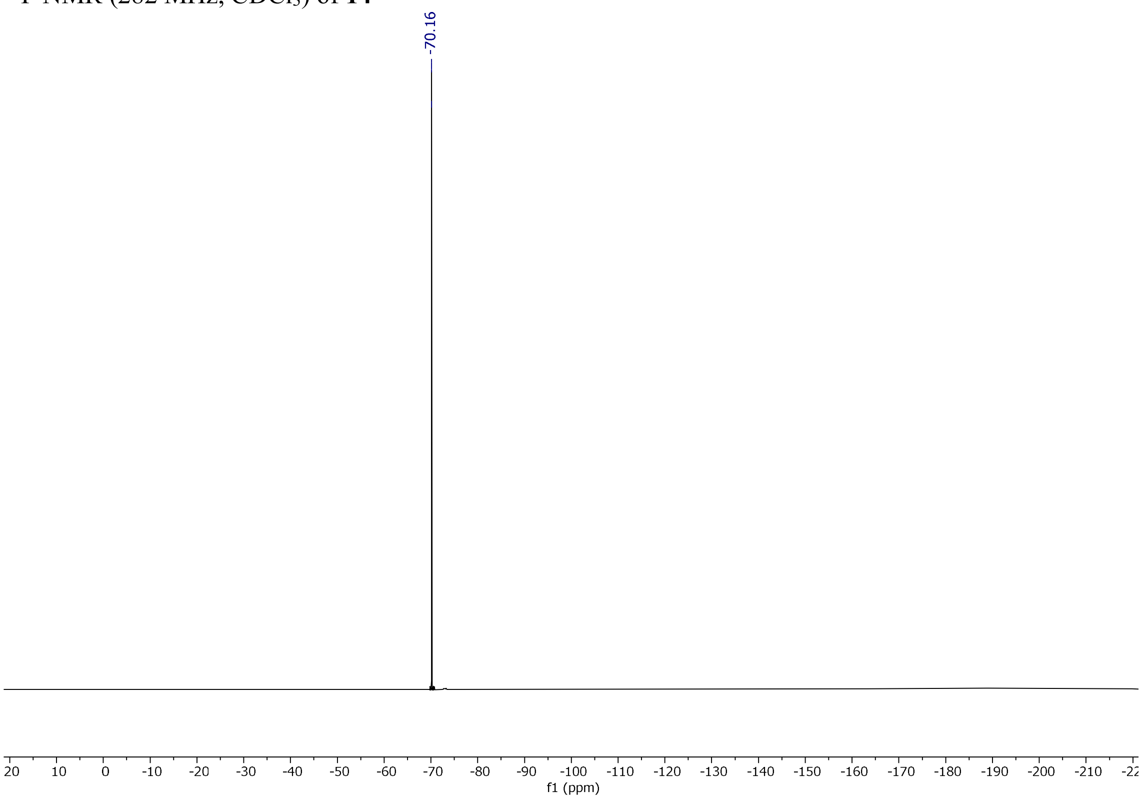

^{1}H NMR (400 MHz, $CDCl_3$) of **15**

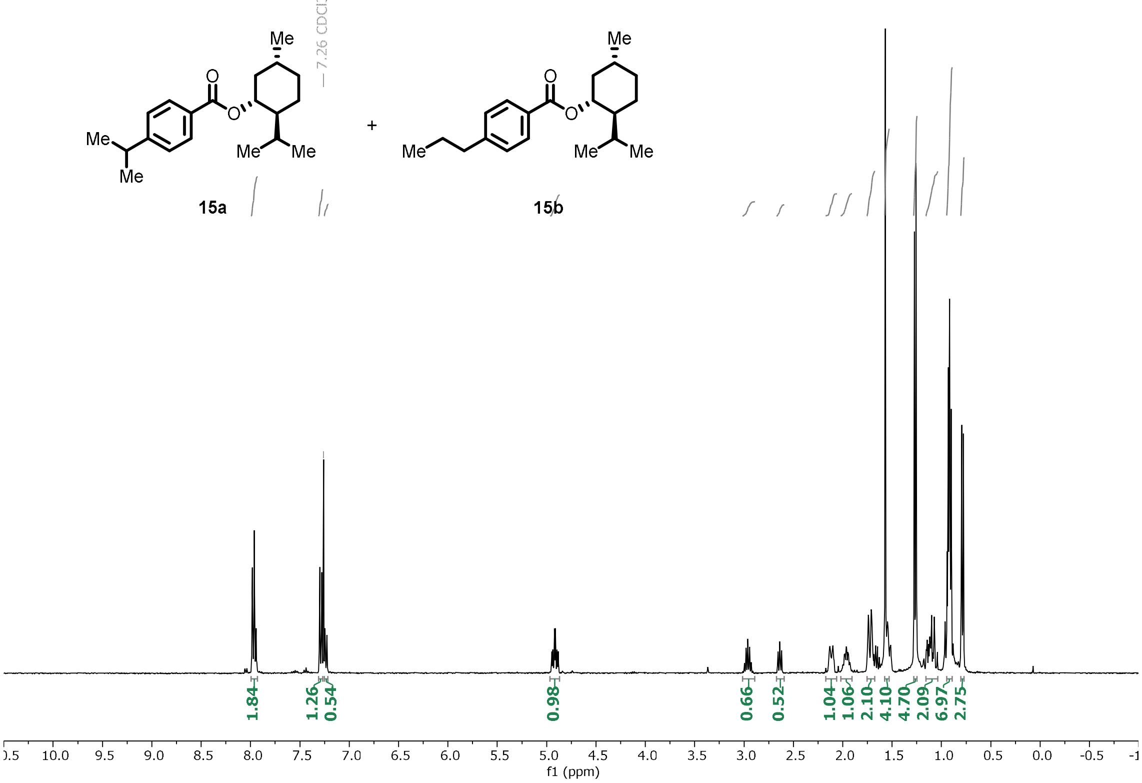

^{13}C NMR (101 MHz, $CDCl_3$) of **15**

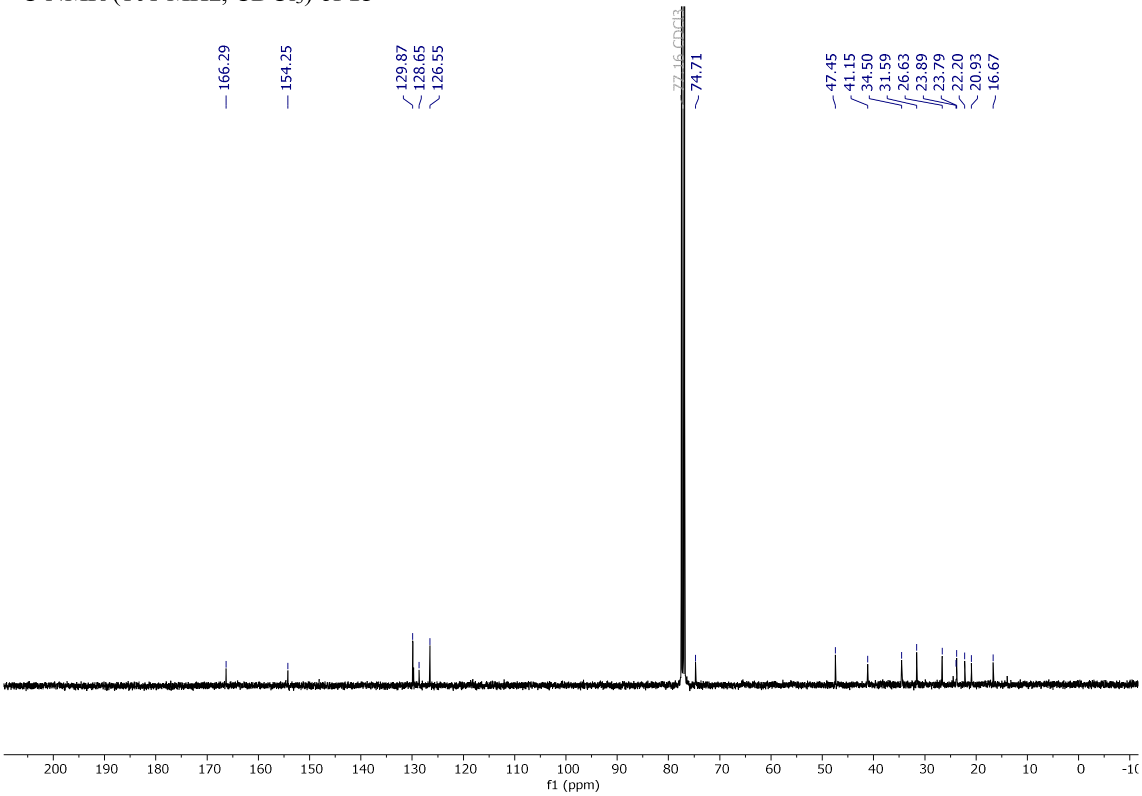

^{1}H NMR (400 MHz, $CDCl_3$) of **16**

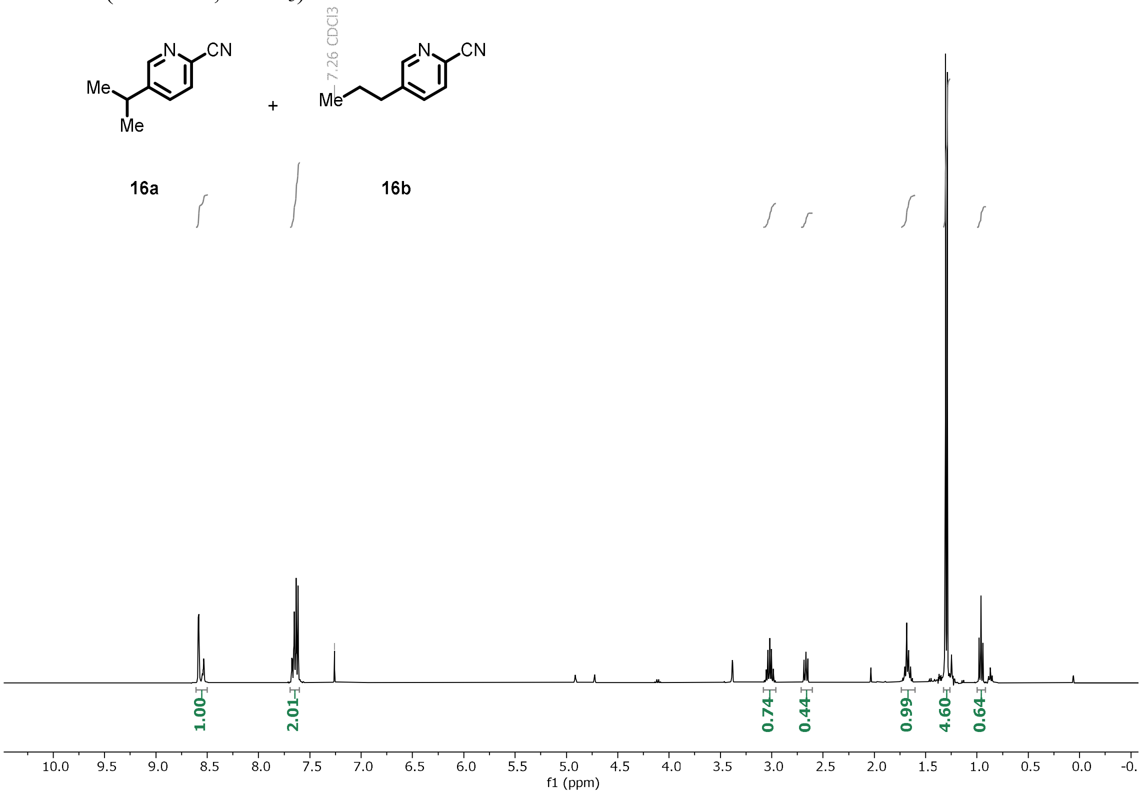

^{13}C NMR (101 MHz, $CDCl_3$) of **16**

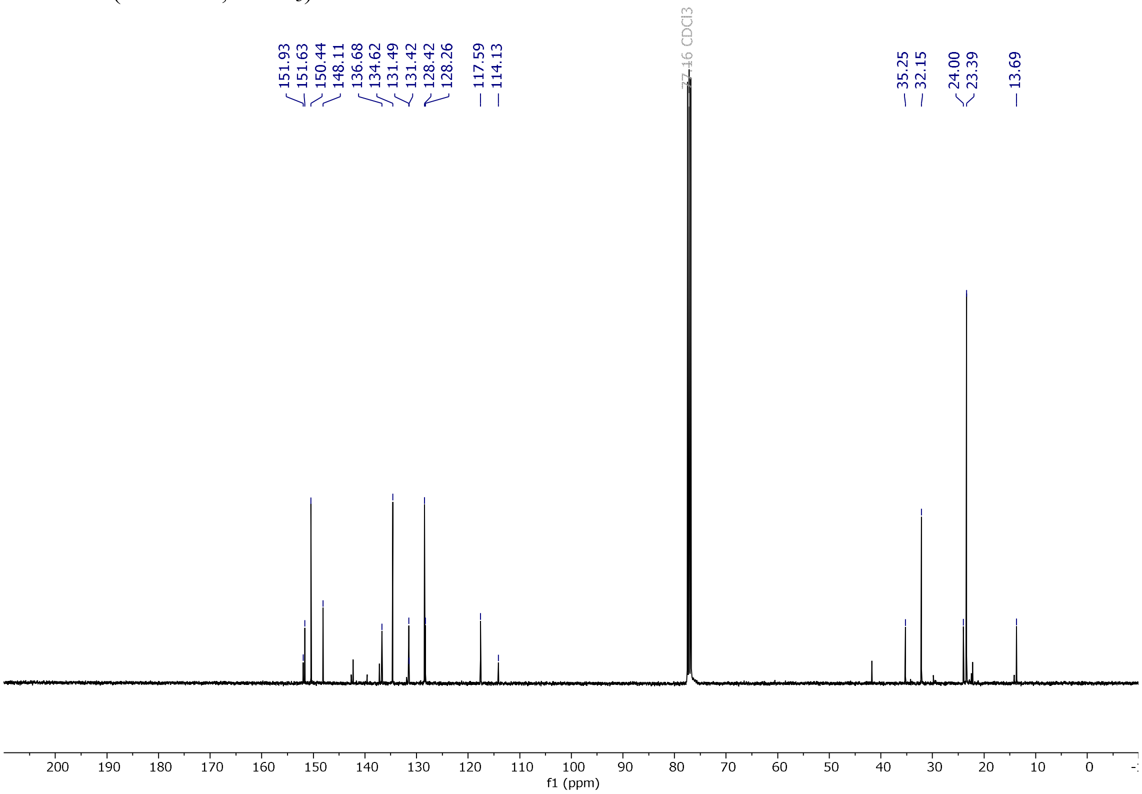

^{1}H NMR (400 MHz, $CDCl_3$) of **17**

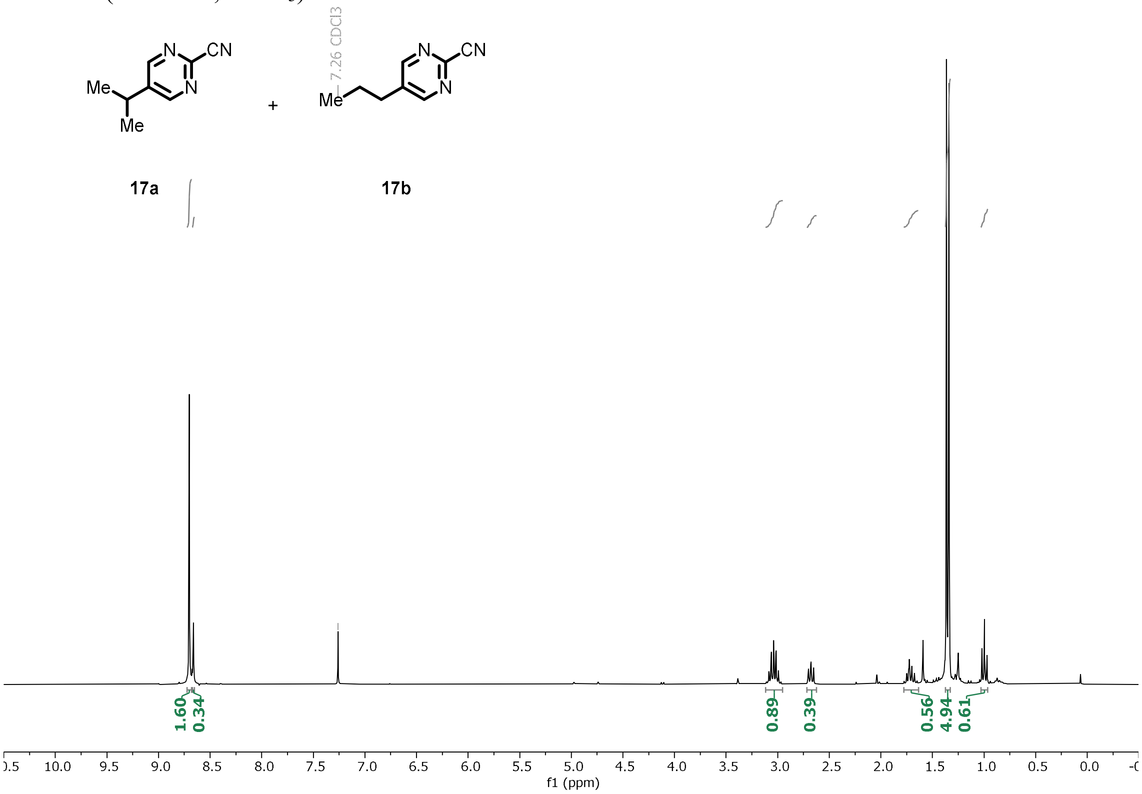

^{13}C NMR (101 MHz, $CDCl_3$) of **17**

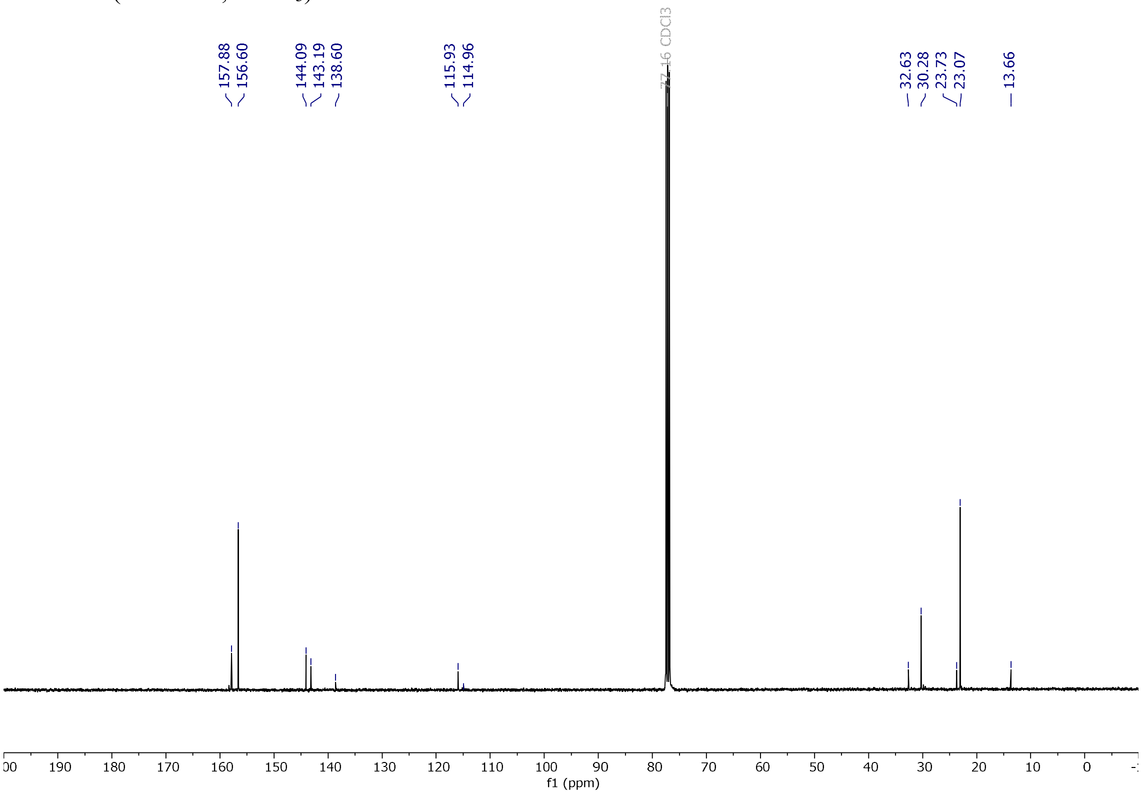

^{1}H NMR (400 MHz, $CDCl_3$) of **18**

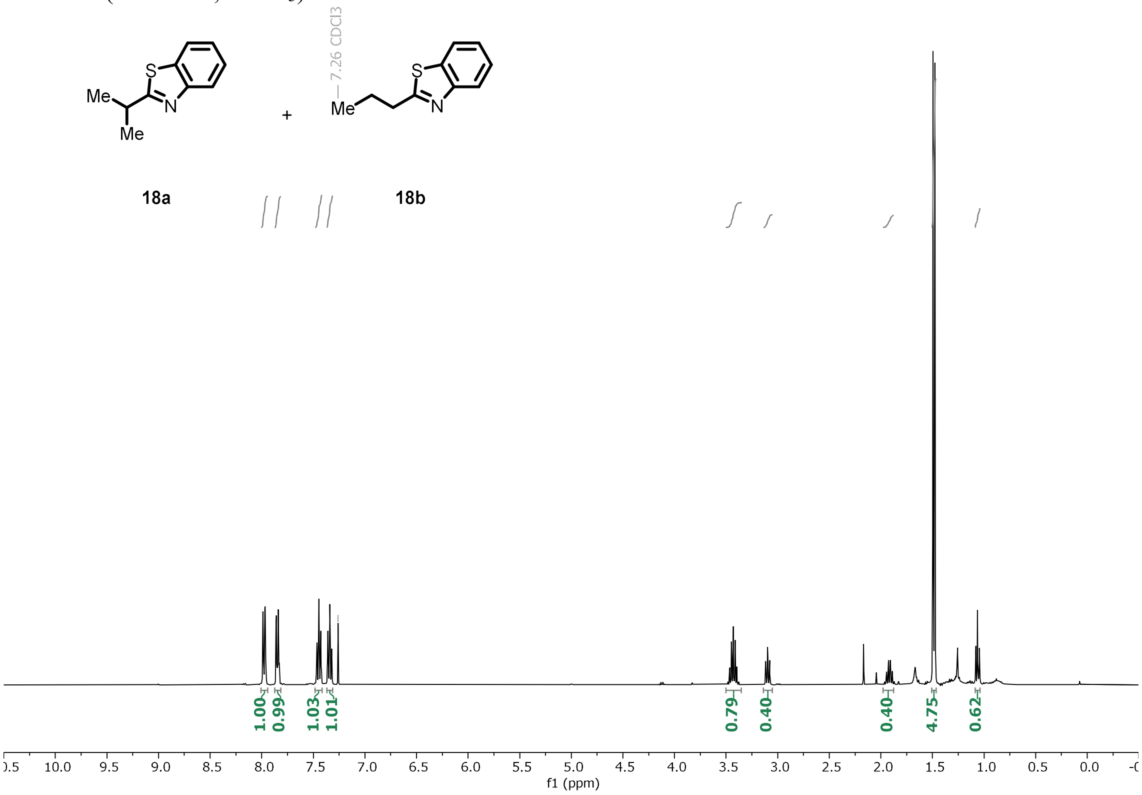

^{13}C NMR (101 MHz, $CDCl_3$) of **18**

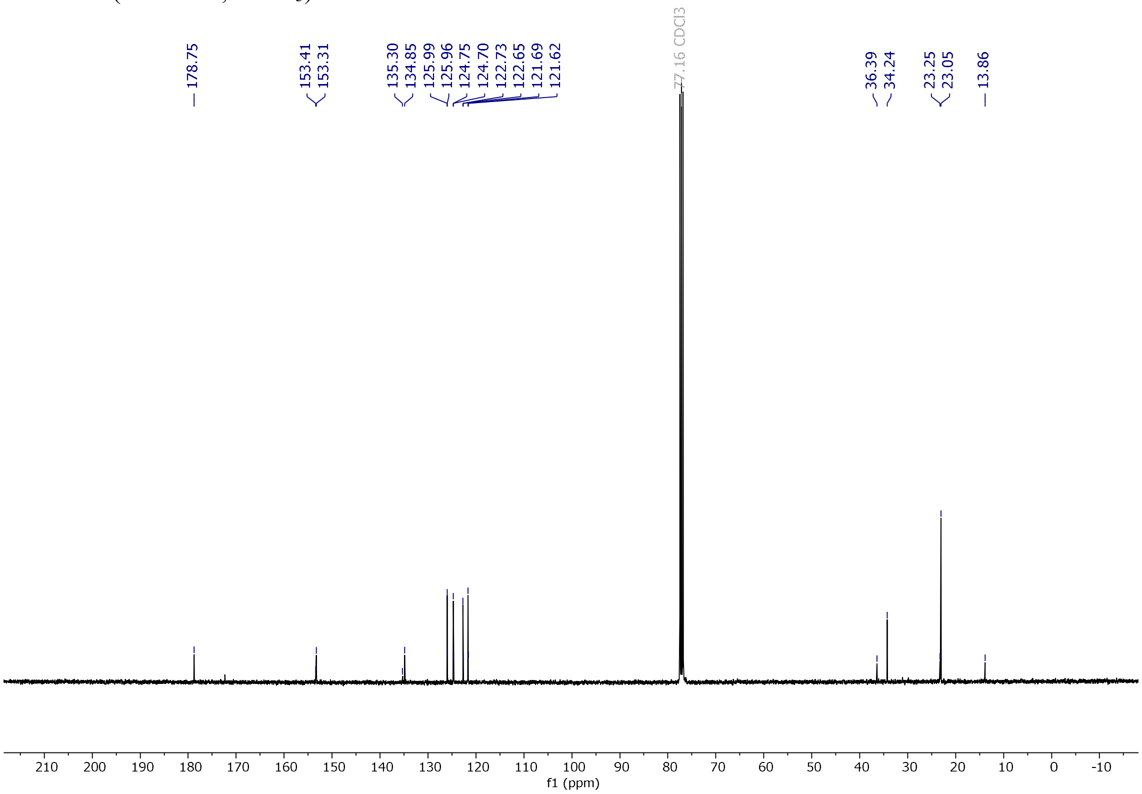

^{1}H NMR (400 MHz, $CDCl_3$) of **19**

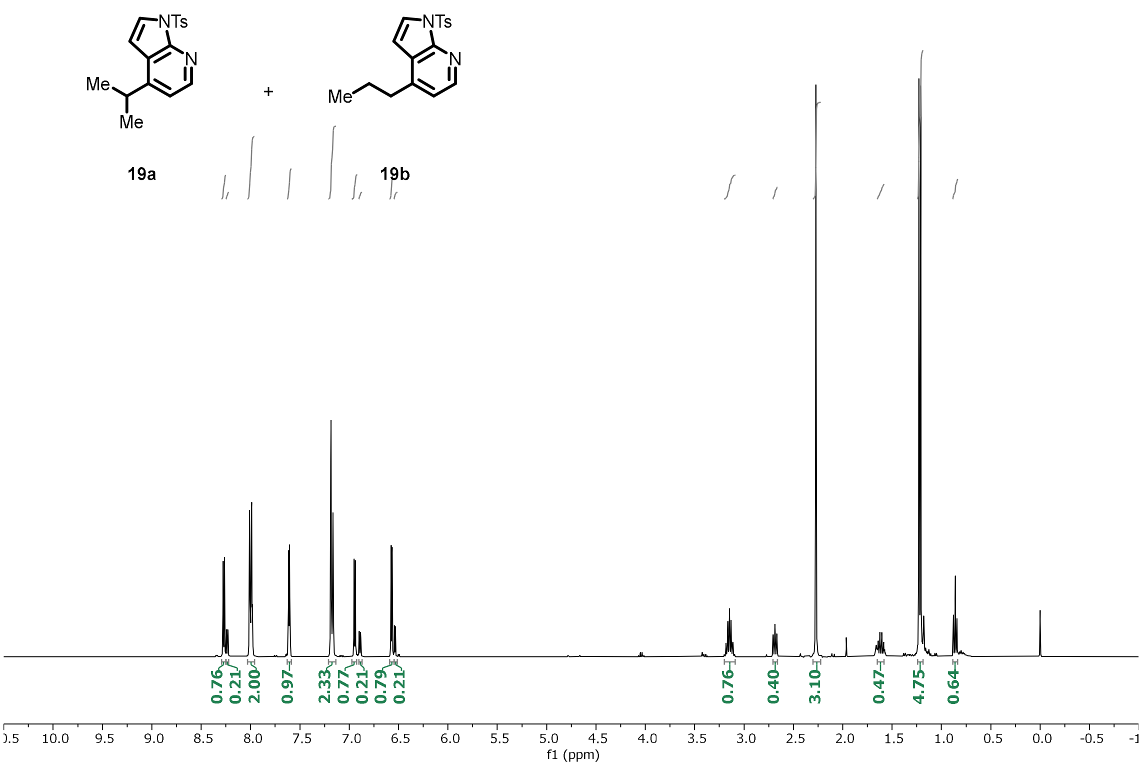

^{13}C NMR (101 MHz, $CDCl_3$) of **19**

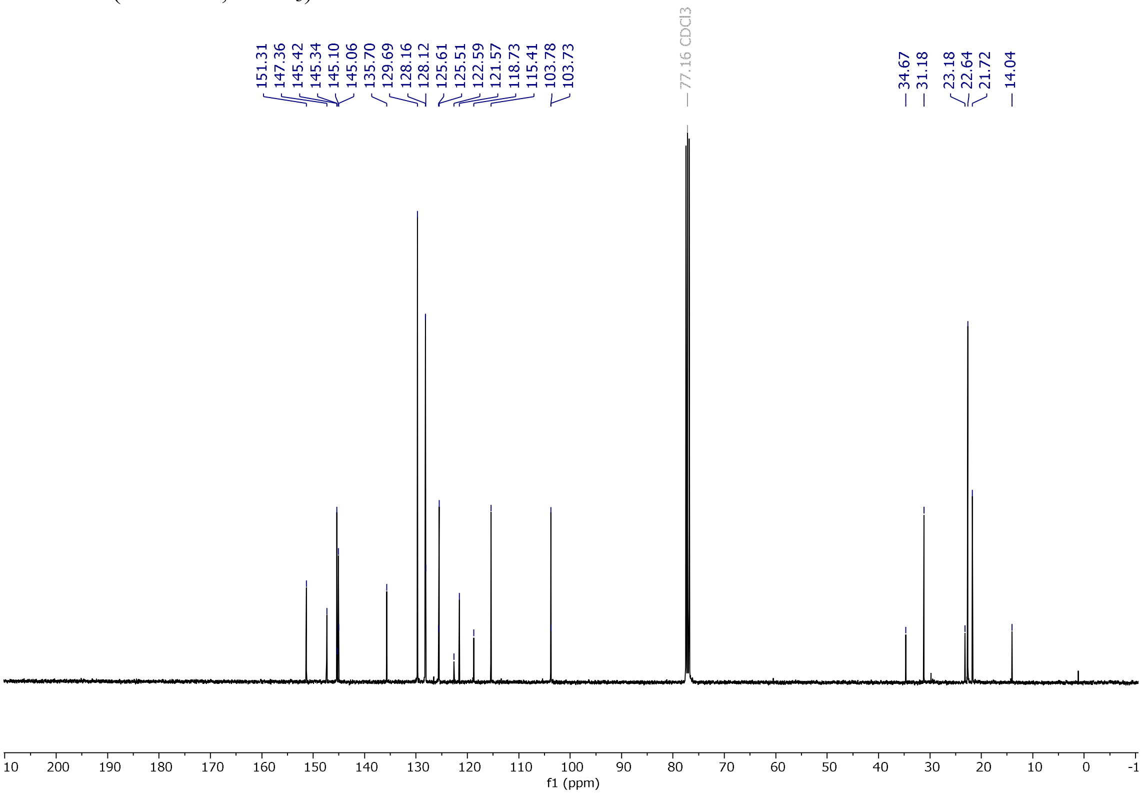

^1H NMR (400 MHz, $CDCl_3$) of **20**

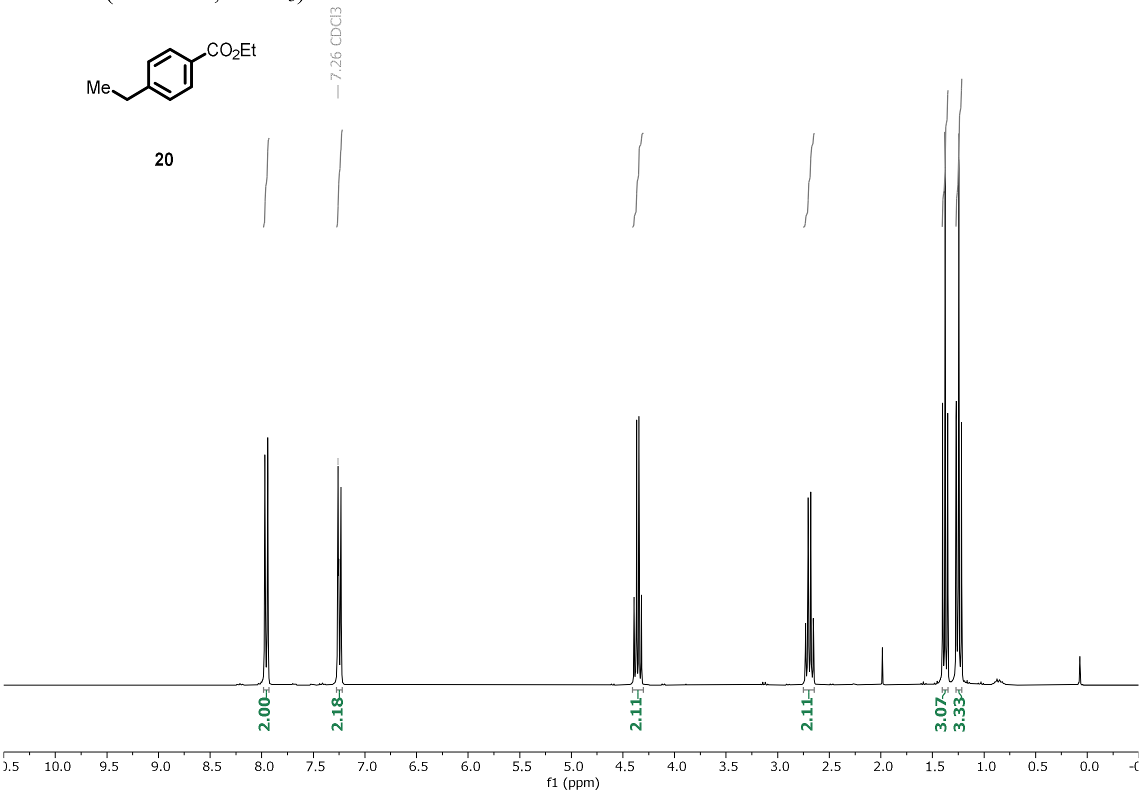

^{13}C NMR (101 MHz, $CDCl_3$) of **20**

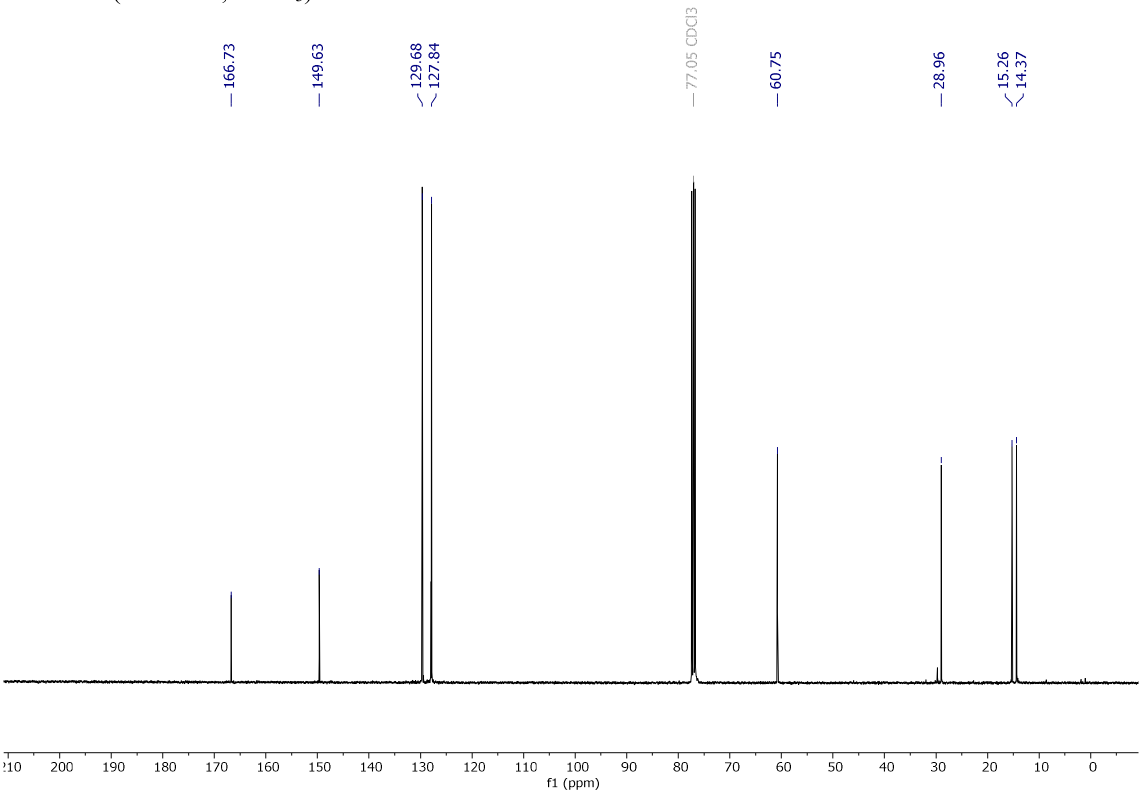

^{1}H NMR (400 MHz, $CDCl_3$) of **21**

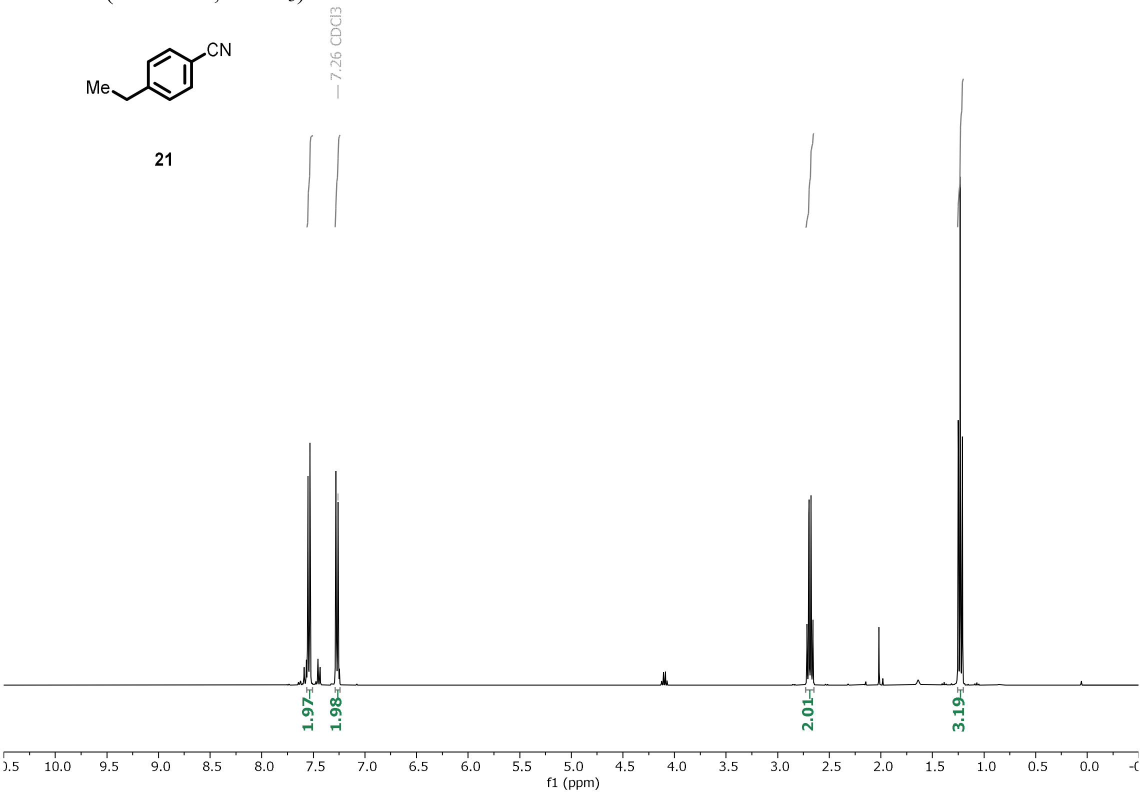

^{13}C NMR (101 MHz, $CDCl_3$) of **21**

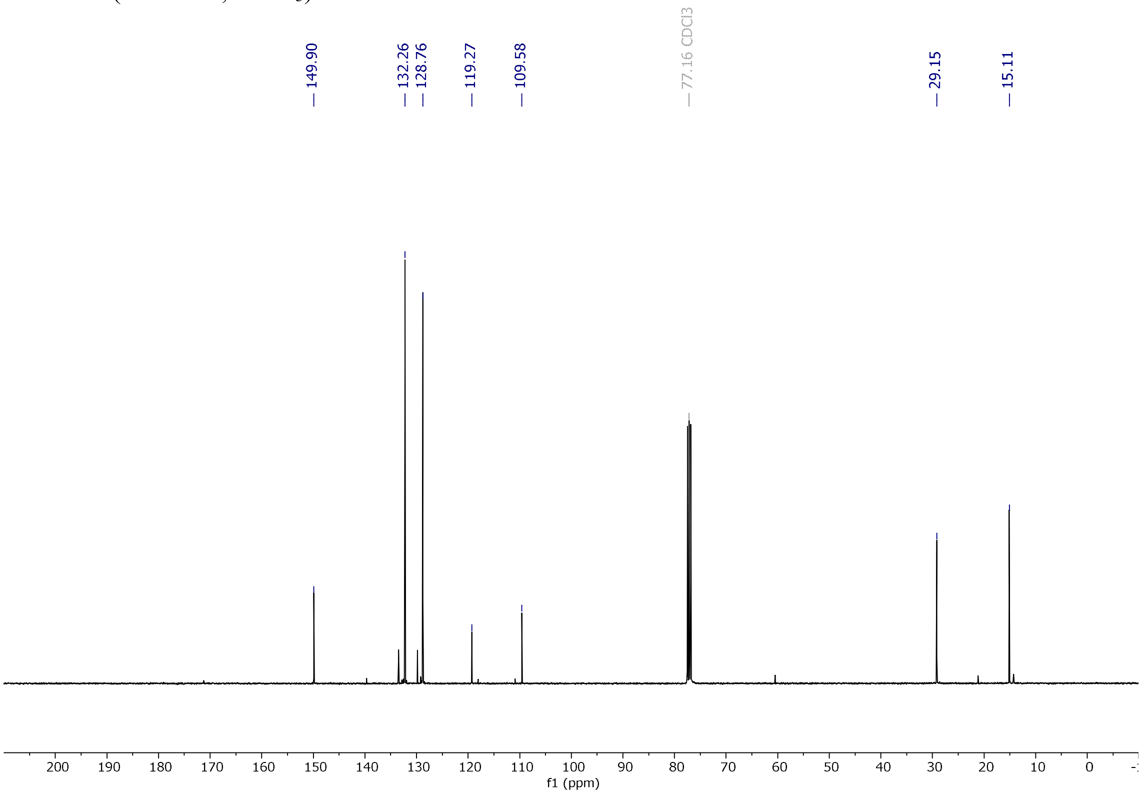

^{1}H NMR (400 MHz, $CDCl_3$) of **22**

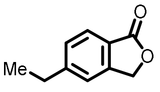

22

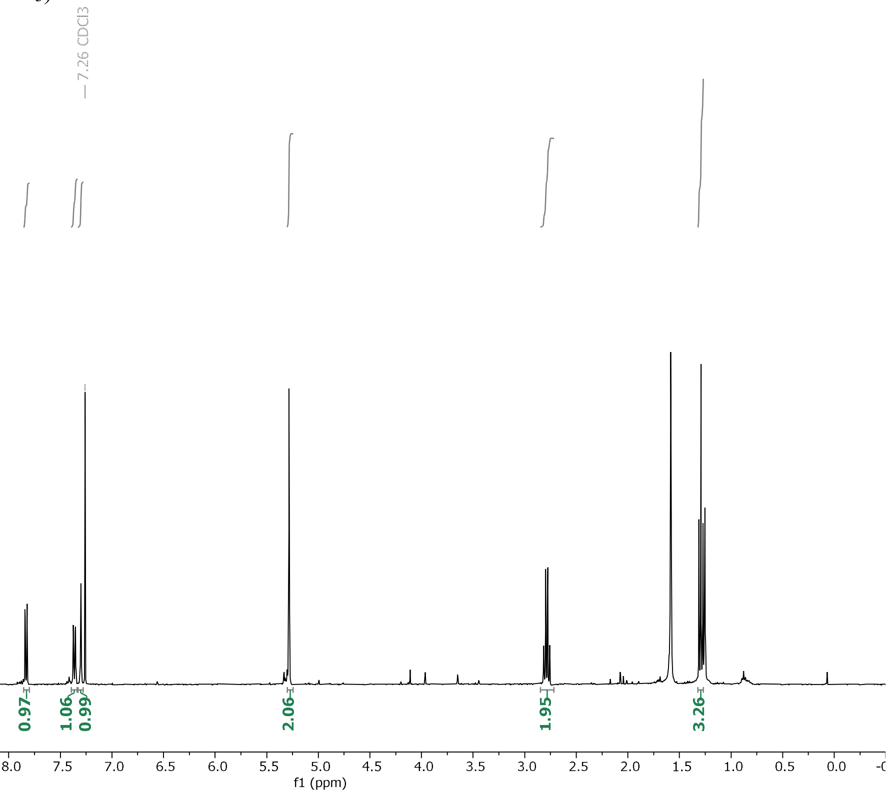

^{13}C NMR (101 MHz, $CDCl_3$) of **22**

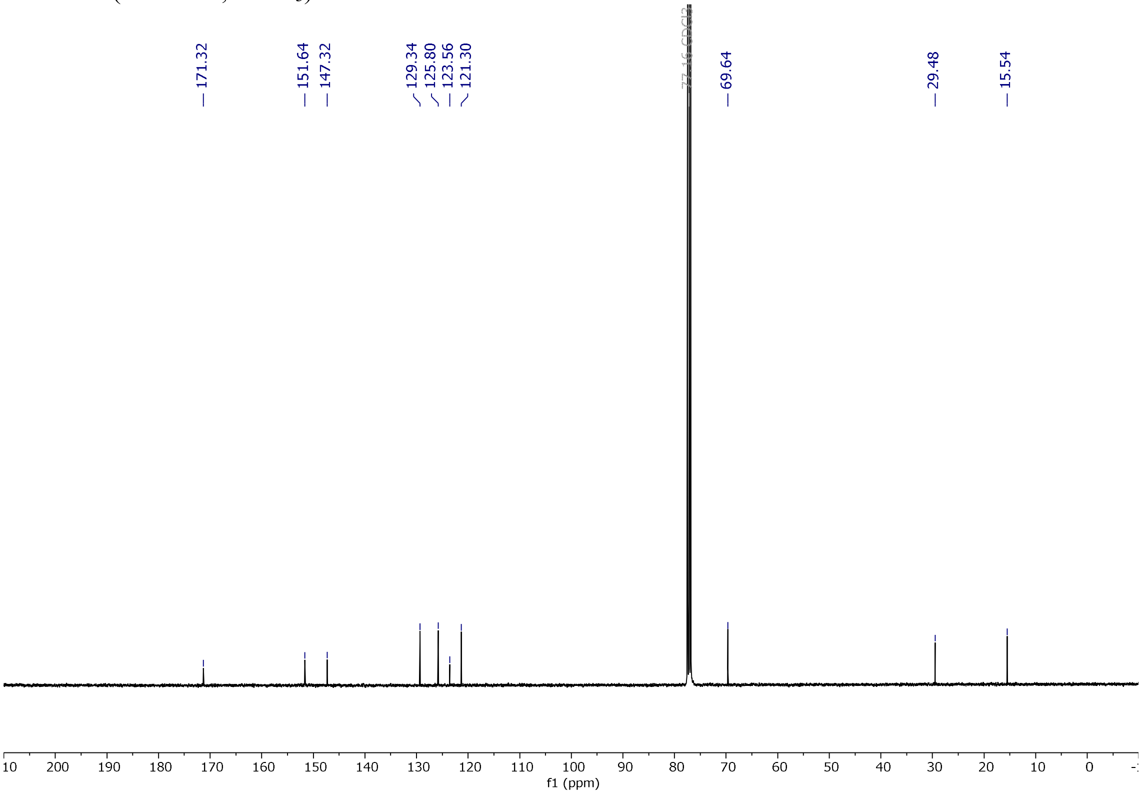

^{1}H NMR (400 MHz, $CDCl_3$) of **23**

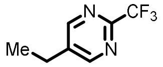

23

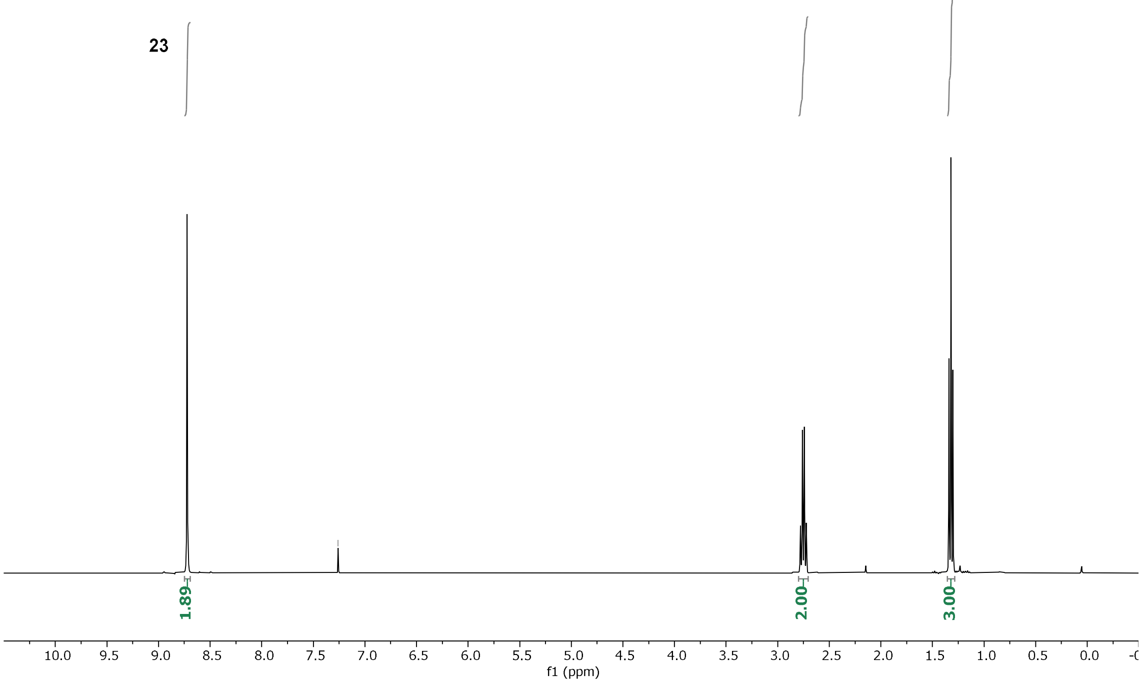

^{13}C NMR (101 MHz, $CDCl_3$) of **23**

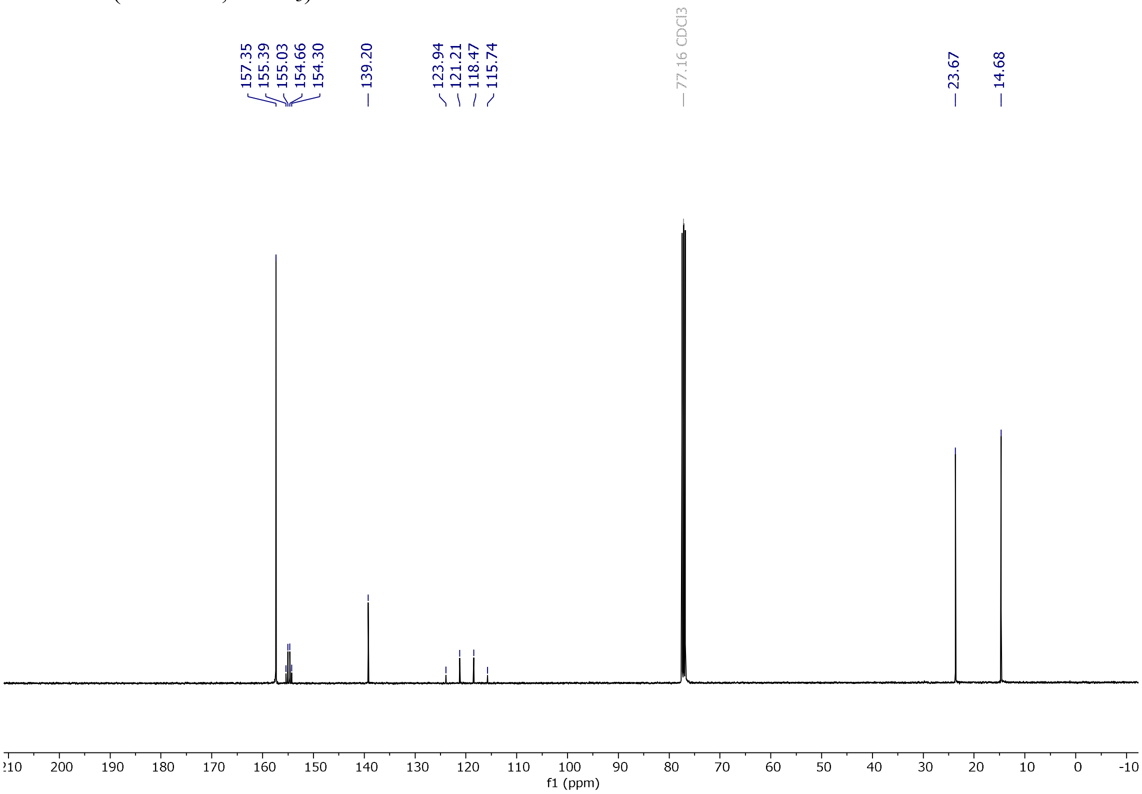

^{19}F NMR (282 MHz, $CDCl_3$) of **23**

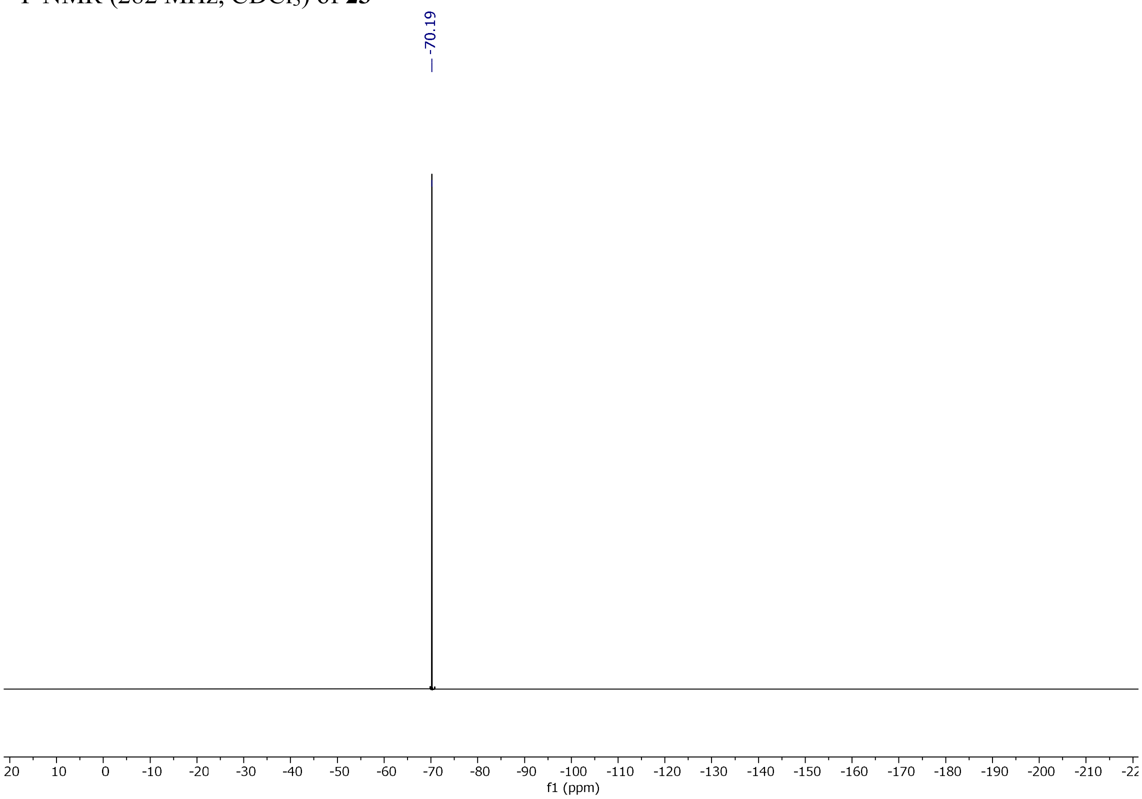

^{1}H NMR (400 MHz, $CDCl_3$) of **24**

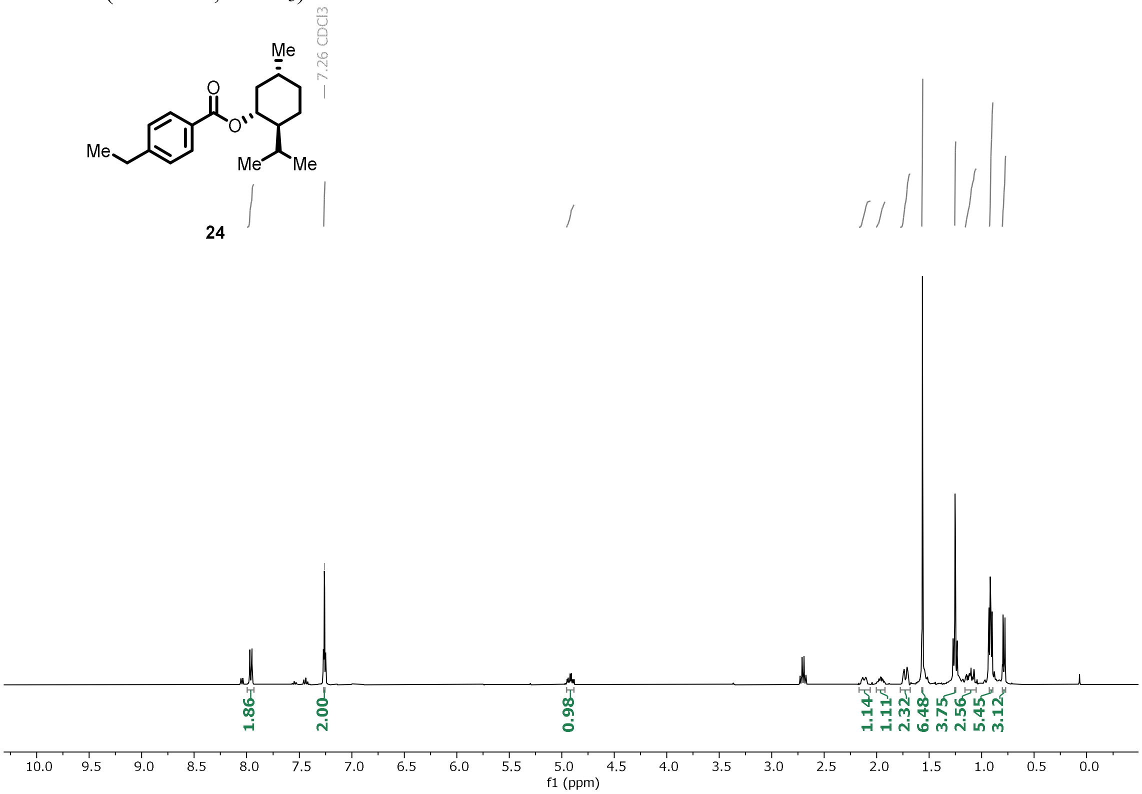

^{13}C NMR (101 MHz, $CDCl_3$) of **24**

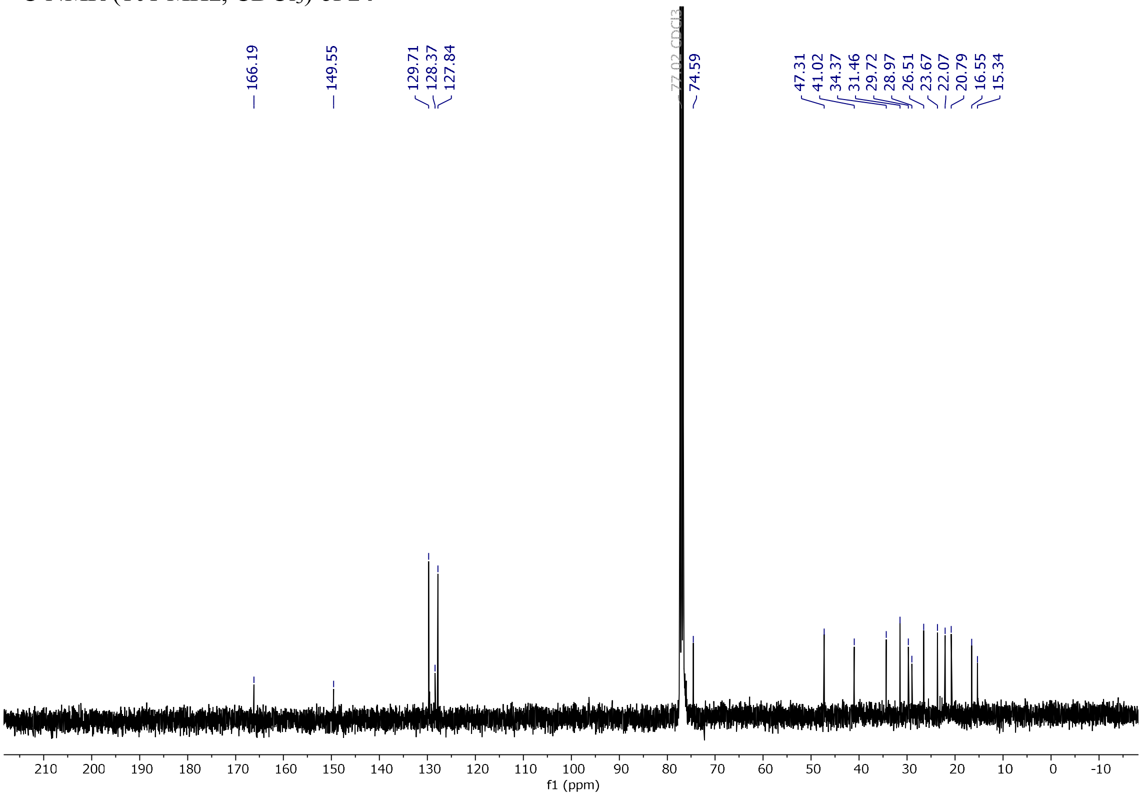

^{1}H NMR (400 MHz, $CDCl_3$) of **25**

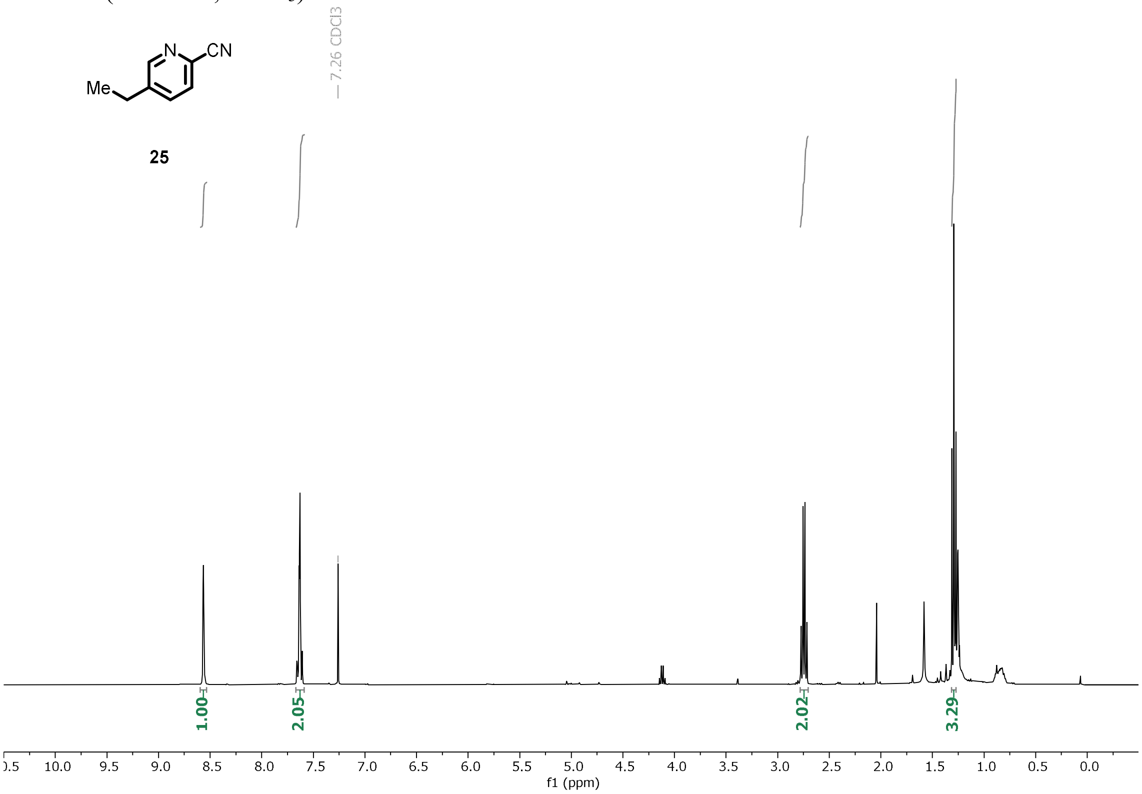

^{13}C NMR (101 MHz, $CDCl_3$) of **25**

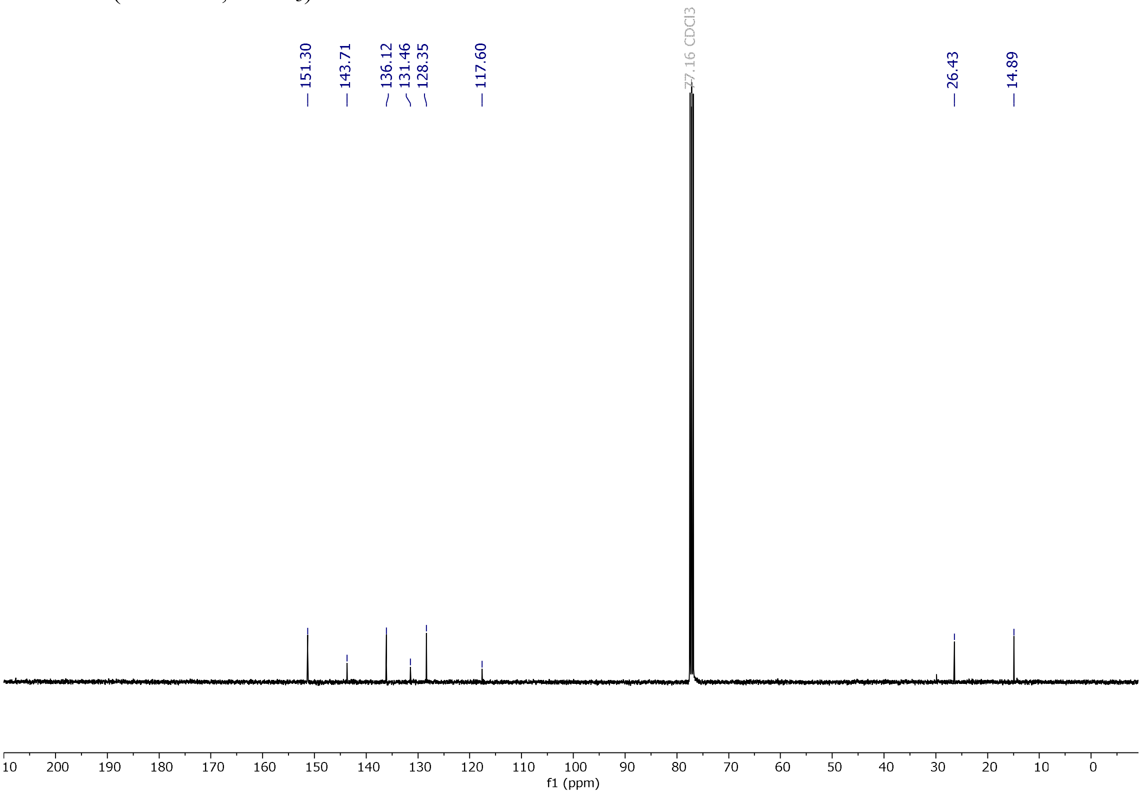

^{1}H NMR (400 MHz, $CDCl_3$) of **26**

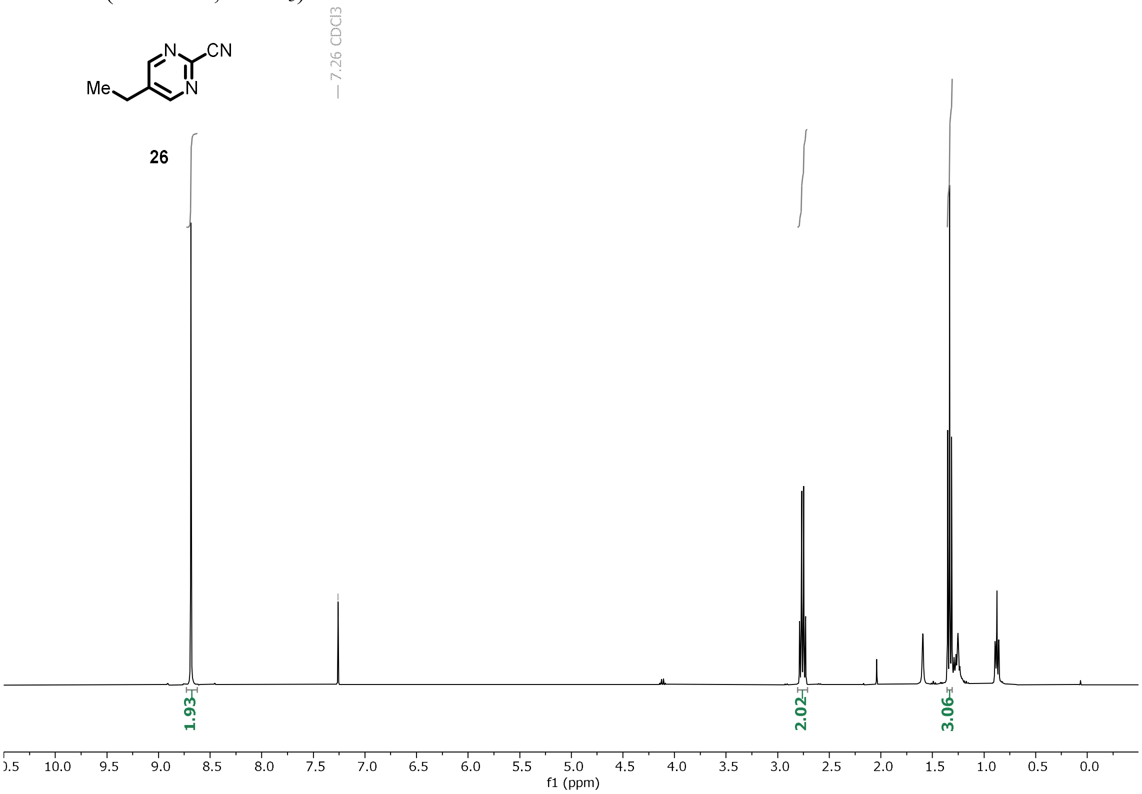

^{13}C NMR (101 MHz, $CDCl_3$) of **26**

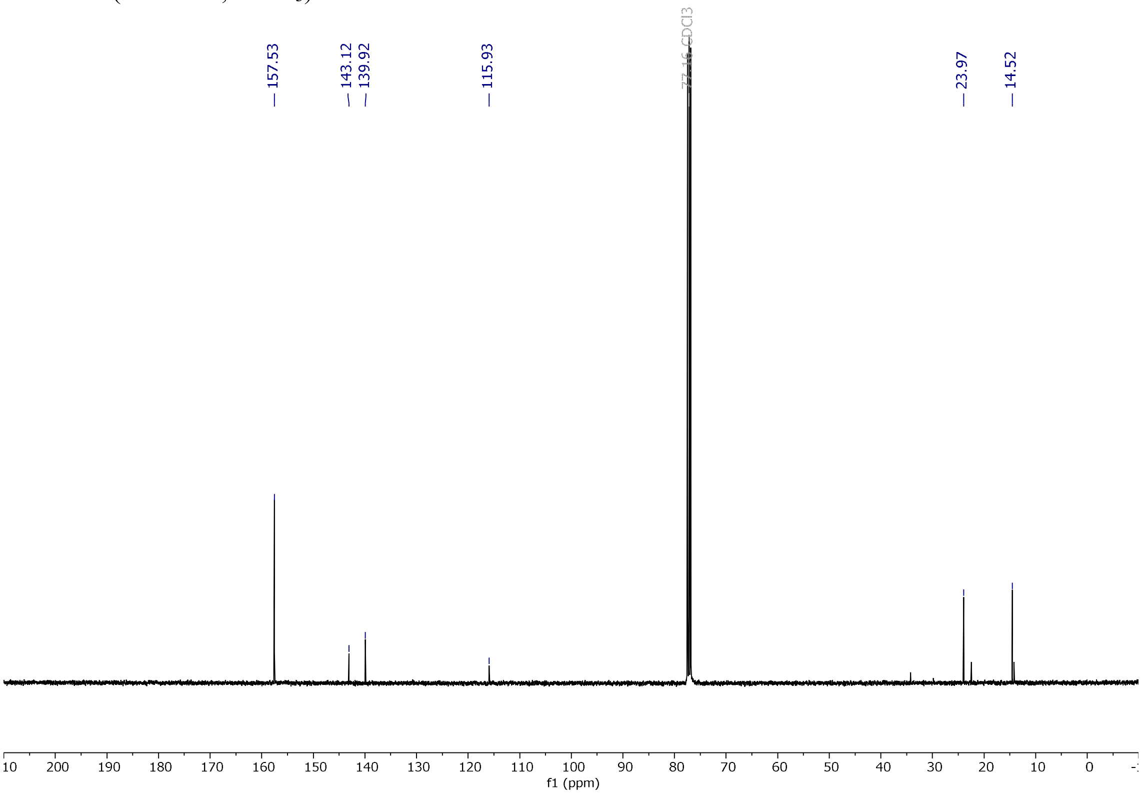

^{1}H NMR (400 MHz, $CDCl_3$) of **27**

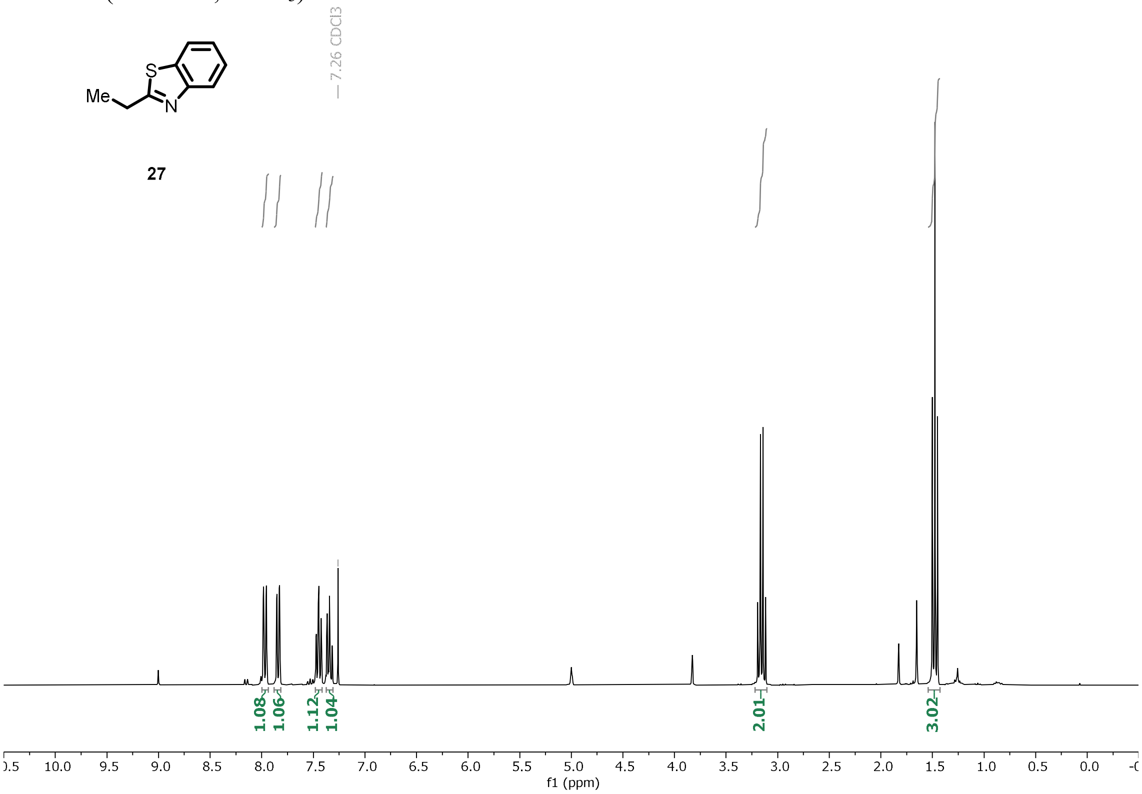

^{13}C NMR (101 MHz, $CDCl_3$) of **27**

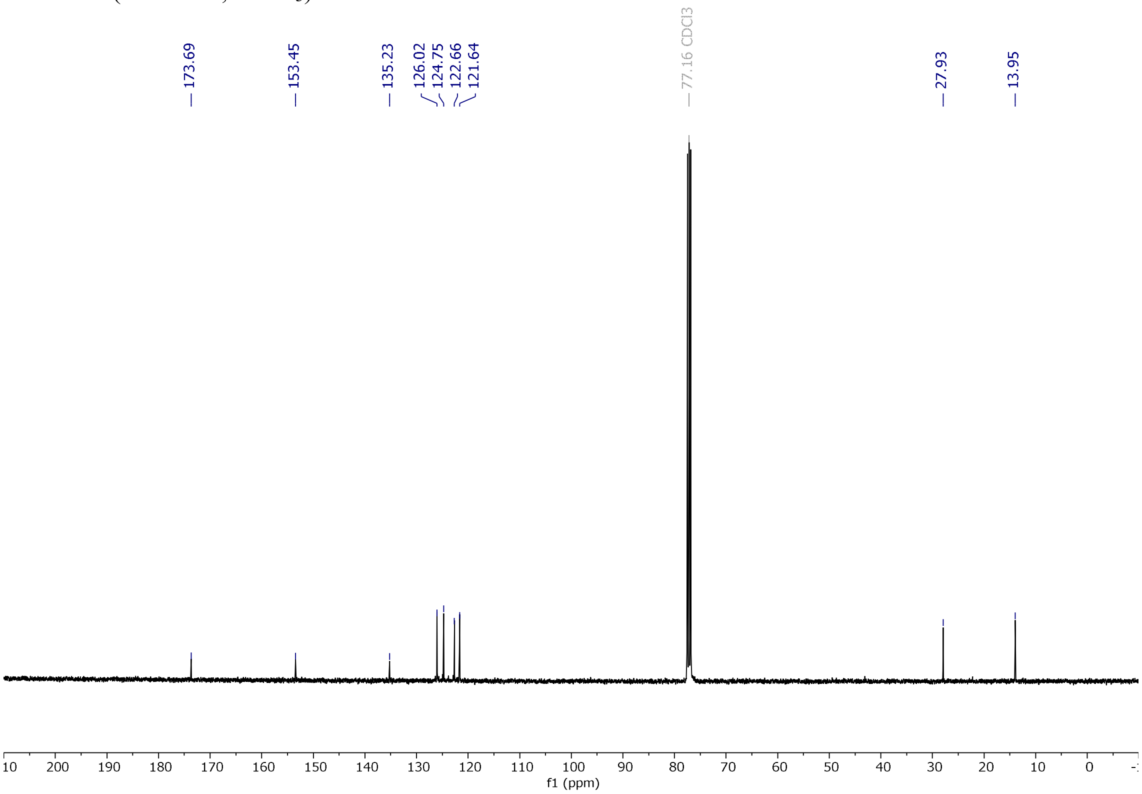

^{1}H NMR (400 MHz, $CDCl_3$) of **28**

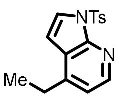

28

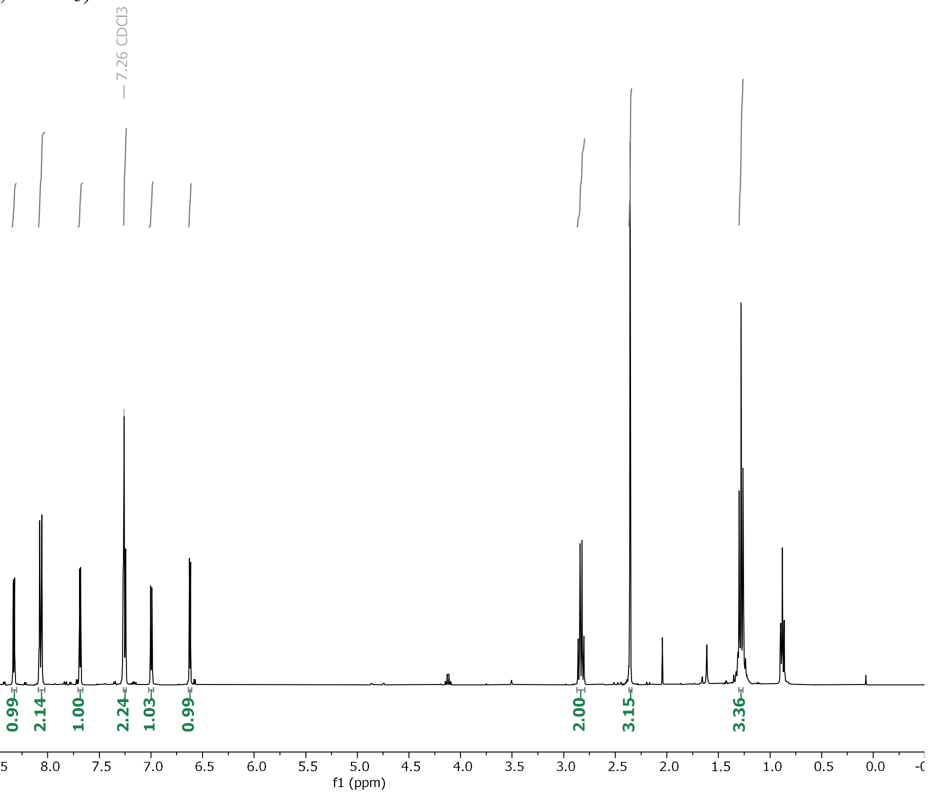

^{13}C NMR (101 MHz, $CDCl_3$) of **28**

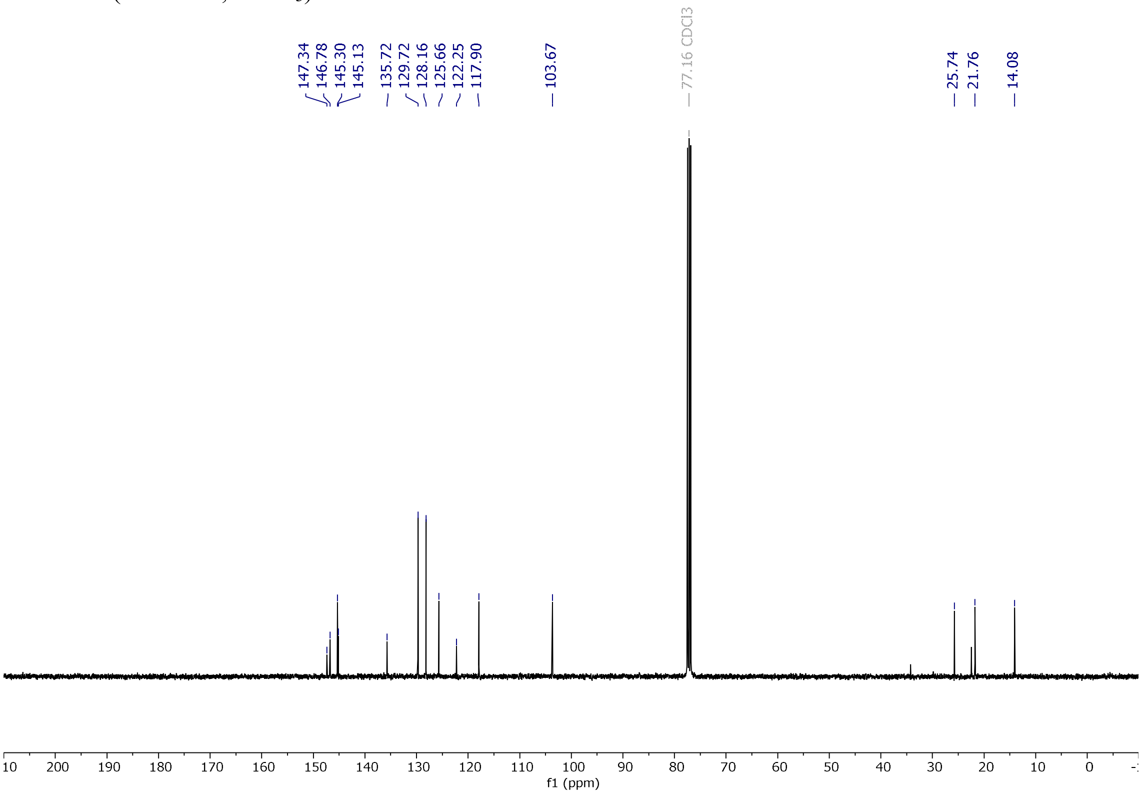

10. References

[1] T. Wan, L. Capaldo, G. Laudadio, A. V. Nyuchev, J. A. Rincón, P. García-Losada, C. Mateos, M. O. Frederick, M. Nuño, T. Noël, *Angew. Chem. Int. Ed.* **2021**, *60*, 17893–17897.

[2] I. B. Perry, T. F. Brewer, P. J. Sarver, D. M. Schultz, D. A. DiRocco, D. W. C. MacMillan, *Nature* **2018**, *560*, 70–75.

[3] R. Y. Nimje, D. Vytla, P. Kuppusamy, R. Velayuthaperumal, L. B. Jarugu, C. A. Reddy, N. K. Chikkananjaiah, R. A. Rampulla, C. L. Cavallaro, J. Li, A. Mathur, A. Gupta, A. Roy, *J. Org. Chem.* **2020**, *85*, 11519-11530.

[4] P. Xue, L. Li, N. Fu, *Org. Lett*. **2022**, *24*, 7595–7599.

[5] T. Wan, Z. Wen, G. Laudadio, L. Capaldo, R. Lammers, J. A. Rincón, P. García-Losada, C. Mateos, M. O. Frederick, R. Broersma, T. Noël, *ACS Cent. Sci*. **2022**, *8*, 51–56.

[6] F. Raymenants, T. M. Masson, J. Sanjosé-Orduna, T. Noël, *Angew. Chem. Int. Ed*. **2023**, *62*, e202308563.

[7] K. Luo, W.C. Yang, K. Wei, Y. Liu, J.K. Wang, L. Wu, *J. Org. Chem*. **2008**, *73*, 3604–3607.

[8] G. Xue, F. Xie, H. Liang, G. Chen, W. Dai, *Org. Lett*. **2022**, *24*, 5590–5595.

[9] K. Luo, W. C. Yang, K. Wei, Y. Liu, J.K. Wang, L. Wu, *Org. Lett*. **2019**, *21*, 7851–7856.

[10] C. Hansch, A. Leo, R. W. Taft, *Chem. Rev*. **1991**, *91*, 165–195.

[11] A. D. Becke, *J. Chem. Phys*. **1993**, *98*, 5648−5652.

[12] C. Lee, W. Yang, R. G. Parr, *Phys. Rev. B* **1988**, *37*, 785−789.

[13] F. Weigend, R. Ahlrichs, *Phys. Chem. Chem. Phys*. **2005**, *7*, 3297–3305.

[14] F. Weigend, Phys. *Chem. Chem. Phys*. **2006**, *8*, 1057–1065.

[15] Gaussian 16, Revision C.02, M. J. Frisch, G. W. Trucks, H. B. Schlegel, G. E. Scuseria, M. A. Robb, J. R. Cheeseman, G. Scalmani, V. Barone, G. A. Petersson, H. Nakatsuji, X. Li, M. Caricato, A. V. Marenich, J. Bloino, B. G. Janesko, R. Gomperts, B. Mennucci, H. P. Hratchian, J. V. Ortiz, A. F. Izmaylov, J. L. Sonnenberg, D. Williams-Young, F. Ding, F. Lipparini, F. Egidi, J. Goings, B. Peng, A. Petrone, T. Henderson, D. Ranasinghe, V. G. Zakrzewski, J. Gao, N. Rega, G. Zheng, W. Liang, M. Hada, M. Ehara, K. Toyota, R. Fukuda, J. Hasegawa, M. Ishida, T. Nakajima, Y. Honda, O. Kitao, H. Nakai, T. Vreven, K. Throssell, J. A. Montgomery, Jr., J. E. Peralta, F. Ogliaro, M. J. Bearpark, J. J. Heyd, E. N. Brothers, K. N. Kudin, V. N. Staroverov, T. A. Keith, R. Kobayashi, J. Normand, K. Raghavachari, A. P. Rendell, J. C. Burant, S. S. Iyengar, J. Tomasi, M. Cossi, J. M. Millam, M. Klene, C. Adamo, R. Cammi, J. W. Ochterski, R. L. Martin, K. Morokuma, O. Farkas, J. B. Foresman, and D. J. Fox, Gaussian, Inc., Wallingford CT, **2016**.

[16] S. Grimme, J. Antony, S. Ehrlich, H. Krieg, *J. Chem. Phys*. **2010**, *132*, 154104.

[17] A. Klamt, G. Schüürmann, *J. Chem. Soc. Perkin Trans*. *2* **1993**, 799–805.

[18] J. Tomasi, M. Persico, *Chem. Rev.* **1994**, *94*, 2027–2094.

[19] J. Andzelm, C. Kölmel, A. Klamt, *J. Chem. Phys*. **1995**, *103*, 9312–9320.

[20] V. Barone, M. Cossi, *J. Phys. Chem. A* **1998**, *102*, 1995–2001.

[21] M. Cossi, N. Rega, G. Scalmani, V. Barone, *J. Comput. Chem*. **2003**, *24*, 669–681.

[22] CYLview20; C. Y. Legault, Unversité de Sherbrooke: Sherbrooke, **2020** (http://www.cylview.org).

[23] P. Vermeeren, T. A. Hamlin, F. M. Bickelhaupt, *Chem. Commun*. **2021**, *57*, 5880–5896.

[24] P. Vermeeren, S. C. C. van der Lubbe, C. Fonseca Guerra, F. M. Bickelhaupt, T. A. Hamlin, *Nat. Protoc.* **2020**, *15*, 649–667.

[25] F. M. Bickelhaupt, K. N. Houk, *Angew. Chem. Int. Ed*. **2017**, *56*, 10076–10086.

[26] F. M. Bickelhaupt, *J. Comput. Chem*. **1999**, *20*, 114–128.

[27] F. M. Bickelhaupt, E. J. Baerends, Kohn-Sham Density Functional Theory: Predicting and Understanding Chemistry in *Reviews in Computational Chemistry*; K. B. Lipkowitz D. B. Boyd, Wiley, Hoboken, **2000**, *15*, 1–86.

[28] T. A. Hamlin, P. Vermeeren, C. F. Guerra, F. M. Bickelhaupt, 8 Energy Decomposition Analysis in the Context of Quantitative Molecular Orbital Theory in *Complementary Bonding Analysis* 199–212 (De Gruyter, **2021**).

[29] R. van Meer, O. V. Gritsenko, E. J. Baerends, *J. Chem. Theory Comput*. **2014**, *10*, 4432–4441.

[30] T. A. Hamlin, D. Svatunek, S. Yu, L. Ridder, I. Infante, L. Visscher, F. M. Bickelhaupt, *Eur. J. Org. Chem.* **2019**, 378–386.

[31] E. van Lenthe, E. J. Baerends, *J. Comput. Chem*. **2003**, *24*, 1142–1156.

[32] G. te Velde, F. M. Bickelhaupt, E. J. Baerends, C. Fonseca Guerra, S. J. A. van Gisbergen, J. G. Snijders, T. Ziegler, *J. Comput. Chem*. **2001**, *22*, 931–967.

[33] C. Fonseca Guerra, J. G. Snijders, G. te Velde, E. J. Baerends, *Theor. Chem. Acc.* **1998**, *99*, 391–403.

[34] ADF2023.101, SCM Theoretical Chemistry, Vrije Universiteit: Amsterdam (The Netherlands), **2023**. http://www.scm.com.

[35] E. van Lenthe, E. J. Baerends, J. G. Snijders, *J. Chem. Phys*. **1993**, *99*, 4597–4610.

[36] E. van Lenthe, E. J. Baerends, J. G. Snijders, *J. Chem. Phys*. **1994**, *101*, 9783–9792.

[37] E. van Lenthe, A. Ehlers, E. J. Baerends, *J. Chem. Phys*. **1999**, *110*, 8943–8953.

[38] M. S. Oderinde, S. Jin, J. Das, C. Jorge, S. Yip; A. Ramirez, D.-R. Wu, Y. Li, J. Kempson, N. A. Meanwell, A. Mathur, T. G. M. Dhar, *ACS Catal.* **2022**, *12*, 12511–12520.
